# Structure Optimization of 12β-*O*-γ-Glutamyl Oleanolic Acid Derivatives Resulting in Potent FXR Antagonist/Modulator for NASH Therapy

**DOI:** 10.3390/ph16050758

**Published:** 2023-05-17

**Authors:** Hao Ma, Yunyang Bao, Shuaishuai Niu, Shaorong Wang, Yiming Li, Hongwei He, Na Zhang, Weishuo Fang

**Affiliations:** 1 State Key Laboratory of Bioactive Substances and Functions of Natural Medicines & Ministry of Health Key Laboratory of Biosynthesis of Natural Products, Institute of Materia Medica, Chinese Academy of Medical Sciences & Peking Union Medical College, 2A Nan Wei Road, Beijing 100050, China; mh346185553@gmail.com (H.M.); nss19970312@outlook.com (S.N.); purplewinds@126.com (S.W.); 2 Key Laboratory of Biotechnology of Antibiotics, The National Health and Family Planning Commission (NHFPC), Institute of Medicinal Biotechnology, Chinese Academy of Medical Sciences & Peking Union Medical College, Beijing 100050, China; 17714333891@163.com (Y.B.); lym_1103@126.com (Y.L.); hehwei@imb.pumc.edu.cn (H.H.); zhangna@imb.pumc.edu.cn (N.Z.)

**Keywords:** oleanolic acid, FXR modulator, gene regulation, liver cirrhosis

## Abstract

The farnesoid X receptor (FXR) plays a crucial role in regulating the metabolism of bile acids, lipids, and sugars. Consequently, it is implicated in the treatment of various diseases, including cholestasis, diabetes, hyperlipidemia, and cancer. The advancement of novel FXR modulators holds immense importance, especially in managing metabolic disorders. In this study, a series of oleanolic acid (OA) derivatives bearing 12β-*O*-(γ-glutamyl) groups were designed and synthesized. Using a yeast one-hybrid assay, we established a preliminary structure–activity relationship (SAR) and identified the most potent compound, **10b**, which selectively antagonizes FXR over other nuclear receptors. Compound **10b** can differentially modulate the downstream genes of FXR, including with the upregulation of the CYP7A1 gene. In vivo testing revealed that **10b** (100 mg·Kg^−1^) not only effectively inhibits lipid accumulation in the liver but also prevents liver fibrosis in both BDL rats and HFD mice. Molecular modeling indicated that the branched substitution of **10b** extends into the H11–H12 region of FXR-LBD, possibly accounting for its CYP7A1 upregulation, which is different from a known OA 12β-alkonate. These findings suggest that 12-glutamyl OA derivative **10b** represents a promising candidate for the treatment of nonalcoholic steatohepatitis (NASH).

## 1. Introduction

Nuclear receptors (NRs) belong to a group of ligand-dependent transcription factors, which regulate the expressions of downstream target genes involved in growth and development. The farnesoid X receptor (FXR) belongs to the nuclear receptor superfamily and is widely distributed in organs such as the liver, kidney, intestinal tract, and adrenal gland [1,2,3].

FXR plays an important physiological role in bile acid metabolism [4]. As the endogenous ligand, bile acid stimulates the FXR regulation of downstream genes, limiting its own synthesis and playing a negative feedback regulatory role. Chenodeoxycholic acid (CDCA, **1**), generated in the liver from cholesterol, is the physiological agonist for FXR [5]. In recent years, the structural modification of CDCA has led to the production of agonists targeting FXR, such as obeticholic acid (OCA, **2**), which has an agonistic activity of up to 100 nM [6]. OCA has received approval as a treatment option for primary biliary cholangitis (PBC) [7]. In addition, while it is under a phase III clinical investigation for NASH therapy [8], it also showed reverse effects as a full agonist, including liver damage [9,10,11] and hyperlipidemia [12,13]. In light of the side effects of FXR agonists, researchers have shifted their focus to partial agonists and antagonists, with an emphasis on selective FXR regulation on its downstream genes [14,15,16]. Such FXR ligands have been proposed as selective bile acid receptor modulators (SBARM) [17].

FXR antagonists have shown effectiveness in in vivo tests for cholestasis and hypercholesterolemia [9,18], hyperglycemia [14] and hyperlipidemia [14,19], and certain malignant tumors, such as pancreatic cancer and intestinal cancer [20,21]. Among them, guggulsterone (**3**) is the first natural compound reported to selectively regulate downstream FXR genes [22,23]. Guggulsterone competes with CDCA to bind the same pocket of FXR, thereby reducing CDCA-induced FXR activation [24]. An analogue of **3**, 16-dehydropregnenolone (DHP, **4**), it specifically targets FXR without affecting other nuclear receptors, demonstrating highly selective FXR antagonism [25]. Another FXR antagonist, Ivermectin (**5**), is also a selective downstream gene regulator of FXR [26]. It exhibits antidiabetic effects by enhancing insulin sensitivity and has fewer side effects.

**Figure 1:**
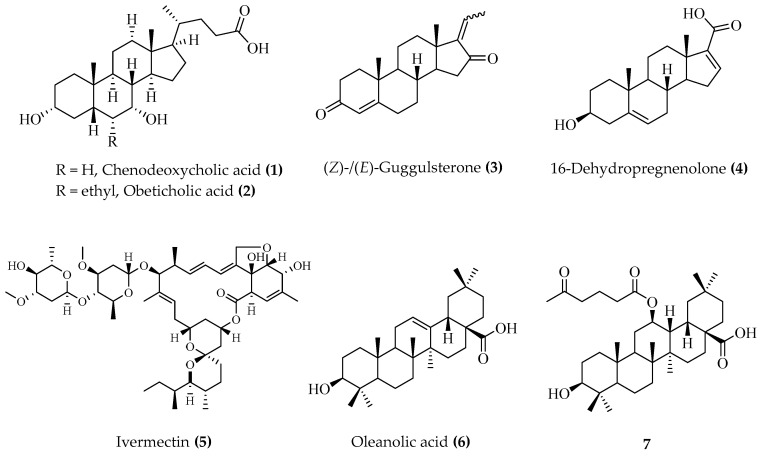
Structures of FXR agonists and antagonists.

Oleanolic acid (OA, **6**) is a widespread natural pentacyclic triterpene that has been found to antagonize FXR and exhibit selective downstream gene modulation [27]. OA binds to FXR-LBD, blocks its interaction with co-activator SRC-3, and inhibits the activity of FXR in a concentration-dependent manner, thereby exerting an FXR antagonistic effect. In the presence of CDCA, OA was observed to significantly reduce the CDCA-mediated expression of bile salt export protein (BSEP), partially reduce the expression of cholesterol 7 alpha-monooxygenase (CYP7A1), and slightly increase the expression of short heterodimer partner (SHP). The expression of organic solute transporter (OST)-β is not affected by OA [27]. The inhibition of OA on the expression of FXR target gene CYP7A1 reduces the biosynthesis of bile acids so as to ameliorate liver toxicity in the presence of a high concentration of bile acids [28]. Figure 1 showed the chemical structures of the FXR agonists/antagonists mentioned above.

In our previous study [19], we started with the binding model (PDB code 4WVD) of FXR with the antagonist ivermectin (**5**) as a reference to simulate the interaction between OA and FXR-LBD. We identified two unoccupied regions above and below the OA structure and used this information to facilitate the design and synthesis of a series of 12β-alkyl OA derivatives, resulting in a potent antagonist and modulator, compound **7** (Figure 1).

From the binding model of compound **7** with FXR-LBD, we envisioned that there is still an unoccupied space around the terminal region of the 12β-O-alkyl substituent. This region of the cavity comprises primarily hydrophobic amino acid residues, including Phe284, Ala291, Ile357, and Met450. Additionally, the area occupied by the 12β-alkyl side chain of compound **7** is similarly composed mainly of hydrophobic amino acid residues, such as Met328, Phe329, and Met365. Our prior research has shown that the inclusion of hydrophilic groups is unfavorable for binding, and practical observations have demonstrated a decrease in antagonistic activity [19]. We wonder if the occupancy of this area with new substituents could enhance the potency, and impact the modification of downstream gene expression patterns. 

**Figure 2:**
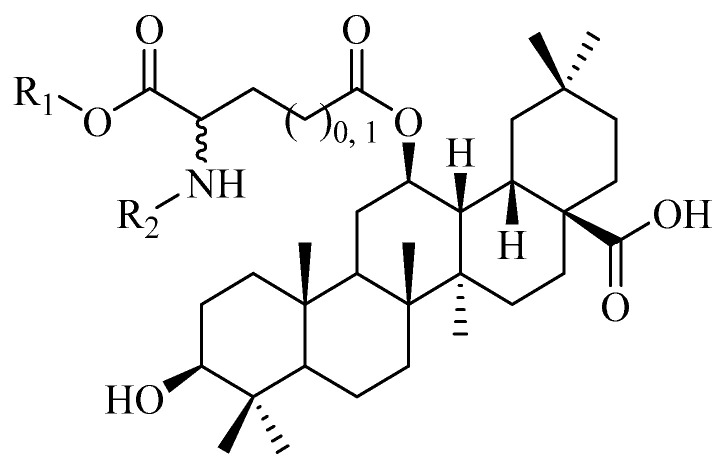
Design of 12β-*O*-β-aspartyl or 12β-*O*-γ-glutamyl OA derivatives.

Following preliminary screening, we discovered that Asp or Glu amino acid residues are fitting as 12β-substituents, as depicted in Figure 2. The main chains of Asp and Glu, which comprise 5–7 atoms, fulfill the requirement for a deep hydrophobic cavity. Additionally, the branched structure of their side chains allows for penetration into unoccupied cavities, which could more effectively occupy the hydrophobic pocket of FXR-LBD. Given that the binding pocket is hydrophobic, we deemed it necessary to introduce an alkyl group to the α-carboxyl/amino group of glutamic acid to form an ester, thereby entirely fulfilling this requirement. Furthermore, by observing the binding pocket, we anticipate an optimal volume group that can completely occupy the binding pocket and elicit the most potent FXR antagonistic activity. Here, we report the synthesis of such 12β-OA derivatives and there in vitro and in vivo effects. 

## 2. Results and Discussion

### 2.1. Chemical Synthesis and Antagonistic Activity of 12β-Aspartyl/Glutamyl OA Derivatives

**Scheme 1:**
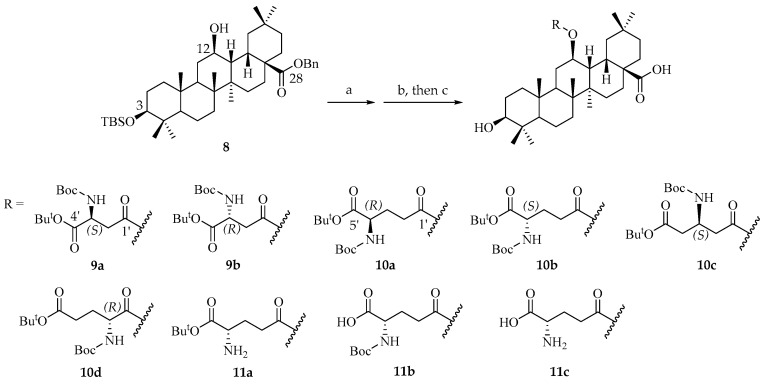
Preparation of 12β-*O*-β-aspartyl and 12β-*O*-γ-glutamyl OA derivatives. Reagents and conditions: (a) EDC, 4-DMAP, toluene, 60 °C overnight, yield: 69.0~99%; (b) AcOH/THF/H_2_O 13:7:3, 30 °C for 72 h, yield: 37.2~76.4%; (c) Pd/C, hydrogen (1 atm.), THF, 23 °C for 5 h, yield: 42.0~84.0%. Compound **11c** was obtained from **10b** via deprotection using trifluoroacetic acid under the following conditions: 20% TFA, DCM, 23 °C for 2 h, and a yield of 80.0%.

The modified intermediate 12β-OH OA (**8**) was obtained from OA in four consecutive steps, as shown in our previous work (refer to Scheme S1 in the supporting information) [19]. As depicted in Scheme 1, the 12β-OH of intermediate **8** was initially esterified by the corresponding amino acids using *N*,*N*′-diisopropylcarbodiimide (DIC) or 1-(3-dimethylaminopropyl)-3-ethylcarbodiimide hydrochloride (EDC) and 4-dimethylaminopyridine (DMAP). The 3-*O*-TBS group and the 28-*O*-benzyl (Bn) group were then removed via acetic acid hydrolysis and Pd/C catalyzed hydrogenolysis, respectively, to obtain the corresponding products. The final products containing the free carboxylic and amino group in the glutamic acid side chain were obtained under a 20% TFA condition.

The antagonistic activity of these compounds was tested at different concentrations using HEK-293T cells, and the IC_50_ values were calculated (Table 1). It is noteworthy that either the free carboxylic acid or the amine group in the 12β-*O*-γ-glutamyl ester significantly decreases the antagonistic activity of compounds. This finding aligns with the hydrophobic nature of the cavity surrounding the terminal of the 12β-alkyl esters. Of the two terminals, free α-amino (**11a**, 2.95 ± 0.68 μM) had a relatively minor effect on antagonistic activity compared to the α-carboxyl terminal (**11b**, 7.65 ± 0.35 μM). One possible explanation is that the presence of electron-withdrawing polar groups has a greater negative impact on the maintenance of antagonistic activity, while the presence of electron-donating polar group has a lesser effect on antagonistic activity. Compound **11c**, which has both terminals free, exhibited the weakest antagonistic activity at 21.3 ± 2.6 μM. This suggests that the presence of an alkyl group has a significant impact on maintaining antagonistic activity. Hence, we adhered to the 12β-OA derivatives bearing Boc-NH and *t*-butyl carboxyl ester groups at the terminal (**9a**,**b**; **10a**–**d**).

Among these compounds, **10d** showed the weakest antagonistic activity (IC_50_ 3.67 ± 0.39 μM), and **10b**, with the natural *L*-glutamyl substituents, is the most potent (IC_50_ 0.44 ± 0.15 μM), being 8.3 times more potent than **10d**. In general, 5-atom-length glutamyl substituents are better than 4-atom-length aspartyl substituents, and the Boc-NH at the terminal position is beneficial to the activity. Additionally, the (*S*)-configuration substituents showed better antagonistic activity than the (*R*)-configuration substituents (**9b** vs. **9a** and **10b** vs. **10a**). 

Based on these results, the 12β-*O*-γ-glutamyl OA derivative **10b**, bearing an (*S*)-glutamyl substituent, was chosen for further modification.

**Scheme 2:**
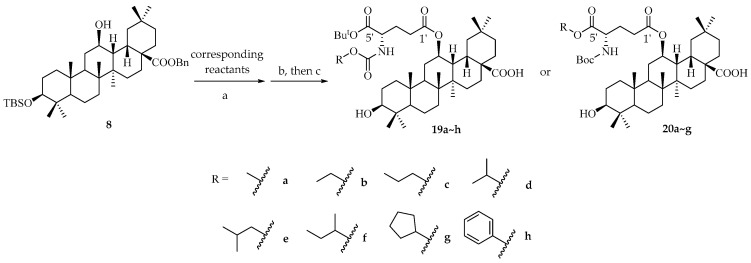
Preparation of 12β-*O*-γ-glutamyl OA derivatives **19a**~**h** and **20a**~**g**. Reagents and conditions: (a) DIC, 4-DMAP, toluene, 60 °C for 12 h, yield: >98%; (b) AcOH/THF/H_2_O 13:7:3, 30 °C for 72 h, yield: 63.6~88.8%; (c) Pd/C, hydrogen (1 atm.), MeOH, 23 °C for 6 h, yield: 55.7~97.1%.

We next investigated the impact of the volume of hydrophobic groups on the antagonistic activity by introducing methyl, ethyl, n-propyl, isopropyl, isobutyl, sec-butyl, cyclopentyl, and phenyl to 4′-carbamate (in place of *t*-butyl). To prepare the corresponding γ-glutamic acids, we derivatized the α-carboxyl as a *tert*-butyl ester and substituted the α-amino with different carbamates. 5-*O*-Benzyl-1-*O*-*tert*-butyloxycarbonyl-(2*S*)-2-aminopentanedioate hydrochloride (**12**) was treated with acid anhydride or chloroformate to generate the corresponding amide (**13a**~**h**), and the 5′-carboxyl benzyl ester was then removed by catalytic hydrogenation to afford corresponding O^α^, N^α^-protected γ-glutamic acids (**14a**~**h**, Scheme S2A). The conjugation of γ-glutamic acids with the key intermediate 12β-hydroxyl OA **8** and the removal of 3-*O*-TBS and 28-benzyl furnished targeted products **19a**–**h** are shown in Scheme 2.

In a similar approach, we prepared the OA 12β-*O*-γ-glutamic ester analogues by replacing the *t*-butyl of the 5′-carboxyl ester with various alkyl groups while retaining 4′-NHBoc substituents in **10b**. Starting from the glutamic acid mono-benzyl ester (**15**), a three step transformation, i.e., the free amino group was protected by Boc (**16**); the derivatization of the free carboxyl group as esters with various alkyl alcohols (**17a**~**h**); and the removal of the *O*-benzyl group were realized to furnish the corresponding γ-glutamic acids (**18a**~**h**, Scheme S2B). The conjugation of intermediates and the subsequent removal of 3- and 28-protective groups yields the targeted products **20a**–**g** (Scheme 2). However, **20h** bearing a 5′-carboxyl benzyl ester group was not obtained as the phenyl ester was also removed under catalytic hydrogenation.

Among products **19a**–**h**, there was no significant difference in antagonistic activity observed when the methoxycarbonyl or ethoxycarbonyl group was added to the 4′-amino of the glutamyl substituent. Compounds **19c** and **19d**, containing n-propoxycarbonyl and isopropoxycarbonyl groups, exhibited similar activity to that of **10b** at 0.4–0.5 μM. As the size of the group increased, the antagonistic activity clearly decreased (**19e**–**g**). The weakest activity is observed for compound **19h** (phenoxycarbonyl group, 2.22 ± 0.55 μM). Compounds **20a**–**g** showed a situation similar to that of series **19**. When the free carboxyl of γ-glutamyl is substituted by isopropyl or isobutyl, the corresponding OA derivatives show the best antagonistic activity (IC_50_ 0.5 μM). The further reduction or increase in the size of substituents will decrease the antagonistic activity significantly.

In summary, we prepared 12β-*O*-β-aspartyl and 12β-O-γ-glutamyl OA derivatives via esterification with their β- and γ-carboxyl groups and found that the (*S*)-configurations of 12β-substituents were more effective in antagonistic activity than the (*R*)-configurations. The best compound was **10b**, which bears the 4′-*N*-Boc-5′-*tert*-butyl-*L*-glutamyl substituent at the 12β position. However, the free 4′-amino or 5′-carboxyl group significantly decreased the activity. After some attempts to improve the potency of **10b** by introducing different size groups, the most potent compounds, **19c** and **19d**, were still less active than **10b**. Therefore, compound **10b** was selected for further study.

### 2.2. Compound ***10b*** Is a Selective FXR Antagonist Differentially Regulating the FXR Downstream Genes

**Figure 3:**
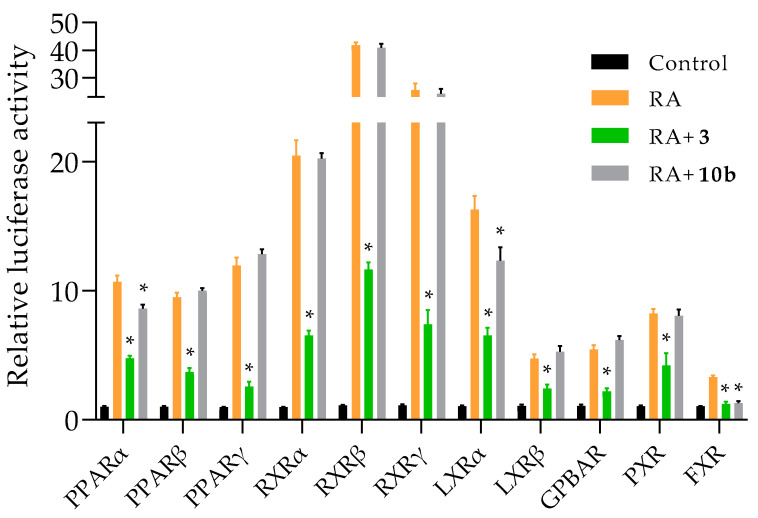
Compound **10b** selectively antagonized FXR. The impact of compound **10b** on various NRs was evaluated using a yeast one-hybrid assay in HEK-293T cells. The cells were transiently co-transfected with a fusion plasmid of Gal4 DBD-NR-LBD, which included PPARα, PPARβ, PPARγ, RXRα, RXRβ, RXRγ, LXRα, LXRβ, GPBAR, PXR, and FXR, in combination with the UAS-TK-Luc reporter. Positive controls, such as PPARα agonist fenofibrate, PPARβ agonist GW0742, PPARγ agonist rosiglitazone, RXR agonist LG100064, LXR agonist GW3965, GPBAR agonist OCA, PXR agonist SR12813, and FXR agonist CDCA (50 μM), were utilized. The values are expressed as the means ± SE of three independent experiments. *: *p* < 0.05 vs. RA (relative agonist).

Next, compound **10b**, along with the well-recognized FXR antagonist guggulsterone (**3**), were tested against a range of metabolic NRs, including RXRα, RXRβ, RXRγ, LXRα, LXRβ, PXR, PPARα, PPARβ, PPARγ, and GPBAR (Figure 3). Most NRs were antagonized by compound **3**, whereas compound **10b** showed potent antagonistic effects (>90%) against FXR and mild antagonistic effects (10–20%) against LXRα (activated by GW3965) and PPARα (activated by fenofibrate). Additionally, compound **10b** showed no agonistic effect for any tested NRs or GPBAR. These results indicate that compound **10b** is a selective FXR antagonist.

**Figure 4:**
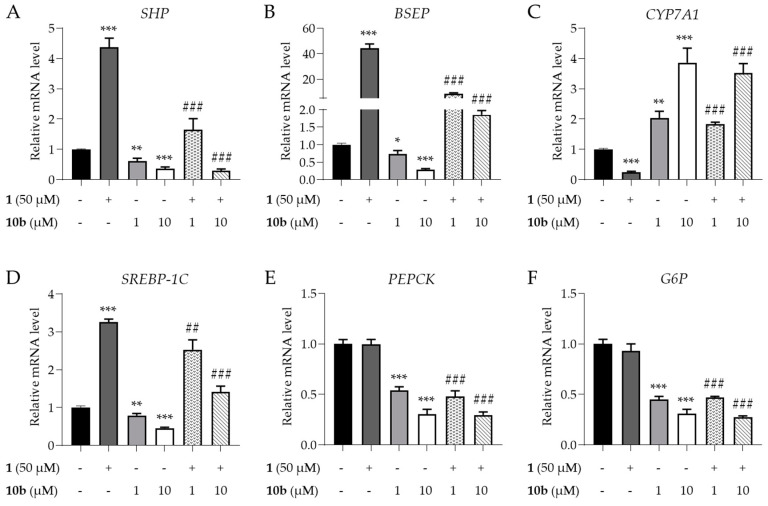
Compound **10b** regulated expression of FXR downstream genes. (**A**) Relative mRNA level of SHP; (**B**) Relative mRNA level of BSEP; (**C**) Relative mRNA level of CYP7A1; (**D**) Relative mRNA level of SREBP1c; (**E**) Relative mRNA level of PEPCK; (**F**) Expression level of G6Pase. HepG2 cells were seeded in a 6-well plate and treated with DMSO and varying concentrations of compound **10b** for 24 h with or without 50 μM of **1**. The mRNA levels of SHP, BSEP, CYP7A1, SREBP1c, PEPCK, and G6Pase were quantified and normalized to β-actin expression. The results, expressed as mean ± SE of three independent experiments, indicate statistical significance as *: *p* < 0.05, **: *p* < 0.01, and ***: *p* < 0.001 vs. control and *p* < 0.01, and ##: *p* < 0.01, ###: *p* < 0.001 vs. **1** (50 μM).

Further mRNA expression assays were conducted to test the regulation of FXR downstream genes by compound **10b** (Figure 4). The activation of FXR with the endogenous agonist CDCA (**1**, 50 μM) led to an increase in the expression of the small heterodimeric partner (SHP) and the bile salt export pump (BSEP). In contrast, it resulted in a decrease in the expression of sterol regulatory element binding protein 1c (SREBP1c) and cholesterol 7α-hydroxylase (CYP7A1), which are known to be suppressed by SHP activation [29]. Compound **1** showed no significant effect on key genes in glucose metabolism, phosphoenolpyruvate carboxykinase (PEPCK), and glucose-6-phosphatase (G6Pase). 

In the presence and absence of compound **1**, 10 μM and 1 μM of compound **10b** inhibit the expression of SHP, BSEP, and SREBP1c significantly (Figure 4A,B,D), while increasing the expression of CYP7A1 (Figure 4C). In our previous study, we found that compound **7** downregulated the CYP7A1 gene [19]. This finding differs from our previous results on the gene regulation of the OA derivative and motivates us to explore whether **10b** is different from **7** in lipid or glucose metabolism by performing crosstalk with bile acid metabolism [30]. Meanwhile, 10 μM and 1 μM of compound **10b** inhibited the expression of PEPCK and G6Pase significantly (Figure 4E,F). 

With all above results, compound **10b** could be considered an FXR modulator that is different from either a canonical agonist or antagonist [31]. It is worth mentioning that its gene regulation pattern is quite different from its close structural analogue **7** (reported in ref. [11]), which significantly reduces CYP7A1 as well as PEPCK and G6Pase expressions.

### 2.3. The Expressions of Liver Fibrosis-Related Genes Were Affected Significantly by Compound ***10b***

After finding that **10b** inhibits the lipid metabolism-regulating gene SHP, as well as cholesterol metabolism-related SREBP and CYP7A1 expressions, we sought to exploit its therapeutic potential in metabolic diseases, such as nonalcoholic fatty liver disease (NAFLD) and its advanced stage, nonalcoholic steatohepatitis (NASH). 

The therapeutic approach to NASH is multifaceted, encompassing lifestyle adjustments such as weight loss and physical exercise, as well as pharmacological interventions on the amelioration of hepatic lipid accumulation, inflammation, oxidative stress, and fibrosis [32]. In addition, to counteract lipid toxicity contributing to NAFLD and NASH, inflammation and fibrosis, the key drivers of disease progression from NAFLD to NASH, should also be addressed as liver fibrosis raises liver-related morbidity and mortality significantly [33]. Therefore, **10b** is evaluated in both high-fat-diet (HFD) mice and bile-duct-ligation (BDL) rats to assess its effect on the reduction in lipid toxicity, inflammation, and fibrosis.

Activated hepatic stellate cells (HSCs) play a crucial role in the onset and progression of liver fibrosis by generating fibrogenic factors and ECM proteins [34]. While FXR expression in human HSCs is relatively low, its significance in HSC biology has been demonstrated. The FXR-SHP regulatory cascade has been shown to impede HSCs and foster the resolution of liver fibrosis. The activation of FXR thwarts HSC activation, and its absence in mice intensifies hepatic inflammation and fibrosis [35]. Researchers have leveraged this pathway by developing orally available FXR agonists, which are undergoing clinical trials [36].

**Figure 5:**
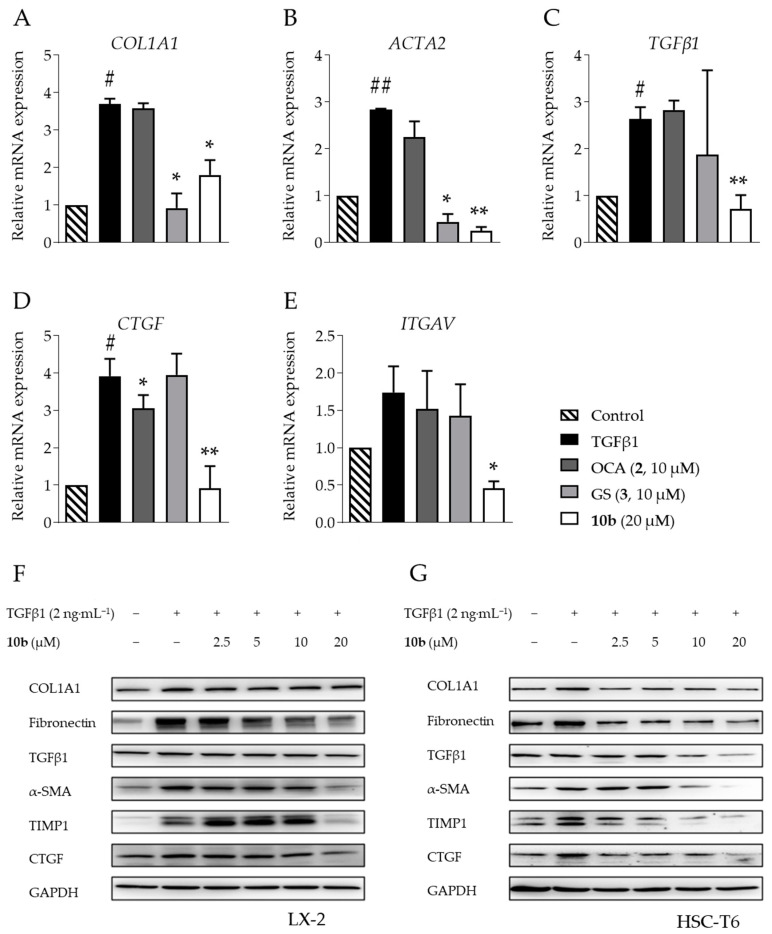
Compound **10b** clearly demonstrated an inhibitory effect on fibrogenic markers. (**A**–**E**) The LX-2 cells underwent a 24 h treatment of 2 ng·mL^−1^ TGFβ1, with or without varying concentrations of candidate compounds, following FBS starvation. Results showed that compound **10b** significantly repressed mRNA expression of fibrogenic genes in LX-2 cells. (**F**,**G**) Compound **10b** effectively suppressed expression of fibrosis marker proteins in both LX-2 cells and HSC-T6 cells. *GAPDH*/GAPDH was used as the loading control. The mean ± SD values were obtained from three independent assays. Significance was indicated by #: *p* < 0.05 and ##: *p* < 0.01 for results that were significantly different from the control group and *: *p* < 0.05 and **: *p* < 0.01 for results that were significantly different from the TGFβ1 treatment groups. ANOVA was followed by Tukey’s test for statistical analysis.

In hepatic stellate cell line LX-2, compound **10b** (20 μM) was found to significantly decrease the mRNA expressions of liver fibrosis marker genes such as collagen type I α-1 (COL1A1), actin α-2 (ACTA2), transforming growth factor β-1 (TGFb1), connective tissue growth factor (CTGF), and integrin α-V (ITGAV) induced by 2 ng·mL^−1^ of TGFβ1, as shown in Figure 5A–E. In contrast, OCA (**2**, 10 μM) only decreased the expression of CTGF, and guggulsterone (**3**, 10 μM) significantly decreased the expressions of COL1A1 and ACTA2.

Next, compound **10b** was investigated at protein level for its inhibitory effect on liver fibrosis markers. The results demonstrated in Figure 5F,G showed that 20 μM of compound **10b** reduced the content of liver-fibrosis-associated proteins TGFβ1, a-SMA, and TIMP1. Therefore, it has a good anti-hepatic fibrosis effect. The regulation of FXR by **10b** can affect the advancement of liver fibrosis. Based on the above results, compound **10b** was prepared for in vivo investigation.

Prior to the animal test, the stability of compound **10b** in biological environments was assessed by its incubation with mouse plasma at 37 °C. The content of **10b** remained almost unchanged by 48 h (Table S1), indicating that its stability is suitable for further in vivo biological assays.

### 2.4. Compound ***10b*** Attenuated Liver Injury and Hepatic Fibrosis in BDL Rats

A bile duct ligation (BDL) rat model was utilized to probe the liver fibrosis amelioration effects of compound **10b** in vivo. BDL induces cholestasis in the rat liver, leading to liver damage and inflammation and ultimately resulting in liver fibrosis. After the BDL procedure, the levels of serum biochemical markers, such as aspartate aminotransferase (AST), alanine aminotransferase (ALT), alkaline phosphatase (ALP), total bile acid (TBA), and total bilirubin (TBiL), significantly increased compared to the levels in the sham group. The oral administration of **10b** at 100 mg·kg^−1^ for 14 consecutive days effectively decreased the levels of AST, ALT, TBA, and TBiL to varying degrees (Table S2), indicating a reduction in liver injury and hepatic fibrosis.

**Figure 6:**
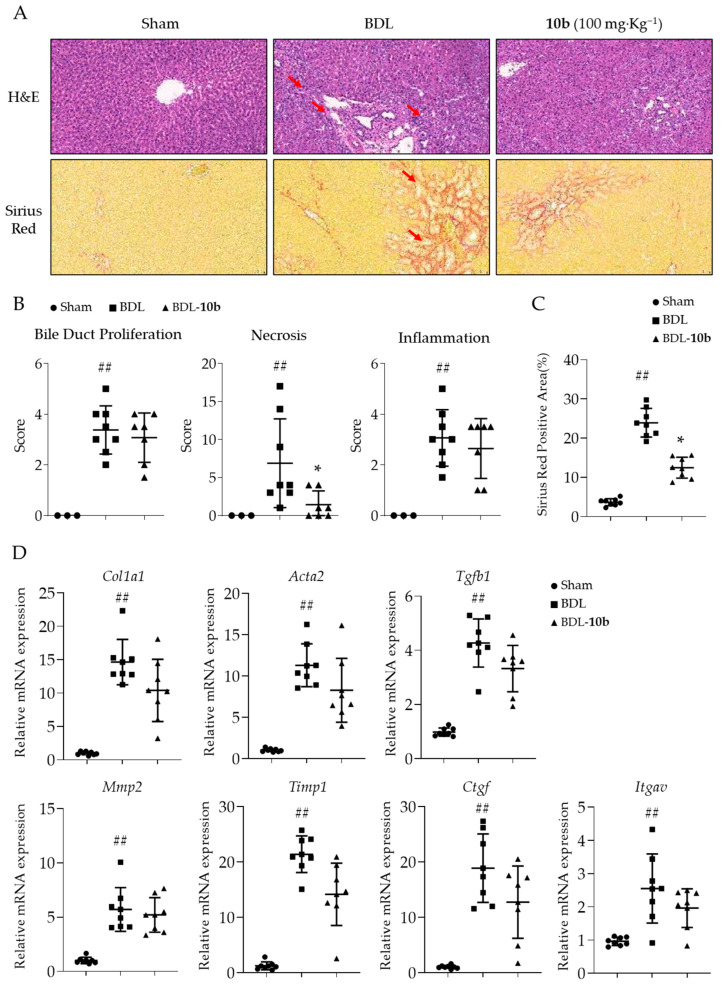
Compound **10b** attenuated BDL-induced liver injury in rats. (**A**) Liver histopathology was observed by H&E and Sirius red staining (magnification 400×; top panel). The red arrows indicated the area of bile duct hyperplasia, accompanied by infiltration of monocytes, structural dysfunction, and proliferation of fibroblasts in the ductal region (H&E). The red arrows highlighted the location of collagen fiber accumulation in the portal area (Sirius red). (**B**,**C**) Blinded quantitative assessment of liver bile duct proliferation, necrosis, and inflammation. Additionally, the percentage of Sirius red positively strained areas. (**D**) mRNA expressions of fibrogenic genes in rat liver samples. *Gapdh* served as the loading control. The values are expressed as the mean ± SD. ##: *p* < 0.01, significantly different from sham group; *: *p* < 0.05, significantly different from BDL-NS group in; ANOVA, followed by Tukey’s test.

Hematoxylin and eosin (H&E) staining revealed significant bile duct hyperplasia and parenchymal necrosis in BDL rat livers, accompanied by inflammation. The administration of compound **10b** substantially reduced these pathological impairments, as demonstrated in Figure 6. In a blinded assessment, the necrosis scores of the **10b**-treated rats were significantly lower than those of the BDL rats (Figure 6B). The collagen-specific Sirius red staining results also showed that liver collagen deposition was more pronounced in the BDL group, including pericellular bridging fibrosis, compared to the normal saline group. However, compound **10b** intervention significantly attenuated collagen accumulation (Figure 6A).

To assess the antifibrotic effect of compound **10b** in rat liver, the expression of hepatic fibrosis markers including *Col1a1*, *Acta2*, *Tgfb1*, *Mmp2*, *Timp1*, *Itgav*, and *Ctgf* were detected. qRT-PCR analysis revealed that the oral administration of **10b** reduced the expression of these fibrogenic genes (Figure 6C), indicating that compound **10b** has an antifibrotic effect on the cholestatic rat model.

Although the BDL model is not a general fatty liver model, bile duct ligation still causes an increase in total triglyceride (TG) and total cholesterol (TC) in rat serum. In this model, the oral administration of **10b** reduced TG and TC in rat serum (Table S2). Therefore, we further investigated the role of **10b** in the HFD mice model.

### 2.5. Compound ***10b*** Ameliorated Hepatic Steatosis and Liver Injury in HFD Mice

Considering that **10b** reduced TC and TG levels in rat serum and inhibited some FXR downstream lipid regulation genes, we utilized a mouse NASH model induced by a CDAHFD diet to investigate the impact of **10b** on the NASH process. The CDAHFD diet is a high-fat diet deficient in choline and methionine, which rapidly induces lipid accumulation in mice livers and leads to NASH. Choline is a crucial component in the formation of very low-density lipoprotein particles. A lack of choline blocks the outward transport of lipids from the liver and, when combined with high-fat feeding, can result in the accumulation of lipids in the liver of mice and induce NASH with liver fibrosis.

**Figure 7:**
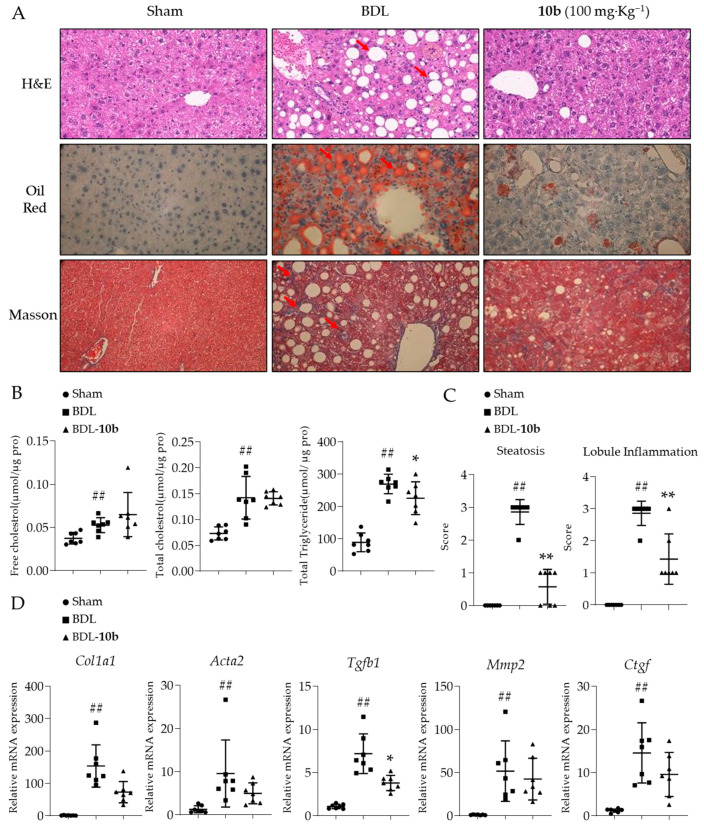
Compound **10b** attenuated CDAHFD-induced NASH in mice. (**A**) Liver histopathology was observed by H&E, Oil Red, and Masson’s staining (magnification 400×; top panel). The red arrows indicated the accumulation of lipid droplets in the liver and the recruitment and infiltration of inflammatory cells (H&E). The red arrows highlighted the accumulation of lipid droplets in the liver (Oil red). The red arrows indicated the presence of a purple fibrous area stained by Masson’s staining, indicating the site of collagen deposition resulting from liver fibrosis (Masson). (**B**) The contents of total triglyceride, total cholesterol, and free cholesterol in the liver of NASH mice. (**C**) Blinded quantitative assessment of liver steatosis and liver lobule inflammation. (**D**) mRNA expressions of fibrogenic genes in mice liver samples. *Gapdh* served as the loading control. The values are expressed as the mean ± SD. ##: *p* < 0.01, significantly different from Ctrl. Group; *: *p* < 0.05, **: *p* < 0.01, significantly different from Model-N.S. group; ANOVA, followed by Tukey’s test.

In mice fed with a CDAHFD diet for 16 weeks, the serum ALT, AST, ALP, and LDH levels were significantly elevated (Table S3). The staining of the pathological section revealed that the mice developed noticeable NASH (Figure 7A). Despite the blockade of the outward transport of TG, the serum TG levels still decreased, and **10b** reduced the TG content in the mice serum (Table S3). Meanwhile, we found a significant increase in the content of TG, TC, and free cholesterol (FC) in the mice livers of the model group (Figure 7B).

FC has been identified as a major contributor to steatosis and inflammatory damage in the liver. We found that **10b** reduced total triglyceride content in the liver but had no significant effect on TC or FC. To better evaluate the severity of the model and the efficacy of the compound, we also performed an NAS score on liver tissue sections to investigate steatosis and hepatic lobular inflammation. Compound **10b** improved intrahepatic steatosis and hepatic lobular inflammation, demonstrating its ability to inhibit liver fat accumulation, and reduce resulting inflammatory infiltration (Figure 7C).

To further investigate the anti-fibrotic effect of compound **10b** in NASH, we also measured the expression of liver fibrosis-related genes in animal liver samples. We found that **10b** can inhibit the mRNA expression of liver fibrosis-related genes in the NASH mouse model (Figure 7D). 

We conducted a further comparison of the therapeutic effect of compound **10b** with similar triterpenoid compounds that have been previously reported. Betulinic acid (BA) is a natural pentacyclic triterpenoid compound that has demonstrated effectiveness in treating NASH [37]. BA has a hydrogen atom substitution at the 12th position, which makes its structure more lipophilic. In HFD mice, a dose of 100 mg·Kg^−1^ of BA as the FXR agonist can reduce liver TG and TC levels, which is similar to the reduction achieved by **10b** for TG. Ilexsaponin A1 (IsA) is a group of 28-carboxyl triterpenoid derivatives that are glycosylated. Structurally, it has better hydrophilicity. A dose of 120 mg·Kg^−1^ of IsA showed a similar effect in reducing serum and liver TG as **10b** while also reducing liver TC levels [38]. The liver histopathological examination revealed that **10b** exhibited a NASH therapeutic effect similar to that of BA and IsA.

Taken together, **10b** effectively inhibits liver lipid accumulation and prevents liver fibrosis in both BDL rats and HFD mice.

### 2.6. Analysis of the Ligand-FXR-LBD Binding for ***10b*** and Analogues ***7*** and ***21***

The binding modes of three OA derivatives, compounds **7**, **10b**, and **21** (synthesized in our previous work), were further analyzed to understand the relationship between the structural difference of ligand and differential FXR gene expressions. From the structural point of view, **10b** is apparently different from **7** and **21** in that it possesses a 4′-BocNH group as a branch to the linear alkyl ester chain at 12β position of OA.

**Figure 8:**
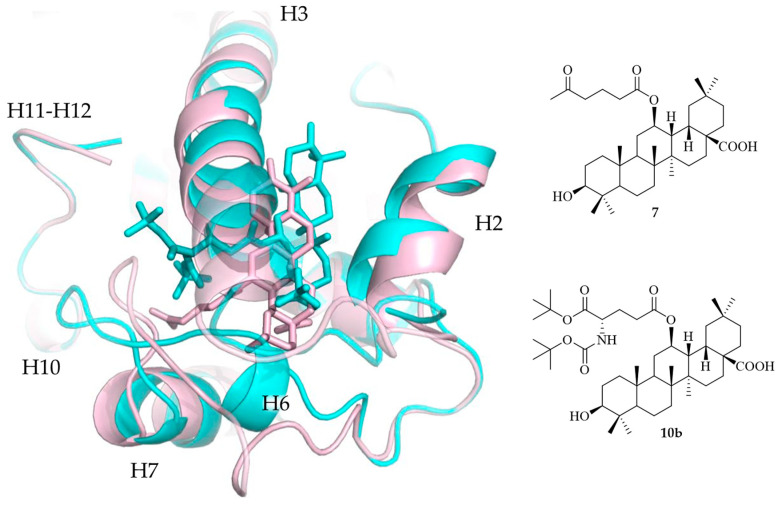
Binding model of compound **7** (pink) and **10b** (cyan) as ligands interacting with FXR-LBD.

For all three compounds, the polar groups at the 3- and 28-positions formed hydrogen bonds with amino acid residues on FXR-LBD, while 12β substituents interact with mainly hydrophobic ones. Compared with compound **7** and **21**, **10b** has a bulky substitution at the 12β position, filling in more cavities within the binding site formed by Leu287, Ala 291, Val325, Met328, Phe329, Ile352, Ile357, Ile362, Met365, His447, and Met450 (Figure S2A). More specifically, the *tert*-butyl group of **10b** extended to the H11–H12 region and increased the distance between H11 and H12 (Figure 8), a region responsible for the gene expression regulation by binding to co-activators and co-suppressors, which impact the downstream gene expression. In contrast, ligands **7** and **21** extended to a region that deviated from H11–H12 (Figure 8, S2B) and exhibited an insignificant difference in their binding modes.

Furthermore, we tried to clarify the distinctions between the mentioned three compounds in terms of their binding to FXR-LBD, with a focus on ΔG [39]. The resulting binding free energy ΔG_bind_ values are presented in Table 2. An analysis of the decomposition of the binding free energy ΔG_bind_ indicates that the lipophilic interaction (ΔG_Lipo_) and the van der Waals interaction (ΔG_vdW_) are the primary contributors. Among these three compounds, **10b** exhibits superior performance (ΔG_Lipo_ −61.34 Kcal·mol^−1^, ΔG_vdW_ −87.73 Kcal·mol^−1^) due to its larger hydrophobic substitution at the 12β position. Compound **7** and compound **21** differ only by a single carbonyl group at the end of the 12β-alkyl side chain. While compound **7** performs better in terms of the van der Waals interaction, this advantage is offset by the need to overcome the greater solvation (ΔG_Solv_GB_) caused by the presence of the carbonyl group. Additionally, compound **21** exhibits greater lipophilicity, and any potential hydrogen bonds contribute only marginally to its overall effect.

## 3. Material and Methods

### 3.1. Chemistry

General Methods: Commercially sourced solvents and starting materials, which included anhydrous solvents and chemicals, were utilized without undergoing any further purification. The reactions involving air- or moisture-sensitive reactants were taken into an argon atmosphere, and the solutions were transferred through oven-dried glassware and syringes. TLC analysis was carried out on silica gel plates (Huayao New Material Technology, Qingdao, China). Column chromatography was carried out on silica gel 200~300 mesh (Innochem Technology, Beijing, China). Nuclear magnetic resonance (^1^H NMR and ^13^C NMR) spectra were recorded in CDCl_3_ or methanol-*d*_4_ on a Bruker Avance 400 spectrometer (Bruker Company, Karlsruhe, Germany). The chemical shifts (*δ* values) and coupling constants (*J* values) are provided in ppm and Hz, respectively, and tetramethylsilane (TMS) serves as the internal standard. The splitting patterns are classified as s for singlet, d for doublet, t for triplet, q for quadruplet, m for multiplet, dd for double doublet, and brs for broad singlet. Room temperature was controlled at 23 ± 2 °C based on laboratory temperature.

Prepare conditions of 12β-aspartyl/glutamyl OA derivatives. To a solution of key intermediate **8** (250 mg, 0.37 mmol, and 1.0 eq.) in 2 mL of toluene, 5 mL of EDC (1.47 mmol, 282 mg, and 4.0 eq.) and 4-DMAP (0.07 mmol, 9.0 mg, and 0.2 eq.) in toluene solution was added slowly. Then, the modified glutamic acid (4.0 eq.) was added in the reaction mixture and heated to 60 °C for stirring for 12 h. After TLC showed that the raw materials were completely consumed, the mixture was diluted with water and extracted with ethyl acetate. The organic layers were combined and dried over sodium sulfate and concentrated in vacuum to obtain crude product, and then directly subjected to flash chromatography on silica gel using PE/EtOAc (20:1~10:1) to obtain intermediates, which were directly used in the next step.

The intermediates obtained in the above steps were dissolved in 15 mL of a mixture of AcOH/THF/H_2_O 13:7:3 and stirred at 35 °C for 72 h. After TLC showed that most of the raw materials were consumed, NaHCO_3_ solution was added in the reaction mixture to remove acetic acid, and the solution was extracted with DCM. The organic layers were combined and dried over sodium sulfate and concentrated in vacuum to obtain crude product. Proceed to the next step without any further purification. The crude products were dissolved in 10 mL of methanol or THF, and 10% of the mass of palladium carbon was added, passing through hydrogen at 1 atmosphere. The reaction mixture was stirred at room temperature for 6 h. The reaction mixture was then filtered with diatomite and diluted with DCM. The organic layers were dried over sodium sulfate and concentrated in vacuum to obtain crude product and then directly subjected to flash chromatography on silica gel using PE/EtOAc (5:1~1:1). The organic layer was finally evaporated to dryness to obtain end products.

Benzyl 3β-(t-butyldimethylsiloxy)-12β-hydroxy-13H-olean-28-oate (**8**).

White solid 28 g, overall yield: 37.8%. ^1^H NMR (400 MHz, CDCl_3_) *δ* (ppm): 7.31–7.41 (m, 5H, Ph), 5.21 (d, 1H, *J* = 12.0 Hz, PhCH_a_), 5.06 (d, 1H, *J* = 12.0 Hz, PhCH_b_), 3.67 (ddd, 1H, *J* = 6.0, 10.0, 11.6 Hz, 12-H), 3.14 (dd, 1H, *J* = 4.8, 11.6 Hz, 3-H), 2.75 (dt, 1H, *J* = 5.2, 11.6 Hz, 18-H), 0.92 (s, 9H, 3Me), 0.88 (s, 9H, TBS-Bu^t^), 0.87 (s, 3H, Me), 0.78 (s, 3H, Me), 0.71 (s, 3H, Me), 0.60 (s, 3H, Me), 0.59 (d, 1H, *J* = 10.0 Hz, 5-H). ^13^C NMR (100 MHz, CDCl_3_) *δ* (ppm): 177.8, 136.5, 128.5, 128.4, 128.1, 79.3, 68.3, 66.0, 55.3, 49.1, 47.4, 43.2, 41.5, 40.5, 39.4, 38.6, 36.8, 36.1, 34.5, 33.4, 33.1, 32.6, 31.8, 31.4, 30.5, 28.7, 28.4, 27.7, 25.9, 23.4, 23.2, 18.4, 18.1, 17.8, 16.2, 15.9, 15.8, −3.7, −4.9. HRMS (ESI) *m*/*z* Calcd for C_43_H_70_O_4_NaSi 701.4936 (M + Na)^+^, found 701.4936.

3-hydroxy-12β-(3′-((tert-butoxycarbonyl)amino)-4′-(tert-butoxy)-L-aspartate)oxy-13H-olean-28-oic acid (**9a**).

White solid 61 mg, overall yield: 70.0%. ^1^H NMR (400 MHz, CDCl_3_) *δ* (ppm): 5.73 (d, 1H, *J* = 8.4 Hz, NH), 4.99–5.05 (m, 1H, 12-H), 4.47 (dt, 1H, *J* = 4.0, 8.4 Hz, H^α^Asp), 3.20 (dd, 1H, *J* = 4.0, 11.6 Hz, 3-H), 2.97 (dd, 1H, *J* = 4.4, 17.2 Hz, H_a_^β^Asp), 2.77 (dd, 1H, *J* = 4.4, 17.2 Hz, H_b_^β^Asp), 2.50 (dt, 1H, *J* = 4.0, 14.0 Hz, 18-H), 2.11 (dd, 1H, *J* = 4.8, 11.6 Hz, 13-H), 1.48 (s, 9H, Boc), 1.43 (s, 9H, Bu^t^), 1.00 (s, 3H, Me), 0.97 (s, 3H, Me), 0.92 (s, 3H, Me), 0.90 (s, 3H, Me), 0.87 (s, 3H, Me), 0.80 (s, 3H, Me), 0.74 (s, 3H, Me), 0.69 (d, 1H, *J* = 10.4 Hz, 5-H). ^13^C NMR (100 MHz, CDCl_3_) *δ* (ppm): 183.8, 170.5, 170.0, 155.8, 81.8, 79.6, 78.7, 72.6, 55.1, 50.3, 48.7, 47.2, 41.4, 40.5, 39.7, 38.8, 38.6, 37.3, 37.1, 36.0, 34.3, 33.2, 33.0, 32.3, 31.4, 30.5, 29.7, 28.8, 28.3, 28.0, 27.3, 27.1, 23.2, 22.7, 18.2, 17.7, 16.4, 15.7, 15.3. HRMS (ESI) *m*/*z* Calcd for C_43_H_72_NO_9_ 746.5202 (M + H)^+^, found: 746.5196.

3-hydroxy-12β-(3′-((tert-butoxycarbonyl)amino)-4′-(tert-butoxy)-D-aspartate)oxy-13H-olean-28-oic acid (**9b**).

White solid 53 mg, overall yield: 68.8%. ^1^H NMR (400 MHz, CDCl_3_) *δ* (ppm): 5.53 (d, 1H, *J* = 8.8 Hz, NH), 4.98–5.04 (m, 1H, 12-H), 4.42 (dt, 1H, *J* = 4.0, 8.8 Hz, H^α^Asp), 3.20 (dd, 1H, *J* = 4.0, 11.2 Hz, 3-H), 2.99 (dd, 1H, *J* = 4.4, 17.6 Hz, H_a_^β^Asp), 2.74 (dd, 1H, *J* = 4.4, 17.6 Hz, H_b_^β^Asp), 2.48 (dt, 1H, *J* = 4.4, 14.0 Hz, 18-H), 2.10 (dd, 1H, *J* = 4.7, 11.4 Hz, 13-H), 1.47 (s, 9H, Boc), 1.43 (s, 9H, Bu^t^), 1.00 (s, 3H, Me), 0.97 (s, 3H, Me), 0.91 (s, 3H, Me), 0.90 (s, 3H, Me), 0.82 (s, 6H, 2Me), 0.75 (s, 3H, Me), 0.66 (d, 1H, *J* = 10.0 Hz, 5-H). ^13^C NMR (100 MHz, CDCl_3_) *δ* (ppm): 184.0, 171.2, 170.1, 155.5, 82.1, 79.7, 78.7, 72.5, 55.2, 50.4, 48.8, 47.2, 41.4, 40.6, 39.7, 38.8, 38.5, 37.1, 36.9, 36.1, 34.3, 33.2, 33.1, 32.4, 31.3, 30.4, 29.7, 28.8, 28.3, 28.0, 27.9, 27.4, 27.2, 23.4, 22.9, 18.2, 17.6, 16.5, 15.8, 15.4. HRMS (ESI) *m*/*z* Calcd for C_43_H_72_NO_9_ 746.5202 (M + H)^+^, found: 746.5196.

3-hydroxy-12β-(4′-((tert-butoxycarbonyl)amino)-5′-(tert-butoxy)-D-glutamate)oxy-13H-olean-28-oic acid (**10a**).

White solid 53 mg, overall yield: 52.0%. ^1^H NMR (400 MHz, CDCl_3_) *δ* (ppm): 5.15 (d, 1H, *J* = 8.0 Hz, NH), 4.97–5.03 (m, 1H, 12-H), 4.22–4.24 (m, 1H, H^α^Glu), 3.19 (dd, 1H, *J* = 4.5, 11.4 Hz, 3-H), 2.50 (dt, 1H, *J* = 4.4, 11.6 Hz, 18-H), 2.43 (ddd, 1H, *J* = 6.0, 10.0, 16.4 Hz, H_a_^γ^Glu), 2.32 (ddd, 1H, *J* = 5.2, 10.4, 16.0 Hz, H_b_^γ^Glu), 2.13–2.21 (m, 1H, H_a_^β^Glu), 2.07 (dd, 1H, *J* = 4.8, 12.0 Hz, 13-H), 1.47 (s, 9H, Boc), 1.44 (s, 9H, Bu^t^), 1.01 (s, 3H, Me), 0.97 (s, 3H, Me), 0.92 (s, 3H, Me), 0.90 (s, 3H, Me), 0.82 (s, 3H, Me), 0.81 (s, 3H, Me), 0.75 (s, 3H, Me), 0.67 (d, 1H, *J* = 9.6 Hz, 5-H). ^13^C NMR (100 MHz, CDCl_3_) *δ* (ppm): 183.2, 172.6, 171.5, 155.5, 82.1, 79.7, 78.8, 71.9, 55.2, 53.3, 48.7, 47.2, 41.4, 40.6, 40.0, 38.9, 38.6, 37.1, 36.0, 34.3, 33.3, 33.2, 32.4, 31.4, 30.6, 30.5, 28.8, 28.4, 27.4, 27.2, 23.4, 22.9, 18.2, 17.8, 16.5, 15.8, 15.4. HRMS (ESI) *m*/*z* Calcd for C_44_H_74_NO_9_ 760.5358 (M + H)^+^, found: 760.5349.

3β-hydroxy-12β-(4′-((tert-butoxycarbonyl)amide)-5′-(tert-butoxy)-L-glutamte)oxy-13H-olean-28-oic acid (**10b**).

White solid 12 g, overall yield: 71.4%. [α${]}_{D}^{20}$ = +8.3° (c 1.2, CHCl_3_). ^1^H NMR (400 MHz, CDCl_3_) *δ* (ppm): 5.09 (d, 1H, *J* = 8.0 Hz, NH), 4.95–5.02 (m, 1H, 12-H), 4.16–4.22 (m, 1H, H^α^Glu), 3.20 (dd, 1H, *J* = 4.4, 11.6 Hz, 3-H), 2.50 (dt, 1H, *J* = 4.0, 14.0 Hz, 18-H), 2.31–2.45 (m, 2H, 2H^γ^Glu), 2.08 (dd, 1H, *J* = 4.0, 12.0 Hz, 13-H), 1.47 (s, 9H, Boc), 1.44 (s, 9H, Bu^t^), 1.01 (s, 3H, Me), 0.97 (s, 3H, Me), 0.92 (s, 3H, Me), 0.89 (s, 3H, Me), 0.82 (s, 3H, Me), 0.81 (s, 3H, Me), 0.75 (s, 3H, Me), 0.67 (d, 1H, *J* = 9.6 Hz, 5-H). ^13^C NMR (100 MHz, CDCl_3_) *δ* (ppm): 183.9, 172.6, 171.4, 155.4, 82.1, 79.8, 78.8, 72.0, 55.2, 53.6, 48.8, 47.2, 41.4, 40.6, 39.9, 38.8, 38.6, 37.1, 36.1, 34.3, 33.2, 32.4, 31.3, 30.7, 30.5, 28.8, 28.3, 28.0, 27.3, 27.1, 23.4, 22.8, 18.2, 17.7, 16.5, 15.9, 15.4. HRMS (ESI) *m*/*z* Calcd for C_44_H_74_NO_9_ 760.5358 (M + H)^+^, found: 760.5349.

3-hydroxy-12β-((S)-3′-((tert-butoxycarbonyl)amino)-5′-(tert-butoxy)-5-oxopentanoyl)oxy-13H-olean-28-oic acid (**10c**).

White solid 93 mg, overall yield: 46.0%. ^1^H NMR (400 MHz, CDCl_3_) *δ* (ppm): 5.37 (d, 1H, *J* = 8.8 Hz, NH), 4.98–5.04 (m, 1H, 12-H), 4.29 (br, 1H, H^α^Glu), 3.20 (dd, 1H, *J* = 4.4, 11.6 Hz, 3-H), 2.56–2.72 (m, 4H, 2CH_2_), 2.46 (dt, 1H, *J* = 4.0, 14.0 Hz, 18-H), 2.08 (dd, 1H, *J* = 4.8, 11.2 Hz, 13-H), 1.45 (s, 9H, Boc), 1.42 (s, 9H, Bu^t^), 1.00 (s, 3H, Me), 0.97 (s, 3H, Me), 0.91 (s, 3H, Me), 0.90 (s, 3H, Me), 0.83 (s, 3H, Me), 0.81 (s, 3H, Me), 0.75 (s, 3H, Me), 0.67 (d, 1H, *J* = 9.6 Hz, 5-H). ^13^C NMR (100 MHz, CDCl_3_) *δ* (ppm): 183.6, 171.4, 170.4, 154.9, 81.0, 79.3, 78.8, 72.2, 55.2, 48.8, 47.2, 44.5, 41.4, 40.6, 39.8, 39.7, 38.8, 38.6, 37.9, 37.0, 36.1, 34.2, 33.3, 33.1, 32.3, 31.3, 30.4, 28.8, 28.4, 28.1, 28.0, 27.4, 27.1, 23.4, 22.8, 18.2, 17.7, 16.5, 15.8, 15.3. HRMS (ESI) *m*/*z* Calcd for C_44_H_74_NO_9_ 760.5358 (M + H)^+^, found: 760.5349.

3-hydroxy-12β-(2′-((tert-butoxycarbonyl)amino)-5′-(tert-butoxy)-D-glutamate)oxy-13H-olean-28-oic acid (**10d**).

White solid 6 mg, overall yield: 10.8%. ^1^H NMR (400 MHz, CDCl_3_) *δ* (ppm): 5.04–5.11 (m, 2H, NH, 12-H), 4.27–4.32 (m, 1H, H^α^Glu), 3.19 (dd, 1H, *J* = 4.4, 11.6 Hz, 3-H), 2.46 (dt, 1H, *J* = 4.0, 13.6 Hz, 18-H), 2.29–2.41 (m, 2H, 2H^γ^Glu), 1.16–2.24 (m, 2H, 2H^β^Glu), 2.10 (dd, 1H, *J* = 4.4, 11.6 Hz, 13-H), 1.44 (s, 18H, 2Bu^t^), 1.01 (s, 3H, Me), 0.97 (s, 3H, Me), 0.93 (s, 3H, Me), 0.90 (s, 3H, Me), 0.82 (s, 3H, Me), 0.81 (s, 3H, Me), 0.76 (s, 3H, Me), 0.67 (d, 1H, *J* = 9.6 Hz, 5-H). ^13^C NMR (100 MHz, CDCl_3_) *δ* (ppm): 172.1, 172.0, 155.4, 80.5, 79.7, 78.8, 73.2, 55.1, 53.4, 48.5, 47.2, 41.4, 40.6, 40.1, 38.8, 38.5, 37.1, 35.8, 33.4, 33.1, 32.3, 32.2, 31.5, 30.5, 29.7, 28.5, 28.3, 28.1, 28.0, 27.3, 27.2, 23.8, 22.6, 18.2, 17.8, 16.5, 15.7, 15.3. HRMS (ESI) *m*/*z* Calcd for C_44_H_74_NO_9_ 760.5358 (M + H)^+^, found: 760.5352.

3-hydroxy-12β-((S)-4′-amino-5′-(tert-butoxy)-5-oxopentanoyl)oxy-13H-olean-28-oic acid (**11a**).

White solid 35 mg, overall yield: 49.8%. ^1^H NMR (400 MHz, methanol-*d*_4_) *δ* (ppm): 5.00–5.07 (m, 1H, 12-H), 3.58 (t, 1H, *J* = 6.4 Hz, H^α^Glu), 3.21 (dd, 1H, *J* = 4.8, 10.8 Hz, 3-H), 2.45–2.54 (m, 3H, 18-H, 2H^γ^Glu), 2.17 (dd, 1H, *J* = 4.8, 11.6 Hz, 13-H), 1.97–2.06 (m, 2H, 2H^β^Glu), 1.49 (s, 9H, Bu^t^), 1.03 (s, 3H, Me), 0.96 (s, 3H, Me), 0.91 (s, 3H, Me), 0.89 (s, 3H, Me), 0.84 (s, 6H, 2Me), 0.74 (s, 3H, Me). HRMS (ESI) *m*/*z* Calcd for C_39_H_66_NO_7_ 660.4834 (M + H)^+^, found: 660.4826.

3-hydroxy-12β-((S)-4′-((tert-butoxycarbonyl)amino)-4′-carboxybutanoyl)oxy-13H-olean-28-oic acid (**11b**).

White solid 77 mg, overall yield: 85.0%. ^1^H NMR (400 MHz, methanol-*d*_4_) *δ* (ppm): 4.95–5.00 (m, 1H, 12-H), 4.26 (br, 1H, H^α^Glu), 3.12 (dd, 1H, *J* = 5.6, 10.4 Hz, 3-H), 2.16–2.60 (m, 4H, 2H^β^Glu, 2H^γ^Glu), 1.45 (s, 9H, Boc), 1.00 (s, 3H, Me), 0.98 (s, 3H, Me), 0.95 (s, 3H, Me), 0.85 (s, 3H, Me), 0.84 (s, 3H, Me), 0.80 (s, 3H, Me), 0.74 (s, 3H, Me). ^13^C NMR (100 MHz, methanol-*d*_4_) *δ* (ppm): 174.4, 80.5, 79.6, 79.4, 79.3, 78.9, 73.6, 56.6, 56.4, 42.7, 41.7, 39.9, 39.8, 38.2, 37.8, 35.9, 35.0, 34.1, 33.7, 33.3, 31.7, 30.7, 30.1, 28.9, 28.6, 28.4, 27.9, 24.3, 19.4, 18.3, 17.3, 16.5, 16.1. HRMS (ESI) *m*/*z* Calcd for C_40_H_65_NO_9_Na 726.4557 (M + H)^+^, found: 726.4546.

3-hydroxy-12β-((S)-4′-amino-4′-carboxybutanoyl)oxy-13H-olean-28-oic acid (**11c**).

To a solution of 50 mg of **10b** in DCM, 50% TFA was added slowly. The mixture was stirred at 23 °C for 4 h. After the material was completely consumed, the mixture was concentrated with vacuum. Then, 1 mL of cold ethyl acetate was added, and cold petroleum ether was added dropwise until white solid precipitated out. The mixture was left to store in refrigerator at 4 °C for 2 h. The precipitate was filtered and rinsed with cold petroleum ether and dried to obtain 16.7 mg of pure white solid with yield of 42.0%. ^1^H NMR (400 MHz, methanol-*d*_4_) *δ* (ppm): 5.02–5.09 (m, 1H, 12-H), 4.70 (dd, 1H, *J* = 4.8, 11.6 Hz, 3-H), 3.97 (t, 1H, *J* = 6.0 Hz, H^α^Glu), 2.56–2.61 (m, 2H, 2H^γ^Glu), 2.51 (dt, 1H, *J* = 4.4, 14.8 Hz, 18-H), 2.13–2.20 (m, 3H, 13-H, 2H^β^Glu), 1.06 (s, 3H, Me), 0.96 (s, 3H, Me), 0.91 (s, 9H, 3Me), 0.89 (s, 3H, Me), 0.84 (s, 3H, Me). ^13^C NMR (100 MHz, methanol-*d*_4_) *δ* (ppm): 181.9, 173.4, 171.7, 87.4, 73.6, 56.2, 53.5, 42.7, 41.8, 41.2, 39.1, 39.0, 38.1, 37.4, 35.3, 34.4, 33.7, 33.4, 32.8, 31.4, 31.2, 29.9, 28.4, 28.2, 27.0, 24.1, 24.0, 23.9, 19.1, 18.2, 16.9, 16.7, 16.3. HRMS (ESI) *m*/*z* Calcd for C_35_H_58_NO_7_ 604.4208 (M + H)^+^, found: 604.4213.

3β-hydroxy-12β-(4′-((methoxycarbonyl)amide)-5′-(tert-butoxy)-L-glutamate)oxy-13H-olean-28-oic acid (**19a**).

White solid 120 mg, overall yield: 47.2%. ^1^H NMR (400 MHz, CDCl_3_) *δ* (ppm): 5.34 (d, 1H, *J* = 8.4 Hz, NH), 4.95–5.03 (m, 1H, 12-H), 4.23–4.29 (m, 1H, H^α^Glu), 3.67 (s, 3H, OMe), 3.20 (dd, 1H, *J* = 4.4, 11.6 Hz, 3-H), 2.49 (dt, 1H, *J* = 4.0, 12.8 Hz, 18-H), 2.31–2.44 (m, 2H, 2H^γ^Glu), 2.14–2.21 (m, 1H, H_a_^β^Glu), 2.08 (dd, 1H, *J* = 4.8, 11.6 Hz, 13-H), 1.94–2.02 (m, 1H, H_b_^β^Glu), 1.47 (s, 9H, Bu^t^), 1.01 (s, 3H, Me), 0.97 (s, 3H, Me), 0.92 (s, 3H, Me), 0.90 (s, 3H, Me), 0.82 (s, 3H, Me), 0.81 (s, 3H, Me), 0.75 (s, 3H, Me), 0.67 (d, 1H, *J* = 10.0 Hz, 5-H). ^13^C NMR (100 MHz, CDCl_3_) *δ* (ppm): 183.6, 172.8 171.2, 156.8, 82.5, 78.9, 72.2, 55.3, 54.1, 52.5, 48.8, 47.3, 41.5, 40.7, 40.0, 39.0, 38.6, 37.2, 36.2, 34.4, 33.4, 33.3, 32.5, 31.4, 30.7, 30.6, 28.9, 28.1, 28.0, 27.4, 27.3, 23.5, 23.0, 18.3, 17.8, 16.6, 16.0, 15.5. HRMS (ESI) *m*/*z* Calcd for C_41_H_68_NO_9_ 718.4889 (M + H)^+^, found: 718.4887.

3β-hydroxy-12β-(4′-((ethoxycarbonyl)amide)-5′-(tert-butoxy)-L-glutamate)oxy-13H-olean-28-oic acid (**19b**).

White solid 157 mg, overall yield: 54.1%. ^1^H NMR (400 MHz, CDCl_3_) *δ* (ppm): 5.28 (d, 1H, *J* = 8.0 Hz, NH), 4.95–5.03 (m, 1H, 12-H), 4.23–4.29 (m, 1H, H^α^Glu), 4.07–4.17 (m, 2H, OCH_2_CH_3_), 3.20 (dd, 1H, *J* = 4.0, 11.6 Hz, 3-H), 2.49 (dt, 1H, *J* = 4.4, 14.0 Hz, 18-H), 2.31–2.44 (m, 2H, 2H^γ^Glu), 2.14–2.21 (m, 1H, H_a_^β^Glu), 2.08 (dd, 1H, *J* = 5.2, 11.6 Hz, 13-H), 1.94–2.00 (m, 1H, H_b_^β^Glu), 1.47 (s, 9H, Bu^t^), 1.24 (t, 3H, *J* = 7.2 Hz, OCH_2_CH_3_), 1.01 (s, 3H, Me), 0.97 (s, 3H, Me), 0.92 (s, 3H, Me), 0.90 (s, 3H, Me), 0.82 (s, 3H, Me), 0.81 (s, 3H, Me), 0.75 (s, 3H, Me), 0.67 (d, 1H, *J* = 10.0 Hz, 5-H). ^13^C NMR (100 MHz, CDCl_3_) *δ* (ppm): 183.5, 172.7 171.3, 156.4, 82.4, 78.9, 72.2, 61.2, 55.3, 54.0, 52.5, 48.9, 47.3, 41.5, 40.7, 40.0, 39.0, 38.6, 37.2, 36.2, 34.4, 33.4, 33.3, 32.5, 31.4, 30.7, 30.6, 29.0, 28.1, 27.4, 27.3, 23.5, 23.0, 18.3, 17.8, 16.6, 16.0, 15.5, 14.7. HRMS (ESI) *m*/*z* Calcd for C_42_H_70_NO_9_ 732.5045 (M + H)^+^, found: 732.5047.

3β-hydroxy-12β-(4′-((propoxycarbonyl)amide)-5′-(tert-butoxy)-L-glutamate)oxy-13H-olean-28-oic acid (**19c**).

White solid 120 mg, overall yield: 47.5%. ^1^H NMR (400 MHz, CDCl_3_) *δ* (ppm): 5.27 (d, 1H, *J* = 8.0 Hz, NH), 4.95–5.02 (m, 1H, 12-H), 4.23–4.28 (m, 1H, H^α^Glu), 3.97–4.07 (m, 2H, OCH_2_CH_2_CH_3_), 3.20 (dd, 1H, *J* = 4.4, 12.0 Hz, 3-H), 2.49 (dt, 1H, *J* = 4.4, 14.0 Hz, 18-H), 2.31–2.44 (m, 2H, 2H^γ^Glu), 2.14–2.21 (m, 1H, H_a_^β^Glu), 2.08 (dd, 1H, *J* = 5.2, 12.0 Hz, 13-H), 1.94–2.01 (m, 1H, H_b_^β^Glu), 1.60–1.65 (m, 2H, OCH_2_CH_2_CH_3_), 1.47 (s, 9H, Bu^t^), 1.01 (s, 3H, Me), 0.97 (s, 3H, Me), 0.93 (t, 3H, *J* = 7.6 Hz, OCH_2_CH_2_CH_3_), 0.92 (s, 3H, Me), 0.90 (s, 3H, Me), 0.82 (s, 3H, Me), 0.81 (s, 3H, Me), 0.75 (s, 3H, Me), 0.67 (d, 1H, *J* = 10.0 Hz, 5-H). ^13^C NMR (100 MHz, CDCl_3_) *δ* (ppm): 183.0, 155.6, 82.3, 78.8, 72.1, 66.8, 55.2, 53.9, 48.8, 47.2, 41.4, 40.6, 39.9, 38.9, 38.6, 37.1, 36.1, 34.3, 33.3, 33.2, 32.4, 31.4, 30.6, 30.5, 28.8, 28.0, 27.3, 27.2, 23.4, 22.9, 22.3, 18.2, 17.8, 16.5, 15.9, 15.4, 10.4. HRMS (ESI) *m*/*z* Calcd for C_43_H_71_NO_9_Na 768.5021 (M + Na)^+^, found: 768.5014.

3β-hydroxy-12β-(4′-((isopropoxycarbonyl)amide)-5′-(tert-butoxy)-L-glutamate)oxy-13H-olean-28-oic acid (**19d**).

White solid 153 mg, overall yield: 57.1%. ^1^H NMR (400 MHz, CDCl_3_) *δ* (ppm): 5.22 (d, 1H, *J* = 8.0 Hz, NH), 4.95–5.02 (m, 1H, 12-H), 4.87–4.92 (m, 1H, OCH(CH_3_)_2_), 4.24 (dt, 1H, *J* = 6.0, 8.0 Hz, H^α^Glu), 3.20 (dd, 1H, *J* = 4.8, 11.6 Hz, 3-H), 2.49 (dt, 1H, *J* = 4.0, 14.0 Hz, 18-H), 2.31–2.44 (m, 2H, 2H^γ^Glu), 2.12–2.20 (m, 1H, H_a_^β^Glu), 2.08 (dd, 1H, *J* = 4.8, 11.6 Hz, 13-H), 1.94–2.01 (m, 1H, H_b_^β^Glu), 1.47 (s, 9H, Bu^t^), 1.23 (d, 6H, *J* = 6.4 Hz, i-Pr-2CH_3_), 1.01 (s, 3H, Me), 0.97 (s, 3H, Me), 0.92 (s, 3H, Me), 0.90 (s, 3H, Me), 0.82 (s, 3H, Me), 0.81 (s, 3H, Me), 0.75 (s, 3H, Me), 0.67 (d, 1H, *J* = 10.0 Hz, 5-H). ^13^C NMR (100 MHz, CDCl_3_) *δ* (ppm): 183.5, 172.6, 171.3, 155.9, 82.3, 78.8, 72.1, 68.5, 55.2, 53.8, 48.8, 47.2, 41.4, 40.6, 39.9, 38.9, 38.6, 37.1, 36.1, 34.3, 33.3, 33.2, 32.4, 31.3, 30.6, 30.5, 29.7, 28.8, 28.0, 27.3, 27.2, 23.4, 22.9, 22.2, 22.1, 18.2, 17.7, 16.5, 15.9, 15.4. HRMS (ESI) *m*/*z* Calcd for C_43_H_72_NO_9_ 746.5201 (M + H)^+^, found: 746.5199.

3β-hydroxy-12β-(4′-((isobutoxycarbonyl)amide)-5′-(tert-butoxy)-L-glutamate)oxy-13H-olean-28-oic acid (**19e**).

White solid 139 mg, overall yield: 53.0%. ^1^H NMR (400 MHz, CDCl_3_) *δ* (ppm): 5.29 (d, 1H, *J* = 8.0 Hz, NH), 4.95–5.02 (m, 1H, 12-H), 4.24 (dt, 1H, *J* = 5.6, 8.0 Hz, H^α^Glu), 3.78–3.92 (m, 2H, OCH_2_CH(CH_3_)_2_), 3.20 (dd, 1H, *J* = 4.4, 11.6 Hz, 3-H), 2.49 (dt, 1H, *J* = 4.4, 14.0 Hz, 18-H), 2.31–2.44 (m, 2H, 2H^γ^Glu), 2.12–2.20 (m, 1H, H_a_^β^Glu), 2.08 (dd, 1H, *J* = 4.8, 11.6 Hz, 13-H), 1.94–2.01 (m, 1H, H_b_^β^Glu), 1.47 (s, 9H, Bu^t^), 1.01 (s, 3H, Me), 0.97 (s, 3H, Me), 0.93 (d, 6H, *J* = 6.5 Hz, OCH_2_CH(CH_3_)_2_), 0.92 (s, 3H, Me), 0.90 (s, 3H, Me), 0.82 (s, 3H, Me), 0.81 (s, 3H, Me), 0.75 (s, 3H, Me), 0.67 (d, 1H, *J* = 10.0 Hz, 5-H). ^13^C NMR (100 MHz, CDCl_3_) *δ* (ppm): 183.5, 172.6, 171.2, 156.4, 82.3, 78.8, 72.1, 71.3, 55.2, 53.8, 48.8, 47.2, 41.4, 40.6, 39.9, 38.9, 38.6, 37.1, 36.1, 34.3, 33.3, 33.2, 32.4, 31.3, 30.6, 30.5, 29.7, 28.8, 28.0, 27.3, 27.2, 23.4, 22.9, 19.1, 18.2, 17.7, 16.5, 15.9, 15.4. HRMS (ESI) *m*/*z* Calcd for C_44_H_74_NO_9_ 760.5358 (M + H)^+^, found: 760.5357.

3β-hydroxy-12β-(4′-((sec-butoxycarbonyl)amide)-5′-(tert-butoxy)-L-glutamate)oxy-13H-olean-28-oic acid (**19f**).

White solid 103 mg, overall yield: 40.9%. ^1^H NMR (400 MHz, CDCl_3_) *δ* (ppm): 5.25 (d, 1H, *J* = 8.4 Hz, NH), 4.95–5.02 (m, 1H, 12-H), 4.69–4.74 (m, 1H, OCH), 4.24 (dt, 1H, *J* = 6.0, 8.0 Hz, H^α^Glu), 3.20 (dd, 1H, *J* = 4.4, 11.6 Hz, 3-H), 2.49 (dt, 1H, *J* = 4.4, 14.0 Hz, 18-H), 2.31–2.44 (m, 2H, 2H^γ^Glu), 2.12–2.20 (m, 1H, H_a_^β^Glu), 2.08 (dd, 1H, *J* = 4.8, 11.2 Hz, 13-H), 1.94–2.01 (m, 1H, H_b_^β^Glu), 1.47 (s, 9H, Bu^t^), 1.20 (d, 3H, *J* = 6.0 Hz, CH_3_), 1.01 (s, 3H, Me), 0.97 (s, 3H, Me), 0.92 (s, 3H, Me), 0.90 (s, 3H, Me), 0.89 (t, 3H, *J* = 4.0 Hz, Me), 0.82 (s, 3H, Me), 0.81 (s, 3H, Me), 0.75 (s, 3H, Me), 0.67 (d, 1H, *J* = 10.0 Hz, 5-H). ^13^C NMR (100 MHz, CDCl_3_) *δ* (ppm): 183.1, 172.6, 171.2, 157.5, 82.3, 78.8, 73.1, 72.1, 55.2, 53.8, 48.8, 47.2, 41.4, 40.6, 39.9, 38.9, 38.6, 37.1, 36.1, 34.3, 33.3, 33.2, 32.4, 31.3, 30.6, 30.5, 29.7, 28.8, 28.0, 27.3, 27.2, 23.4, 22.9, 19.7, 18.2, 17.7, 16.5, 15.8, 15.4. HRMS (ESI) *m*/*z* Calcd for C_44_H_74_NO_9_ 760.5358 (M + H)^+^, found: 760.5361.

3β-hydroxy-12β-(4′-(((cyclopentyloxy)carbonyl)amide)-5′-(tert-butoxy)-L-glutamate)oxy-13H-olean-28-oic acid (**19g**).

White solid 140 mg, overall yield: 54.6%. ^1^H NMR (400 MHz, CDCl_3_) *δ* (ppm): 5.20 (d, 1H, *J* = 8.0 Hz, NH), 5.08 (br, 1H, Cyclopentyl-CH), 4.95–5.02 (m, 1H, 12-H), 4.24 (dt, 1H, *J* = 6.0, 8.0 Hz, H^α^Glu), 3.20 (dd, 1H, *J* = 4.4, 11.2 Hz, 3-H), 2.49 (dt, 1H, *J* = 4.4, 14.0 Hz, 18-H), 2.31–2.44 (m, 2H, 2H^γ^Glu), 2.12–2.20 (m, 1H, H_a_^β^Glu), 2.08 (dd, 1H, *J* = 4.8, 11.4 Hz, 13-H), 1.94–2.01 (m, 1H, H_b_^β^Glu), 1.47 (s, 9H, Bu^t^), 1.01 (s, 3H, Me), 0.97 (s, 3H, Me), 0.92 (s, 3H, Me), 0.89 (s, 3H, Me), 0.81 (s, 3H, Me), 0.75 (s, 3H, Me), 0.67 (d, 1H, *J* = 10.0 Hz, 5-H). ^13^C NMR (100 MHz, CDCl_3_) *δ* (ppm): 183.4, 172.6, 171.3, 156.1, 82.3, 78.8, 77.8, 72.1, 55.2, 53.8, 48.8, 47.2, 41.4, 40.6, 39.9, 38.9, 38.6, 37.1, 36.1, 34.3, 33.3, 33.2, 32.8, 32.7, 32.4, 31.3, 30.6, 30.5, 29.7, 28.8, 28.0, 27.9, 27.3, 27.2, 23.7, 23.6, 23.4, 22.9, 19.7, 18.2, 17.7, 16.5, 15.8, 15.4. HRMS (ESI) *m*/*z* Calcd for C_45_H_74_NO_9_ 772.5358 (M + H)^+^, found: 772.5355.

3β-hydroxy-12β-(4′-((phenoxycarbonyl)amide)-5′-(tert-butoxy)-L-glutamate)oxy-13H-olean-28-oic acid (**19h**).

White solid 142 mg, overall yield: 47.5%. ^1^H NMR (400 MHz, CDCl_3_) *δ* (ppm): 7.34 (dd, 2H, *J* = 8.0, 8.4 Hz, Ph-2H), 7.19 (dd, 1H, *J* = 8.0, 8.4 Hz, Ph-H), 7.12 (d, 1H, *J* = 7.6 Hz, Ph-2H), 5.73 (d, 1H, *J* = 8.4 Hz, NH), 4.97–5.05 (m, 1H, 12-H), 4.32 (dt, 1H, *J* = 5.2, 7.6 Hz, H^α^Glu), 3.17 (dd, 1H, *J* = 4.4, 11.2 Hz, 3-H), 2.49 (dt, 1H, *J* = 4.4, 14.0 Hz, 18-H), 2.41–2.49 (m, 2H, 2H^γ^Glu), 2.18–2.27 (m, 1H, H_a_^β^Glu), 2.08 (dd, 1H, *J* = 4.8, 12.0 Hz, 13-H), 2.01–2.08 (m, 1H, H_b_^β^Glu), 1.49 (s, 9H, Bu^t^), 1.01 (s, 3H, Me), 0.96 (s, 3H, Me), 0.91 (s, 3H, Me), 0.89 (s, 3H, Me), 0.82 (s, 3H, Me), 0.78 (s, 3H, Me), 0.74 (s, 3H, Me), 0.67 (d, 1H, *J* = 10.0 Hz, 5-H). ^13^C NMR (100 MHz, CDCl_3_) *δ* (ppm): 183.4, 172.6, 170.8, 154.3, 129.3, 125.4, 121.6, 82.7, 78.8, 72.2, 55.2, 54.1, 48.8, 47.2, 41.4, 40.6, 39.9, 38.8, 38.5, 37.1, 36.1, 34.3, 33.3, 33.2, 32.4, 31.4, 30.6, 30.5, 28.8, 28.0, 27.8, 27.3, 27.2, 23.4, 22.9, 18.2, 17.7, 16.5, 15.8, 15.4.

3β-hydroxy-12β-(4′-((tert-butoxycarbonyl)amide)-5′-methoxy-L-glutamate)oxy-13H-olean-28-oic acid (**20a**).

White solid 97 mg, overall yield: 33.2%. ^1^H NMR (400 MHz, CDCl_3_) *δ* (ppm): 5.20 (d, 1H, *J* = 8.0 Hz, NH), 4.95–5.02 (m, 1H, 12-H), 4.30–4.33 (m, 1H, H^α^Glu), 3.75 (s, 3H, OMe), 3.20 (dd, 1H, *J* = 4.0, 11.6 Hz, 3-H), 2.49 (dt, 1H, *J* = 3.6, 13.6 Hz, 18-H), 2.40 (t, 2H, *J* = 8.0 Hz, 2H^γ^Glu), 2.14–2.21 (m, 1H, H_a_^β^Glu), 2.08 (dd, 1H, *J* = 5.2, 11.6 Hz, 13-H), 1.94–2.00 (m, 1H, H_b_^β^Glu), 1.44 (s, 9H, Bu^t^), 1.01 (s, 3H, Me), 0.97 (s, 3H, Me), 0.92 (s, 3H, Me), 0.90 (s, 3H, Me), 0.82 (s, 3H, Me), 0.81 (s, 3H, Me), 0.75 (s, 3H, Me), 0.67 (d, 1H, *J* = 10.0 Hz, 5-H). ^13^C NMR (100 MHz, CDCl_3_) *δ* (ppm): 183.8, 172.8 172.5, 155.4, 80.0, 78.8, 72.2, 55.2, 53.0, 52.4, 48.7, 47.2, 41.5, 41.4, 40.6, 39.9, 38.8, 38.5, 37.0, 36.0, 34.3, 33.2, 33.1, 32.4, 31.3, 30.4, 28.8, 28.3, 28.0, 27.6, 27.3, 27.1, 23.3, 22.8, 18.2, 17.7, 16.6, 15.8, 15.3. HRMS (ESI) *m*/*z* Calcd for C_41_H_67_NO_9_Na 740.4708 (M + Na)^+^, found: 740.4706.

3β-hydroxy-12β-(4′-((tert-butoxycarbonyl)amide)-5′-ethoxy-L-glutamate)oxy-13H-olean-28-oic acid (**20b**).

White solid 159 mg, overall yield: 49.5%. ^1^H NMR (400 MHz, CDCl_3_) *δ* (ppm): 5.17 (d, 1H, *J* = 7.6 Hz, NH), 4.96–5.02 (m, 1H, 12-H), 4.30 (dt, 1H, *J* = 6.0, 6.0 Hz, H^α^Glu), 4.20 (q, 2H, *J* = 7.2 Hz, OCH_2_CH_3_), 3.20 (dd, 1H, *J* = 4.4, 11.2 Hz, 3-H), 2.49 (dt, 1H, *J* = 4.4, 14.0 Hz, 18-H), 2.40 (t, 2H, *J* = 7.2 Hz, 2H^γ^Glu), 2.14–2.20 (m, 1H, H_a_^β^Glu), 2.08 (dd, 1H, *J* = 5.2, 11.2 Hz, 13-H), 1.95–2.00 (m, 1H, H_b_^β^Glu), 1.44 (s, 9H, Bu^t^), 1.28 (t, 3H, *J* = 6.8 Hz, OCH_2_CH_3_), 1.01 (s, 3H, Me), 0.97 (s, 3H, Me), 0.92 (s, 3H, Me), 0.89 (s, 3H, Me), 0.82 (s, 3H, Me), 0.81 (s, 3H, Me), 0.75 (s, 3H, Me), 0.67 (d, 1H, *J* = 10.0 Hz, 5-H). ^13^C NMR (100 MHz, CDCl_3_) *δ* (ppm): 183.7, 172.5 172.3, 155.4, 79.9, 78.8, 72.1, 61.5, 55.2, 53.0, 48.7, 47.2, 41.5, 41.4, 40.6, 39.9, 38.8, 38.5, 37.0, 36.0, 34.3, 33.2, 33.1, 32.4, 31.3, 30.4, 28.8, 28.3, 28.0, 27.7, 27.3, 27.1, 23.3, 22.8, 18.2, 17.7, 16.5, 15.3, 14.2. HRMS (ESI) *m*/*z* Calcd for C_42_H_69_NO_9_Na 754.4865 (M + Na)^+^, found: 754.4858.

3β-hydroxy-12β-(4′-((tert-butoxycarbonyl)amide)-5′-propoxy-L-glutamate)oxy-13H-olean-28-oic acid (**20c**).

White solid 150 mg, overall yield: 89.8%. ^1^H NMR (400 MHz, CDCl_3_) *δ* (ppm): 5.16 (d, 1H, *J* = 8.0 Hz, NH), 4.96–5.02 (m, 1H, 12-H), 4.30 (dt, 1H, *J* = 6.0, 6.0 Hz, H^α^Glu), 4.10 (t, 2H, *J* = 6.4 Hz, OCH_2_CH_2_CH_3_), 3.20 (dd, 1H, *J* = 4.4, 11.2 Hz, 3-H), 2.49 (dt, 1H, *J* = 4.4, 14.0 Hz, 18-H), 2.40 (t, 2H, *J* = 7.2 Hz, 2H^γ^Glu), 2.14–2.20 (m, 1H, H_a_^β^Glu), 2.08 (dd, 1H, *J* = 5.2, 11.2 Hz, 13-H), 1.95–2.00 (m, 1H, H_b_^β^Glu), 1.44 (s, 9H, Bu^t^), 1.01 (s, 3H, Me), 0.97 (s, 3H, Me), 0.95 (t, 3H, *J* = 7.6 Hz, OCH_2_CH_2_CH_3_), 0.92 (s, 3H, Me), 0.89 (s, 3H, Me), 0.82 (s, 3H, Me), 0.81 (s, 3H, Me), 0.75 (s, 3H, Me), 0.67 (d, 1H, *J* = 10.0 Hz, 5-H). ^13^C NMR (100 MHz, CDCl_3_) *δ* (ppm): 183.6, 172.5 172.4, 155.4, 79.9, 78.8, 72.1, 67.0, 55.2, 53.0, 48.7, 47.2, 41.4, 40.6, 39.9, 38.8, 38.5, 37.0, 36.0, 34.3, 33.2, 33.1, 32.4, 31.3, 30.5, 30.4, 28.8, 28.3, 28.0, 27.7, 27.3, 27.1, 23.3, 22.8, 21.9, 18.2, 17.7, 16.5, 15.3, 10.4. HRMS (ESI) *m*/*z* Calcd for C_43_H_71_NO_9_Na 768.5021 (M + Na)^+^, found: 768.5012.

3β-hydroxy-12β-(4′-((tert-butoxycarbonyl)amide)-5′-isopropoxy-L-glutamate)oxy-13H-olean-28-oic acid (**20d**).

White solid 125 mg, overall yield: 74.8%. ^1^H NMR (400 MHz, CDCl_3_) *δ* (ppm): 5.15 (d, 1H, *J* = 8.0 Hz, NH), 5.04 (tt, 1H, *J* = 6.0, 6.0 Hz, OCHMe_2_), 4.96–5.00 (m, 1H, 12-H), 4.25 (dt, 1H, *J* = 6.4, 6.8 Hz, H^α^Glu), 3.20 (dd, 1H, *J* = 4.4, 11.2 Hz, 3-H), 2.49 (dt, 1H, *J* = 4.4, 14.0 Hz, 18-H), 2.37–2.42 (m, 2H, 2H^γ^Glu), 2.13–2.27 (m, 1H, H_a_^β^Glu), 2.08 (dd, 1H, *J* = 5.2, 11.2 Hz, 13-H), 1.95–2.00 (m, 1H, H_b_^β^Glu), 1.44 (s, 9H, Bu^t^), 1.27 (d, 3H, *J* = 6.0 Hz, OCHMe_a_), 1.25 (d, 3H, *J* = 6.0 Hz, OCHMe_b_), 1.01 (s, 3H, Me), 0.97 (s, 3H, Me), 0.92 (s, 3H, Me), 0.89 (s, 3H, Me), 0.82 (s, 3H, Me), 0.81 (s, 3H, Me), 0.75 (s, 3H, Me), 0.67 (d, 1H, *J* = 10.0 Hz, 5-H). ^13^C NMR (100 MHz, CDCl_3_) *δ* (ppm): 183.7, 172.5 171.8, 155.4, 79.9, 78.8, 72.1, 69.2, 55.2, 53.2, 48.7, 47.2, 41.4, 40.6, 39.9, 38.8, 38.5, 37.0, 36.0, 34.3, 33.2, 33.1, 32.4, 31.3, 30.5, 30.4, 28.8, 28.3, 28.0, 27.8, 27.3, 27.1, 23.3, 22.8, 21.8, 21.7, 18.2, 17.7, 16.5, 15.8, 15.3. HRMS (ESI) *m*/*z* Calcd for C_43_H_71_NO_9_Na 768.5021 (M + Na)^+^, found: 768.5012.

3β-hydroxy-12β-(4′-((tert-butoxycarbonyl)amide)-5′-isobutoxy-L-glutamate)oxy-13H-olean-28-oic acid (**20e**).

White solid 133 mg, overall yield: 43.4%. ^1^H NMR (400 MHz, CDCl_3_) *δ* (ppm): 5.13 (d, 1H, *J* = 8.0 Hz, NH), 4.96–5.02 (m, 1H, 12-H), 4.32 (dt, 1H, *J* = 6.0, 6.4 Hz, H^α^Glu), 3.92 (d, 2H, *J* = 6.8 Hz, OCH_2_CHMe_2_), 3.20 (dd, 1H, *J* = 4.4, 11.2 Hz, 3-H), 2.49 (dt, 1H, *J* = 4.4, 14.0 Hz, 18-H), 2.40 (t, 2H, *J* = 7.6 Hz, 2H^γ^Glu), 2.14–2.20 (m, 1H, H_a_^β^Glu), 2.09 (dd, 1H, *J* = 5.2, 11.2 Hz, 13-H), 1.94–2.02 (m, 2H, H_b_^β^Glu, OCH_2_CHMe_2_), 1.44 (s, 9H, Bu^t^), 1.01 (s, 3H, Me), 0.97 (s, 3H, Me), 0.95 (d, 6H, *J* = 6.8 Hz, OCH_2_CHMe_2_), 0.92 (s, 3H, Me), 0.89 (s, 3H, Me), 0.82 (s, 3H, Me), 0.81 (s, 3H, Me), 0.75 (s, 3H, Me), 0.67 (d, 1H, *J* = 10.0 Hz, 5-H). ^13^C NMR (100 MHz, CDCl_3_) *δ* (ppm): 182.6, 172.5 172.3, 155.4, 79.9, 78.8, 72.1, 71.5, 55.2, 53.1, 48.7, 47.2, 41.4, 40.6, 39.9, 38.8, 38.5, 37.0, 36.0, 34.3, 33.2, 33.1, 32.4, 31.3, 30.5, 30.4, 28.8, 28.3, 28.0, 27.8, 27.7, 27.3, 27.2, 23.3, 22.9, 19.0, 18.2, 17.7, 16.5, 15.8, 15.3. HRMS (ESI) *m*/*z* Calcd for C_44_H_74_NO_9_ 760.5358 (M + H)^+^, found: 760.5354.

3β-hydroxy-12β-(4′-((tert-butoxycarbonyl)amide)-5′-(sec-butoxy)-L-glutamate)oxy-13H-olean-28-oic acid (**20f**).

White solid 137 mg, overall yield: 44.8%. ^1^H NMR (400 MHz, CDCl_3_) *δ* (ppm): 5.13 (d, 1H, *J* = 8.4 Hz, NH), 4.96–5.02 (m, 1H, 12-H), 4.88 (ddq, 1H, *J* = 2.8, 6.0, 13.2 Hz, OCH(CH_3_)CH_2_CH_3_), 4.28 (dt, 1H, *J* = 6.0, 6.4 Hz, H^α^Glu), 3.20 (dd, 1H, *J* = 4.4, 11.2 Hz, 3-H), 2.49 (dt, 1H, *J* = 4.4, 14.0 Hz, 18-H), 2.40 (t, 2H, *J* = 7.6 Hz, 2H^γ^Glu), 2.14–2.20 (m, 1H, H_a_^β^Glu), 2.09 (dd, 1H, *J* = 5.2, 11.4 Hz, 13-H), 1.94–2.02 (m, 1H, H_b_^β^Glu), 1.44 (s, 9H, Bu^t^), 1.23 (d, 3H, *J* = 6.0 Hz, OCH(CH_3_)CH_2_CH_3_), 1.01 (s, 3H, Me), 0.97 (s, 3H, Me), 0.92 (s, 3H, Me), 0.90 (t, 3H, *J* = 6.4 Hz, OCH(CH_3_)CH_2_CH_3_), 0.89 (s, 3H, Me), 0.82 (s, 3H, Me), 0.81 (s, 3H, Me), 0.75 (s, 3H, Me), 0.67 (d, 1H, *J* = 10.0 Hz, 5-H). ^13^C NMR (100 MHz, CDCl_3_) *δ* (ppm): 183.1, 172.5 171.9, 155.4, 79.9, 78.8, 73.8, 73.7, 72.1, 55.2, 53.3, 53.2, 48.7, 47.2, 41.4, 40.6, 39.9, 38.8, 38.5, 37.1, 36.0, 34.3, 33.2, 33.1, 32.4, 31.3, 30.6, 30.4, 28.8, 28.7, 28.3, 28.0, 27.8, 27.3, 27.2, 23.4, 22.9, 19.4, 19.3, 18.2, 17.7, 16.5, 15.8, 15.3, 9.7. HRMS (ESI) *m*/*z* Calcd for C_44_H_74_NO_9_ 760.5358 (M + H)^+^, found: 760.5359.

3β-hydroxy-12β-(4′-((tert-butoxycarbonyl)amide)-5′-cyclopentyloxy-L-glutamate)oxy-13H-olean-28-oic acid (**20g**).

White solid 179 mg, overall yield: 53.2%. ^1^H NMR (400 MHz, CDCl_3_) *δ* (ppm): 5.19–5.23 (m, 1H, cyclopentyl-CH), 5.12 (d, 1H, *J* = 8.0 Hz, NH), 4.95–5.02 (m, 1H, 12-H), 4.25 (dt, 1H, *J* = 6.0, 6.4 Hz, H^α^Glu), 3.20 (dd, 1H, *J* = 4.4, 11.2 Hz, 3-H), 2.49 (dt, 1H, *J* = 4.4, 14.0 Hz, 18-H), 2.36–2.41 (m, 2H, 2H^γ^Glu), 2.08–2.20 (m, 1H, H_a_^β^Glu), 2.10 (dd, 1H, *J* = 5.2, 11.2 Hz, 13-H), 1.93–1.98 (m, 1H, H_b_^β^Glu), 1.55–1.68 (m, 8H, cyclopentyl-8H), 1.44 (s, 9H, Bu^t^), 1.23 (d, 3H, *J* = 6.0 Hz, OCH(CH_3_)CH_2_CH_3_), 1.01 (s, 3H, Me), 0.97 (s, 3H, Me), 0.92 (s, 3H, Me), 0.89 (s, 3H, Me), 0.82 (s, 3H, Me), 0.81 (s, 3H, Me), 0.75 (s, 3H, Me), 0.67 (d, 1H, *J* = 10.0 Hz, 5-H). ^13^C NMR (100 MHz, CDCl_3_) *δ* (ppm): 182.8, 172.5 172.0, 155.4, 79.9, 78.8, 78.4, 72.1, 55.2, 53.1, 48.7, 47.2, 41.4, 40.6, 39.9, 38.8, 38.5, 37.1, 36.0, 34.3, 33.2, 33.1, 32.7, 32.6, 32.4, 31.3, 30.6, 30.4, 28.8, 28.7, 28.3, 28.0, 27.8, 27.3, 27.2, 23.7, 23.6, 23.4, 22.9, 18.2, 17.7, 16.5, 15.8, 15.3. HRMS (ESI) *m*/*z* Calcd for C_45_H_74_NO_9_ 772.5358 (M + H)^+^, found: 772.5356.

### 3.2. Modeling

The FXR crystal structure, identified by the PDB code 4WVD, was processed using Discovery Studio (DS) V4.5 (Accelrys, Biovia, San Diego, CA, USA). First, hydrogen atoms were introduced, and protonation was carried out. Next, irrelevant protein fractions, primitive ligand (ivermectin), and water molecules were removed. The CHARMm force field was employed to optimize the crystal structure energetically. Based on the position of the primitive ligand, active pockets were defined with a sphere radius of 16.0 Å. DS was used to import oleanolic acid C-ring modified compounds and minimize their energy with the CHARMm force field. The CDocker protocol, utilizing default parameters, was implemented in DS to screen the compound library, generating 10 poses for each compound. The docking results were evaluated based on “-CDOCKER_INTERACTION energy” to predict binding potency. Docking figures were generated using the PyMOL program (DeLano Scientific LLC: Palo Alto, CA, USA).

For the calculation of Gibbs free energy, Schrödinger Prime MM-GBSA was utilized to compute the complexes formed between the three compounds and FXR. VSGB was selected as the solvation model, OPLS4 was chosen for the force field, and the residues within a 5 Å range around the binding pocket ligand were optimized using hierarchical sampling. The following terms contribute to the estimated free energy of binding (ΔG_bind_) in the program: ΔG_Coulomb_ for coulomb energy, ΔG_Covalent_ for covalent binding energy, ΔG_vdW_ for van der Waals energy, ΔG_Lipo_ for lipophilic energy, ΔG_Solv_GB_ for generalized born electrostatic solvation energy, ΔG_Hbond_ for hydrogen bonding correction, ΔG_Packing_ for π-π packing correction, and ΔG_SelfCont_ for self-contact correction.

### 3.3. Biological Evaluation

#### 3.3.1. Regents

The following antibodies and reagents were purchased for use in the study: anti-α-smooth muscle actin antibody (α-SMA; A5228) from Sigma-Aldrich (St Louis, MO, USA); anti-MMP2 (#40994), anti-GAPDH (#5174), and anti-Tubulin (#2148) antibodies and normal rabbit IgG (#2729) from Cell Signaling Technology (Danvers, MA, USA); anti-COL1A1 (PAB17205), anti-Fibronectin (ab2413), anti-Timp1 (ab211926), and anti-TGFβ1 (ab179695) antibodies from Abnova (Colorado, USA) and Abcam; and TGFβ1 (240-B) from R&D Systems (Minneapolis, MN, USA). In addition, ABI Taqman primers/probes were purchased from Applied Biosystems (Foster City, CA, USA).

#### 3.3.2. Cell Culture

Cells were purchased from the ATCC cell bank and cultured in DMEM medium (Hyclone, #SH30243.01, Logan, Utah, USA) supplemented with 10% fetal calf serum (BioInd, #04–001-1ACS, Kibbutz Beit Haemek, Israel) and 1% 100 U·mL^−1^penicillin-100 mg·mL^−1^ streptomycin (Gibco, #15140–122, Carlsbad, California, USA) at 37 °C in a 5% CO_2_ environment. Cells were verified to be free of mycoplasma contamination.

#### 3.3.3. Yeast One-Hybrid Assay

Cell counting: HEK-293T cells in logarithmic growth phase were digested with 0.25% trypsin, collected, and centrifuged. The trypsin-containing medium was discarded, and 2 mL of fresh phenol red-free DMEM medium containing 5% fetal bovine serum was added. The cells were then mixed with the medium to prepare a cell suspension and counted on a cell counting plate.

Cell plating: A determined number of cells were taken from the cell suspension, diluted with phenol red-free DMEM medium containing 5% fetal bovine serum, and plated at a concentration of 10,000 cells per well (100 µL medium) in a 96-well plate. The plate was then incubated at 37 °C and 5% CO_2_ for 24 h.

Cell transfection: Fresh phenol red-free DMEM medium was taken in a sterile environment, and the previously extracted pFXR-Binding plasmid and PG4.35[luc2P-9XGA4UAS-Hygro] plasmid were added. The mixture was stirred and allowed to stand at 23 °C for 3 min. Then, a 3% amount of transfection reagent was added to the mixture, and it was allowed to stand at 23 °C for 15 min. The mixture was then added to the 96-well plate at a volume of 10 µL per well and incubated at 37 °C in a 5% CO_2_ environment for 24 h.

Cell dosing: CDCA was dissolved in a certain amount of DMSO to prepare a 100 mM stock solution, which was further diluted and added to the 96-well plate at a volume of 10 µL per well (50 µM CDCA). The OA derivatives were prepared as 10 mM DMSO stock solutions and further diluted in a concentration gradient, with 10 µL added to each well. The final concentrations of the derivatives ranged from 10 µM to 0.03 µM, with 7 concentration gradients using 2-fold dilution. The test was set with 3 duplicated wells.

Antagonism activity test: The firefly-Renilla Dual Fluorescence Assay Kit (Promega, #E2940, Madison, WI, USA) was used to measure the antagonism activity of the OA derivatives. Firstly, the firefly fluorescence reagent was added to the 96-well plate at a volume of 70 µL per well and shaken in darkness for 15 min. Then, the fluorescence intensity was measured at a wavelength of 645 ± 30 nm in a microplate reader (BioTek, Winooski, VT, USA). Next, the volume of Renilla reagent was added to the 96-well plate at a volume of 70 µL per well and shaken in darkness for 15 min. The fluorescence intensity was then measured at a wavelength of 525 ± 20 nm.

Data processing: The fluorescence data of firefly were divided by the fluorescence data of Renilla to remove errors, and the antagonism efficiency was calculated using the following formula: antagonism efficiency (%) = [1 − (F_Compds._ − F_DMSO_)/(F_CDCA_ − F_DMSO_)] × 100%. In the formula, F represents fluorescence intensity. The antagonistic efficiency data were obtained as a concentration–effect curve using Graphpad Prism 8.0 software, and the corresponding IC_50_ value was calculated.

#### 3.3.4. NR Selectivity

To assess the compound’s specificity, the mammalian one-hybrid assay was conducted. In this assay, HEK-293T cells were co-transfected with a fusion plasmid of Gal4 DBD-NR LBD (comprising RXRα, RXRβ, RXRγ, PPARα, PPARβ, PPARγ, LXRα, LXRβ, GPBAR, and PXR) and the UAS-TK-Luc reporter, followed by treatment with various concentrations of the compound. The positive control consisted of the corresponding nuclear receptor agonist, and the concentration used in the experiment was set to the EC_50_ of the agonist.

#### 3.3.5. Quantitative Real-Time PCR

Cells and liver tissue samples were subjected to RNA extraction and purification, followed by reverse transcription of equal amounts of total RNA into cRNAs using a Transcriptor First Strand cDNA Synthesis Kit (Roche). The relative expression levels of the target gene were determined using TaqMan probe/primers with an ABI Q3 quantitative real-time PCR system, while *GAPDH*/*Gapdh* was utilized as an internal control for mRNA expression. The fold change in the expression of the target mRNA was calculated using Equation 2^−ΔΔCt^.

#### 3.3.6. Immunoprecipitation and Western Blot

The total protein concentration in cell and liver tissue lysates was determined using a BCA kit (Beyotime Biotechnology, P0009). Following immune precipitation with appropriate antibodies overnight at 4 °C and incubation with Protein A/G Plus-agarose (Santa Cruz Biotechnology, sc-2003), immunocomplexes or lysates were subjected to SDS-PAGE gel electrophoresis and transferred onto PVDF membranes. Target proteins were detected using specific primary antibodies and an appropriate secondary antibody, with electrochemiluminescence carried out according to the manufacturer’s instructions with a Tanon 5200 imaging system (Shanghai, China). The internal control utilized was GADPH.

#### 3.3.7. Animal Experiments

All animal experiments were conducted in accordance with the guidelines and approved by the local Institutional Animal Care and Use Committee (Beijing, China). Male Sprague Dawley rats (6 weeks old, weighing 160–180 g, and specific pathogen-free class) and C57BL/6N mice (8 weeks old, weighing 18–22 g, and specific pathogen-free class) were obtained from Vital River Laboratory Animal Technology Co., Ltd. (Beijing, China). The laboratory animals were housed in a pathogen-free environment with controlled temperature (20–24 °C), relative humidity (40–60%), and a 12 h light/dark cycle. Random group assignments were made for the animals, and all animal samples were analyzed in a blinded manner.

#### 3.3.8. Bile Duct Ligation (BDL) Surgery in Rats

The study involved 24 rats that were randomly assigned to 1 of 3 groups, including sham, BDL-N.S., and BDL-**10b**-100mg·kg^−1^, ensuring 8 animals per group using Research Randomizer (RRID: SCR_008563). Of the 24 rats, 16 underwent BDL surgery, wherein the choledochal duct was ligated with surgical sutures after administering isoflurane anesthesia. After 24 h, the BDL rats were randomly divided into three groups and received daily gavages of either normal saline (BDL-NS group) or 100 mg·kg^−1^ of compound **10b** suspended in 0.5% sodium carboxymethylcellulose solution (BDL-**10b** groups) for 14 days. A healthy control group was established with eight rats undergoing sham surgery. Bodyweights were measured daily, and blood samples were collected from the abdominal aorta after an overnight fast. Euthanasia was performed using isoflurane, following which bile was collected using a syringe, and liver tissue samples were collected. Some of the samples were fixed with 4% paraformaldehyde for subsequent histological examination, while others were stored at −80 °C for further analysis.

#### 3.3.9. CDHFD-Induced Mice Model

A total of 24 C57BL/6N mice were randomly assigned to three groups, including Ctrl., Model-N.S., and Model-**10b**-100 mg·kg^−1^, with eight animals per group. The randomization process was performed using Research Randomizer (RRID: SCR_008563). CDHFD (D09100310) diet was obtained from Research Diets Inc. Sixteen mice were fed the CDHFD diet for 12 weeks to induce NASH, with eight mice receiving a normal diet as a control. At the beginning of the 13th week, the mice in the model group were randomly assigned into two groups and given daily gavages of either normal saline (Model-N.S. group) or 100 mg·kg^−1^ of compound **10b** suspended in 0.5% sodium carboxymethylcellulose solution (Model-**10b** groups) for four weeks. Daily bodyweights were recorded, and blood samples were collected from the abdominal aorta following an overnight fast. Afterward, the mice were euthanized using isoflurane. Liver tissue samples were obtained, and some were treated with 4% paraformaldehyde to prepare them for future histological analysis, while others were kept at −80 °C for later examination. Maximum liver lobule was collected for each mouse.

#### 3.3.10. Serum Biochemical Parameters Detection

Kits purchased from Zhongsheng Beikong Biotechnology (Beijing, China) were used to measure the serum concentrations of alanine aminotransferase, aspartate aminotransferase, alkaline phosphatase, γ-glutamyl transpeptidase, total cholesterol, total triglyceride, low-density lipoprotein (LDL), high-density lipoprotein (HDL) cholesterol, lactate dehydrogenase, total bile acid, and total bilirubin. The samples were analyzed on a Hitachi 7100 Analyzer.

#### 3.3.11. Examination of Liver Tissue Samples Using Histological Methods

The liver tissue was fixed with formalin, embedded in paraffin, and sectioned. The sections were stained with H&E, Oil Red, Masson’s, and Sirius Red. Liver necrosis, inflammation, bile duct proliferation, hepatic steatosis, and lobule inflammation were evaluated on a scale of 1–5 in a blinded manner using a Leica DM1000 microscope. Five randomly selected manifold area fields of vision were used to quantify the red area in each field of vision (eight in each group), which was counted using ImageJ software. The liver hydroxyproline concentration was determined using kits from Nanjing, China, in accordance with the manufacturer’s instructions.

#### 3.3.12. TC, TG, and Free Cholesterol Detection of Liver Sample

The mouse liver samples were cut into small pieces of 50 mg size and homogenized in TG, TC, and FC extracts using a high-speed tissue homogenizer. The TG, TC, and FC were then extracted from the tissue, and their contents in the sample were calculated using the protein content for calibration. The determination process utilized the test kit for TC, TG, and FC of high-fat samples from Applygen Technologies Inc. (E1025, E1026, E1027).

#### 3.3.13. Data Analysis

All data are presented as mean ± standard deviation (SD). For BDL rat experiments, eight rats were randomly assigned to each group. For CDHFD-diet-induced NASH model, eight C57BL/6N mice were randomly assigned to each group. For in vitro experiments, three independent replicates were performed. The control group was used as a reference value of “1”, and the test values were expressed as fold changes relative to the control value.

Statistical analysis was performed for three independent experiments. When the F-test achieved a *p*-value less than 0.05 and there was no significant violation of homogeneity of variance, a one-way ANOVA with Tukey’s post hoc test was employed. Statistical significance was determined at a level of *p* < 0.05 or *p* < 0.01.

## 4. Conclusions

In this research, a variety of 12β-*O*-β-aspartyl and 12β-*O*-γ-glutamyl derivatives of OA were created and synthesized. We also examined the SAR with respect to several structural factors, including chirality, carbon chain length, distance between α-amino and OA nucleus, and the size of 12β-aspartyl/glutamyl substituents. As a result, a potent FXR antagonistic compound, **10b**, was selected for serial in vitro and in vivo assays. It upregulated CYP7A1, a gene important to bile acid metabolism. This upregulation is distinct from that observed with another previously explored OA derivative, **7**. It also showed inhibitory effects on liver fibrosis at molecular and cellular levels. The in vivo tests demonstrated that compound **10b** effectively inhibits lipid accumulation in the liver and prevents liver fibrosis in both BDL rats and HFD mice. The binding model indicated that the *tert*-butyl groups of **10b** extended to the H11–H12 region of FXR-LBD, which may potentially account for its selective gene regulation.

In conclusion, the findings of our study indicate that compound **10b** may have potential as a lead compound in the search for further FXR modulators. In addition, minor structural modification in the FXR ligand (e.g., 12-OA) can be exploited to change gene regulation pattern so as to modify phenotypes in different metabolic diseases.

## Figures and Tables

**Table 1:** The antagonistic activity of 12β-OA derivatives.

Compounds	IC_50_ (μM) ^a^	Compounds	IC_50_ (μM)
**9a**	0.95 ± 0.14	**9b**	2.39 ± 0.35
**10a**	1.95 ± 0.27	**10b**	0.44 ± 0.15
**10c**	1.34 ± 0.23	**10d**	3.67 ± 0.39
**11a**	2.95 ± 0.68	**11b**	7.65 ± 0.35
**11c**	21.3 ± 2.6	**19a**	0.55 ± 0.22
**19b**	0.57 ± 0.36	**19c**	0.45 ± 0.13
**19d**	0.47 ± 0.16	**19e**	0.92 ± 0.14
**19f**	0.60 ± 0.04	**19g**	0.72 ± 0.18
**19h**	2.18 ± 0.56	**20a**	1.10 ± 0.19
**20b**	1.02 ± 0.21	**20c**	0.56 ± 0.13
**20d**	0.55 ± 0.09	**20e**	1.00 ± 0.17
**20f**	1.01 ± 0.18	**20g**	0.82 ± 0.11

^a^ The experimental data were obtained using yeast one-hybrid assay, and the IC_50_ values were calculated from the results of three independent tests (mean ± SE). CDCA (50 μM) was used as agonist.

**Table 2:** Binding energies of 12β-substituted OA derivatives with FXR-LBD.

Compds.	ΔG_bind_ (Kcal·mol^−1^)	ΔG_Coulomb_ (Kcal·mol^−1^)	ΔG_Covalent_ (Kcal·mol^−1^)	ΔG_Hbond_ (Kcal·mol^−1^)	ΔG_Lipo_ (Kcal·mol^−1^)	ΔG_Packing_ (Kcal·mol^−1^)	ΔG_SelfCont_ (Kcal·mol^−1^)	ΔG_Solv_GB_ (Kcal·mol^−1^)	ΔG_vdW_ (Kcal·mol^−1^)
**7**	−100.65	−8.17	3.60	−1.35	−41.09	0.04	0.01	18.03	−71.70
**10b**	−127.83	−0.84	12.10	−1.11	−61.34	0.05	−0.01	11.06	−87.73
**21**	−108.01	−4.21	3.19	−0.40	−49.91	0.26	−0.14	4.02	−60.81

## Data Availability

Data is contained within the article.

## Supplementary Materials

The following supporting information can be downloaded at https://www.mdpi.com/article/10.3390/ph16050758/s1. Scheme S1: Synthesis route for key intermediate **8**; Scheme S2: Preparation of the corresponding γ-glutamic acids; Figure S1: The relationship curve between the absorption peak area and mass of compound **10b** at 220 nm; Table S1: The contents of compound **10b** extracted from mouse plasma in different time periods; Table S2: Serum biochemical markers of BDL rats (m = 8); Table S3: Serum biochemical markers of HFD mice (n = 7); Figure S2: (A) Binding mode of compound **10b** (green) as ligand interacting with FXR-LBD; (B) Binding model of compound **7** (pink) and **21** (orange) as ligands interacting with FXR-LBD; Figures S3–S76: NMR of intermediates (**14a–h** and **18a–h**) and compounds (**8**, **9a**,**b**, **10a**–**d**, **11a**–**c**, **19a**–**h**, and **20a**–**g**); Figures S77–S92: MS of intermediates **14a**–**h** and **18a**–**h**; Figures S93–S115: HRMS of compound **8**, **9a**,**b**, **10a**–**d**, **11a**–**c**, **19c**–**g**, and **20a**–**g**.
