# Supplementary material for: Structure Optimization of 12β-O-γ-Glutamyl Oleanolic Acid Derivatives Resulting in Potent FXR Antagonist/Modulator for NASH Therapy
Source: Pharmaceuticals (Basel). 2023 May 17;16(5):758. doi: 10.3390/ph16050758 (PMC10221314; doi:10.3390/ph16050758)
Supplement: Supplementary file 1 [file pharmaceuticals-16-00758-s001.zip › pharmaceuticals-2362817-supplementary.pdf]

Supporting Information

Structure Optimization of 12β-*O*-γ-Glutamyl Oleanolic Acid Derivatives Resulting In FXR Antagonist/Modulator for NASH therapy

Hao Ma [1,†], Yunyang Bao [2,†], Shuaishuai Niu [1], Shaorong Wang [1], Yiming Li [2], Hongwei He [2], Na Zhang [2], Weishuo Fang [1,*]

[1] *State Key Laboratory of Bioactive Substances and Functions of Natural Medicines & Ministry of Health Key Laboratory of Biosynthesis of Natural Products, Institute of Materia Medica, Chinese Academy of Medical Sciences & Peking Union Medical College, 2A Nan Wei Road, Beijing 100050, China*

[2] *Key Laboratory of Biotechnology of Antibiotics, the National Health and Family Planning Commission (NHFPC), Institute of Medicinal Biotechnology, Chinese Academy of Medical Sciences and Peking Union Medical College, Beijing 100050, China*

* Correspondence: wfang@imm.ac.cn

†: These authors contributed equally to this work.

Contents

Contents	Page
1. Preparation of key intermediate **8**	2
2. Preparation of γ-glutamic acids **14a~h** and **18a~h** bearing different size of protecting groups	3
3. Stability test of compound **10b**	8
4. Compound **10b** affects markers of liver injury and hepatic fibrosis in BDL rats	9
5. Compound **10b** affects serum biochemical markers in HFD mice	10
6. Binding model of 12β-OA derivative interacting with FXR-LBD	11
7. NMR and HRMS spectra of all compounds	12

1. Preparation of key intermediate **8**

Starting with oleanolic acid (OA, **6**), the C-28 carboxyl group was protected with benzyl (Bn) to create benzyl 3β-hydroxy-olean-28-oate (Scheme S1), which improved the solubility of the substrates and facilitated product purification in subsequent reactions. The t-butylmethylsilyl (TBS) was chosen to protect 3-OH group. This yielded the 3-*O*-TBS derivative in high yield (81%) upon treatment with t-butyldimethylsilyl trifluoromethanesulfonate (TBSOTf) and imidazole. We prepared the 12-oxo derivative through m-CPBA oxidation of the olefin, followed by rearrangement of an epoxide intermediate. The newly formed chiral center (C-13) was assigned as β based on stereochemistry described previously, which was supported by an NOESY experiment. The C-12 carbonyl group was then reduced using $NaBH_4$ in tetrahydrofuran (THF) in the presence of trace water to produce the 12β- and 12α-hydroxyl derivatives in a ratio of approximately 2:1. The 12β-hydroxy OA (**8**) was finally separated through chromatography on silica gel (Pe/EtOAc: 20:1-10:1).

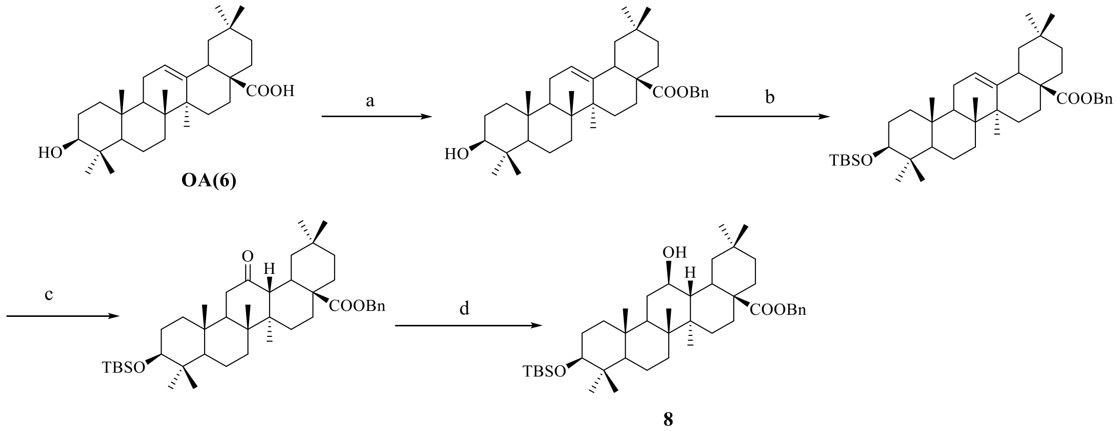

Scheme S1. Synthesis route for key intermediate **8**. *Reagents and conditions*: (a) BnBr, K_2CO_3, tetrabutylammonium bromide (TBAB), THF, H_2O; (b) TBSOTf, imidazole, CH_2Cl_2; (c) *m*-CPBA, CH_2Cl_2; (d) $NaBH_4$, THF.

2. Preparation of γ-glutamic acids bearing different size of protecting groups.

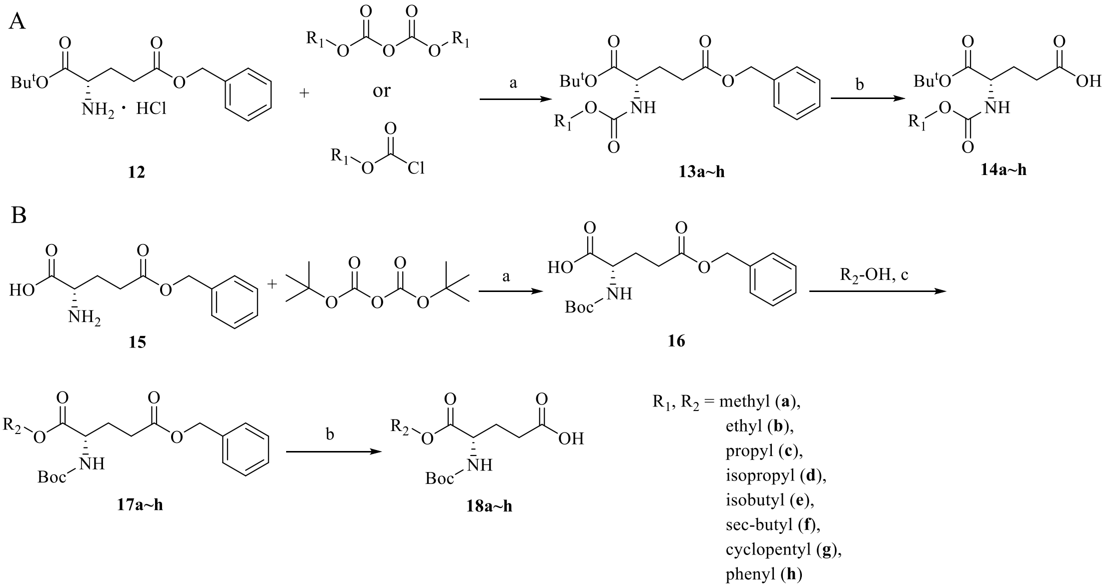

Scheme S2. Preparation of the corresponding γ-glutamic acids. Reagents and conditions: (a) DIPEA, THF/H_2O 4:1, 23°C for 8 h, yield: > 98% (for anhydride), or DIPEA, DCM, 23°C for 4 h, yield: >98% (for acyl chloride); (b) Pd/C, hydrogen (1 atm.), MeOH, 23°C for 6 h, yield: 55.7~97.1%; (c)DIC, 4-DMAP, DCM, 23°C for 8 h, yield: >98%.

As shown in Scheme S2, For α-carboxyl terminal protected by *tert*-butyl ester, amino-terminal introduced different substituents of glutamic acid modification, the following method was prepared as follows: to a solution of *L*-glutamic acid-5-benzyl ester-1-*tert*-butyl ester hydrochloride (1 g, 3.0 mmol, 1.0 eq.) in 15 mL of THF/H_2O 4:1 mixture, DIPEA 1.3 mL (9.0 mmol, 3.0 eq.) and acid anhydrides containing different substituents (2.0 eq.) were slowly added. The reaction mixture was stirred at room temperature for 12 hours (if it is acid chlorides substituted by different substituents, the reaction solvent should be adjusted to dry DCM, and the reagent equivalent should be the same as above). Then the mixture was diluted with water and extracted with ethyl acetate. The organic layers were combined and dried over sodium sulfate and concentrated in vacuum to obtain crude product using to the next step without any purification. The obtained intermediates were dissolved in 15 mL of methanol, with 10% of the mass of palladium carbon. The hydrogen gas at 1 atmosphere was passed through, and the reaction mixture was stirred at room temperature. After 6 hours, the mixture was diluted with water and extracted with ethyl acetate. The organic layers were combined and dried over sodium sulfate and concentrated in vacuum to obtain crude products, and then directly subjected to flash chromatography on silica gel using PE/EtOAc (2:1) to obtain intermediates quantitatively.

*5-(tert-butoxy)-4-((methoxycarbonyl)amino)-L-glutamic acid (**14a**).*

White solid 441 mg, overall yield: 55.7%. $[a]_D^{20}$ = +14.4° (c 1.3, $CHCl_3$). ^{1}H NMR (400 MHz, $CDCl_3$) *d* (ppm): 10.66 (br, 1H, COOH), 5.40 (d, 1H, *J* = 6.8 Hz, NH), 4.26-4.32 (m, 1H, 4-H), 3.68 (s, 3H, OC$\underline{H_3}$), 2.37-2.53 (m, 2H, 2-2H), 2.17-2.20 (m, 1H, 3-H_a), 1.90-1.99 (m, 1H, 3-H_b), 1.47 (s, 9H, OBu^t). ^{13}C NMR (100 MHz, $CDCl_3$) δ(ppm): 178.0, 171.1, 156.8, 82.7, 53.7, 52.4, 30.0, 28.0, 27.8. MS (ESI) *m/z* 284.2 $(M+Na)^+$.

*5-(tert-butoxy)-4-((ethoxycarbonyl)amino)-L-glutamic acid (**14b**).*

White solid 792 mg, overall yield: 94.8%. $[a]_D^{20}$ = +11.4° (c 1.2, $CHCl_3$). ^{1}H NMR (400 MHz, $CDCl_3$) *d* (ppm): 10.56 (br, 1H, COOH), 5.34 (d, 1H, *J* = 7.6 Hz, NH), 4.26-4.32 (m, 1H, 4-H), 4.12 (q, 2H, *J* = 6.8 Hz, OC$\underline{H_2}CH_3$), 2.37-2.53 (m, 2H, 2-2H), 2.15-2.23 (m, 1H, 3-H_a), 1.89-1.99 (m, 1H, 3-H_b), 1.47 (s, 9H, OBu^t), 1.25 (t, 3H, *J* = 7.2 Hz, $OCH_2C\underline{H_3}$). ^{13}C NMR (100 MHz, $CDCl_3$) δ(ppm): 177.9, 171.1, 156.4, 82.6, 61.3, 53.5, 30.0, 28.0, 28.0, 14.5. MS (ESI) *m/z* 298.1 $(M+Na)^+$.

*5-(tert-butoxy)-4-((propoxycarbonyl)amino)-L-glutamic acid (**14c**).*

White solid 758 mg, overall yield: 86.4%. $[a]_D^{20}$ = +10.8° (c 2.0, $CHCl_3$). ^{1}H NMR (400 MHz, $CDCl_3$) *d* (ppm): 10.58 (br, 1H, COOH), 5.34 (d, 1H, *J* = 7.2 Hz, NH), 4.27-4.32 (m, 1H, 4-H), 4.12 (t, 2H, *J* = 6.4 Hz, OC$\underline{H_2}CH_2CH_3$), 2.37-2.53 (m, 2H, 2-2H), 2.17-2.23 (m, 1H, 3-H_a), 1.89-1.99 (m, 1H, 3-H_b), 1.59-1.68 (m, 2H, $OCH_2C\underline{H_2}CH_3$), 1.47 (s, 9H, OBu^t), 0.93 (t, 3H, *J* = 7.2 Hz, $OCH_2CH_2C\underline{H_3}$). ^{13}C NMR (100 MHz, $CDCl_3$) δ (ppm): 177.8, 171.1, 156.5, 82.6, 66.9, 53.5, 30.0, 28.0, 28.0, 22.3, 10.3. MS (ESI) *m/z* 312.2 $(M+Na)^+$.

*5-(tert-butoxy)-4-((isopropoxycarbonyl)amino)-L-glutamic acid (**14d**).*

White solid 836 mg, overall yield: 95.3%. $[a]_D^{20}$ = +9.0° (c 1.2, $CHCl_3$). ^{1}H NMR (400 MHz, $CDCl_3$) *d* (ppm): 9.80 (br, 1H, COOH), 5.26 (d, 1H, *J* = 7.6 Hz, NH), 4.86-4.92 (m, 1H, OC$\underline{H}Me_2$), 4.27-4.30 (m, 1H, 4-H), 2.37-2.52 (m, 2H, 2-2H), 2.16-2.21 (m, 1H, 3-H_a), 1.89-1.98 (m, 1H, 3-H_b), 1.47 (s, 9H, OBu^t), 1.23 (d, 6H, *J* = 6.0 Hz, OCH$\underline{Me_2}$). ^{13}C NMR (100 MHz, $CDCl_3$) δ(ppm): 177.9, 171.1, 156.0, 82.5, 68.7, 53.5, 30.0, 28.0, 28.0, 22.0. MS (ESI) *m/z* 312.3 $(M+Na)^+$.

*5-(tert-butoxy)-4-((isobutoxycarbonyl)amino)-L-glutamic acid (**14e**).*

White solid 828 mg, overall yield: 90.1%. $[a]_D^{20}$ = +8.9° (c 1.3, $CHCl_3$). ^{1}H NMR

(400 MHz, $CDCl_3$) *d* (ppm): 5.36 (br, 1H, NH), 4.26-4.31 (m, 1H, 4-H), 3.84 (d, 2H, *J* = 6.4 Hz, $OC\underline{H_2}CHMe_2$), 2.37-2.52 (m, 2H, 2-2H), 2.15-2.23 (m, 1H, 3-H_a), 1.85-1.96 (m, 2H, 3-H_b, $OCH_2C\underline{H}Me_2$), 1.47 (s, 9H, OBu^t), 0.92 (d, 6H, *J* = 6.4 Hz, $OCH_2CH\underline{Me_2}$). ^{13}C NMR (100 MHz, $CDCl_3$) δ (ppm): 177.7, 171.2, 156.5, 82.6, 71.4, 53.6, 30.0, 28.0, 28.0, 28.0, 19.0. MS (ESI) *m/z* 326.3 $(M+Na)^+$.

*5-(tert-butoxy)-4-((sec-butoxycarbonyl)amino)-L-glutamic acid (**14f**).*

White solid 972 mg, overall yield: 97.1%. $[a]_D^{20}$ = +7.8° (c 1.9, $CHCl_3$). ^1H NMR (400 MHz, $CDCl_3$) *d* (ppm): 10.29 (br, 1H, COOH), 5.26 (d, 1H, *J* = 7.6 Hz, NH), 4.70-4.74 (m, 1H, 1'-H), 4.26-4.31 (m, 1H, 4-H), 2.37-2.52 (m, 2H, 2-2H), 2.15-2.20 (m, 1H, 3-H_a), 1.89-1.98 (m, 1H, 3-H_b), 1.50-1.63 (m, 2H, 2'-2H), 1.47 (s, 9H, OBu^t), 1.20 (d, 3H, *J* = 6.0 Hz, 1'-$C\underline{H_3}$), 0.90 (t, 3H, *J* = 7.6 Hz, 3'-3H). ^{13}C NMR (100 MHz, $CDCl_3$) δ (ppm): 177.7, 171.1, 156.3, 82.5, 73.3, 53.5, 30.0, 29.0, 28.0, 28.0, 19.7, 9.6. MS (ESI) *m/z* 326.3 $(M+Na)^+$.

*5-(tert-butoxy)-4-(((cyclopentyloxy)carbonyl))amino)-L-glutamic acid (**14g**).*

White solid 811 mg, overall yield: 84.8%. $[a]_D^{20}$ = +7.7° (c 1.7, $CHCl_3$). ^1H NMR (400 MHz, $CDCl_3$) *d* (ppm): 10.28 (br, 1H, COOH), 5.24 (d, 1H, *J* = 7.6 Hz, NH), 5.08 (br, 1H, cyclopentyl-H), 4.26-4.31 (m, 1H, 4-H), 2.36-2.52 (m, 2H, 2-2H), 2.15-2.21 (m, 1H, 3-H_a), 1.90-1.97 (m, 1H, 3-H_b), 1.80-1.85 (m, cyclopentyl-2H), 1.68-1.70 (m, 4H, cyclopentyl-4H), 1.53-1.60 (m, 2H, cyclopentyl-2H), 1.47 (s, 9H, OBu^t). MS (ESI) *m/z* 338.3 $(M+Na)^+$. Known compound.

*5-(tert-butoxy)-4-((phenoxycarbonyl)amino)-L-glutamic acid (**14h**).*

White solid 803 mg, overall yield: 81.9%. $[a]_D^{20}$ = +10.1° (c 2.2, $CHCl_3$). ^1H NMR (400 MHz, $CDCl_3$) *d* (ppm): 7.35 (dd, 2H, *J* = 7.6, 8.4 Hz, Ph-2H), 7.20 (dd, 1H, *J* = 7.6, 8.4 Hz, Ph-H), 7.12 (d, 2H, *J* = 7.6 Hz, Ph-2H), 5.78 (d, 1H, *J* = 8.0 Hz, NH), 4.36 (ddd, 1H, *J* = 5.2, 8.0, 8.4 Hz, 4-H), 2.41-2.57 (m, 2H, 2-2H), 2.21-2.29 (m, 1H, 3-H_a), 1.97-2.06 (m, 1H, 3-H_b), 1.49 (s, 9H, OBu^t). ^{13}C NMR (100 MHz, $CDCl_3$) δ (ppm): 177.9, 170.8, 154.4, 150.8, 129.3, 125.5, 121.6, 83.0, 53.8, 29.9, 28.0, 27.7. MS (ESI) *m/z* 346.2 $(M+Na)^+$.

For α-carboxyl terminal modified with esters of different substituents and amino terminal protected by *tert*-butoxycarbonyl, the following method was prepared as follows: to a solution of *L*-glutamic acid-γ-benzyl (1 g, 4.2 mmol, 1.0 eq.) in 15 mL

THF/H_2O 4:1 mixture, DIPEA (1.3 mL, 9.0 mmol, 3.0 eq.) and $(Boc)_2O$ (2.0 eq.) were added slowly, and the mixture was stirred at 23°C for 12 hours. Then the mixture was diluted with water and extracted with ethyl acetate. The organic layers were combined and dried over sodium sulfate and concentrated in vacuum to obtain crude product using to the next step directly. The intermediate in DCM 15 mL was added alcohols with different substituents (equivalent ratio 1:1). Then DIC (1.0 eq.) and 4-DMAP (0.2 eq). were added slowly, and the mixture was stirred at room temperature for 12 hours. Then the mixture was diluted with water and extracted with ethyl acetate. The organic layers were combined and dried over sodium sulfate and concentrated in vacuum to obtain crude products using to the next step without any purification. The corresponding g-glutamic acids were prepared under the same catalytic hydrogenation conditions as previously described, and were subsequently subjected to flash chromatography on silica gel using PE/EtOAc (2:1) as the solvent.

*4-((tert-butoxycarbonyl)amino)-5-methoxy-L-glutamic acid (**18a**).*

White solid 325 mg, overall yield: 98.8%. $[\alpha]_D^{20}$ = +13.9° (c 1.2, $CHCl_3$). ^1H NMR (400 MHz, $CDCl_3$) *d* (ppm): 9.28 (br, 1H, COOH), 5.19 (d, 1H, *J* = 7.6 Hz, NH), 4.34-4.40 (m, 1H, 4-H), 3.75 (s, 3H, $OC\underline{H_3}$), 2.39-2.53 (m, 2H, 2-2H), 2.17-2.22 (m, 1H, 3-H_a), 1.92-1.97 (m, 1H, 3-H_b), 1.44 (s, 9H, Boc). MS (ESI) *m/z* 284.3 $(M+Na)^+$. Known compound.

*4-((tert-butoxycarbonyl)amino)-5-ethoxy-L-glutamic acid (**18b**).*

White solid 314 mg, overall yield: 89.2%. $[\alpha]_D^{20}$ = +12.0° (c 1.4, $CHCl_3$). ^1H NMR (400 MHz, $CDCl_3$) *d* (ppm): 9.42 (br, 1H, COOH), 5.21 (d, 1H, *J* = 7.6 Hz, NH), 4.31-4.36 (m, 1H, 4-H), 4.20 (q, 2H, *J* = 7.2 Hz, $OC\underline{H_2}CH_3$), 2.39-2.53 (m, 2H, 2-2H), 2.16-2.23 (m, 1H, 3-H_a), 1.90-2.01 (m, 1H, 3-H_b), 1.44 (s, 9H, Boc), 1.29 (t, 3H, *J* = 7.2 Hz, $OCH_2C\underline{H_3}$). MS (ESI) *m/z* 298.2 $(M+Na)^+$. Known compound.

*4-((tert-butoxycarbonyl)amino)-5-propoxy-L-glutamic acid (**18c**).*

White solid 286 mg, overall yield: 85.6%. $[\alpha]_D^{20}$ = +10.5° (c 1.2, $CHCl_3$). ^1H NMR (400 MHz, $CDCl_3$) *d* (ppm): 5.20 (d, 1H, *J* = 7.6 Hz, NH), 4.32-4.38 (m, 1H, 4-H), 4.10 (t, 3H, *J* = 6.4 Hz, $OC\underline{H_2}CH_2CH_3$), 2.39-2.54 (m, 2H, 2-2H), 2.16-2.23 (m, 1H, 3-H_a), 1.92-1.99 (m, 1H, 3-H_b), 1.63-1.72 (m, 2H, $OCH_2C\underline{H_2}CH_3$), 1.44 (s, 9H, Boc), 1.29 (t, 3H, *J* = 7.6 Hz, $OCH_2CH_2C\underline{H_3}$). MS (ESI) *m/z* 312.3 $(M+Na)^+$. Known compound.

*4-((tert-butoxycarbonyl)amino)-5-isopropoxy-L-glutamic acid (**18d**).*

White solid 300 mg, overall yield: 75.4%. $[\alpha]_D^{20}$ = +10.1° (c 1.2, $CHCl_3$). ^{1}H NMR (400 MHz, $CDCl_3$) *d* (ppm): 5.20 (d, 1H, *J* = 7.6 Hz, NH), 5.00-5.10 (m, 1H, OCHMe$_2$), 4.27-4.32 (m, 1H, 4-H), 2.38-2.53 (m, 2H, 2-2H), 2.15-2.20 (m, 1H, 3-H$_a$), 1.89-1.98 (m, 1H, 3-H$_b$), 1.44 (s, 9H,Boc), 1.23 (d, 6H, *J* = 6.0 Hz, OCHMe$_2$). MS (ESI) *m/z* 312.2 (M+Na)$^+$. Known compound.

*4-((tert-butoxycarbonyl)amino)-5-isobutoxy-L-glutamic acid (**18e**).*

White solid 320 mg, overall yield: 81.0%. $[\alpha]_D^{20}$ = +8.7° (c 1.8, $CHCl_3$). ^{1}H NMR (400 MHz, $CDCl_3$) *d* (ppm): 11.21 (s, 1H, COOH), 5.20 (d, 1H, *J* = 8.0 Hz, NH), 4.34-4.39 (m, 1H, 4-H), 3.92 (d, 2H, *J* = 6.8 Hz, OCH$_2$CHMe$_2$), 2.39-2.54 (m, 2H, 2-2H), 2.18-2.23 (m, 1H, 3-H$_a$), 1.91-2.01 (m, 2H, 3-H$_b$, OCH$_2$CHMe$_2$), 1.44 (s, 9H, Boc), 0.94 (d, 6H, *J* = 6.8 Hz, OCH$_2$CHMe$_2$). ^{13}C NMR (100 MHz, $CDCl_3$) δ (ppm): 178.0, 172.3, 155.5, 80.2, 71.6, 52.8, 30.1, 28.3, 27.8, 27.7, 19.0. MS (ESI) *m/z* 326.3 (M+Na)$^+$.

*4-((tert-butoxycarbonyl)amino)-5-(sec-butoxy)-L-glutamic acid (**18f**).*

White solid 325 mg, overall yield: 62.4%. $[\alpha]_D^{20}$ = +8.2° (c 1.0, $CHCl_3$). ^{1}H NMR (400 MHz, $CDCl_3$) *d* (ppm): 5.22 (d, 1H, *J* = 7.2 Hz, NH), 4.89 (ddq, 1H, *J* = 3.2, 6.4, 13.2 Hz, 1'-H), 4.29-4.34 (m, 1H, 4-H), 2.37-2.53 (m, 2H, 2-2H), 2.15-2.22 (m, 1H, 3-H$_a$), 1.89-1.98 (m, 1H, 3-H$_b$), 1.52-1.67 (m, 2H, 2'-2H), 1.44 (s, 9H, Boc), 1.23 (d, 3H, *J* = 5.6 Hz, 1'-CH$_3$), 0.90 (t, 3H, *J* = 7.6 Hz, 3'-3H). ^{13}C NMR (100 MHz, $CDCl_3$) δ (ppm): 177.9, 171.9, 155.6, 80.1, 74.0, 53.0, 30.1, 28.7, 28.3, 27.9, 19.4, 9.7. MS (ESI) *m/z* 326.3 (M+Na)$^+$.

*4-((tert-butoxycarbonyl)amino)-5-cyclopentyloxy-L-glutamic acid (**18g**).*

White solid 344 mg, overall yield: 69.6%. $[\alpha]_D^{20}$ = +7.8° (c 1.4, $CHCl_3$). ^{1}H NMR (400 MHz, $CDCl_3$) *d* (ppm): 11.04 (br, 1H, COOH), 5.17-5.23 (m, 2H, NH, cyclopentyl-H), 4.26-4.31 (m, 1H, 4-H), 2.38-2.52 (m, 2H, 2-2H), 2.14-2.20 (m, 1H, 3-H$_a$), 1.84-1.97 (m, 3H, 3-H$_b$, cyclopentyl-2H), 1.68-1.76 (m, 4H, cyclopentyl-4H), 1.59-1.62 (m, 2H, cyclopentyl-2H), 1.44 (s, 9H, Boc). MS (ESI) *m/z* 338.3 (M+Na)$^+$. Known compound.

*4-((tert-butoxycarbonyl)amino)-5-phenoxy-L-glutamic acid (**18h**).*

White solid 155 mg, overall yield: 30.9%. $[\alpha]_D^{20}$ = +10.4° (c 1.8, $CHCl_3$). ^{1}H NMR (400 MHz, $CDCl_3$) *d* (ppm): 7.38 (dd, 2H, *J* = 8.0, 8.4 Hz, Ph-2H), 7.23-7.26 (m, 1H,

Ph-H), 7.10 (d, 2H, J = 8.0 Hz, Ph-2H), 5.22 (d, 1H, J = 8.0 Hz, NH), 4.58-4.61 (m, 1H, 4-H), 2.51-2.65 (m, 2H, 2-2H), 2.36-2.39 (m, 1H, 3-H_a), 2.09-2.15 (m, 1H, 3-H_b), 1.47 (s, 9H, Boc). MS (ESI) *m/z* 346.3 $(M+Na)^+$. Known compound.

3. Stability test of compound **10b**

To preliminarily determine the stability of compound **10b** in a biological environment, an in vitro experimental verification method was employed. The trace compound was dissolved in mouse plasma to simulate the biological environment. Samples were taken at different time intervals at 37°C, and the content of the compound was determined by HPLC C18 analytical column. The peak area of the absorption at 220 nm was utilized for content calculation.

The standard curve shown in Fig. S1 can be plotted using the peak area and the mass of compound **10b** in the corresponding volume of the solution. The relationship is y = 23.253x+ 100.67 (R^2 = 0.9994), which meets linearity requirements and can be used for subsequent operations.

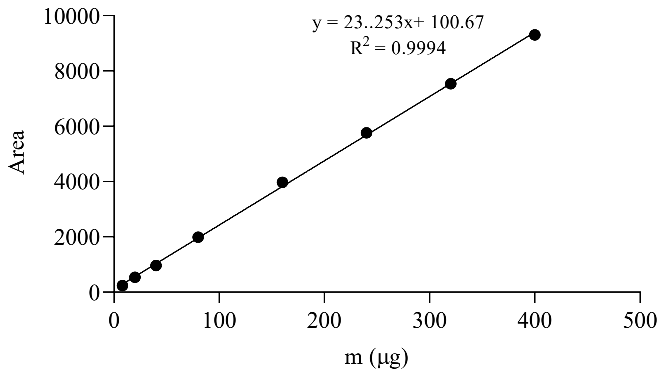

Fig. S1. The relationship curve between the absorption peak area and mass of compound **10b** at 220 nm.

Subsequently, a mouse with a body weight of 20 g and blood volume of 1 mL was calculated based on the subsequent in vivo experiment's dosage (100 mg·Kg^{-1}). Assuming 100% absorption, the converted plasma drug concentration is 2 mg·mL^{-1}. Based on the calculation, 0.5 mg of **10b** was added to 200 mL of mouse plasma to prepare a mixed solution. The plasma was then processed after 1h, 6h, 12 h, 24 h, and 48 h, respectively. The compound content in the sample was compared to the initial content with UV absorption and retention time (t = 6.5 min). As shown in Table S1, no significant decrease in the content of **10b** was observed between 0 to 48 hours, indicating that compound **10b** existed stably in mouse plasma.

Table S1. The contents of compound **10b** extracted from mouse plasma in different time periods.

Time (37ºC)	Peak area	Mass of **10b** (μg)	Theoretical mass of **10b** (μg)	Recovery rate
0 h	710.1	26.2	38.5	68.0 %
1 h	743.3	27.3	38.5	70.9 %
6 h	781.0	29.2	38.5	75.8 %
12 h	667.1	24.4	38.5	63.4 %
24 h	714.8	26.4	38.5	68.5 %
48 h	786.8	29.5	38.5	76.6 %

4. Compound **10b** affects markers of liver injury and hepatic fibrosis in BDL rats

Table S2. Serum biochemical markers of BDL rats (n=8).

	**Sham(n=8)**	**BDL(n=8)**	**10b 100mg·kg^{-1}(n=7)**
Serum ALT (U·L^{-1})	27.75±3.86	102.38±41.49$^{\#\#}$	84.83±18.11
Serum AST (U·L^{-1})	116±20.02	659.75±179.41$^{\#\#}$	435.5±98.62*
Serum ALP (U·L^{-1})	178.5±54.74	371.00±41.17$^{\#\#}$	391.33±52.42
Serum γ-GT (U·L^{-1})	/	39.63±22.93$^{\#\#}$	37.5±6.74
Serum CHO (mmol·L^{-}	1.57±0.16	2.55±0.44$^{\#\#}$	2.08±0.43
Serum LDL (mmol·L^{-}	1.57±0.16	1.08±0.22$^{\#\#}$	1.06±0.22
Serum HDL (mmol·L^{-}	0.65±0.08	0.47±0.12$^{\#\#}$	0.29±0.06**
Serum TG (mmol·L^{-1})	0.27±0.12	0.74±0.24$^{\#\#}$	0.67±0.19
Serum TBA (μmol·L^{-1})	8.53±2.65	228.45±31.99$^{\#\#}$	202.60±30.80
Serum TBiL (μmol·L^{-}	0.78±0.21	177.45±19.94$^{\#\#}$	180.00±26.16
Bile TBA(μmol·L^{-1})	/	258.46±69.07	249.4±103.67
Bile TBiL (μmol·L^{-1})	/	5.63±1.51	5.8±2.46
Urine TBA(μmol·L^{-1})	-0.53±1.62	24.75±20.07$^{\#\#}$	15.38±5.76
Urine TBiL (μmol·L^{-1})	2.93±4.72	6.65±4.09$^{\#\#}$	5.33±1.50

5. Compound **10b** affects serum biochemical markers in HFD mice

Table S3. Serum biochemical markers of HFD mice (n=7)

	Ctrl (n=7)	Model (n=7)	**10b** 100mg·kg^{-1}(n=7)
Serum ALT (U·L^{-1})	32.63±30.61	258.33±43.36$^{\#\#}$	325.43±88.40
Serum AST (U·L^{-1})	114.88±32.21	326.83±43.41$^{\#\#}$	401.86±91.05
Serum ALP (U·L^{-1})	59.00±8.30	128.83±9.50$^{\#\#}$	189.71±29.41
Serum LDH (U·L^{-1})	474.00±188.43	1563.67±245.77$^{\#\#}$	1326.57±264.54
Serum CHO (mmol·L^{-1})	2.79±0.32	2.13±0.42$^{\#\#}$	2.36±0.44
Serum LDL (mmol·L^{-1})	0.14±0.02	0.33±0.13$^{\#\#}$	0.23±0.04
Serum HDL (mmol·L^{-1})	1.89±0.32	1.03±0.20$^{\#\#}$	1.22±0.33
Serum TG (mmol·L^{-1})	1.24±0.25	1.08±0.18	0.85±0.30
Serum Glu (mmol·L^{-1})	8.72±0.94	3.80±1.37$^{\#\#}$	5.71±1.25
Serum TBA (μmol·L^{-1})	1.34±0.57	25.72±4.10$^{\#\#}$	23.46±12.81
Serum TBiL (μmol·L^{-1})	1.48±1.21	3.80±1.13$^{\#\#}$	3.81±0.92

Values are presented as the mean ± SD (n=7); ## $p<0.01$, significantly different from the sham group.

6. Binding model of 12β-OA derivative interacting with FXR-LBD

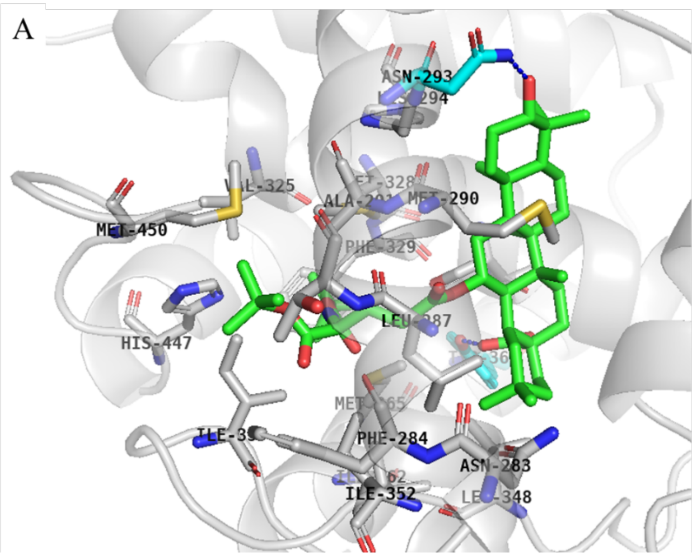

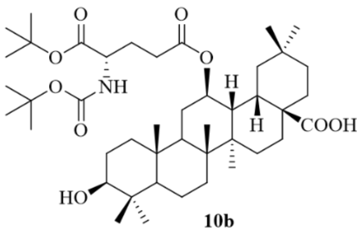

10b

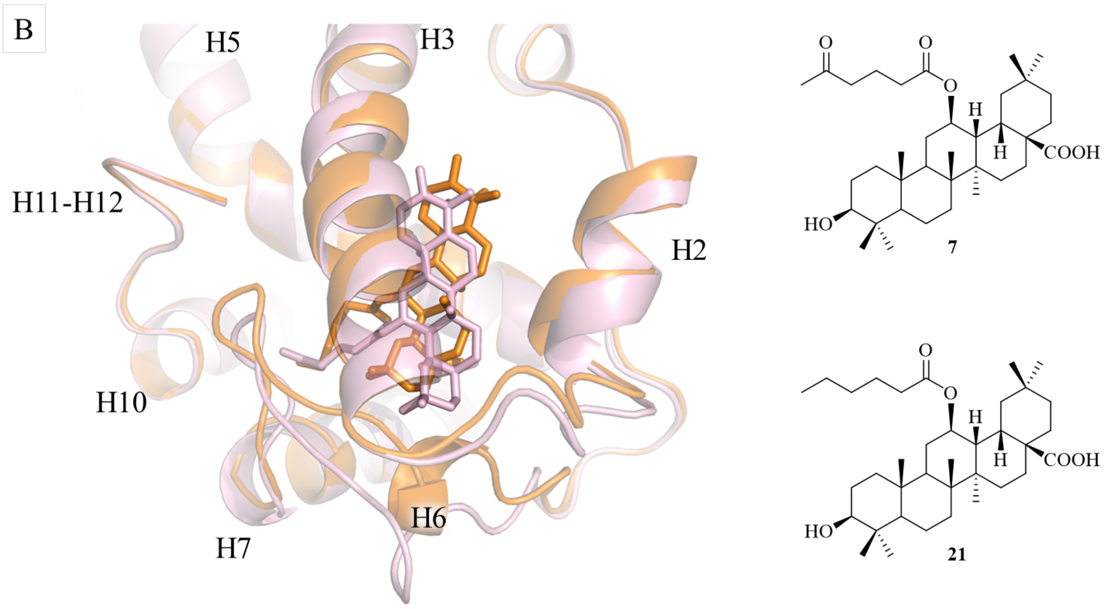

Fig. S2. (A) Binding model of compound **10b** (green) as ligand interacting with FXR-LBD. (B) Binding model of compound **7** (pink) and **21** (orange) as ligands interacting with FXR-LBD.

7. NMR and HRMS spectra of all compounds

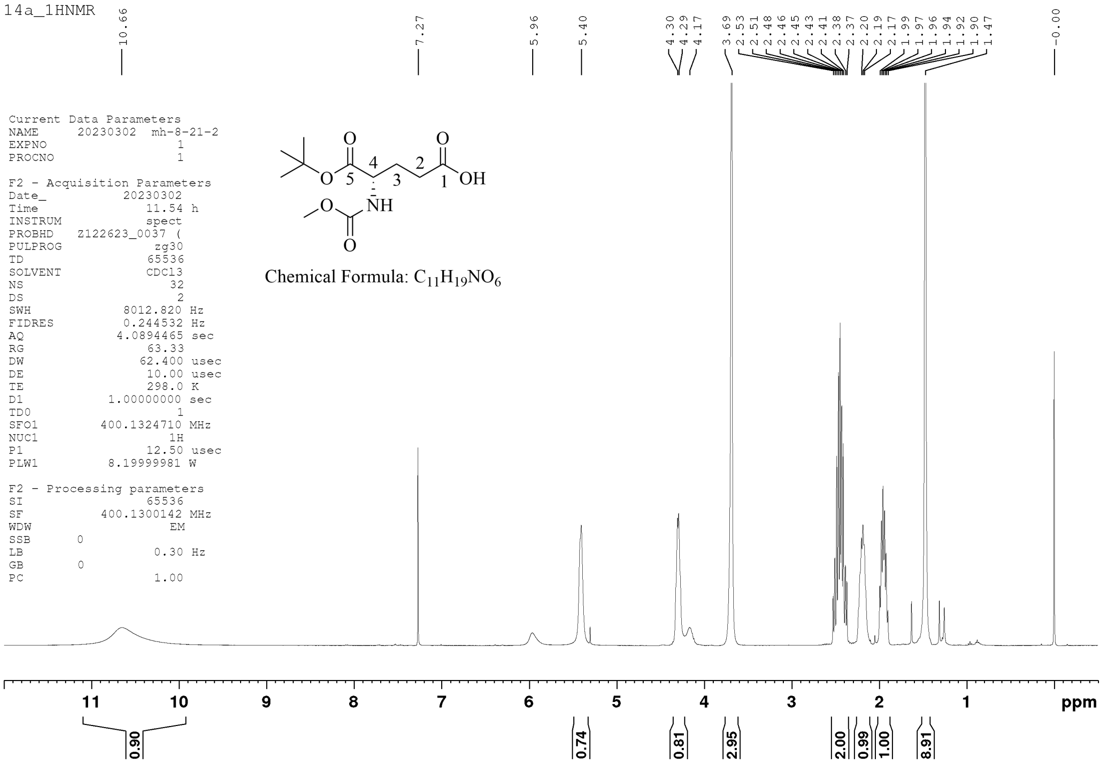

Fig. S3. ^{1}H NMR of intermediate **14a**

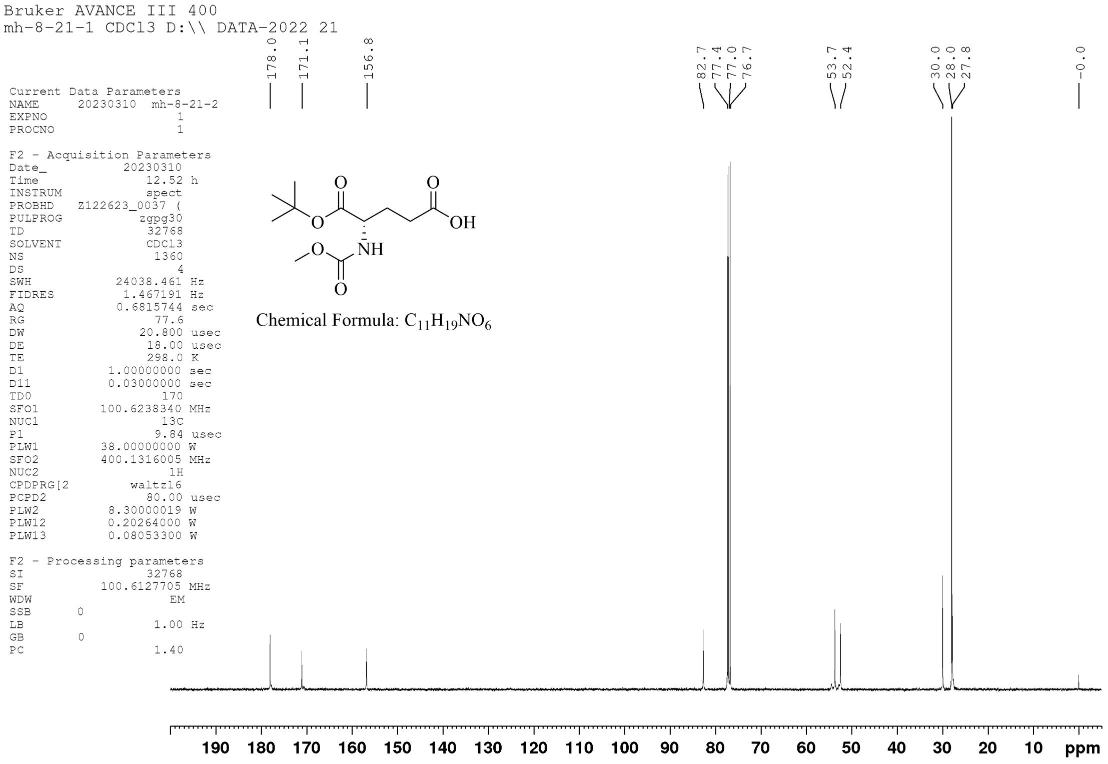

Fig. S4. ^{13}C NMR of intermediate **14a**

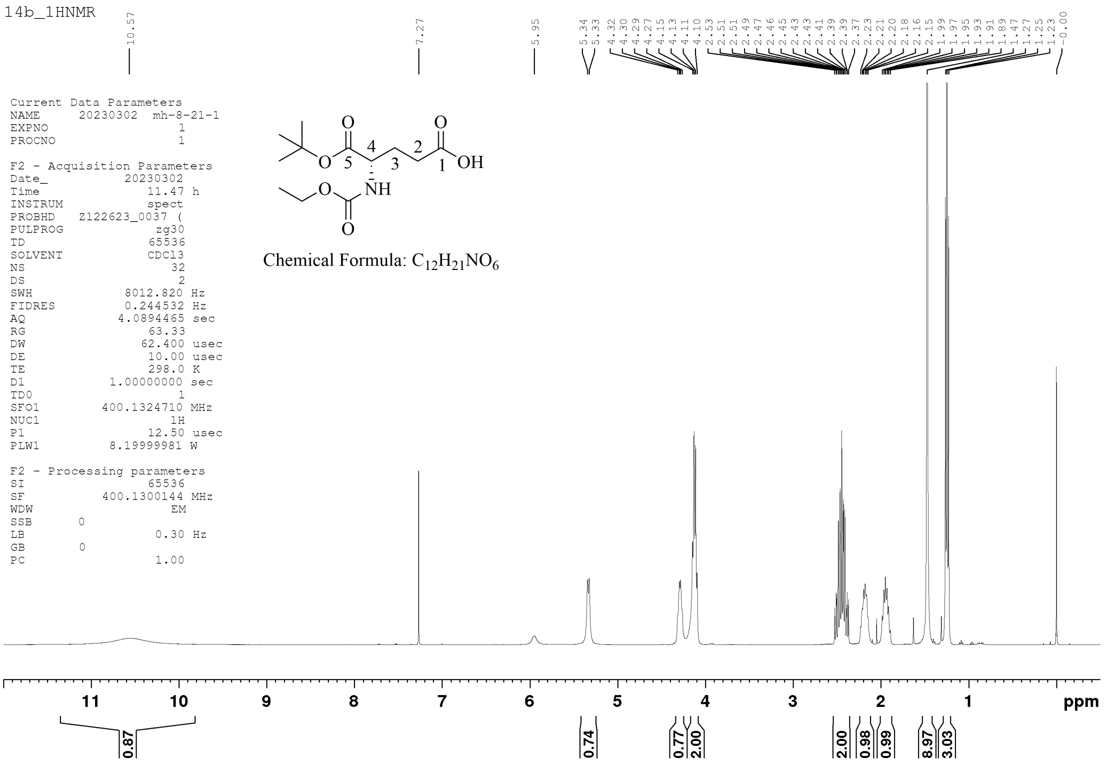

Fig. S5. ^{1}H NMR of intermediate **14b**

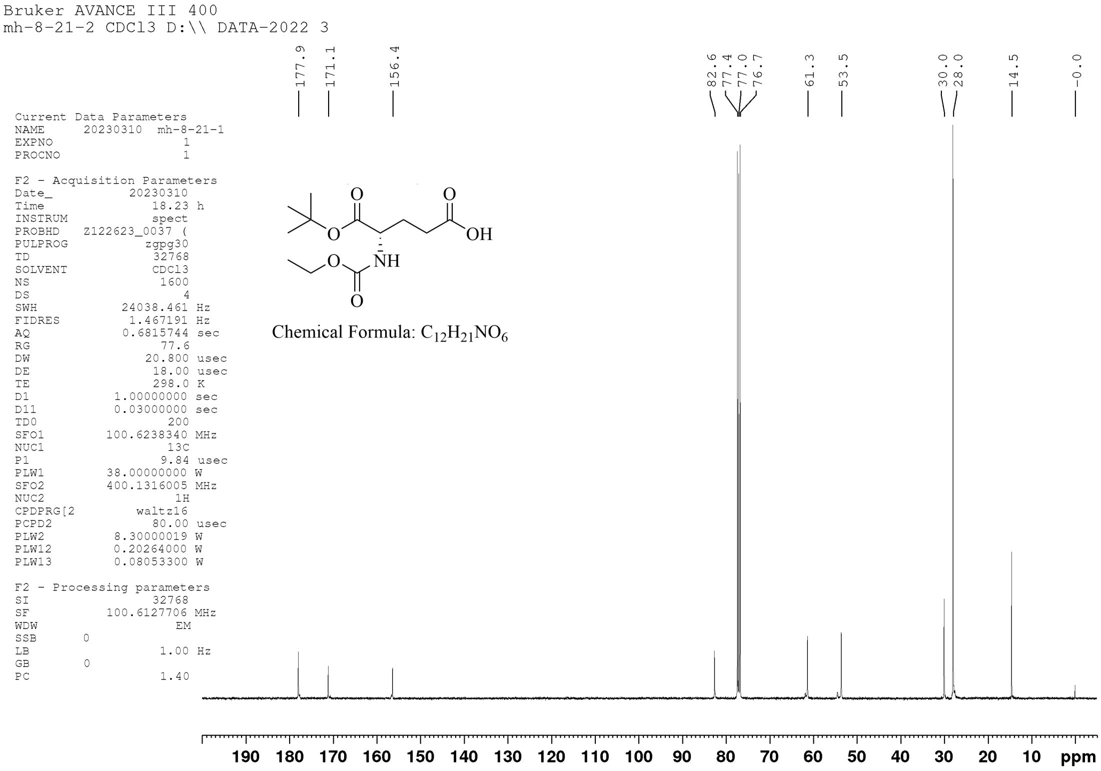

Fig. S6. ^{13}C NMR of intermediate **14b**

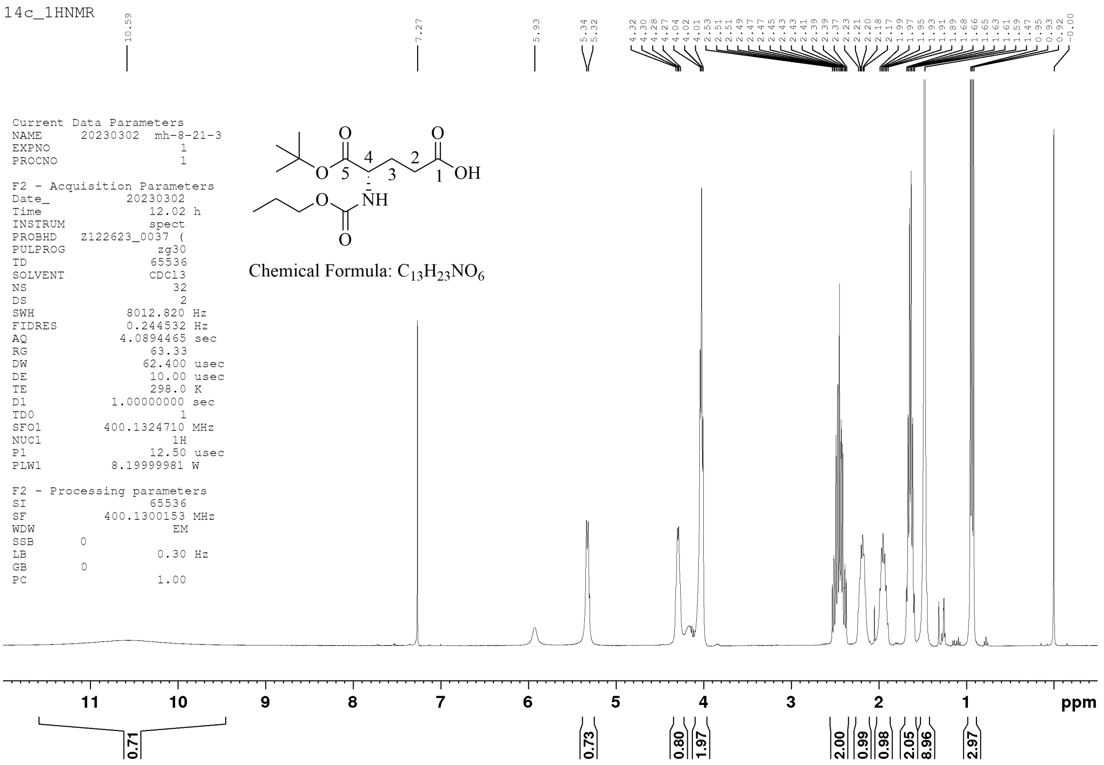

Fig. S7. ^{1}H NMR of intermediate **14c**

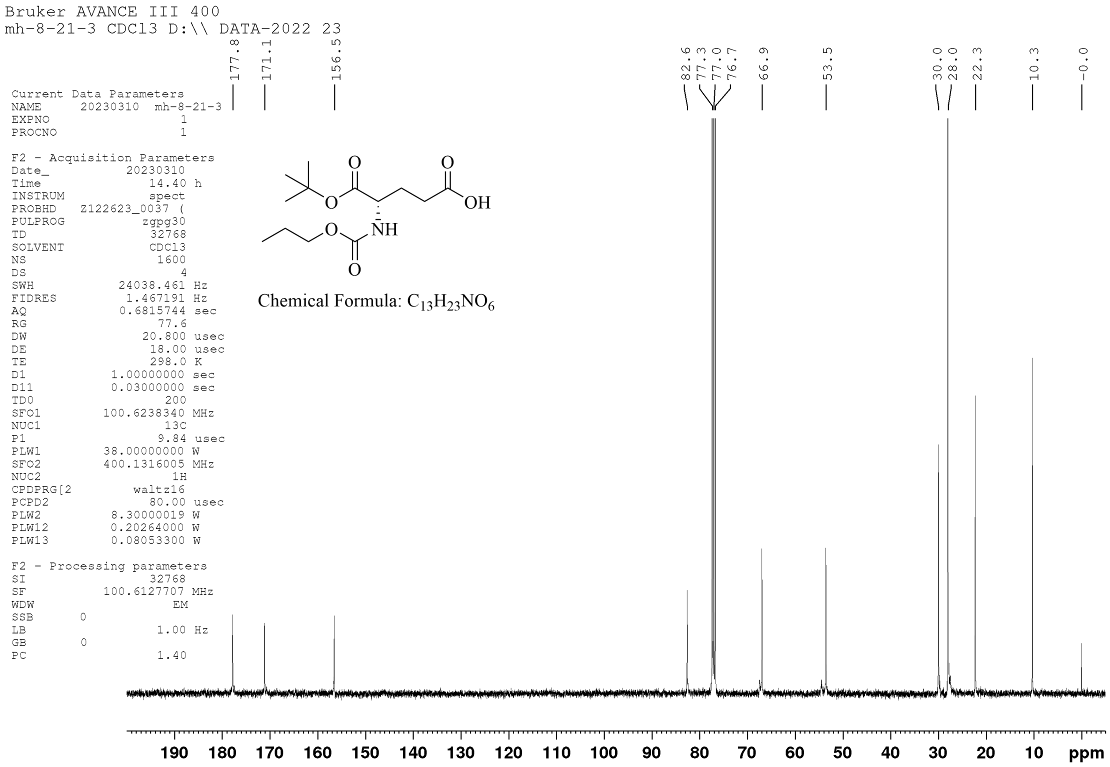

Fig. S8. ^{13}C NMR of intermediate **14c**

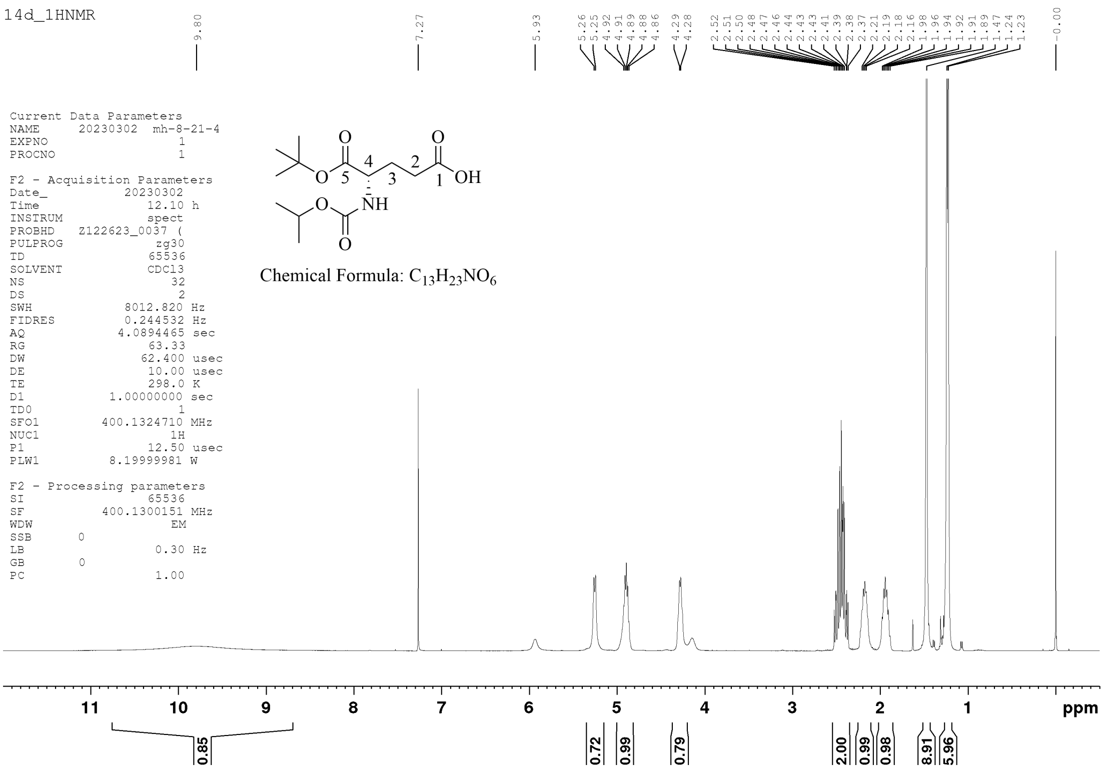

Fig. S9. ^{1}H NMR of intermediate **14d**

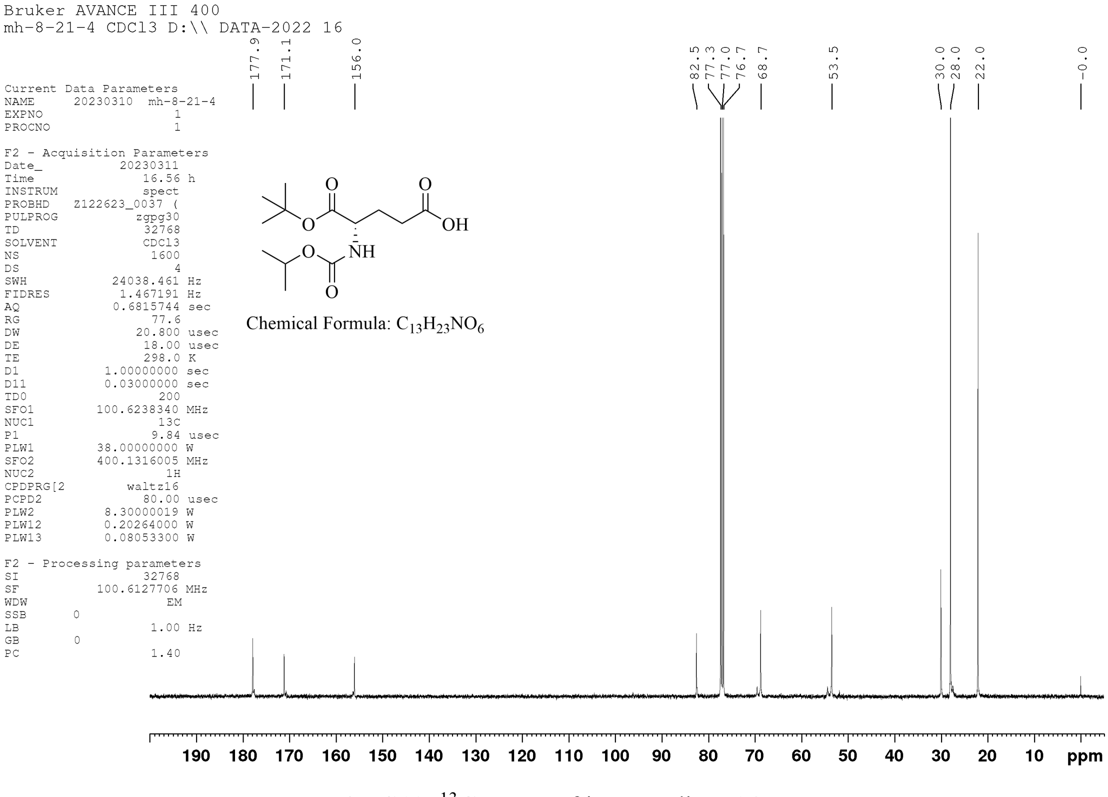

Fig. S10. ^{13}C NMR of intermediate **14d**

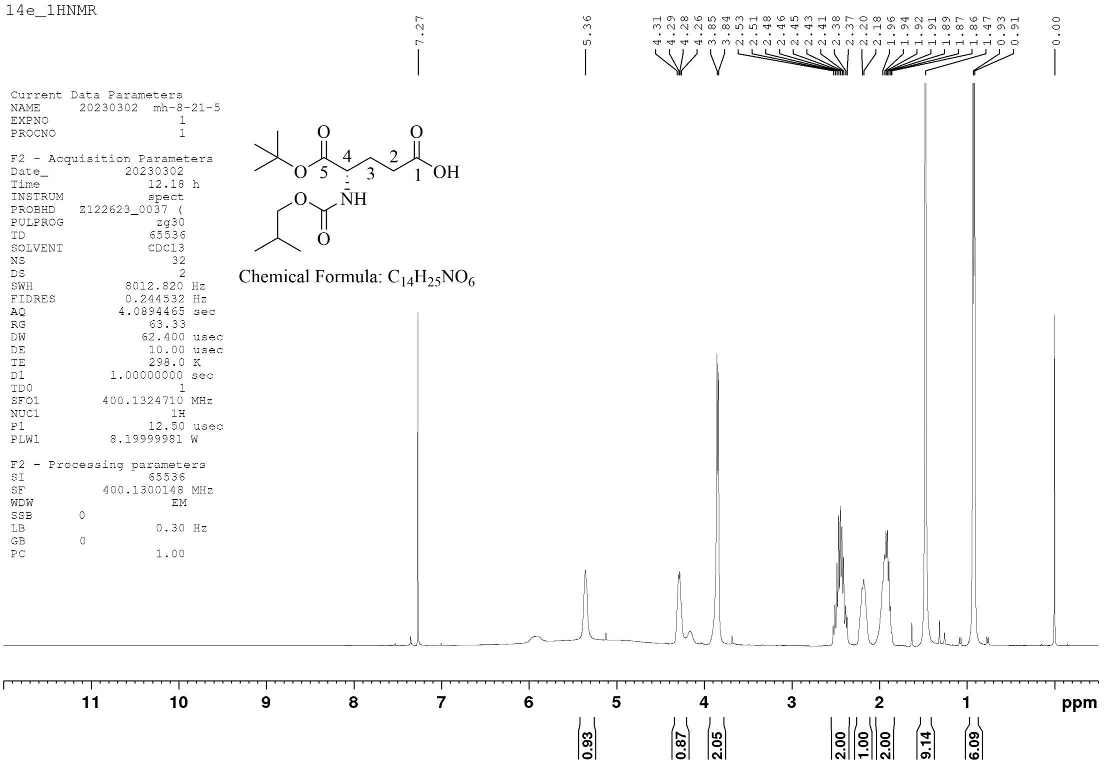

Fig. S11. ^{1}H NMR of intermediate **14e**

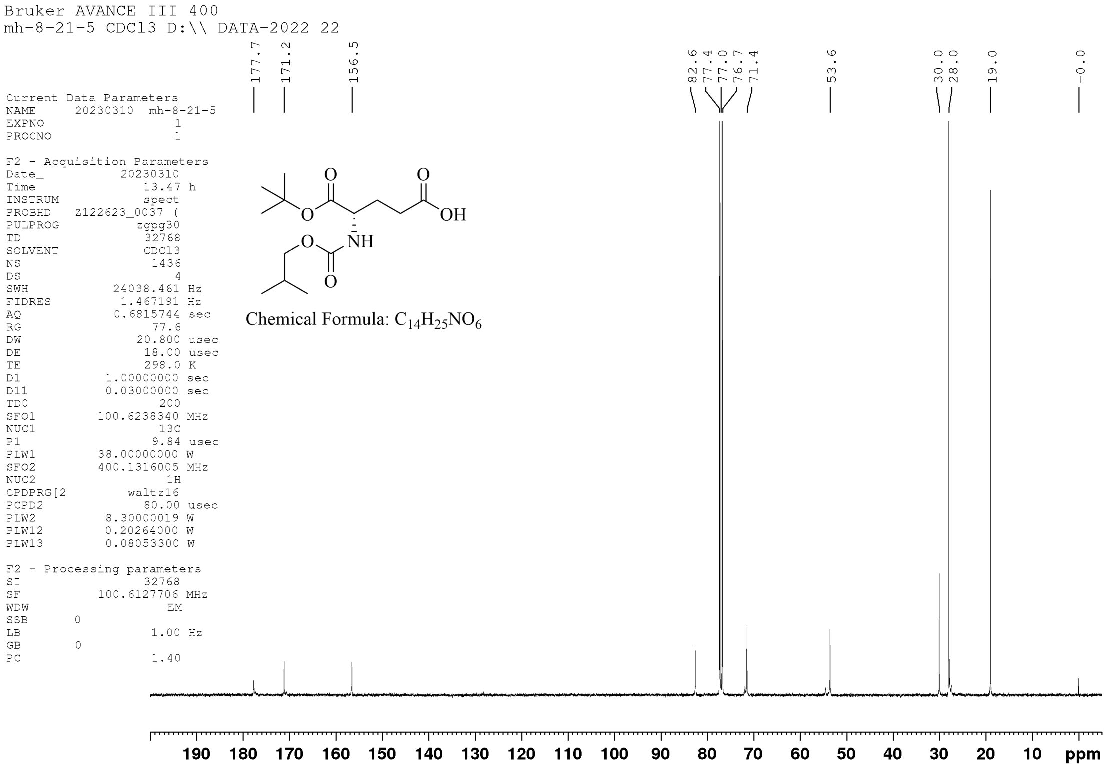

Fig. S12. ^{13}C NMR of intermediate **14e**

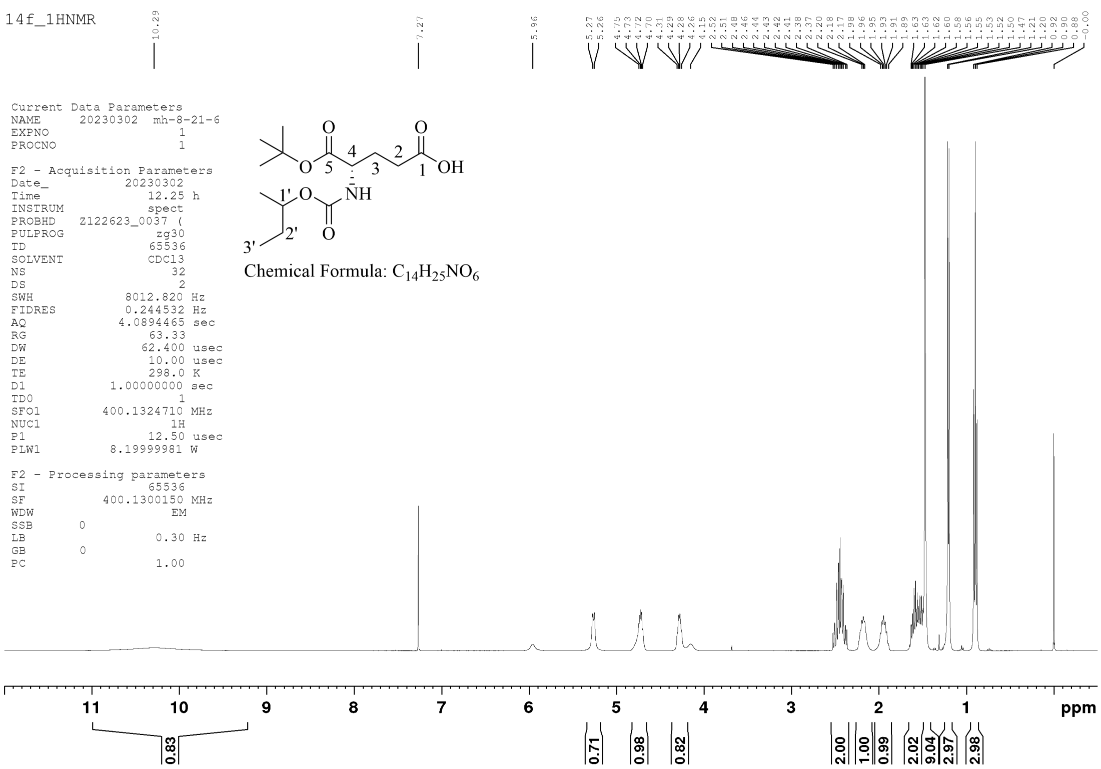

Fig. S13. ^{1}H NMR of intermediate **14f**

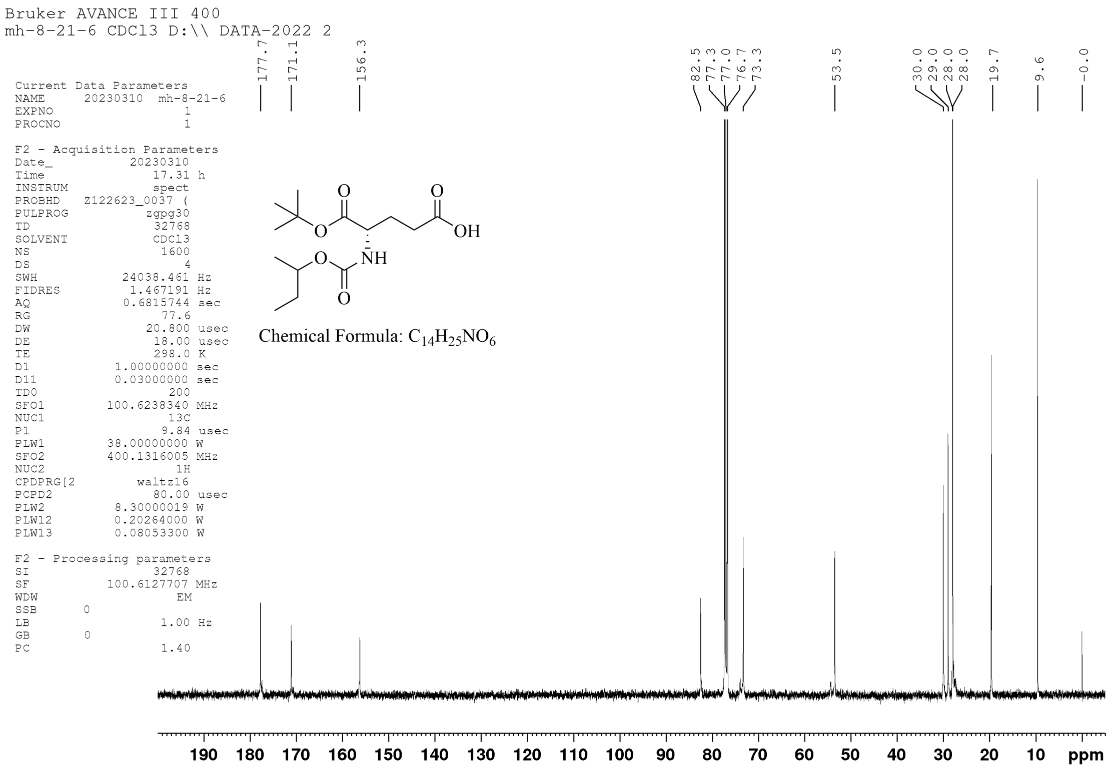

Fig. S14. ^{13}C NMR of intermediate **14f**

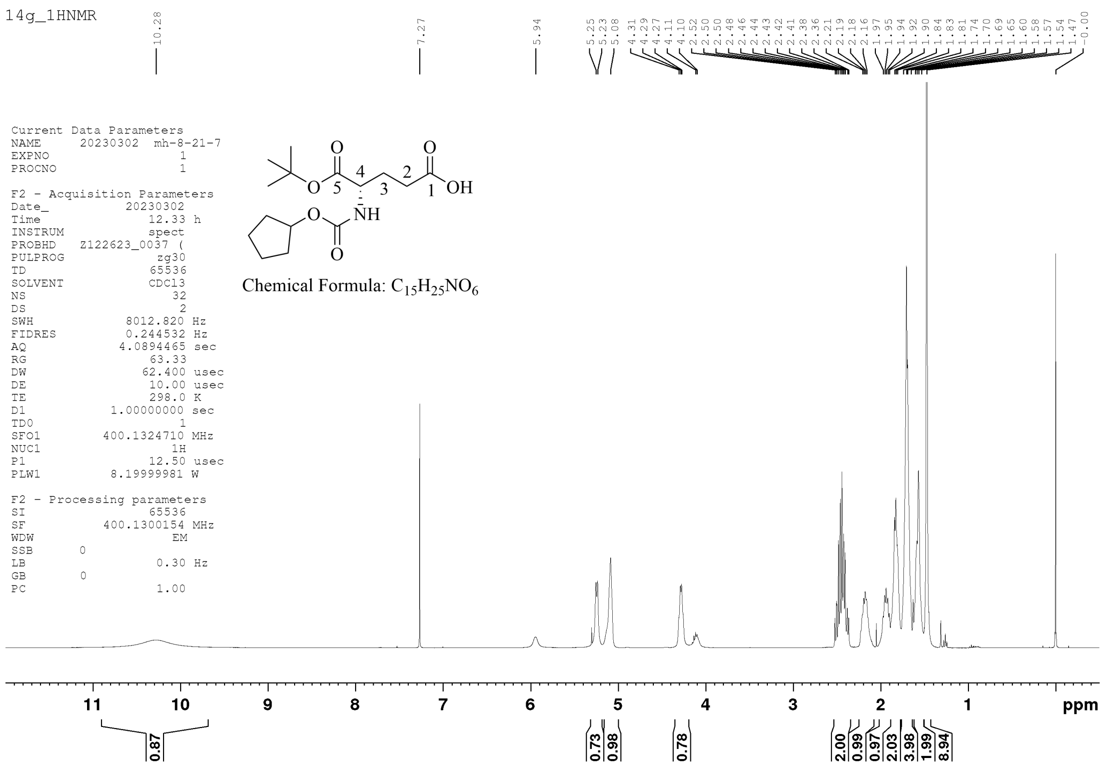

Fig. S15. ^{1}H NMR of intermediate **14g**

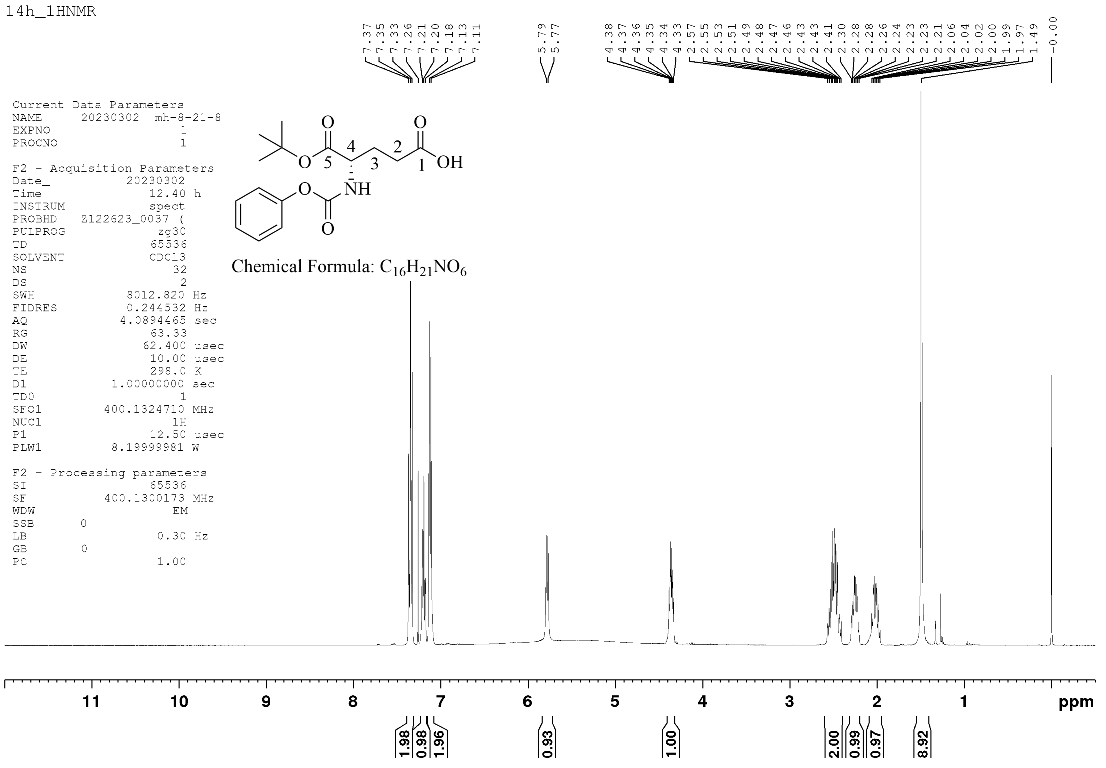

Fig. S16. ^{1}H NMR of intermediate **14h**

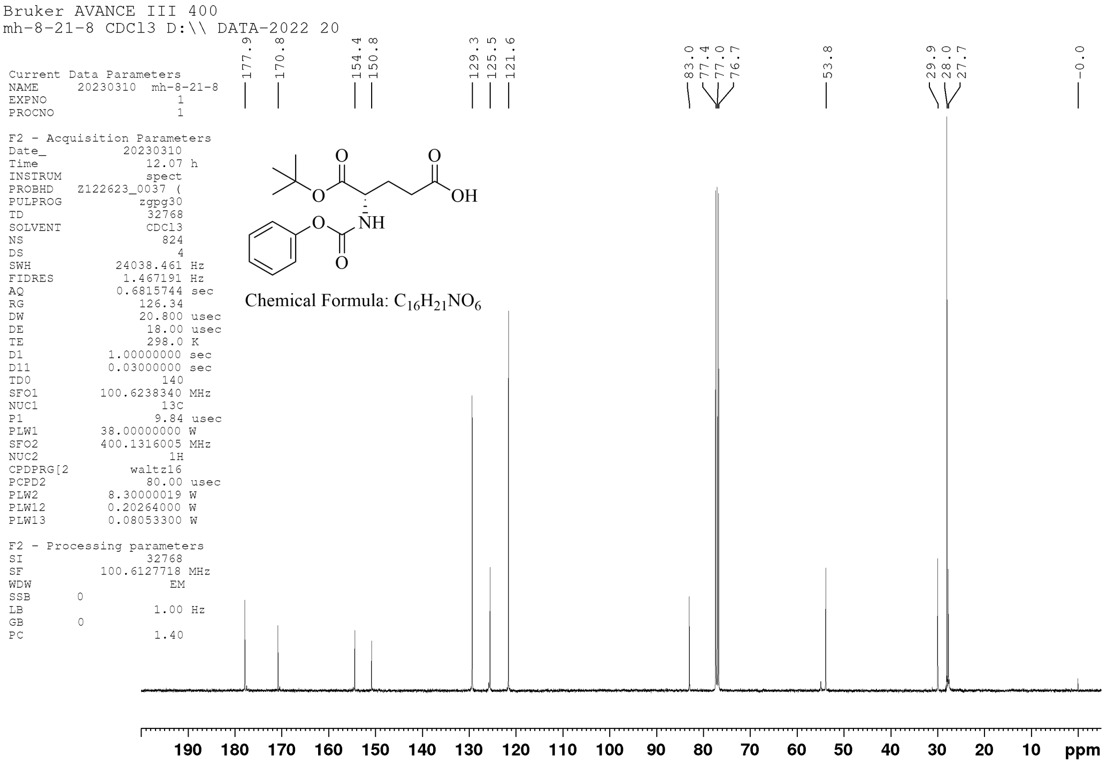

Fig. S17. ^{13}C NMR of intermediate **14h**

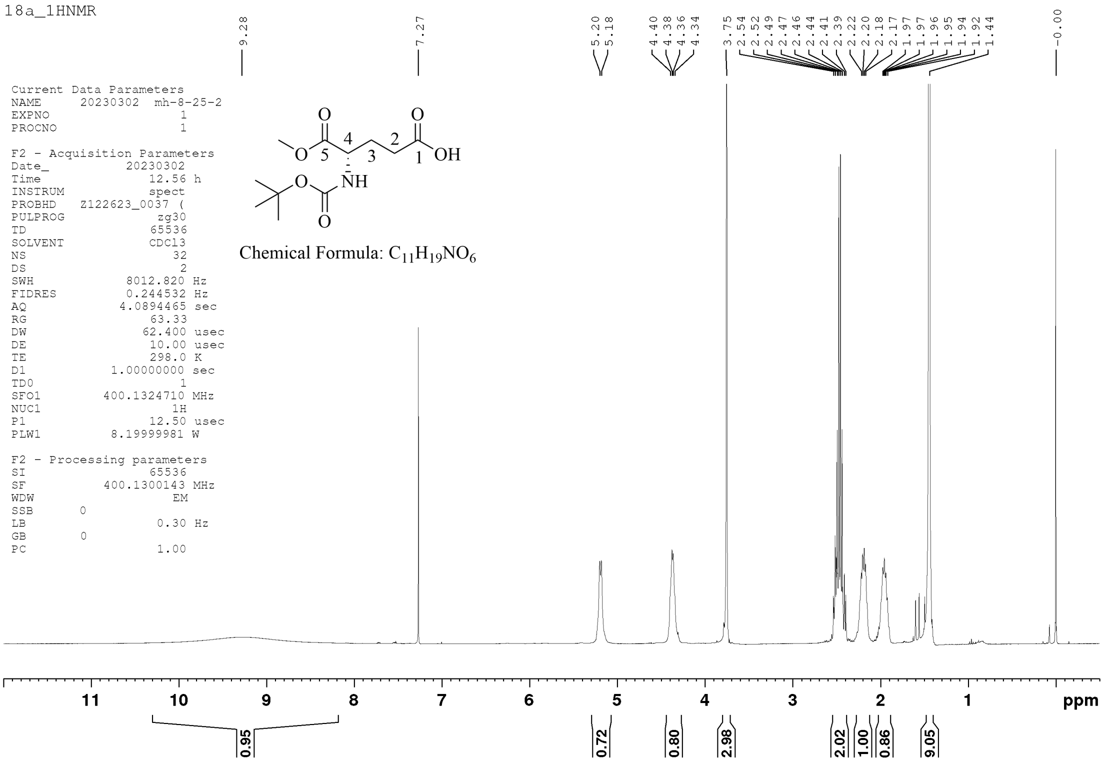

Fig. S18. ^{1}H NMR of intermediate **18a**

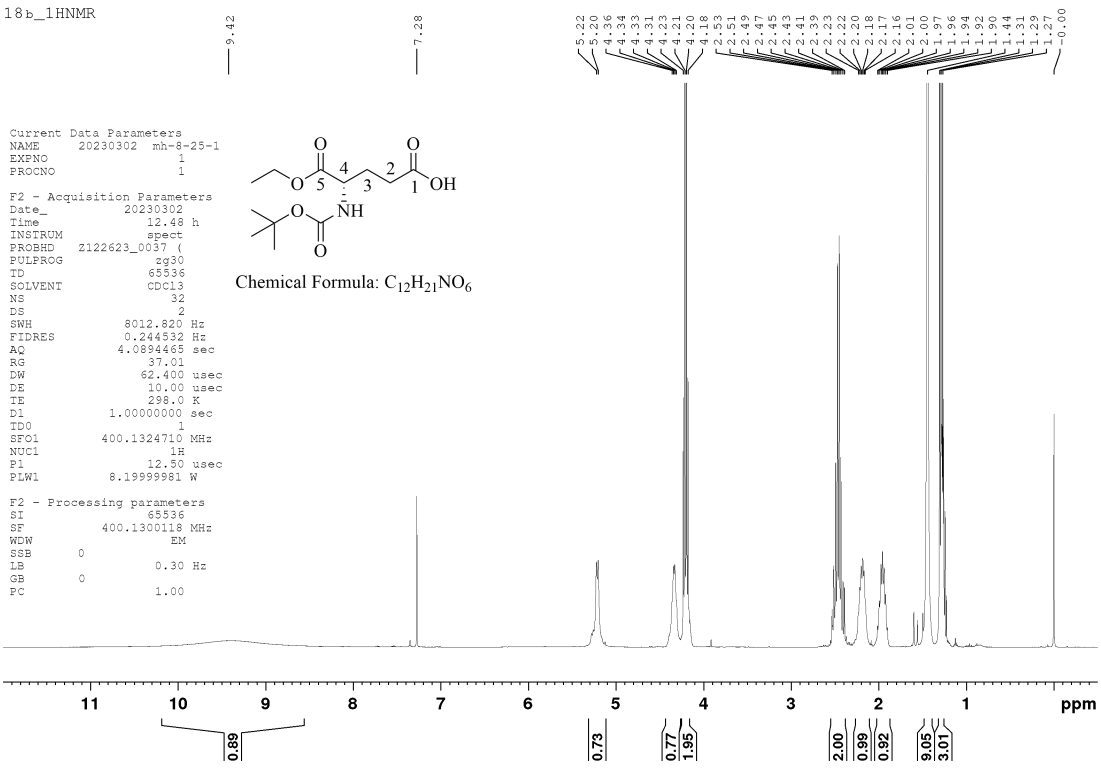

Fig. S19. ^{1}H NMR of intermediate **18b**

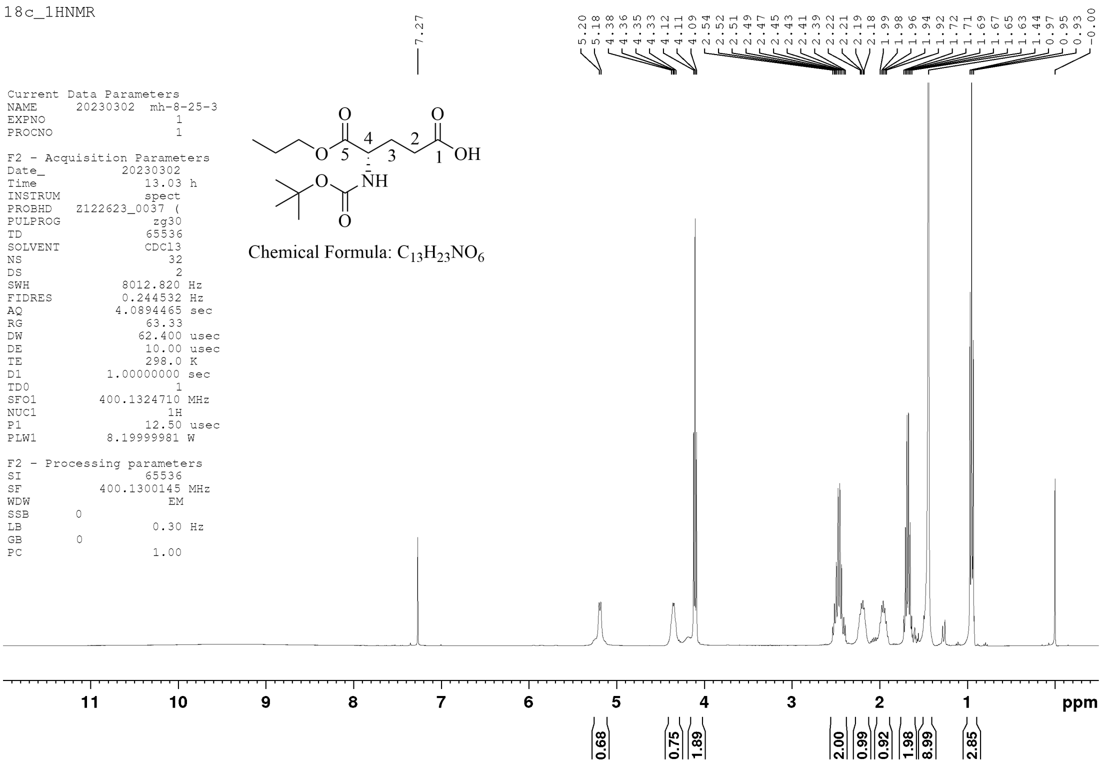

Fig. S20. ^{1}H NMR of intermediate **18c**

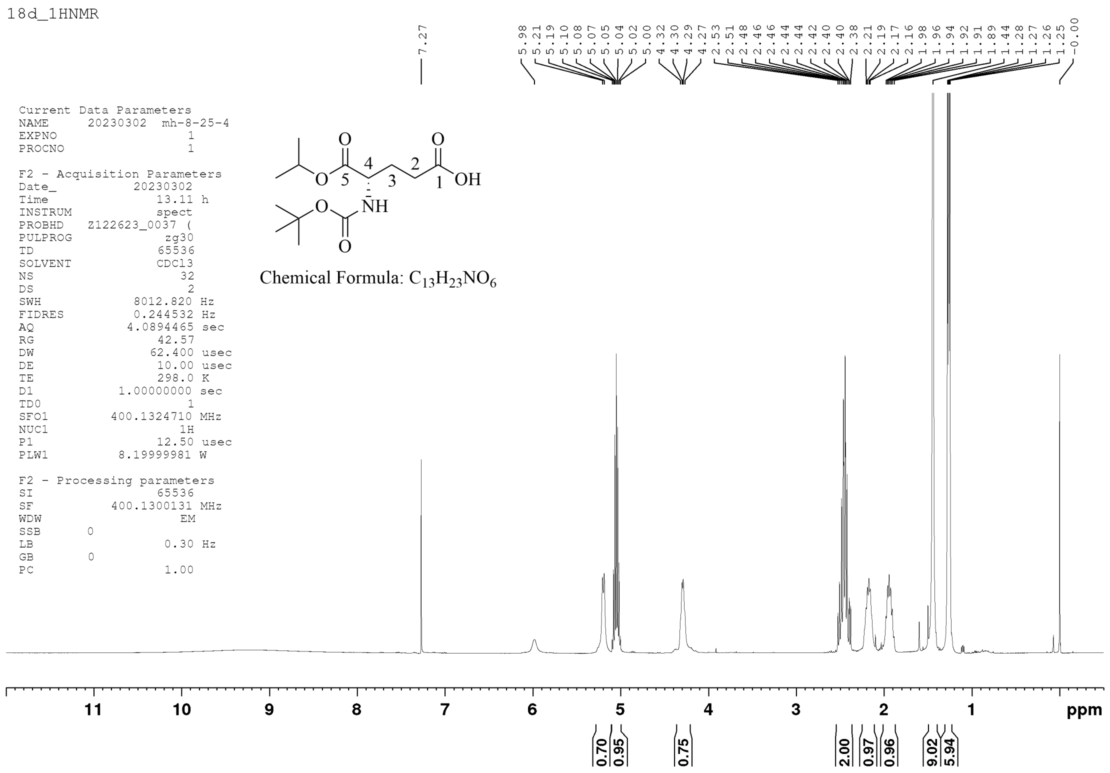

Fig. S21. ^{1}H NMR of intermediate **18d**

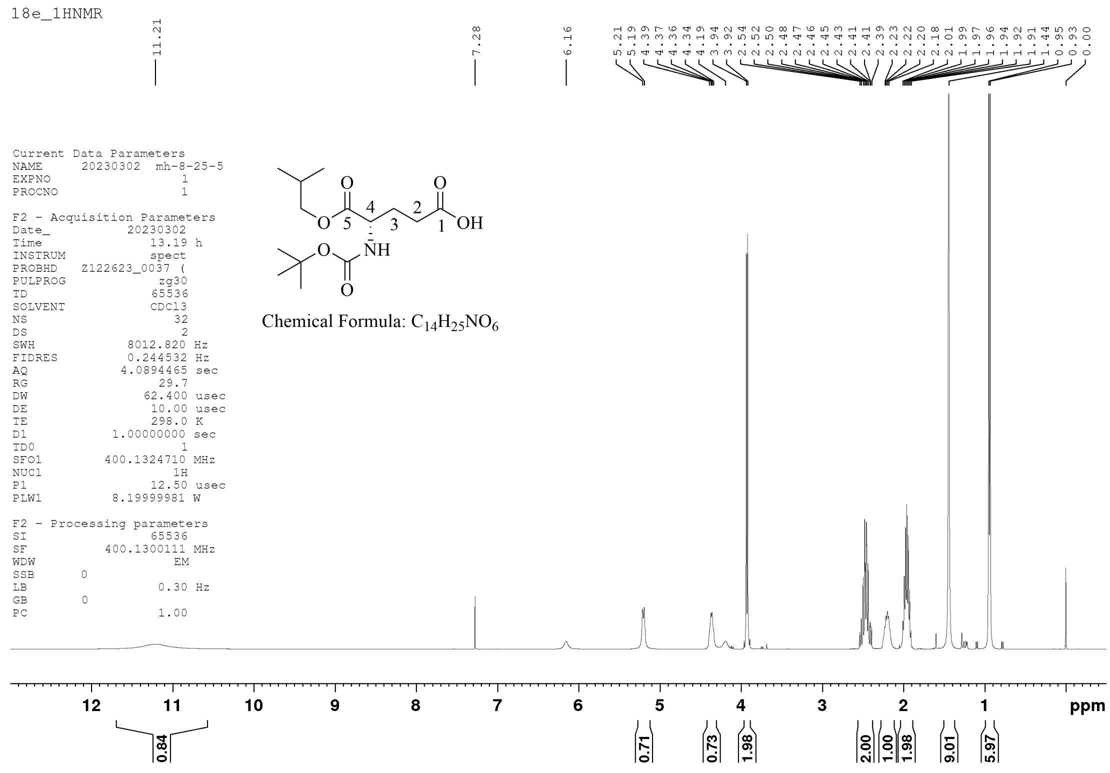

Fig. S22. ^{1}H NMR of intermediate **18e**

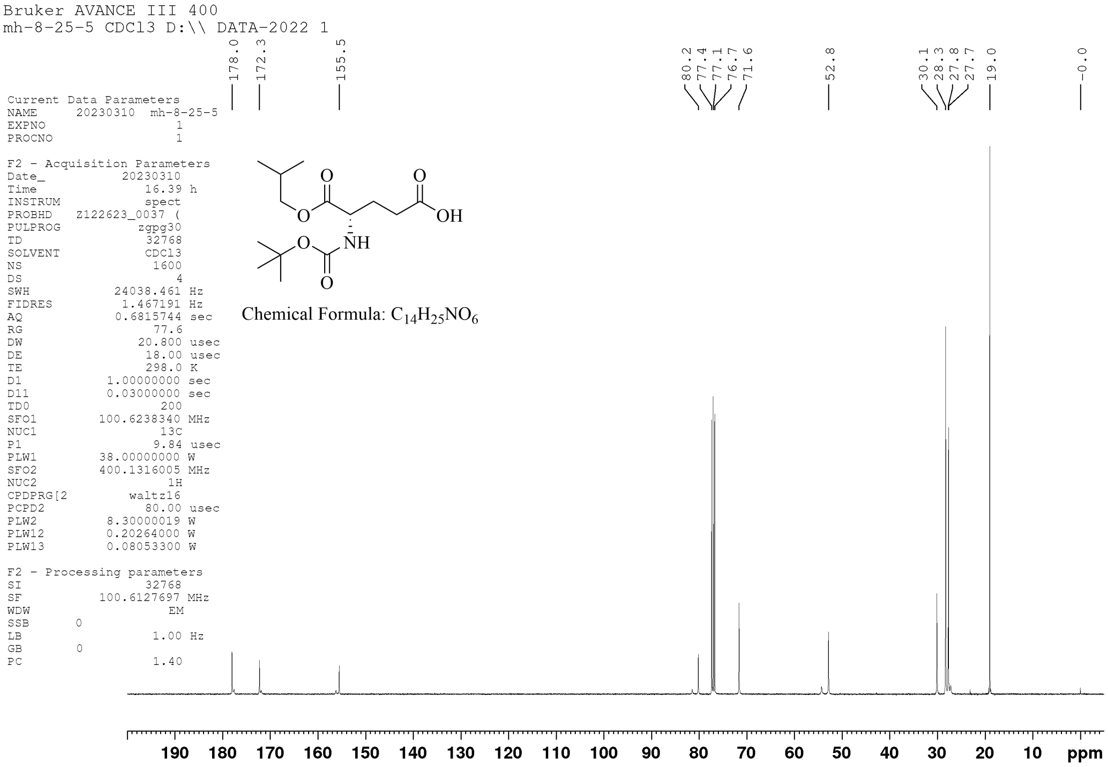

Fig. S23. ^{13}C NMR of intermediate **18e**

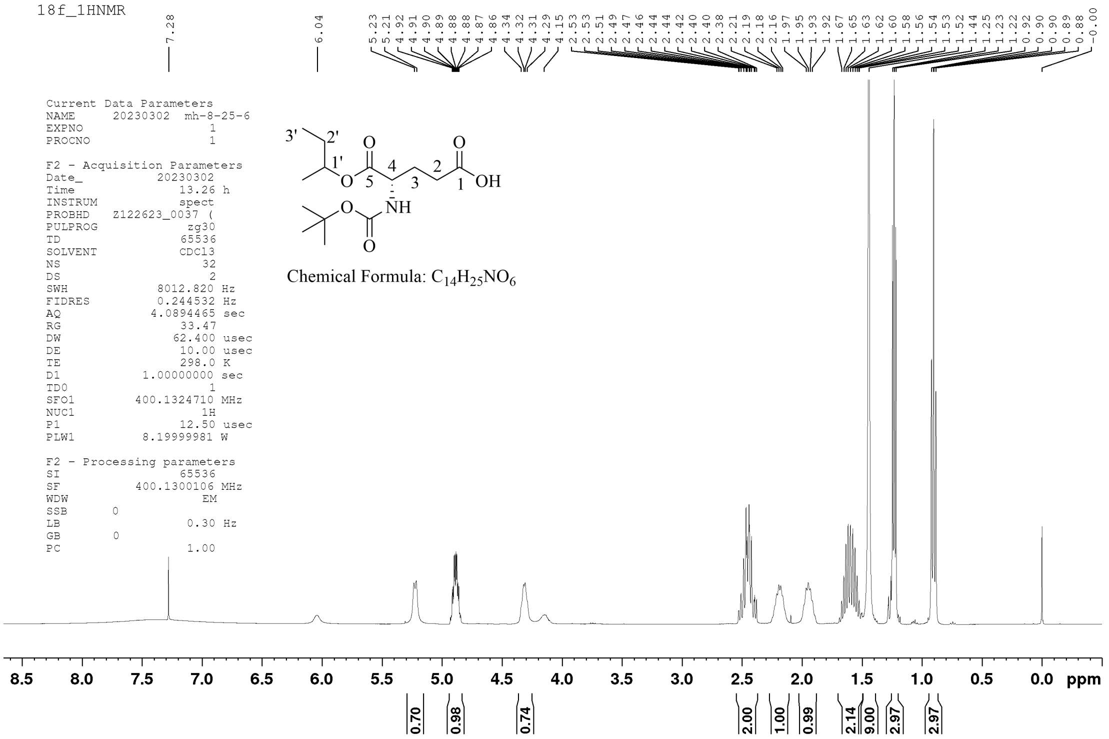

Fig. S24. ^{1}H NMR of intermediate **18f**

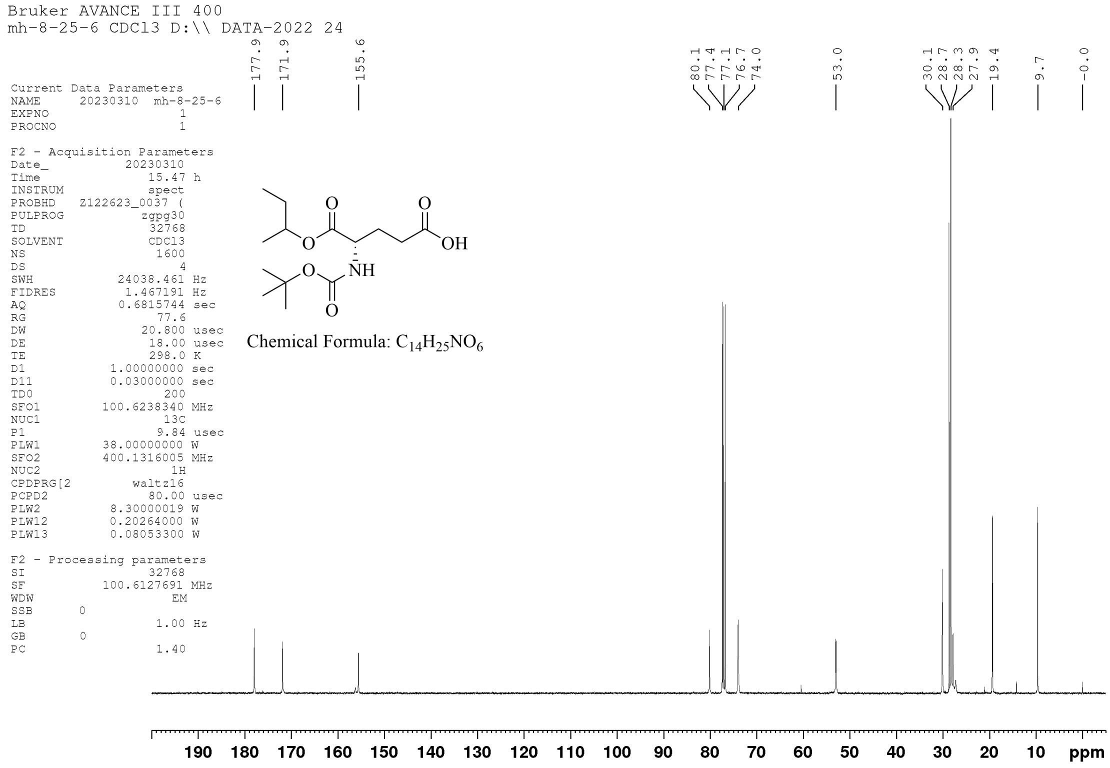

Fig. S25. ^{13}C NMR of intermediate **18f**

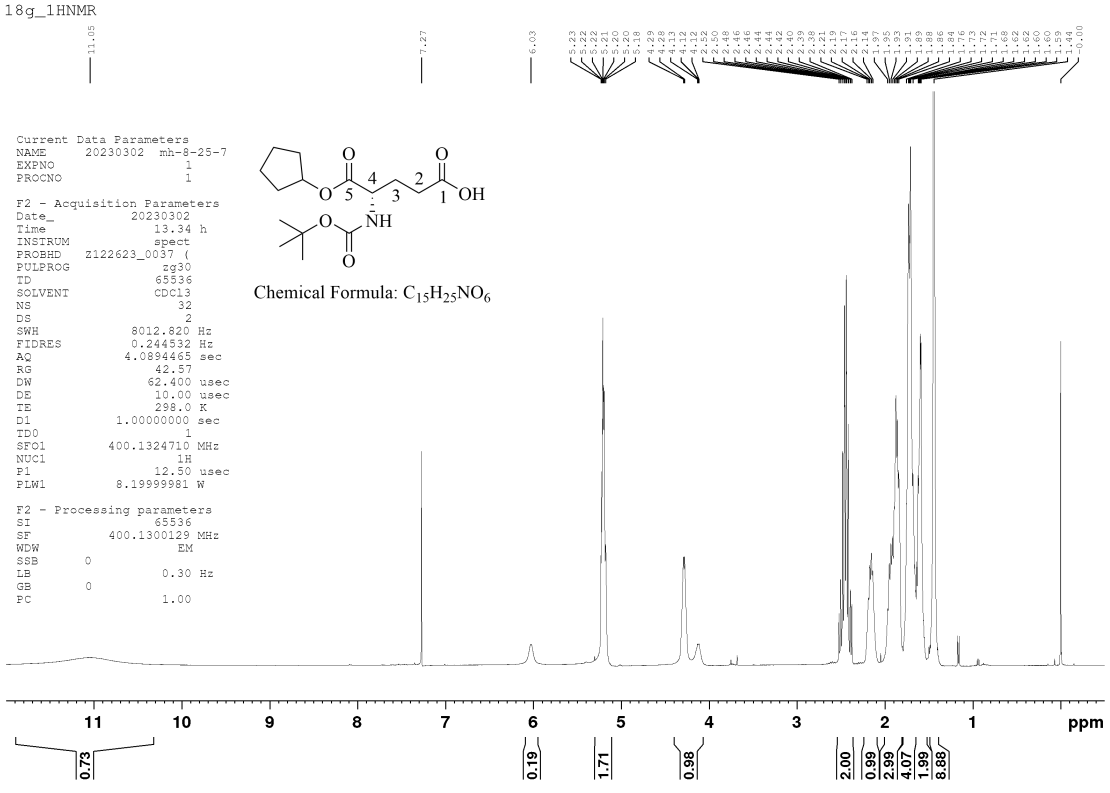

Fig. S26. ^{1}H NMR of intermediate **18g**

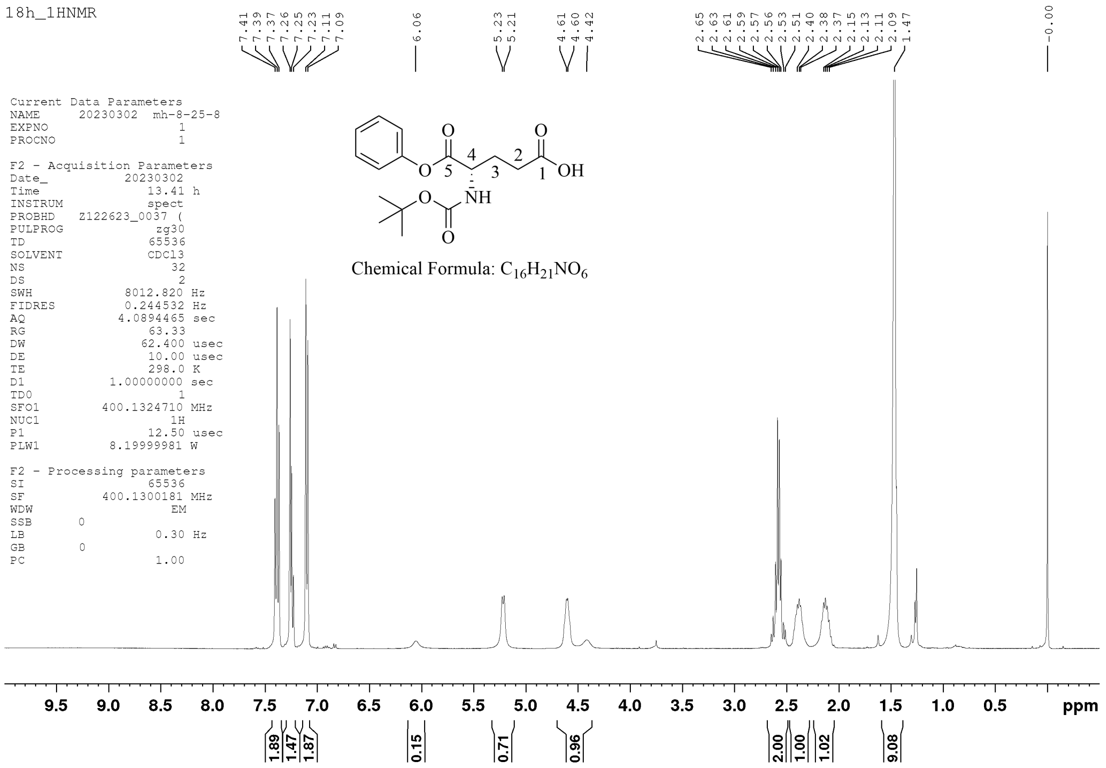

Fig. S27. ^{1}H NMR of intermediate **18h**

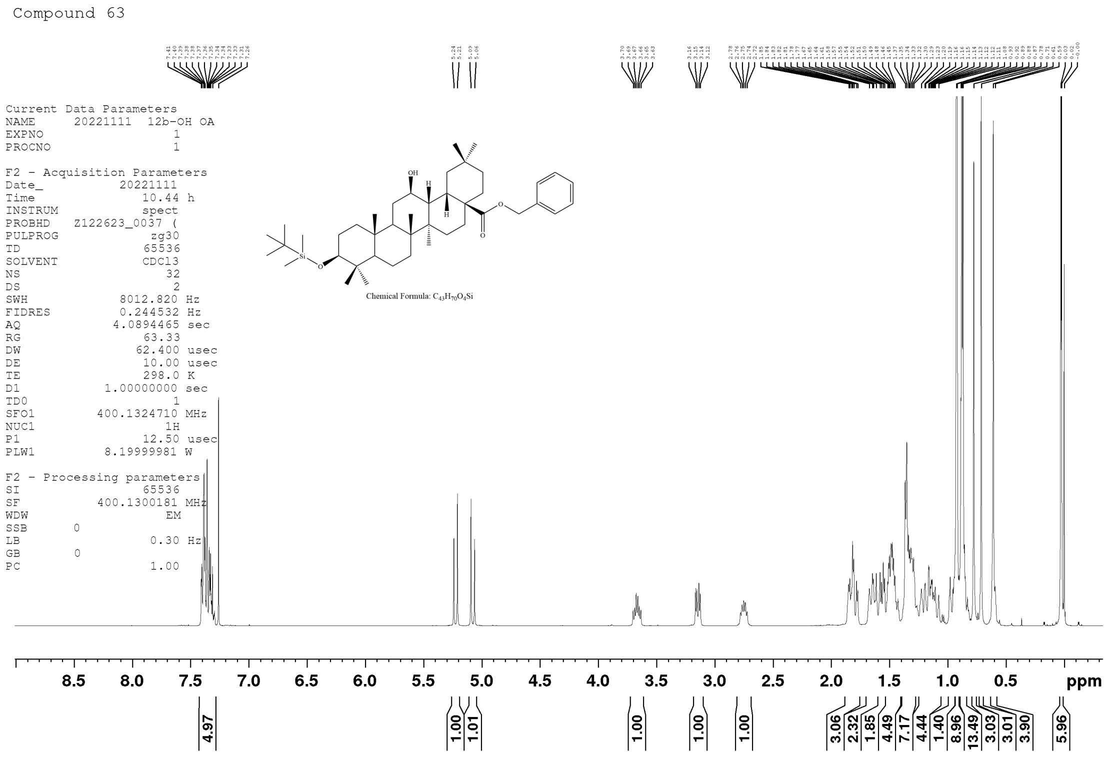

Fig. S28. ^{1}H NMR of compound **8**

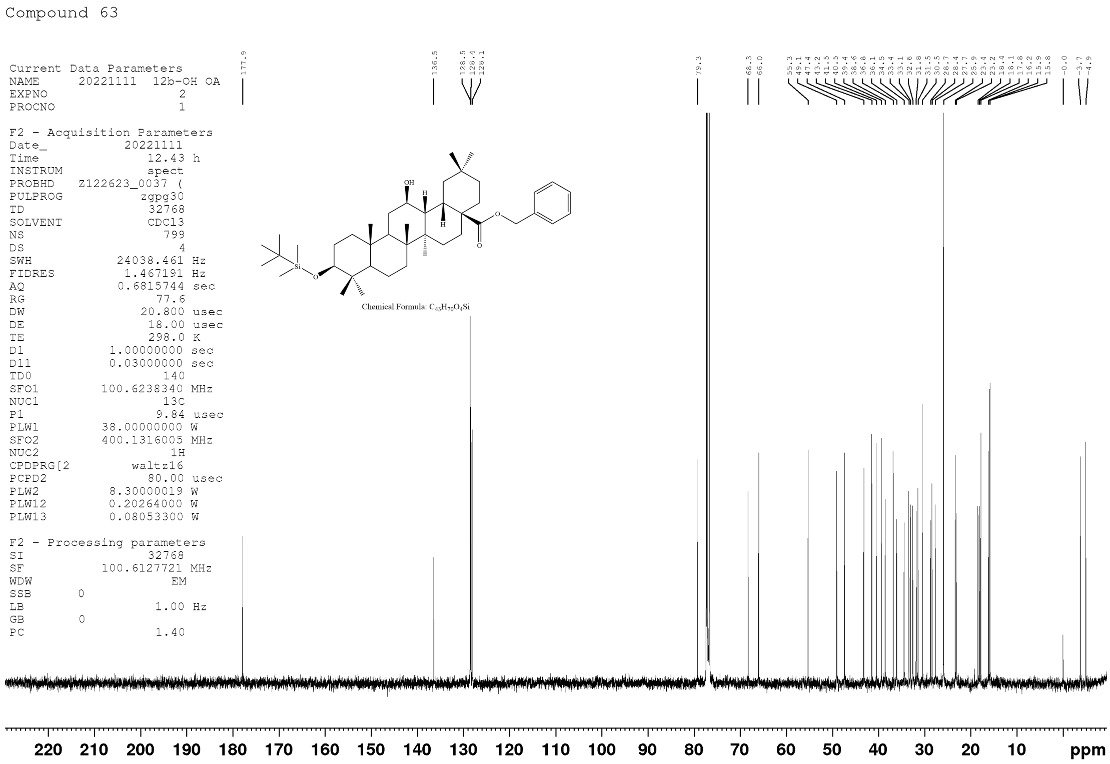

Fig. S29. ^{13}C NMR of compound **8**

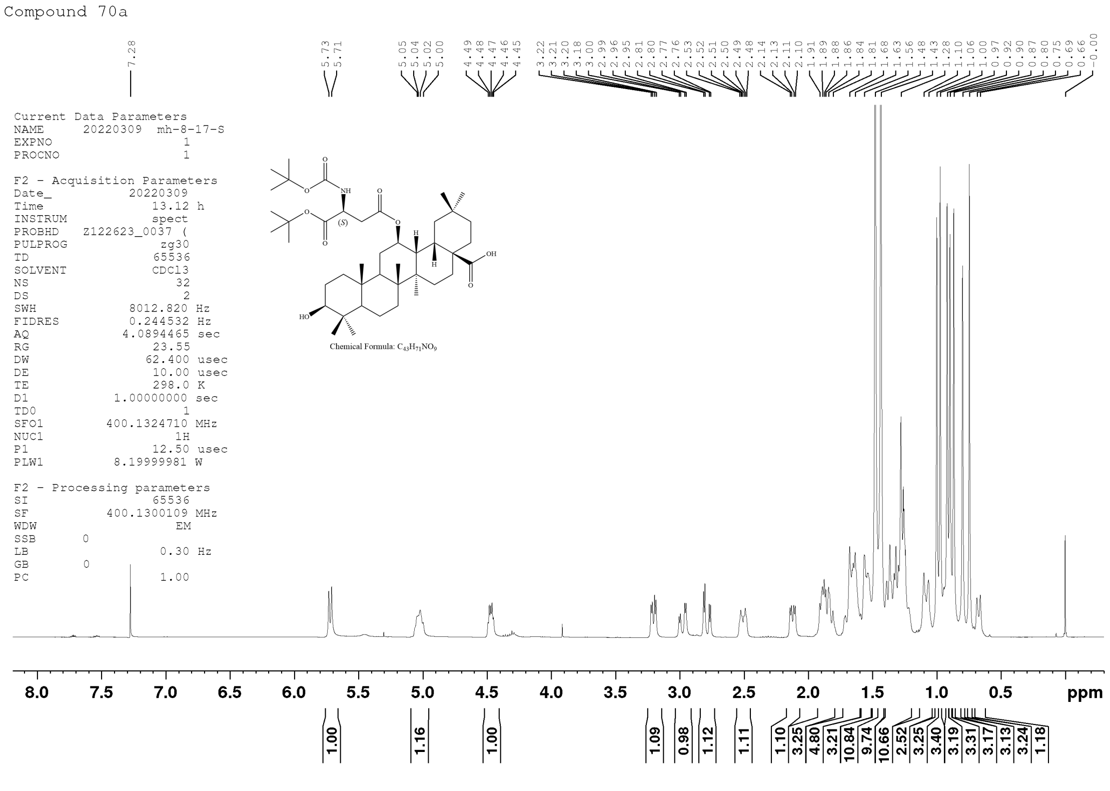

Fig. S30. ^{1}H NMR of compound **9a**

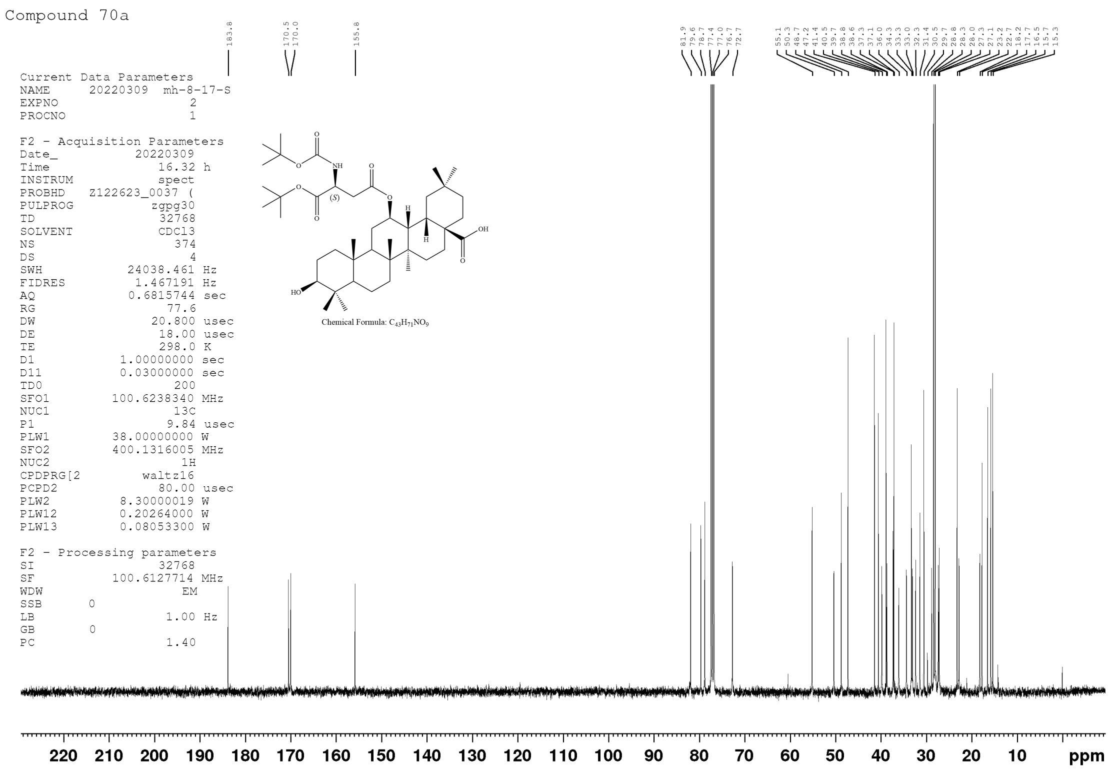

Fig. S31. ^{13}C NMR of compound **9a**

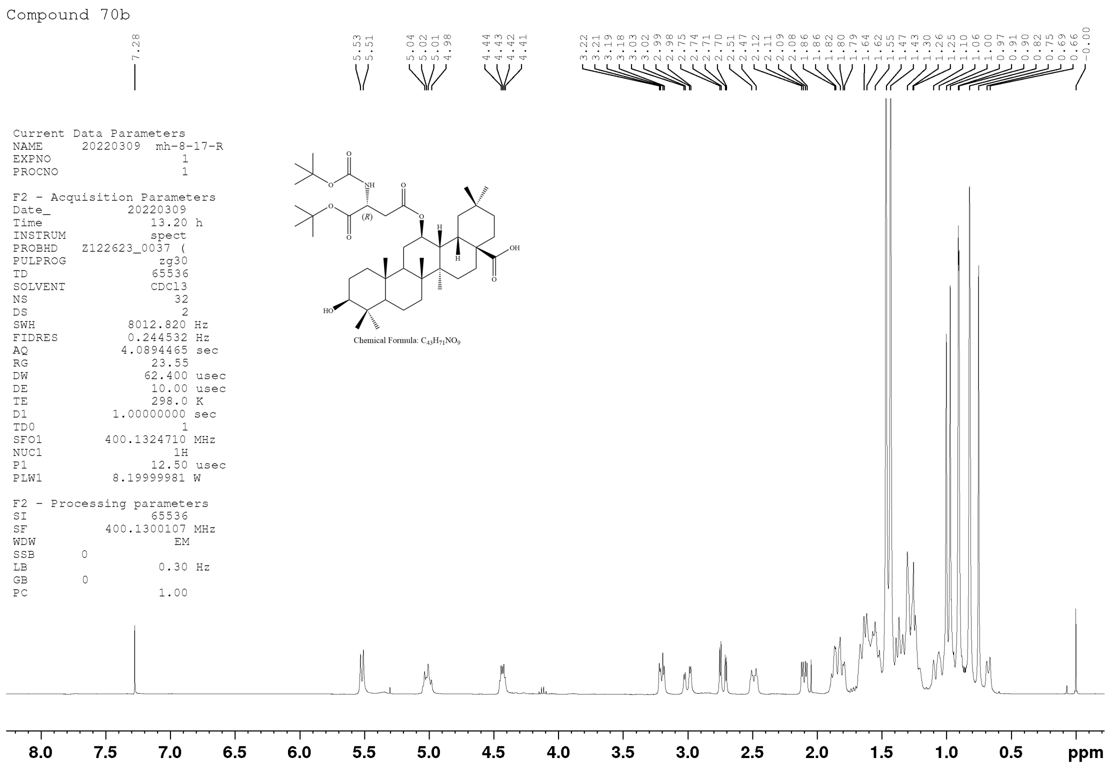

Fig. S32. ^{1}H NMR of compound **9b**

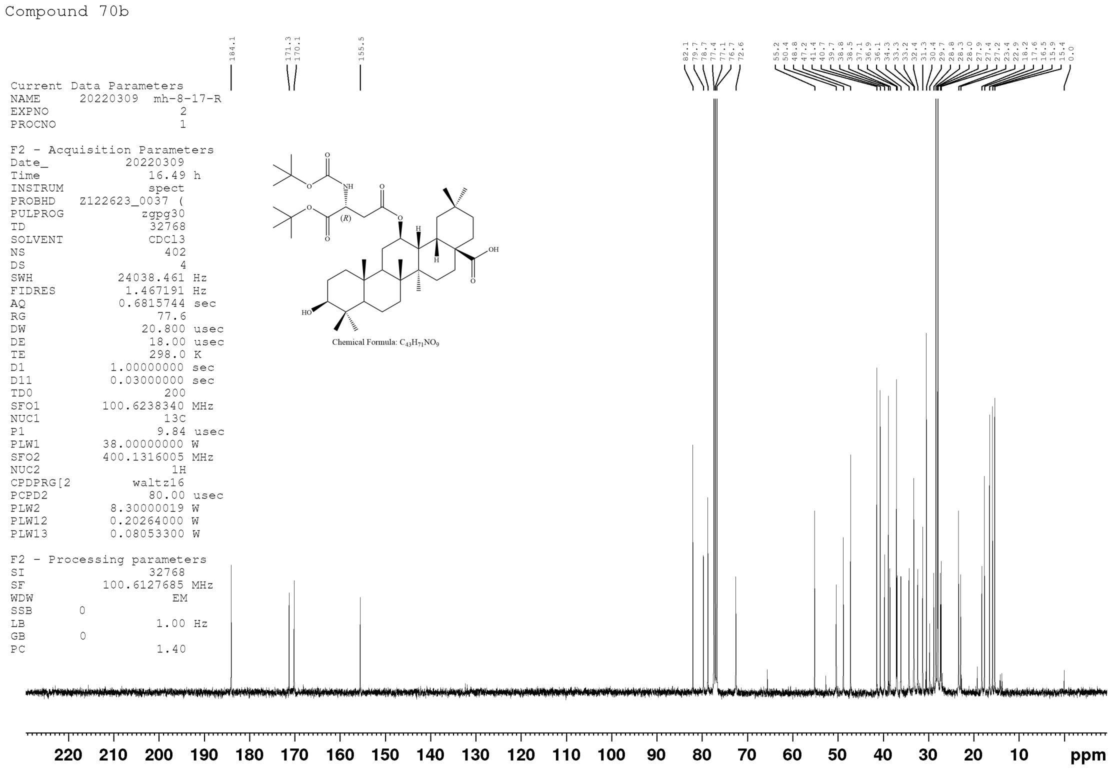

Fig. S33. ^{13}C NMR of compound **9b**

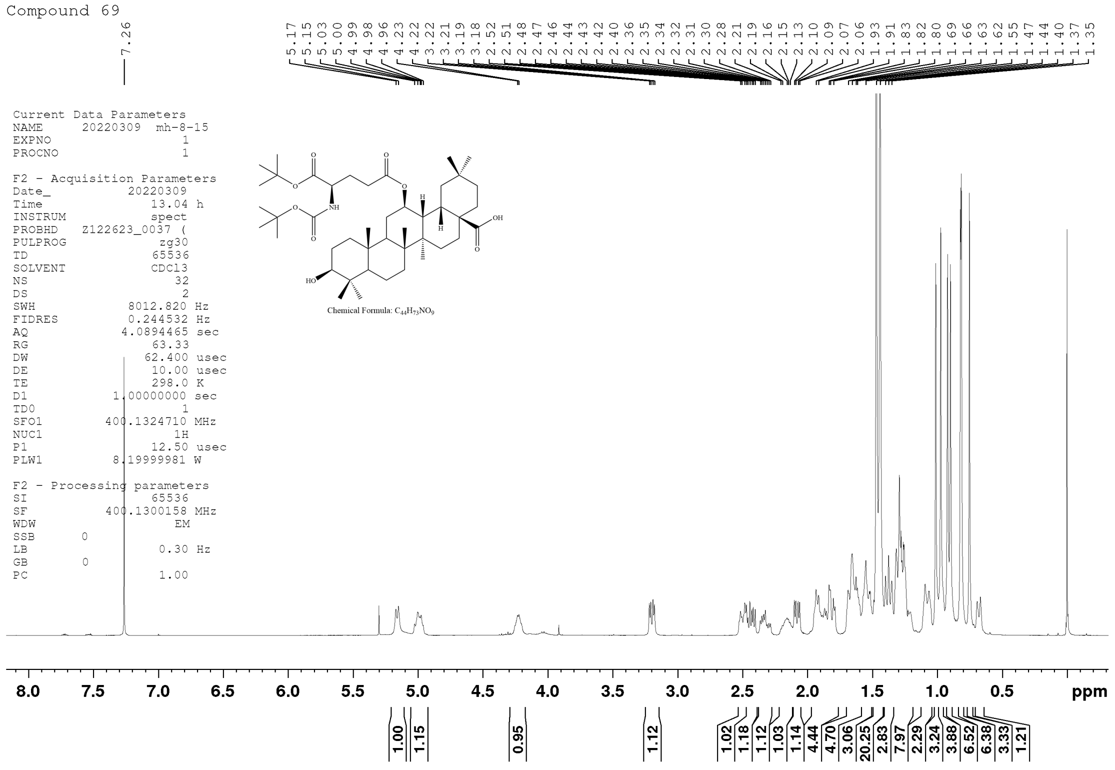

Fig. S34. ^{1}H NMR of compound **10a**

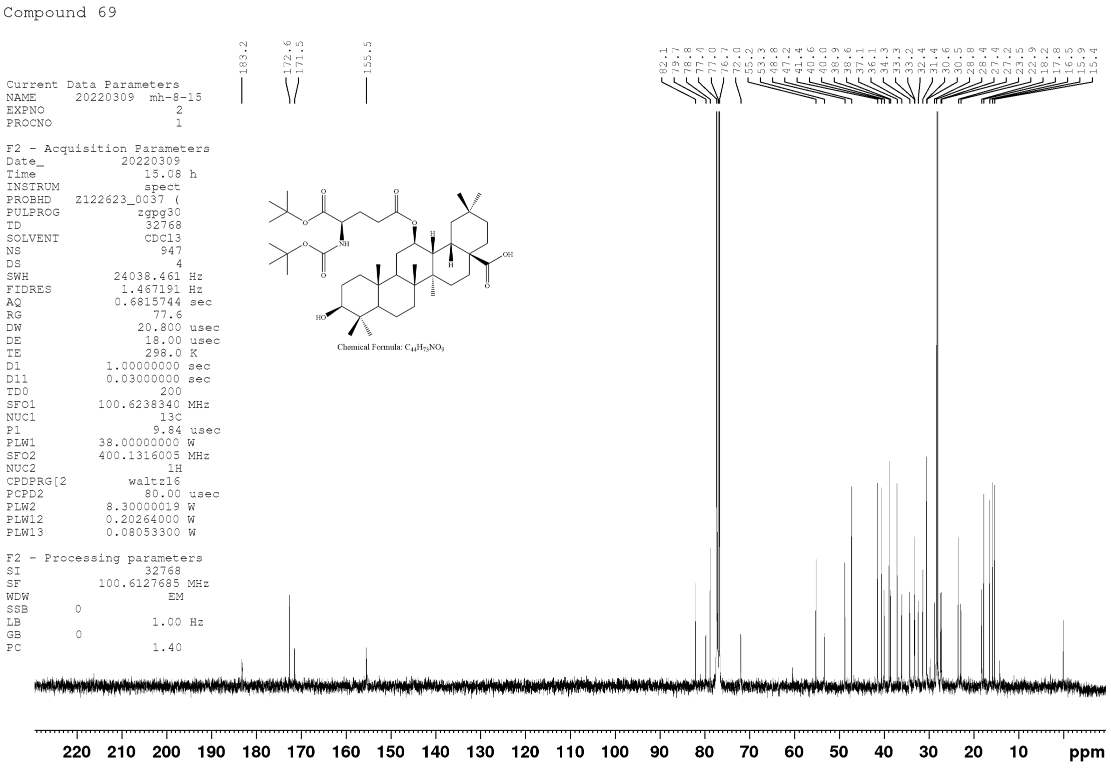

Fig. S35. ^{13}C NMR of compound **10a**

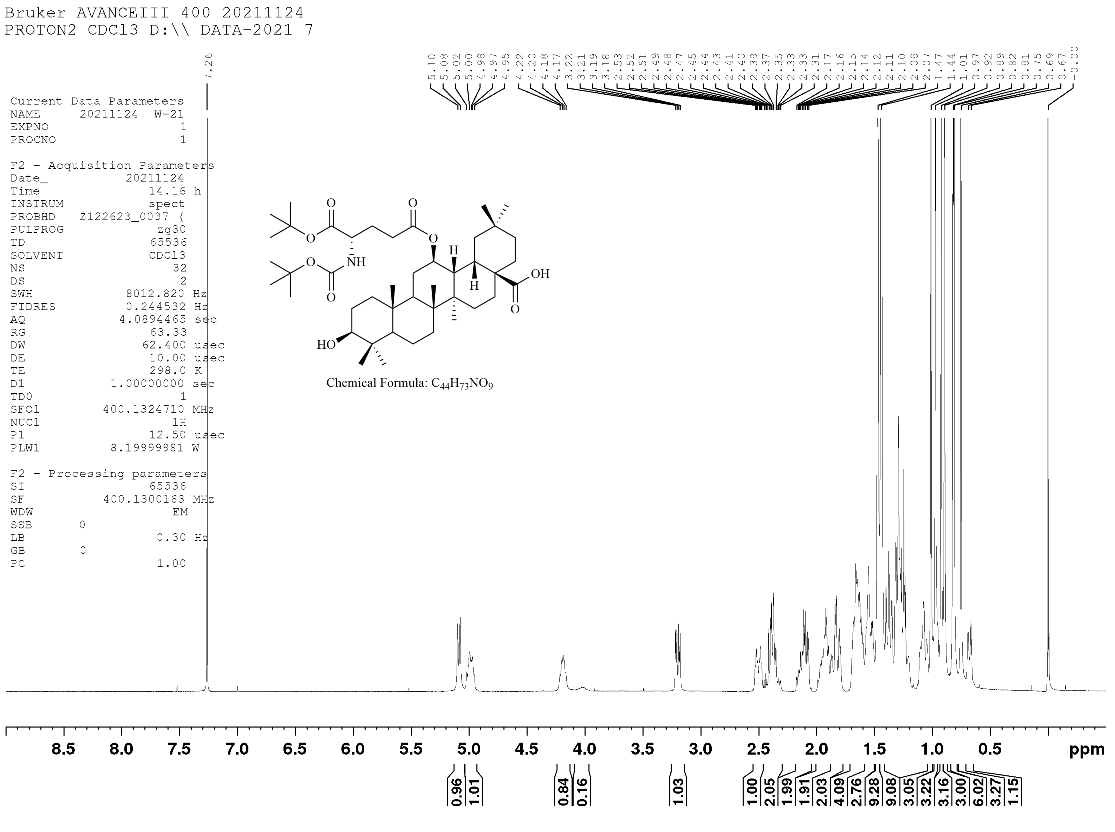

Fig. S36. ^{1}H NMR of compound **10b**

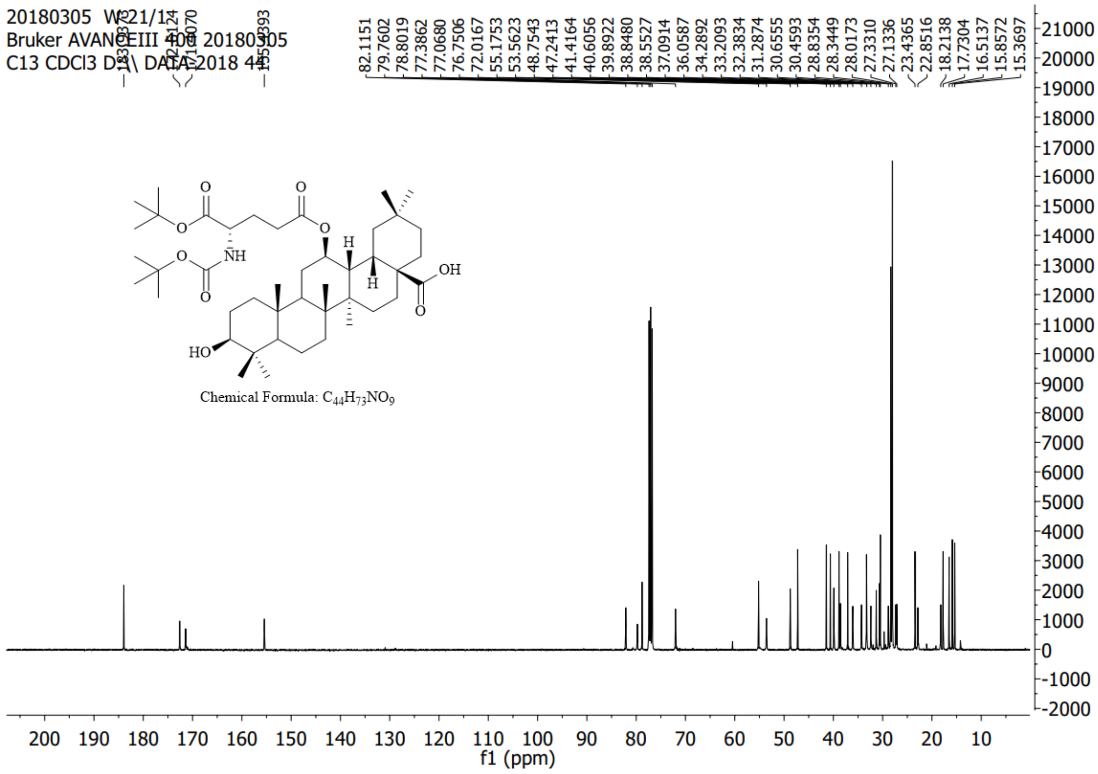

Fig. S37. ^{13}C NMR of compound **10b**

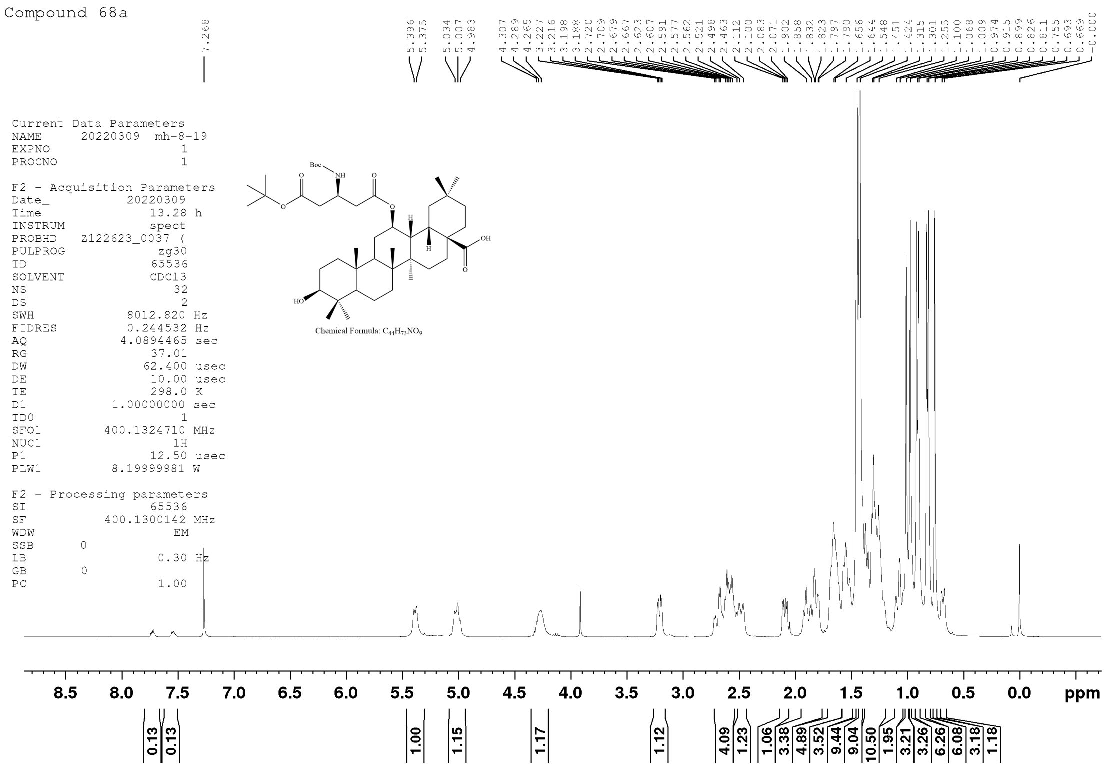

Fig. S38. ^{1}H NMR of compound **10c**

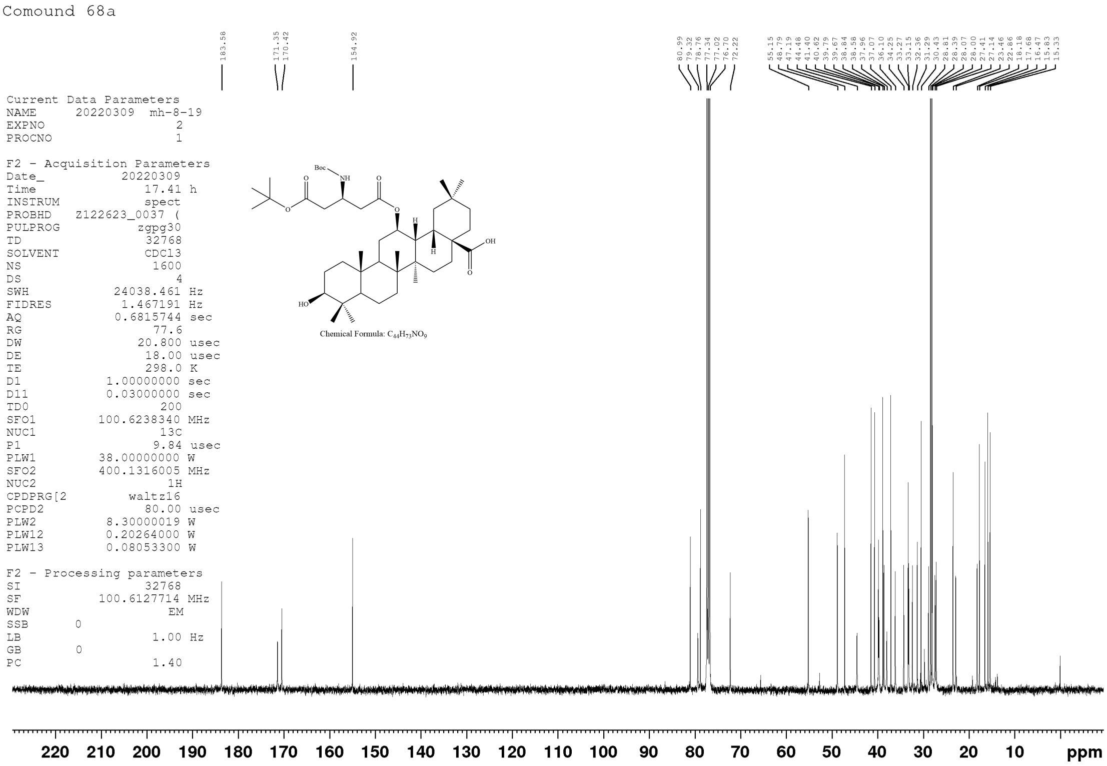

Fig. S39. ^{13}C NMR of compound **10c**

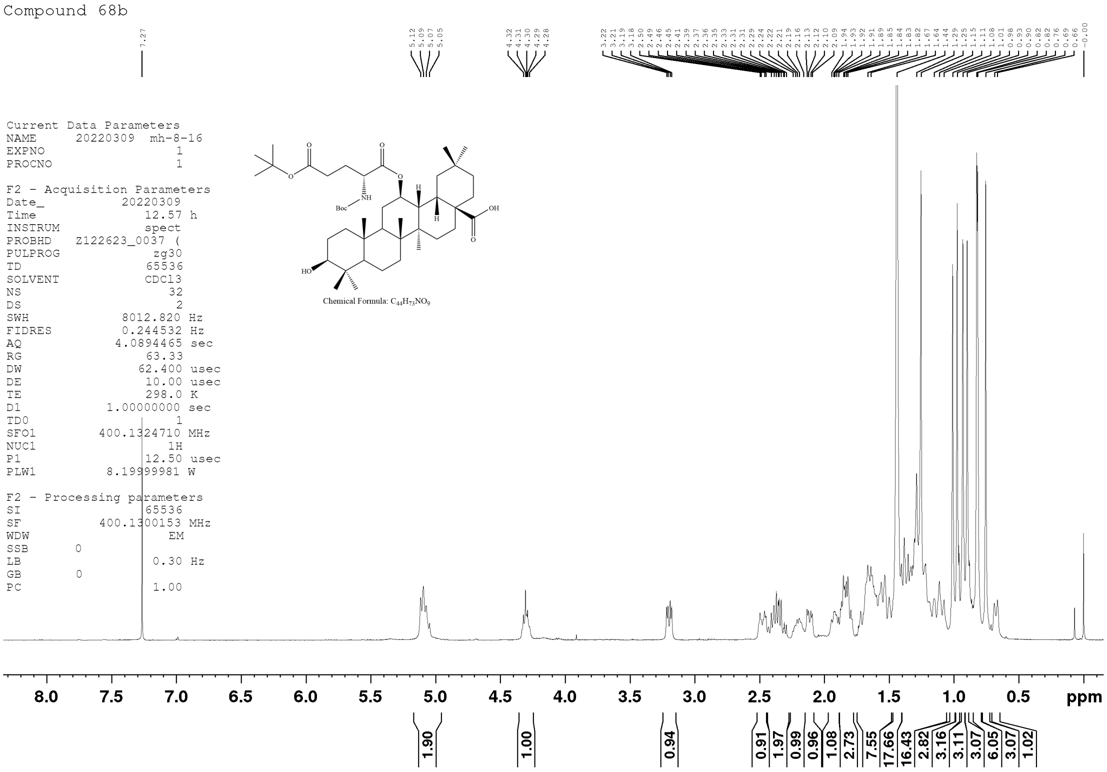

Fig. S40. ^{1}H NMR of compound **10d**

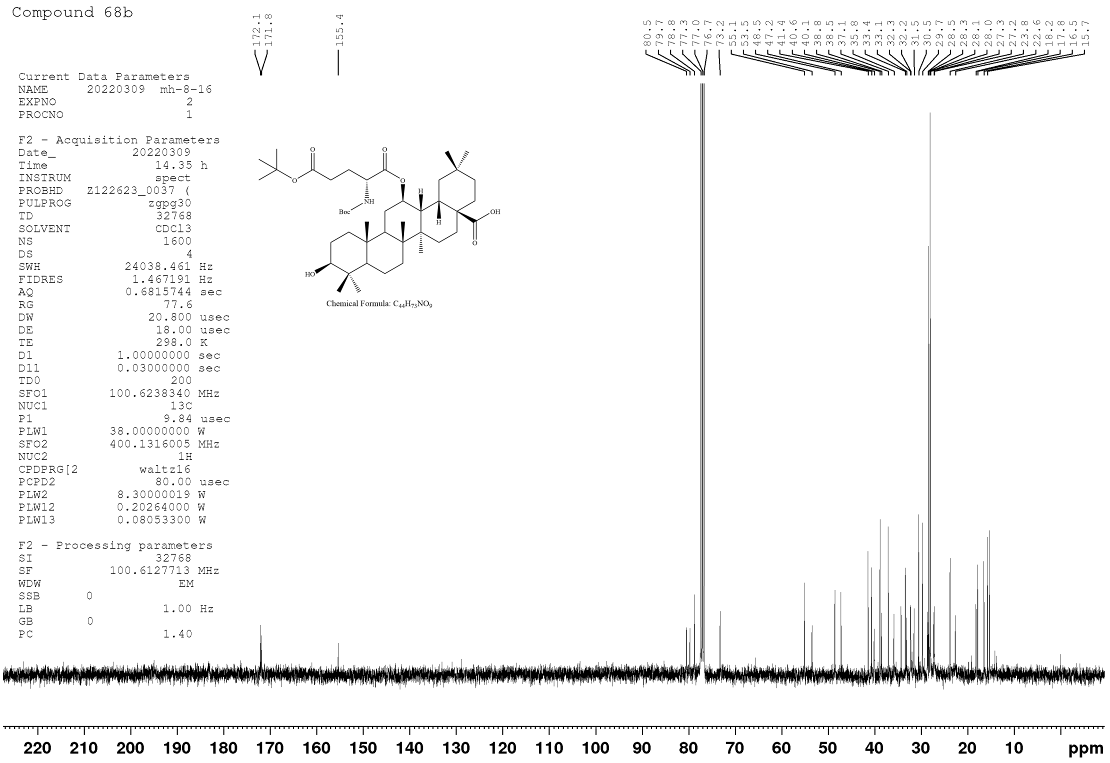

Fig. S41. ^{13}C NMR of compound **10d**

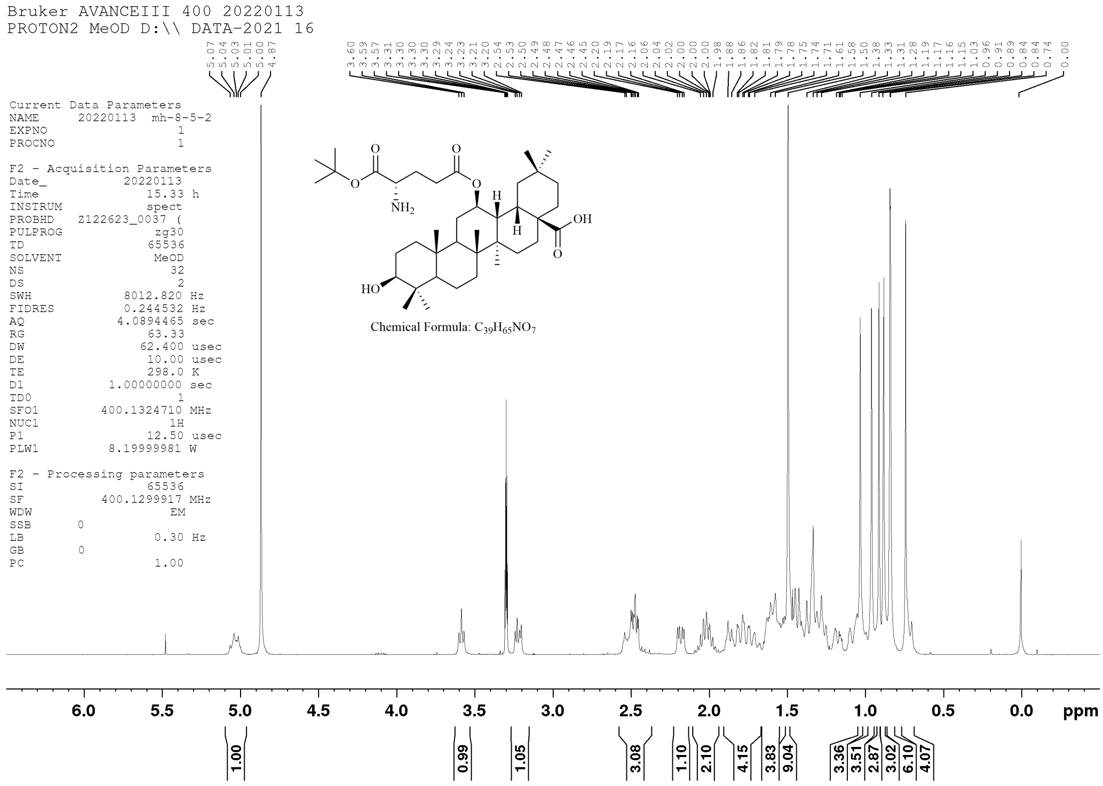

Fig. S42. ^{1}H NMR of compound **11a**

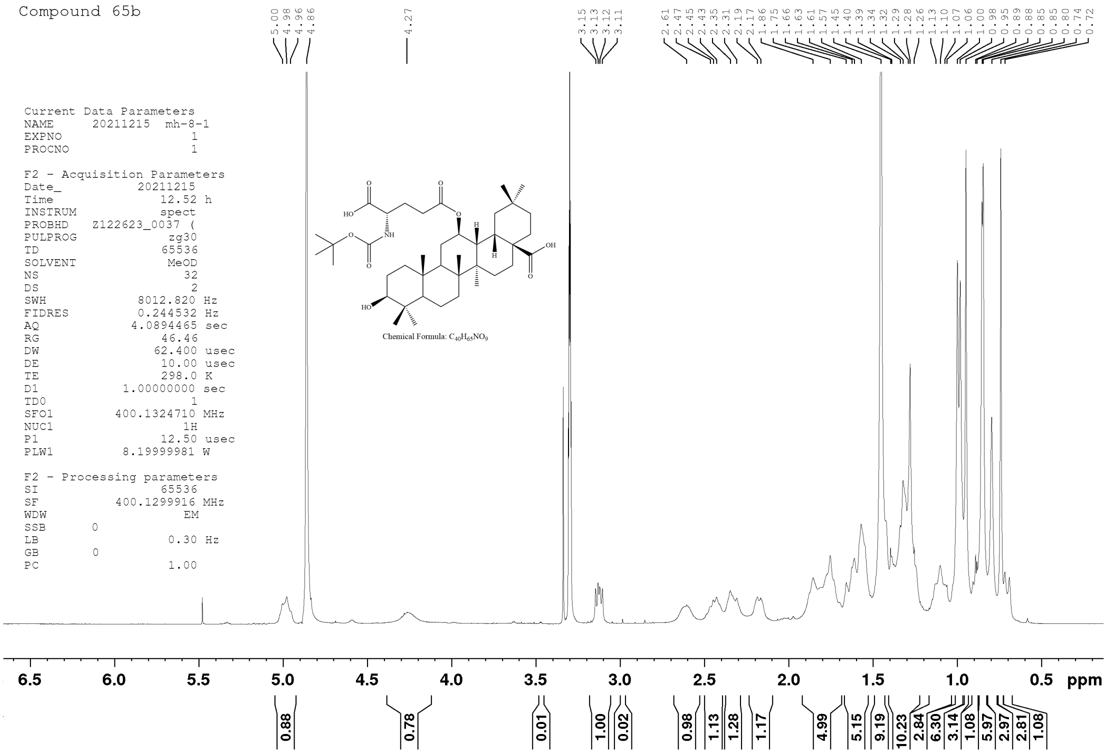

Fig. S43. ^{1}H NMR of compound **11b**

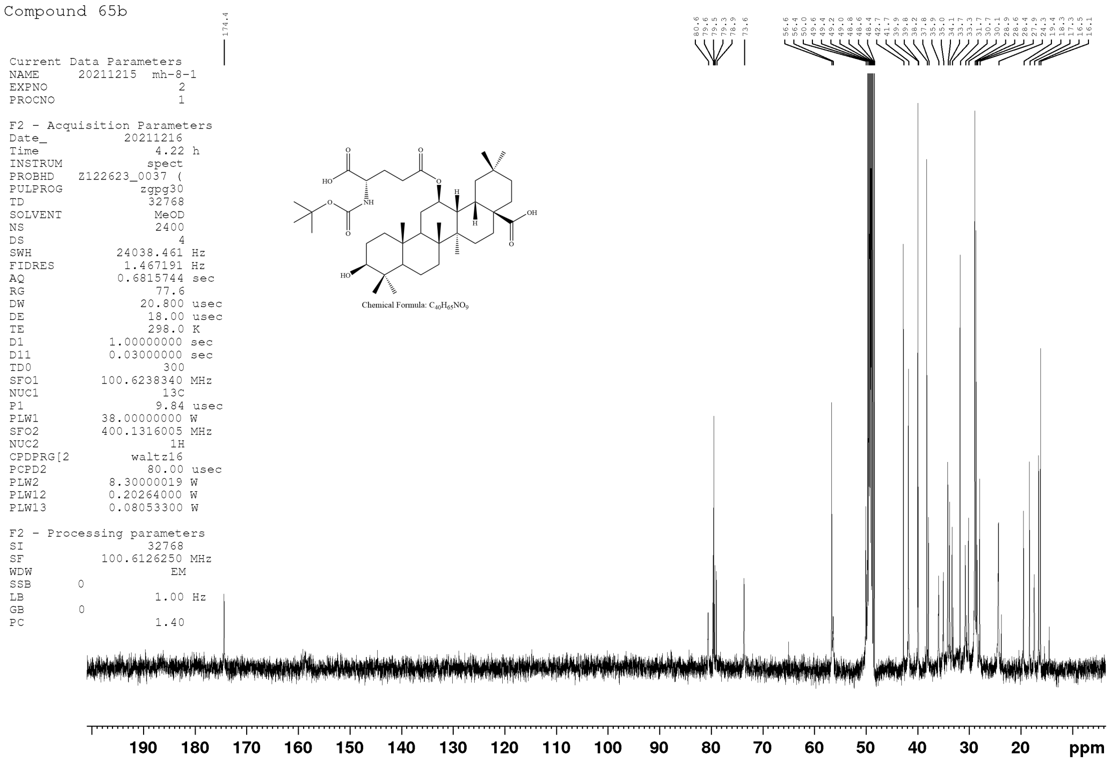

Fig. S44. ^{13}C NMR of compound **11b**

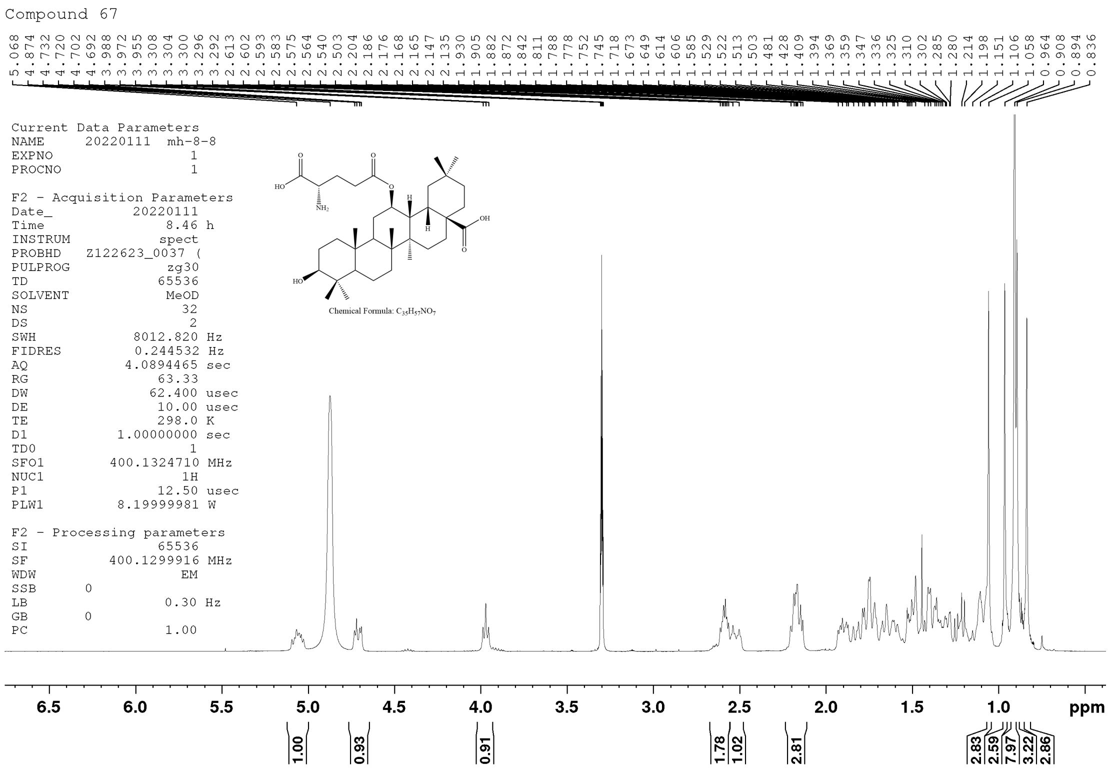

Fig. S45. ^{1}H NMR of compound **11c**

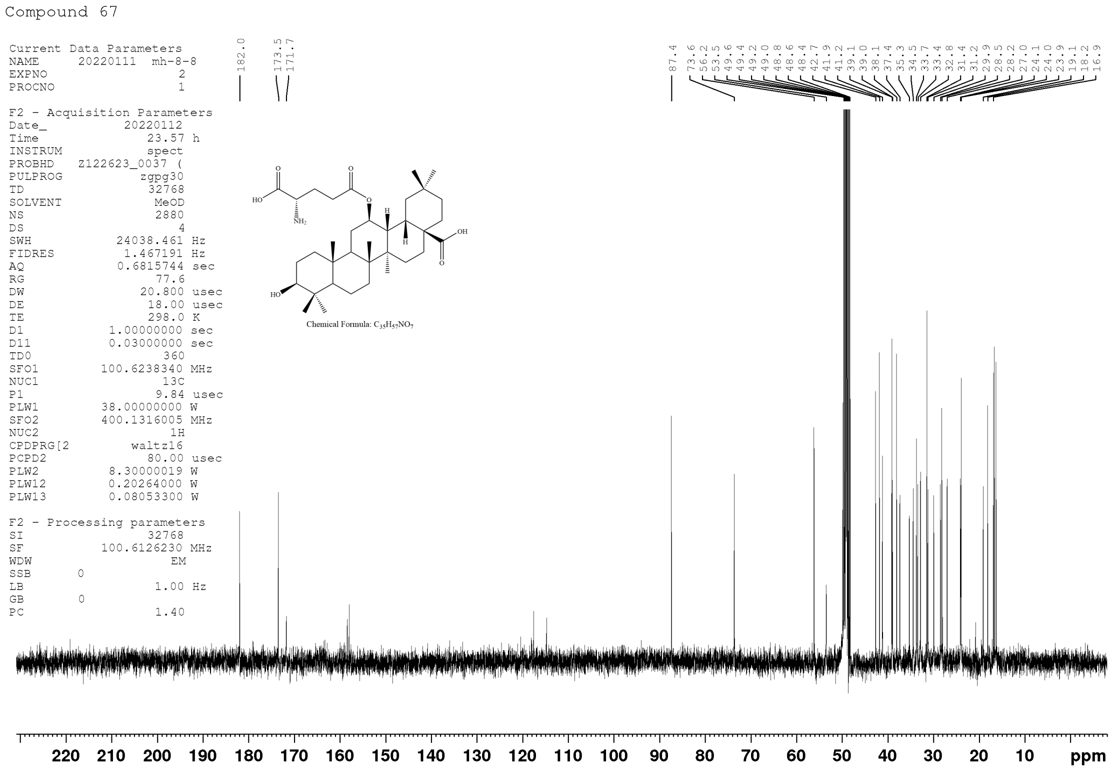

Fig. S46. ^{13}C NMR of compound **11c**

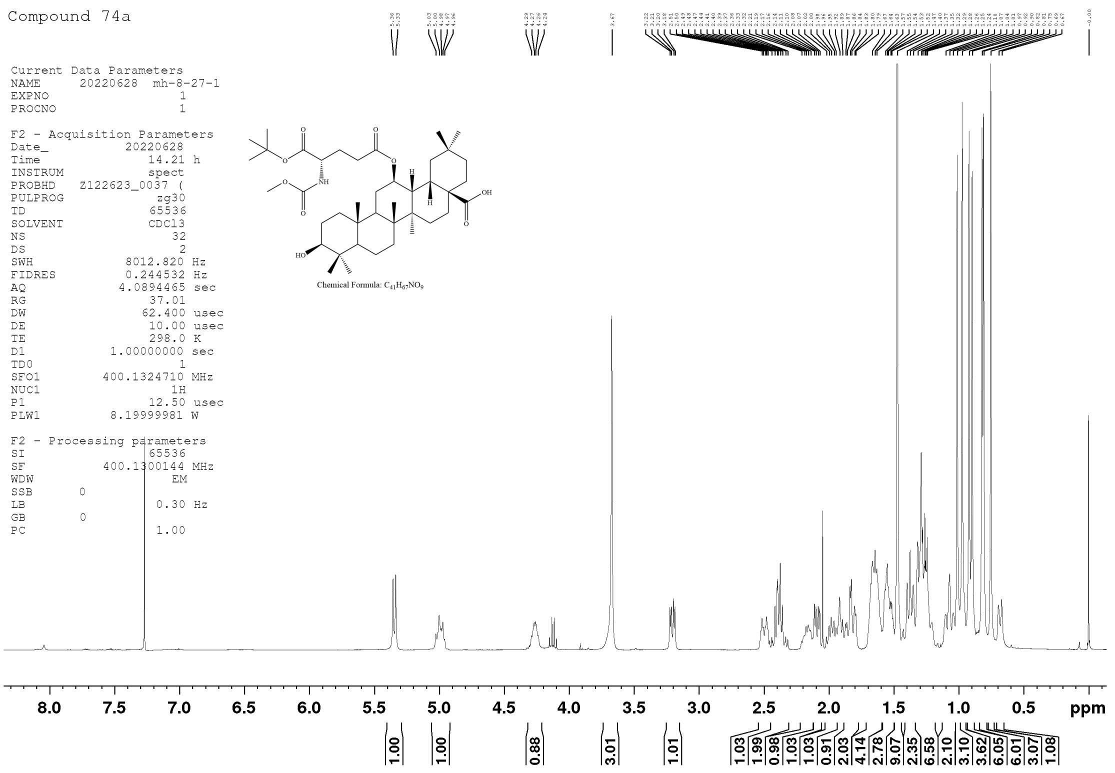

Fig. S47. ^{1}H NMR of compound **19a**

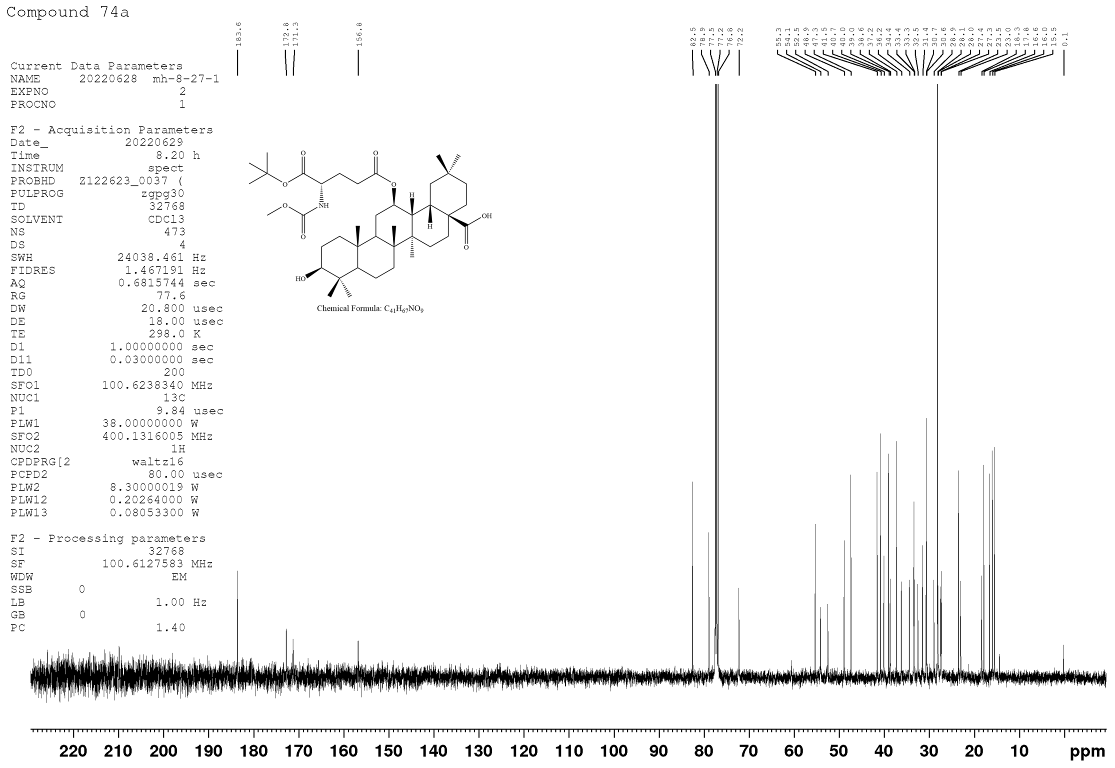

Fig. S48. ^{13}C NMR of compound **19a**

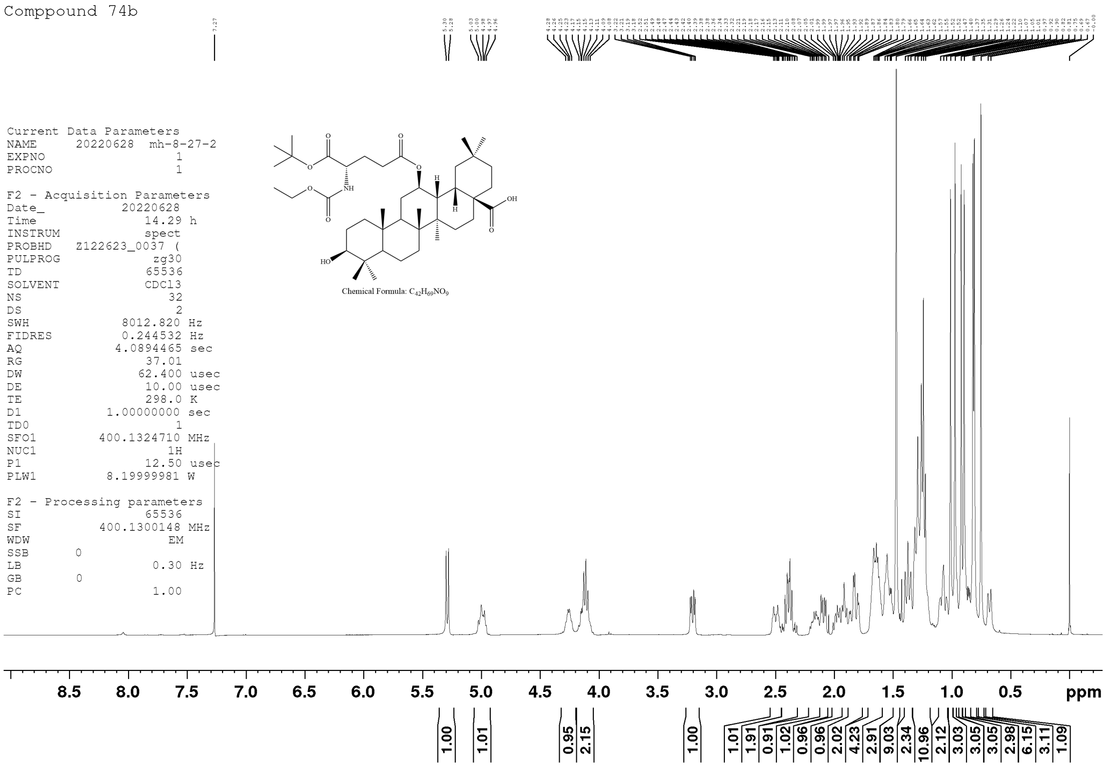

Fig. S49. ^{1}H NMR of compound **19b**

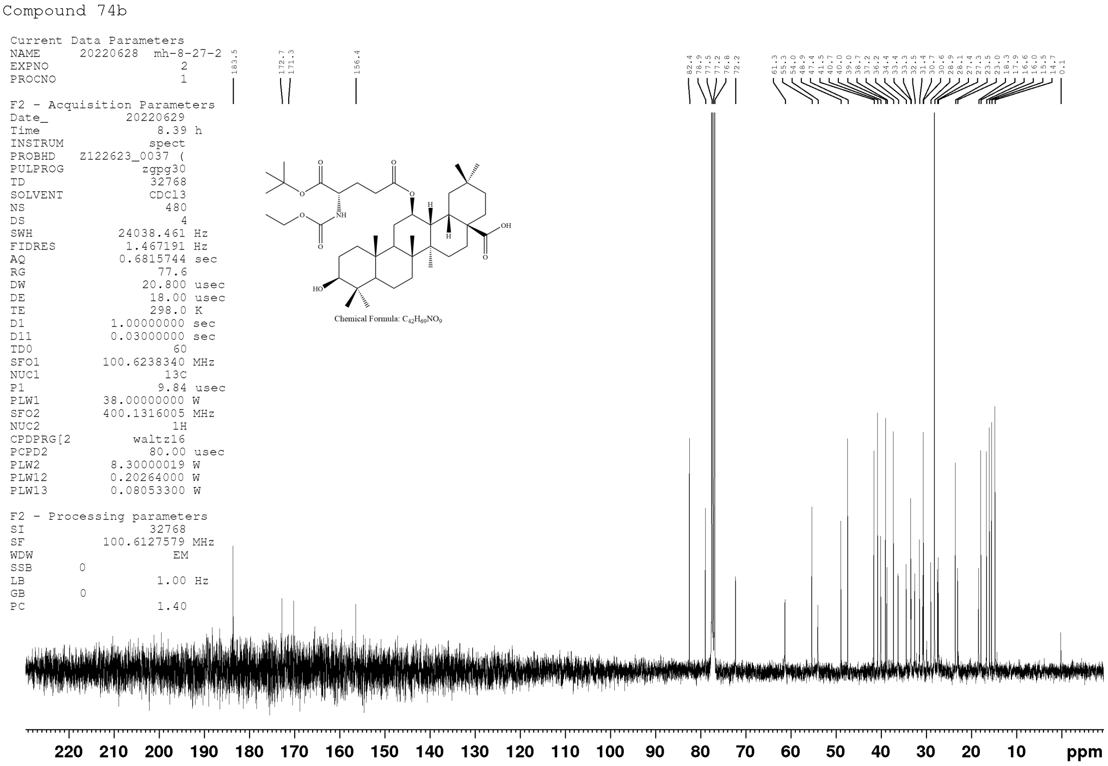

Fig. S50. ^{13}C NMR of compound **19b**

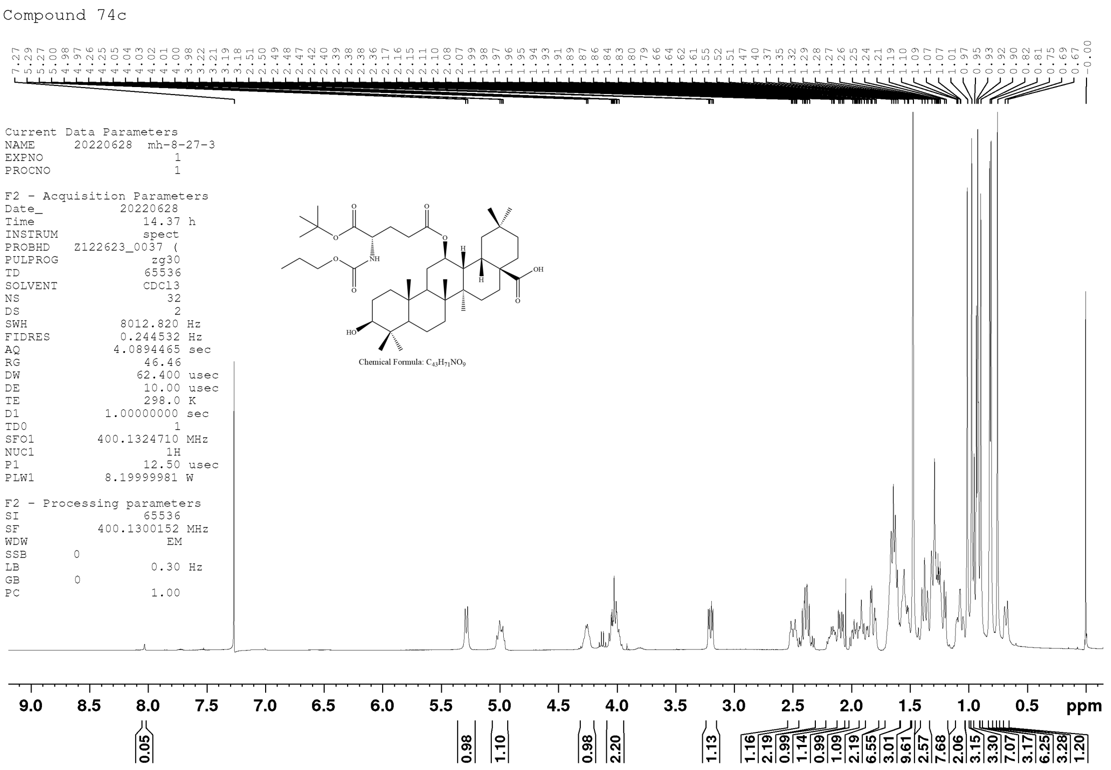

Fig. S51. ^{1}H NMR of compound **19c**

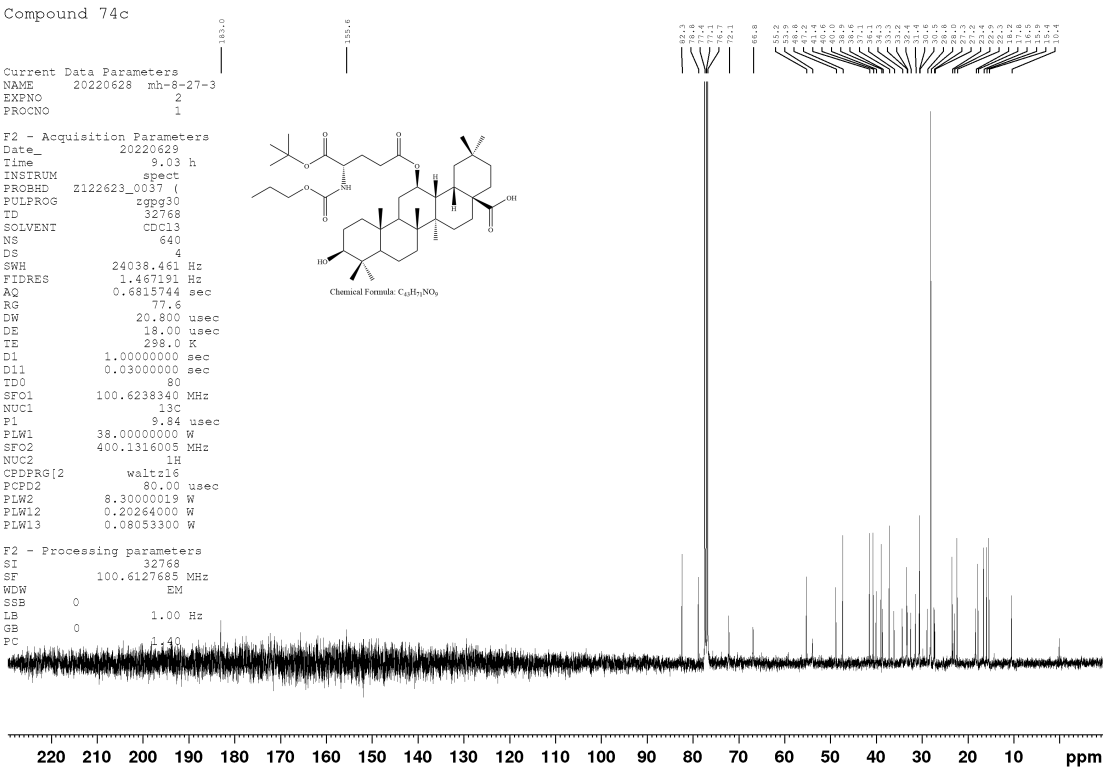

Fig. S52. ^{13}C NMR of compound **19c**

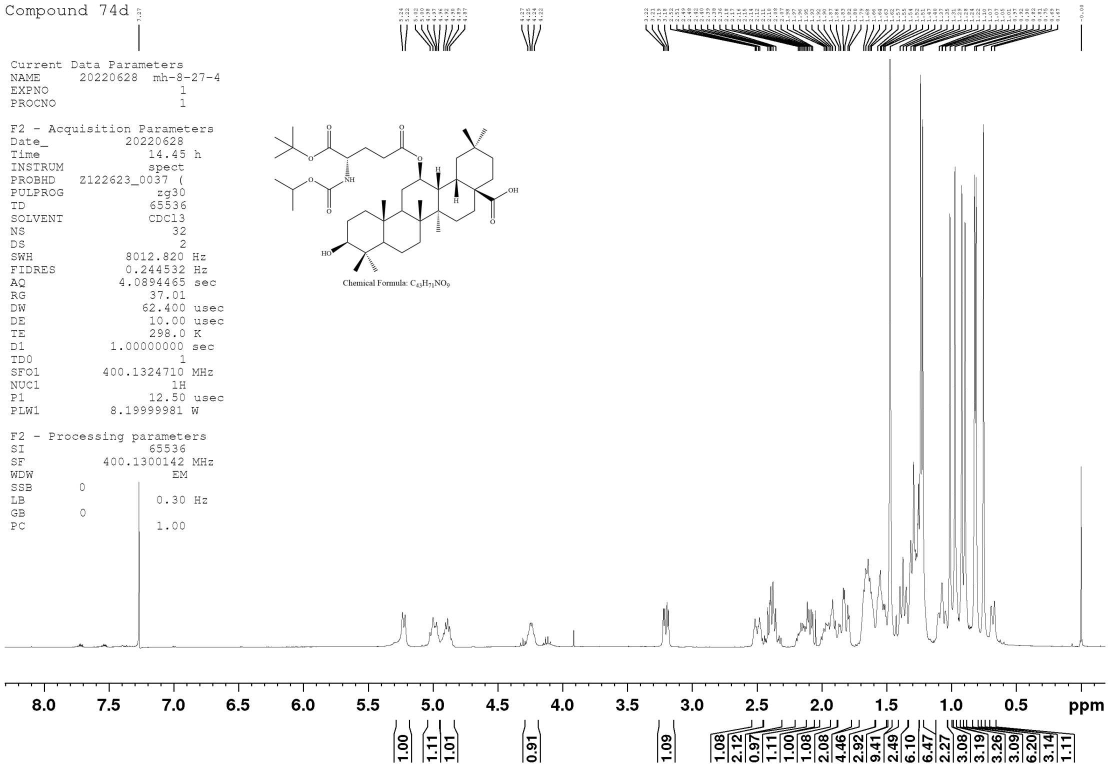

Fig. S53. ^{1}H NMR of compound **19d**

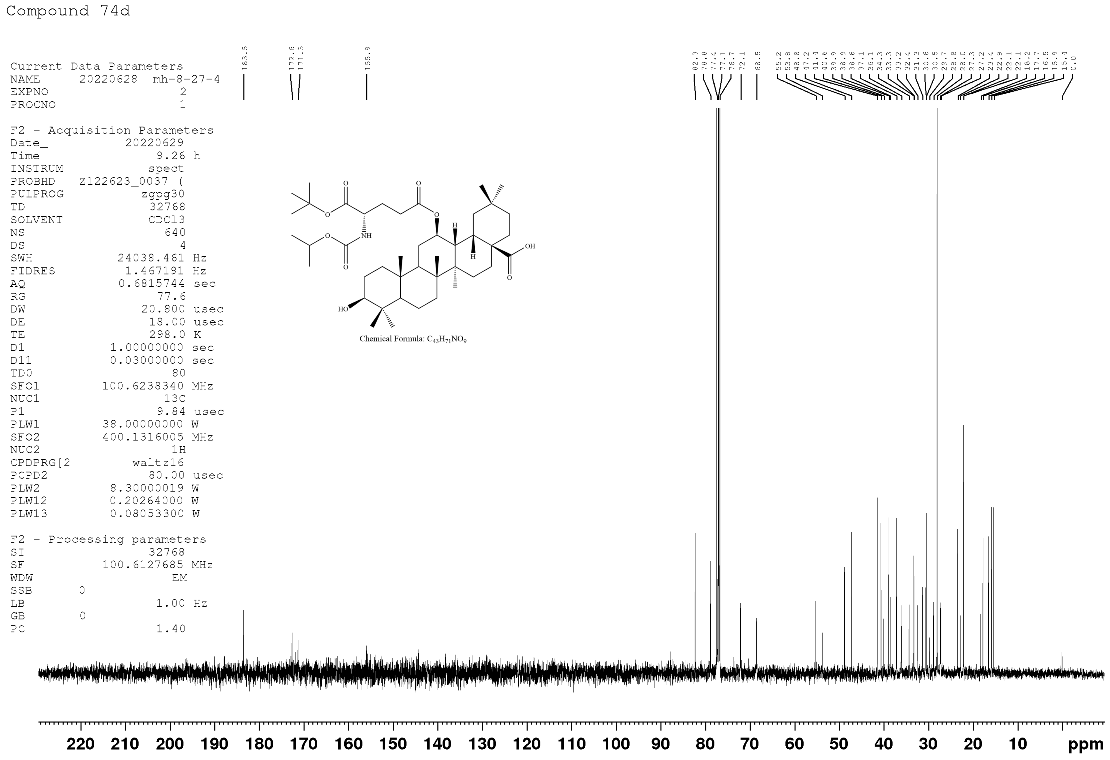

Fig. S54. ^{13}C NMR of compound **19d**

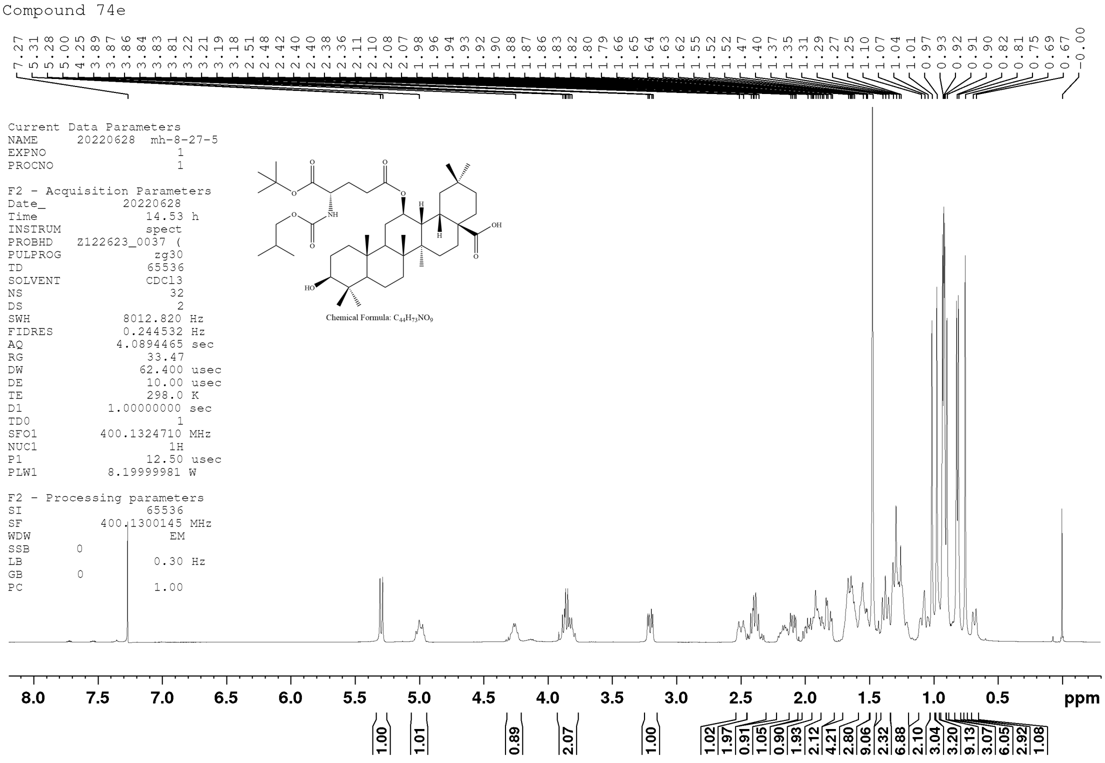

Fig. S55. ^{1}H NMR of compound **19e**

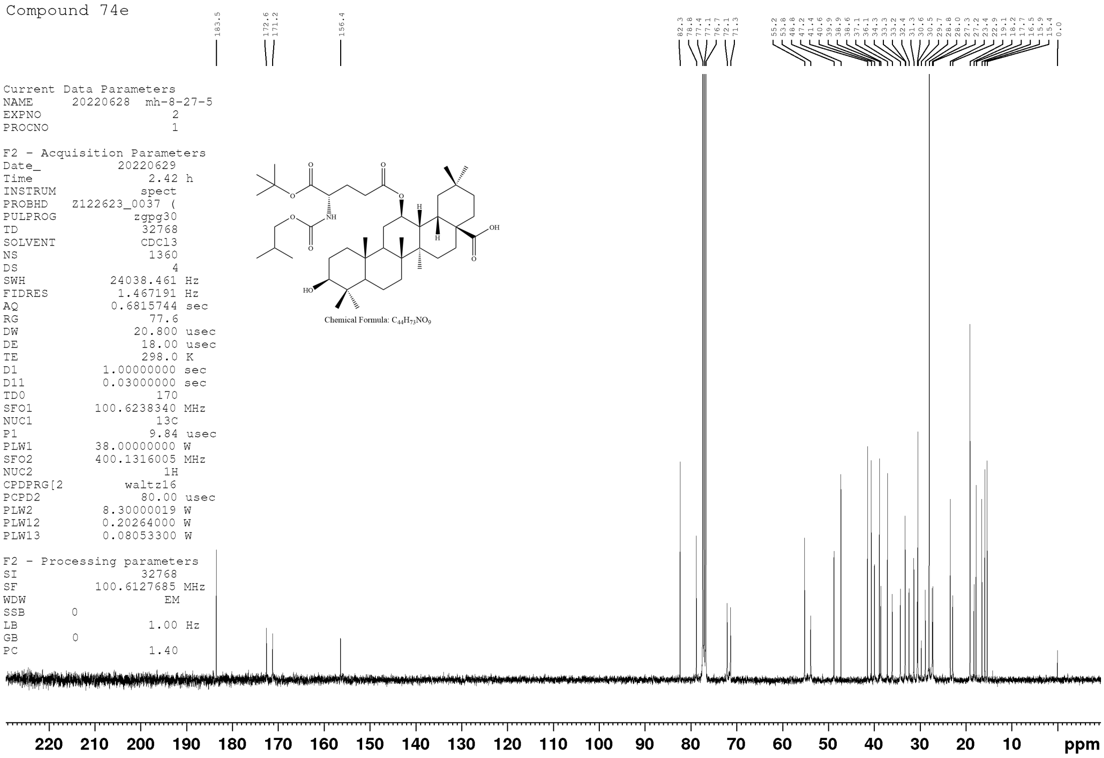

Fig. S56. ^{13}C NMR of compound **19e**

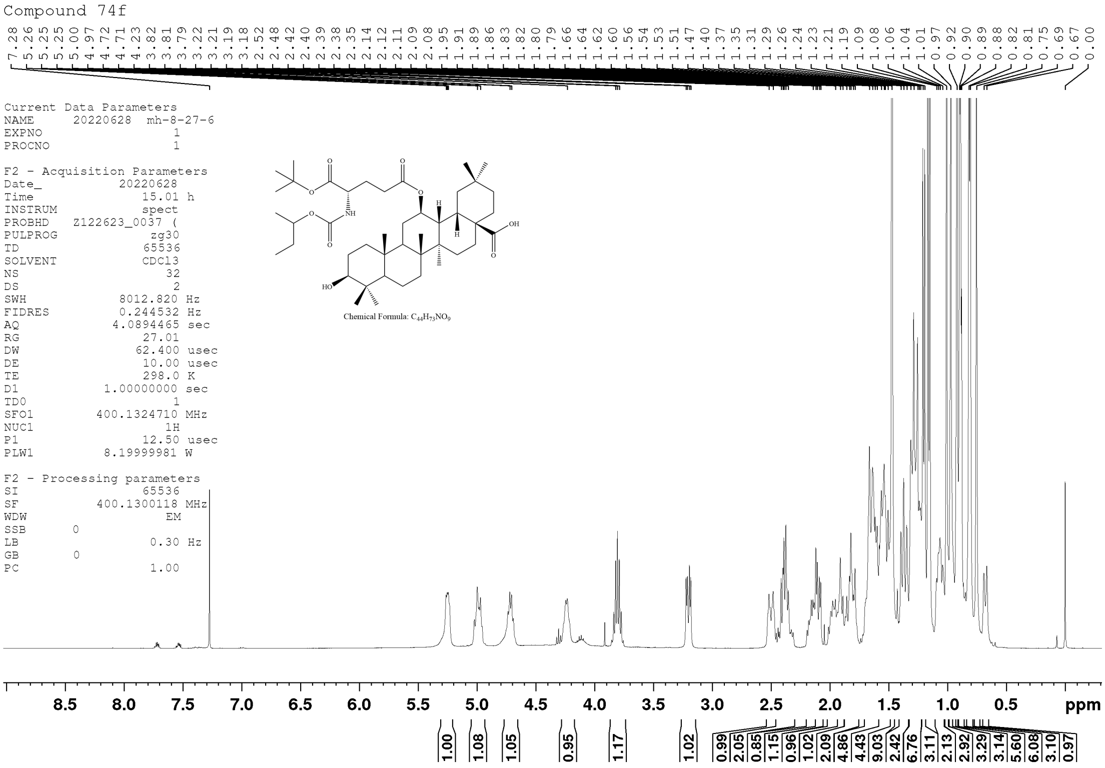

Fig. S57. ^{1}H NMR of compound **19f**

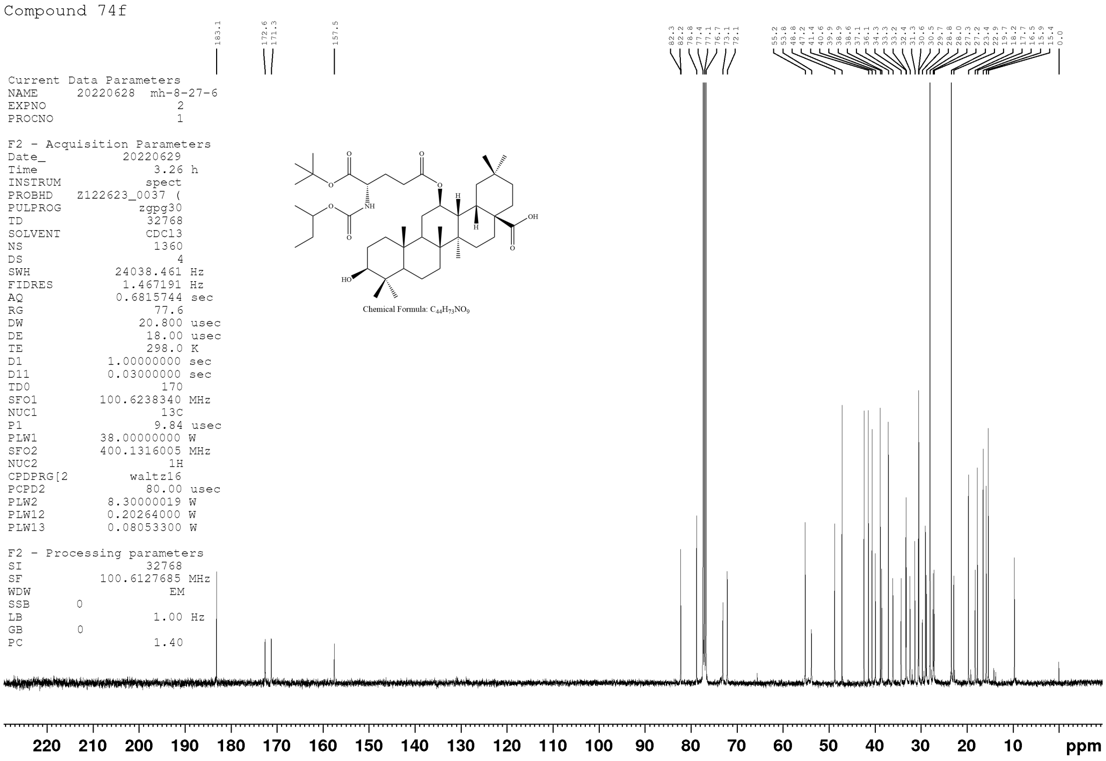

Fig. S58. ^{13}C NMR of compound **19f**

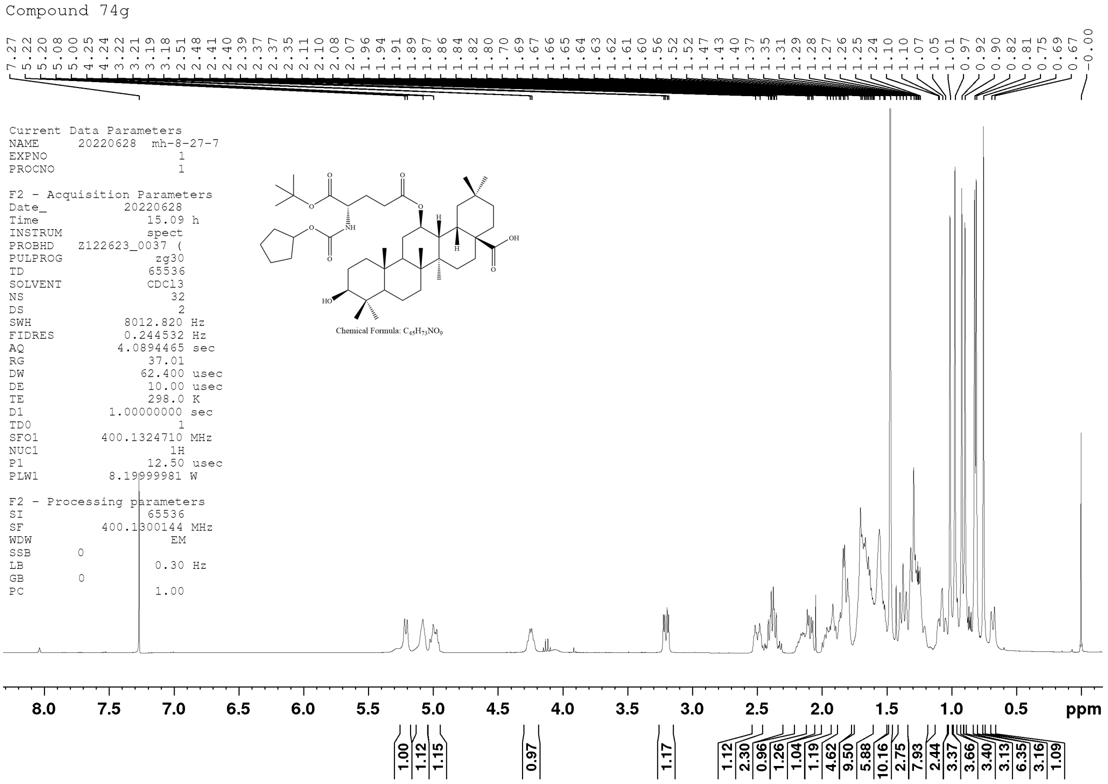

Fig. S59. ^{1}H NMR of compound **19g**

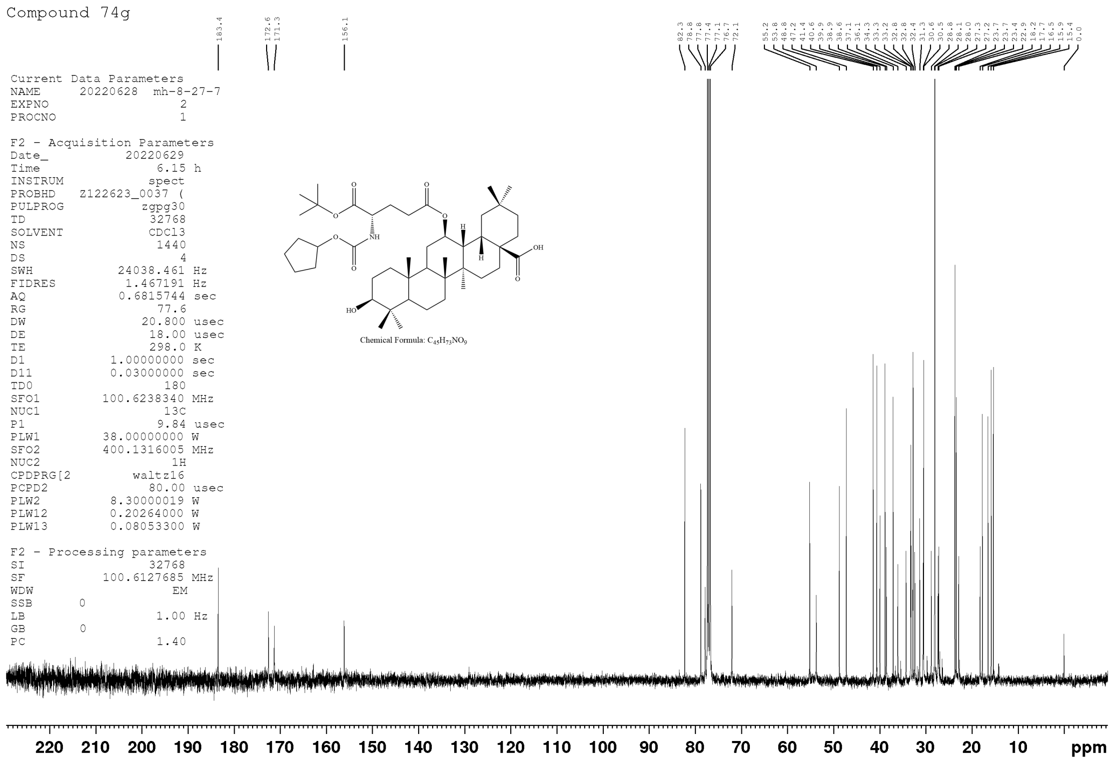

Fig. S60. ^{13}C NMR of compound **19g**

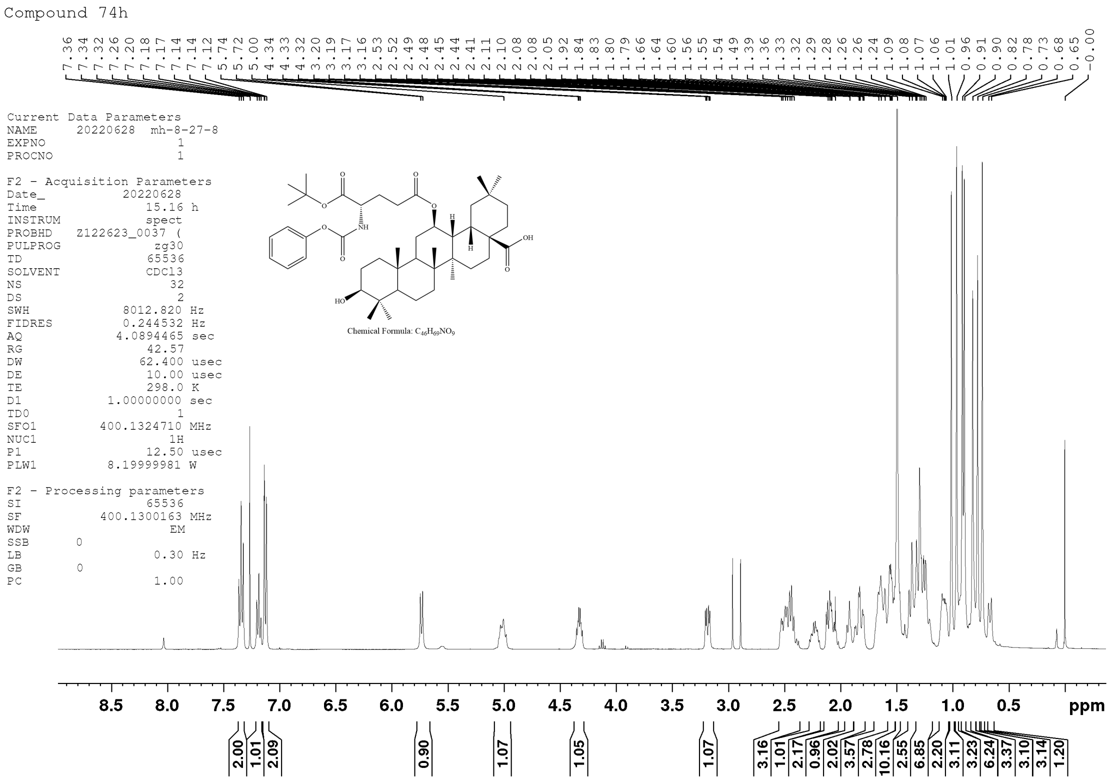

Fig. S61. ^{1}H NMR of compound **19h**

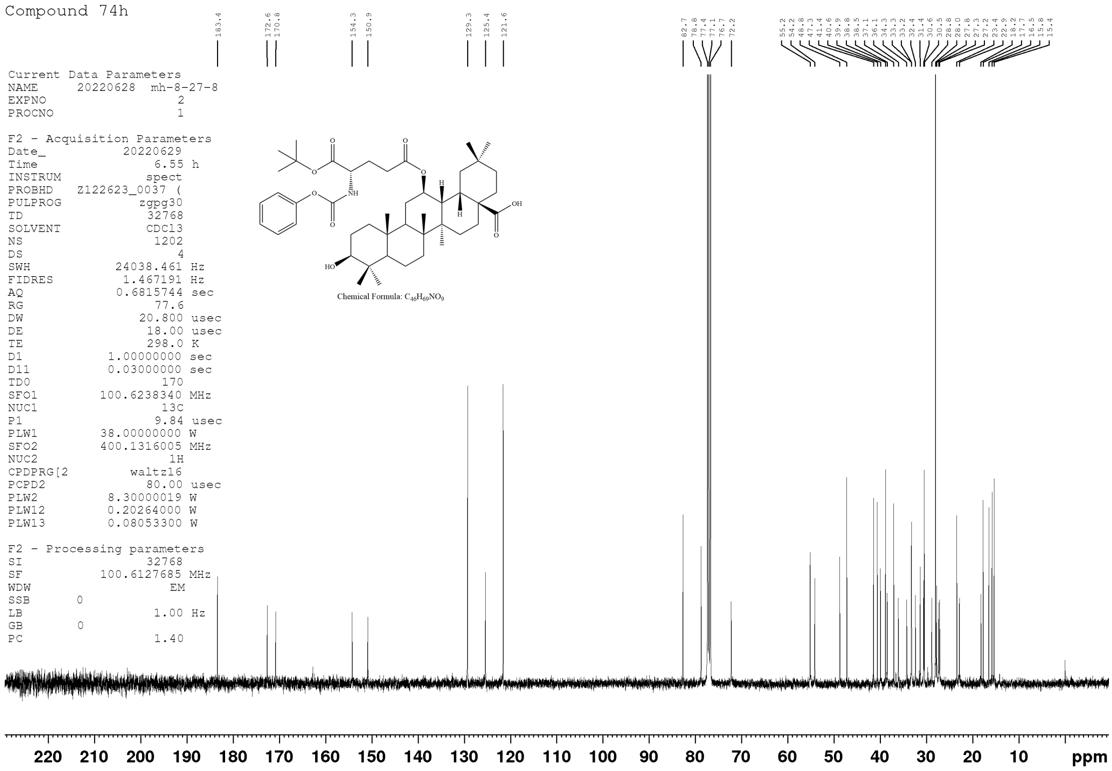

Fig. S62. ^{13}C NMR of compound **19h**

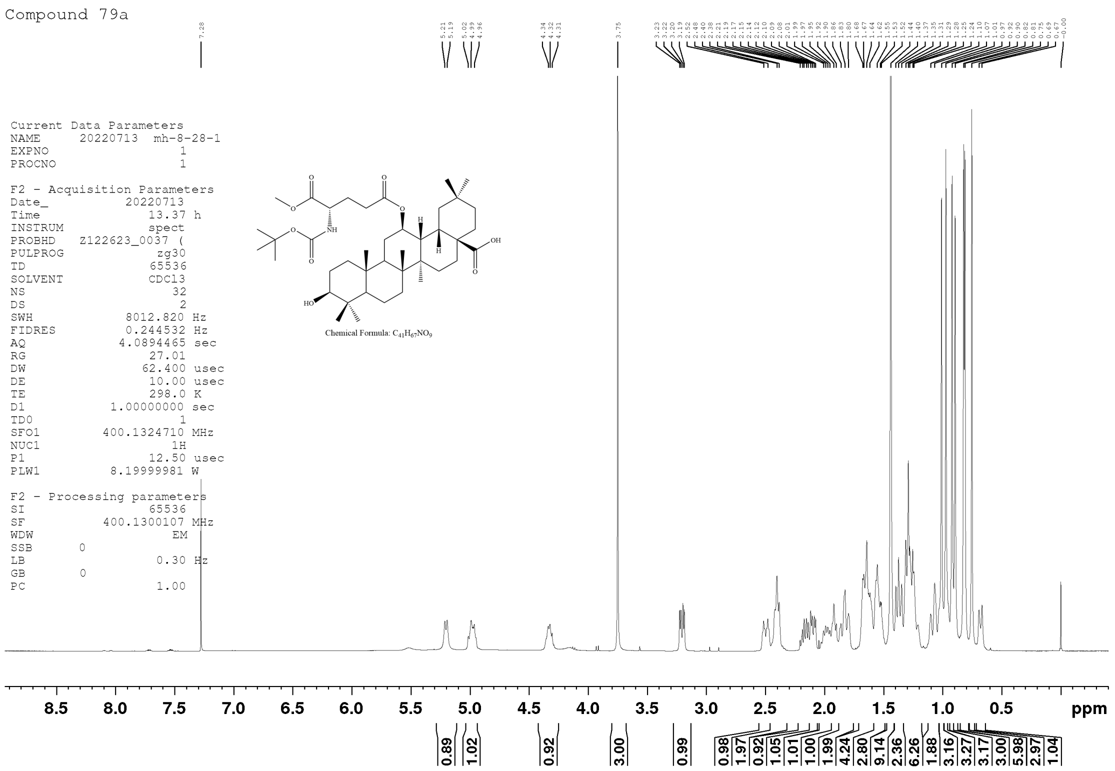

Fig. S63. ^{1}H NMR of compound **20a**

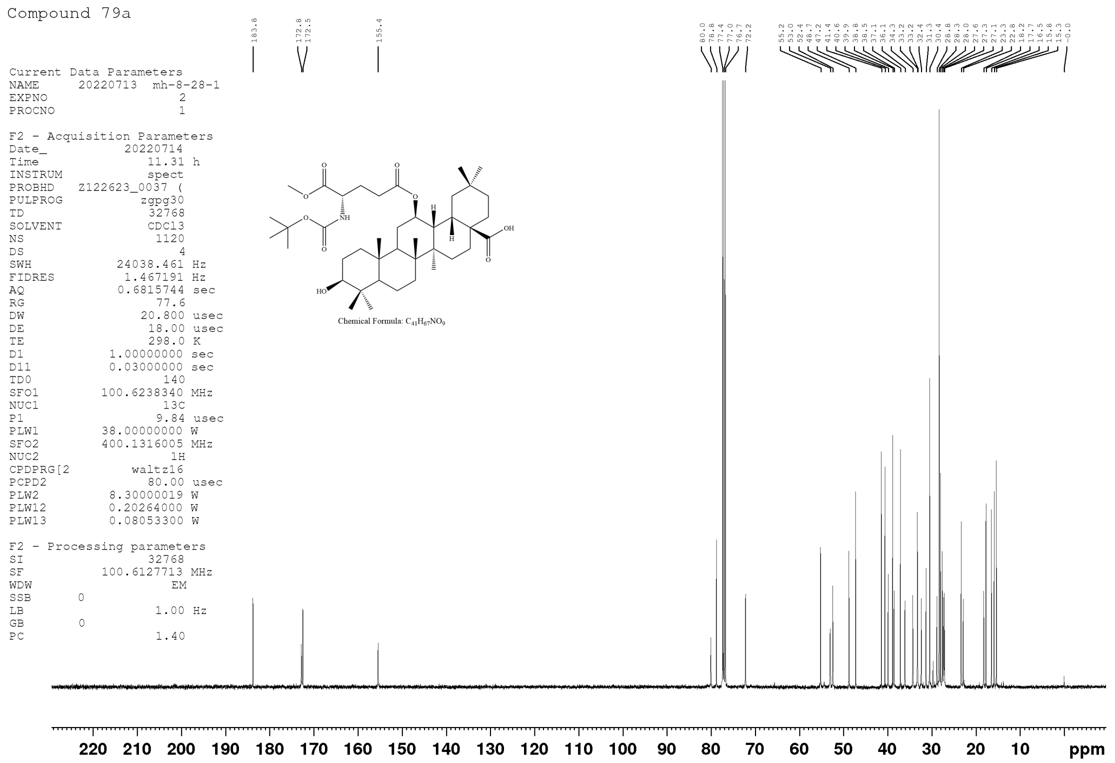

Fig. S64. ^{13}C NMR of compound **20a**

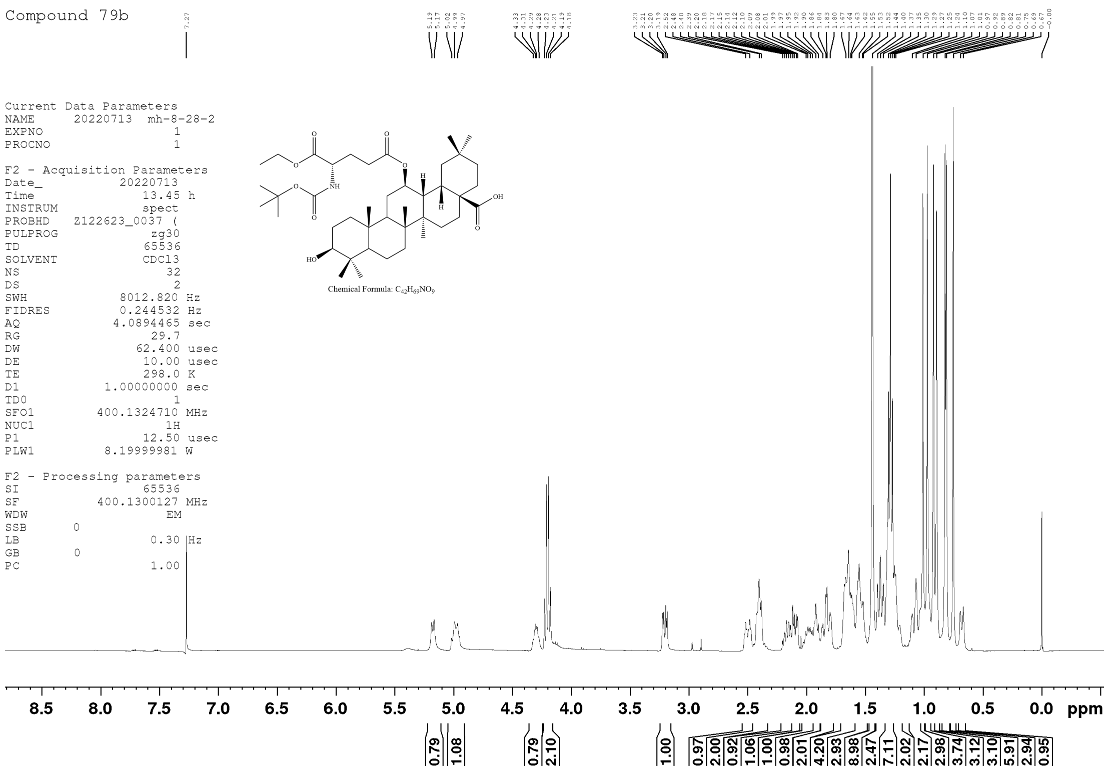

Fig. S65. ^{1}H NMR of compound **20b**

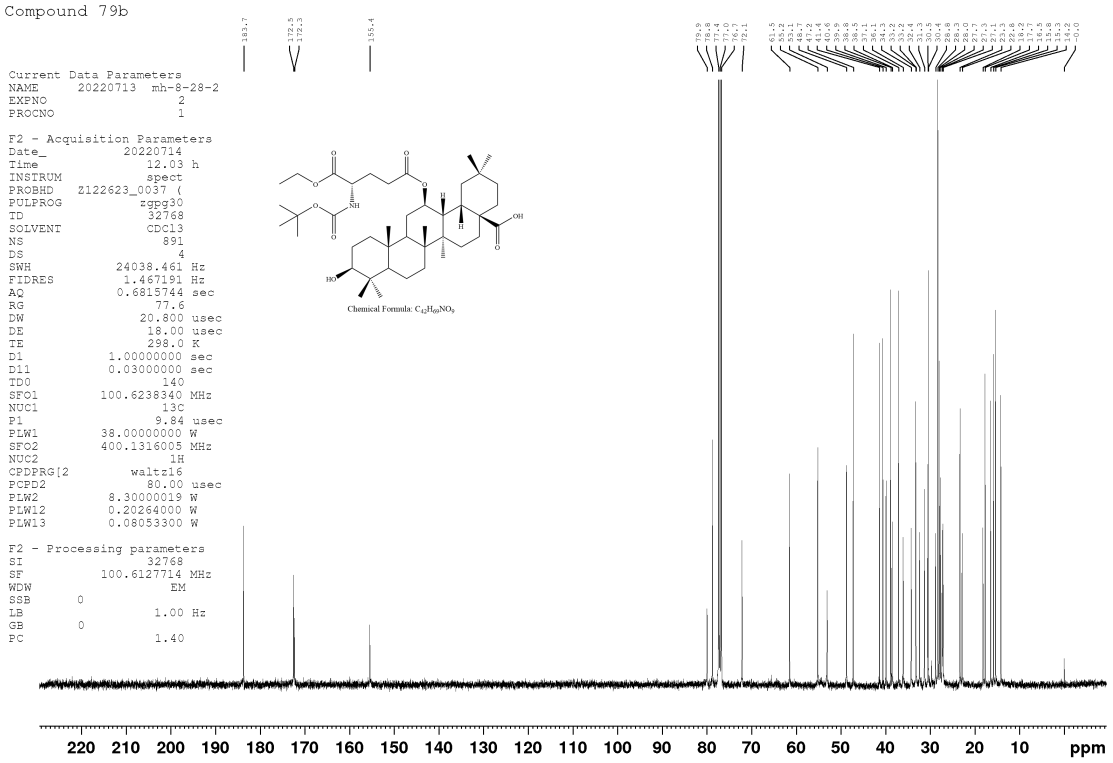

Fig. S66. ^{13}C NMR of compound **20b**

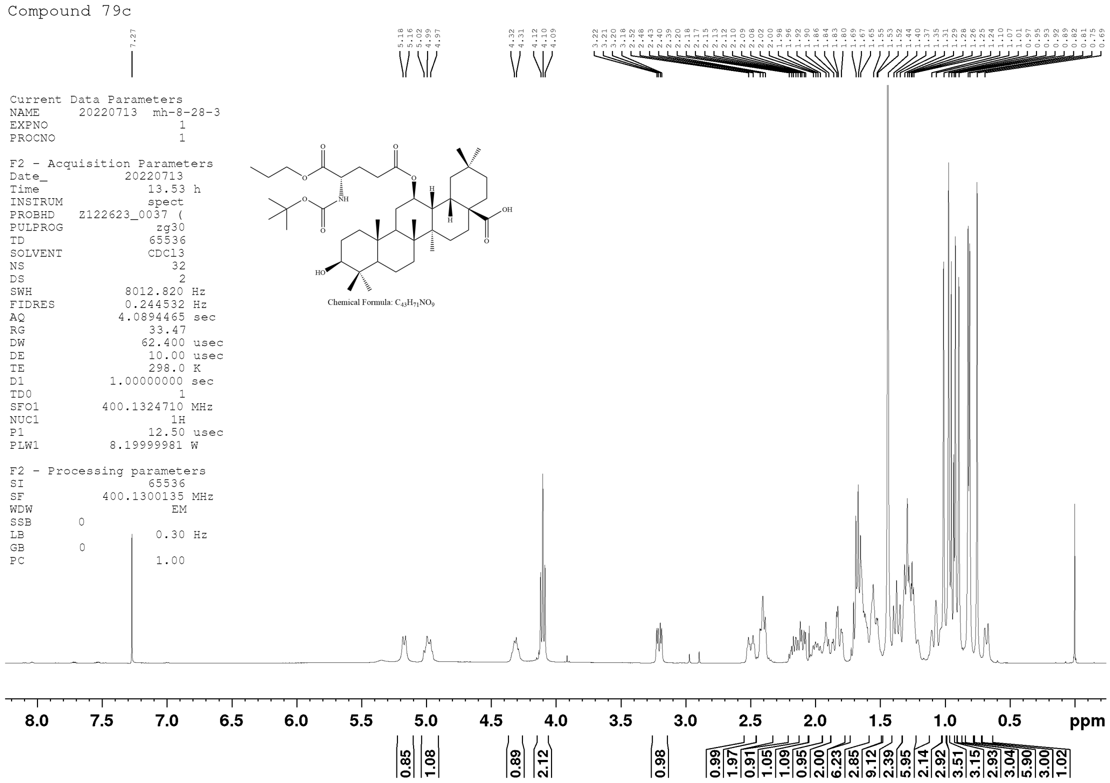

Fig. S67. ^{1}H NMR of compound **20c**

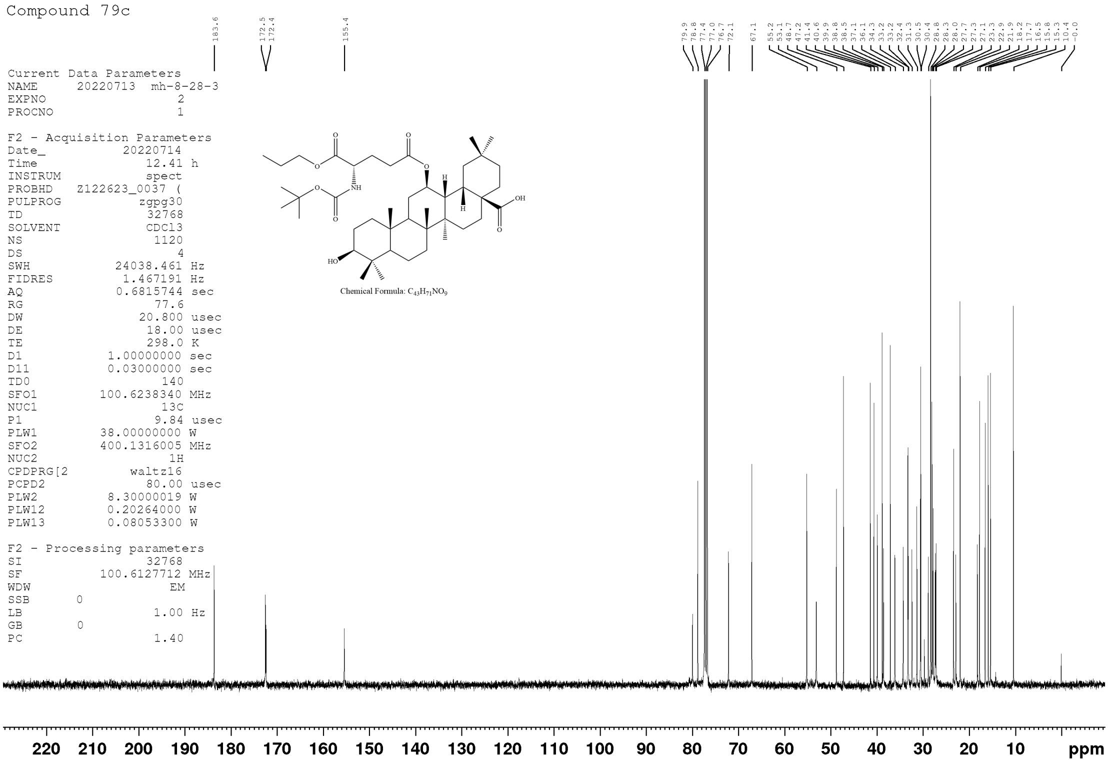

Fig. S68. ^{13}C NMR of compound **20c**

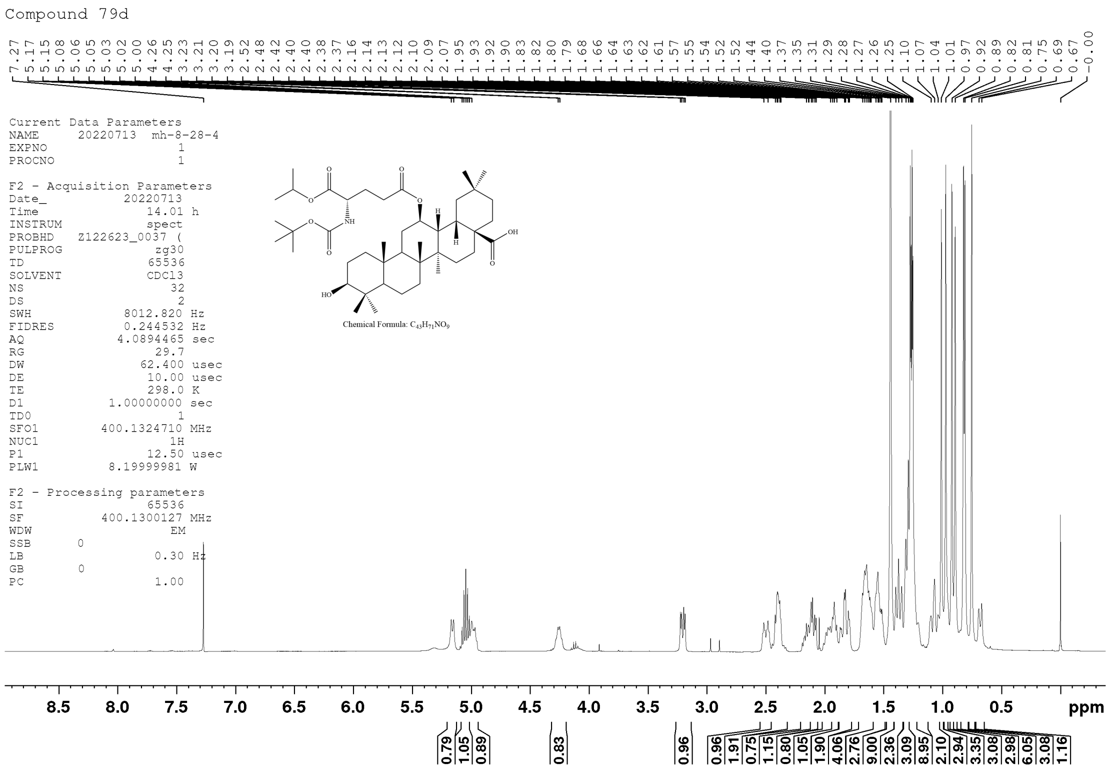

Fig. S69. ^{1}H NMR of compound **20d**

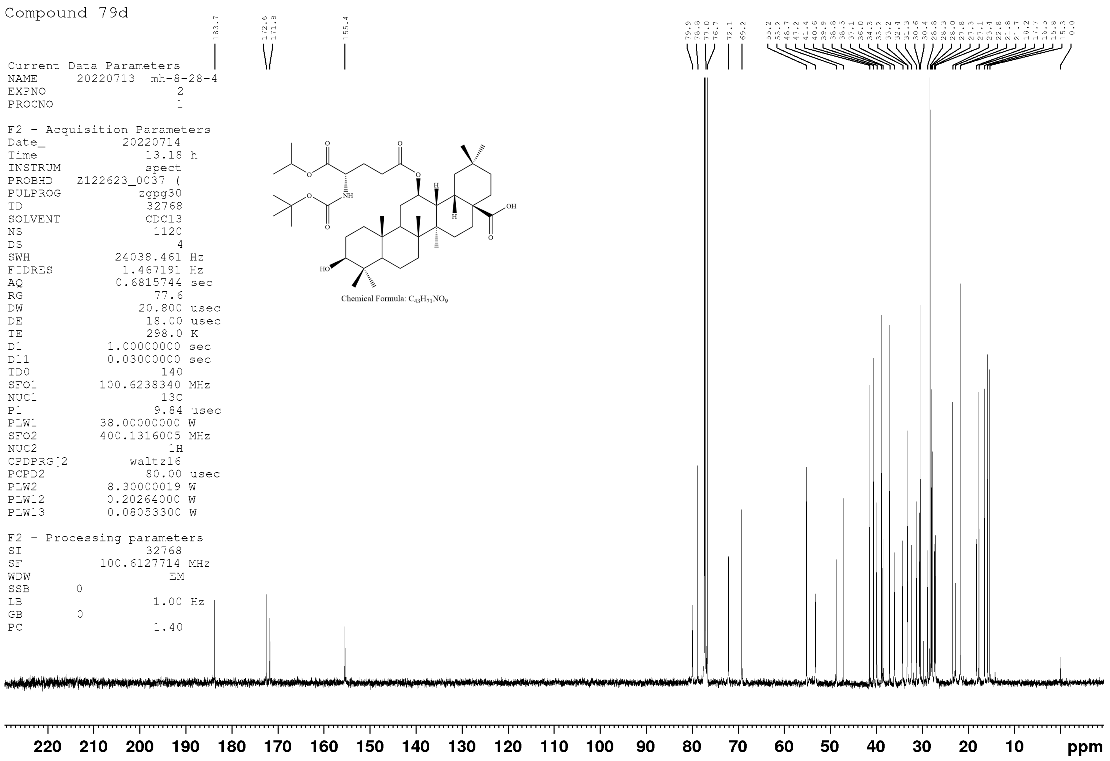

Fig. S70. ^{13}C NMR of compound **20d**

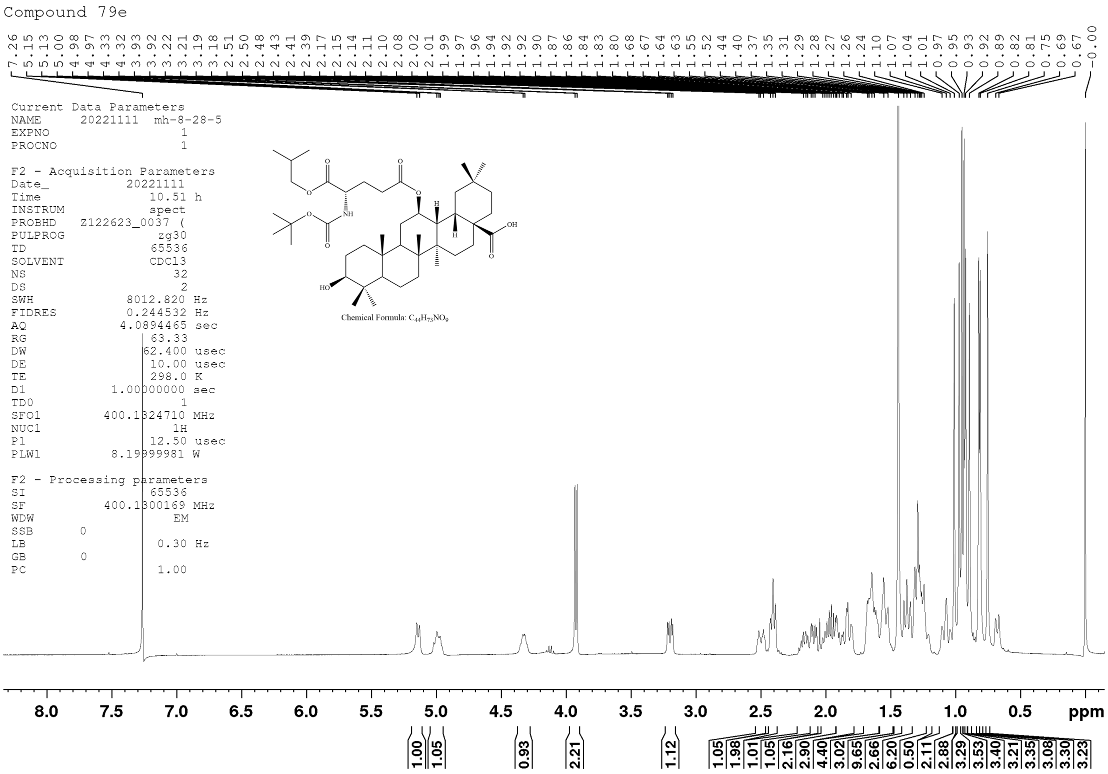

Fig. S71. ^{1}H NMR of compound **20e**

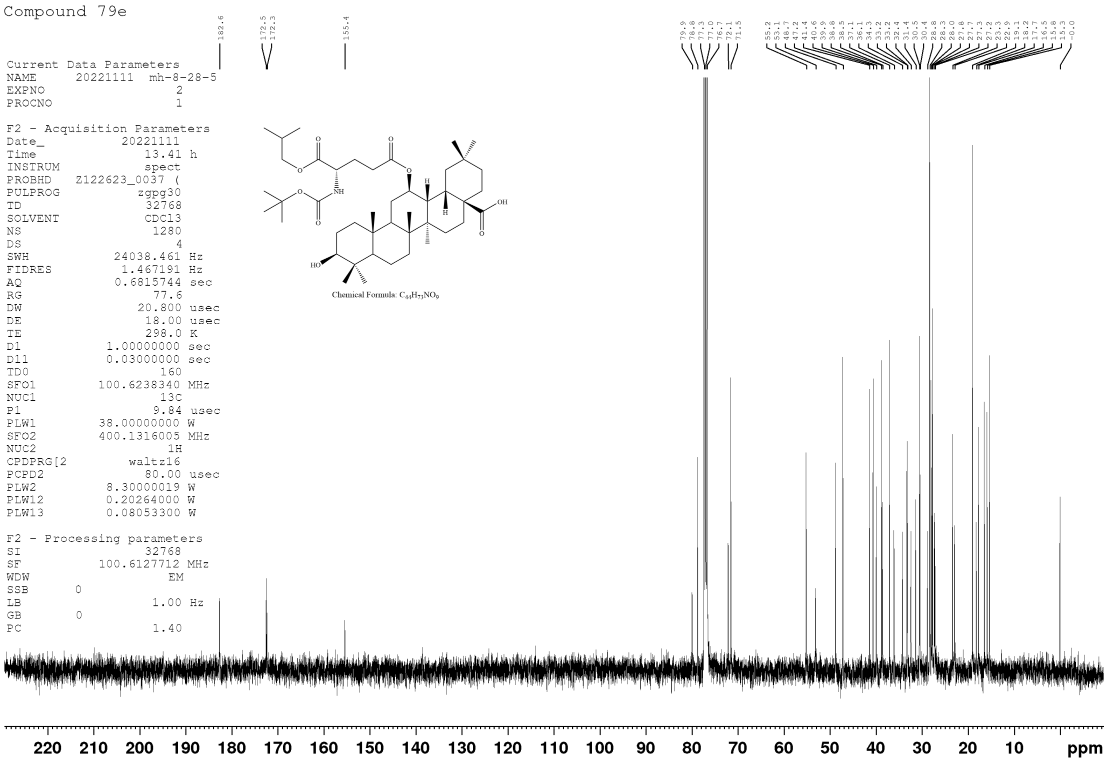

Fig. S72. ^{13}C NMR of compound **20e**

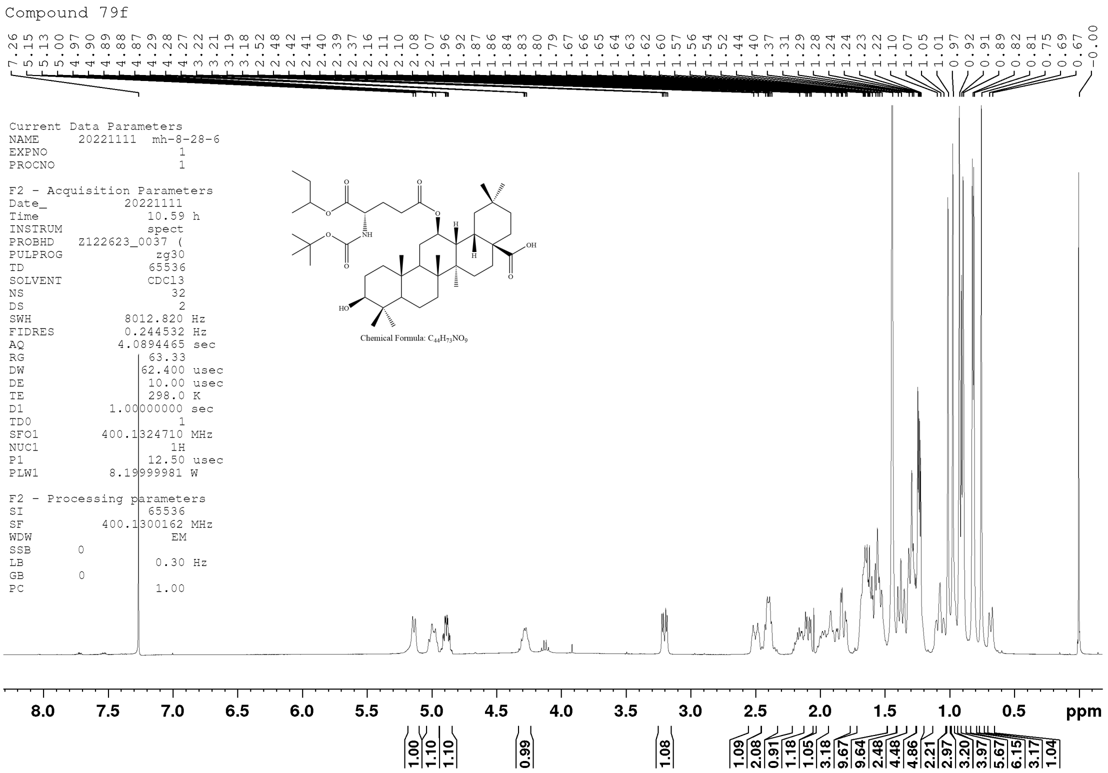

Fig. S73. ^{1}H NMR of compound **20f**

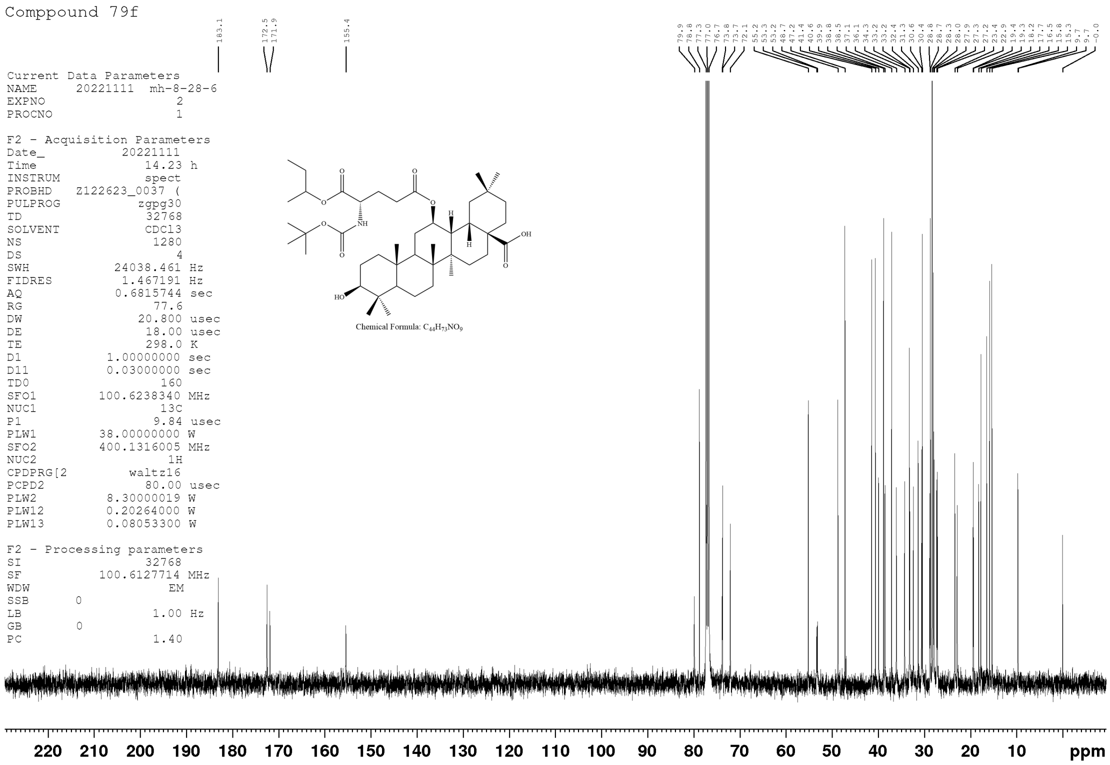

Fig. S74. ^{13}C NMR of compound **20f**

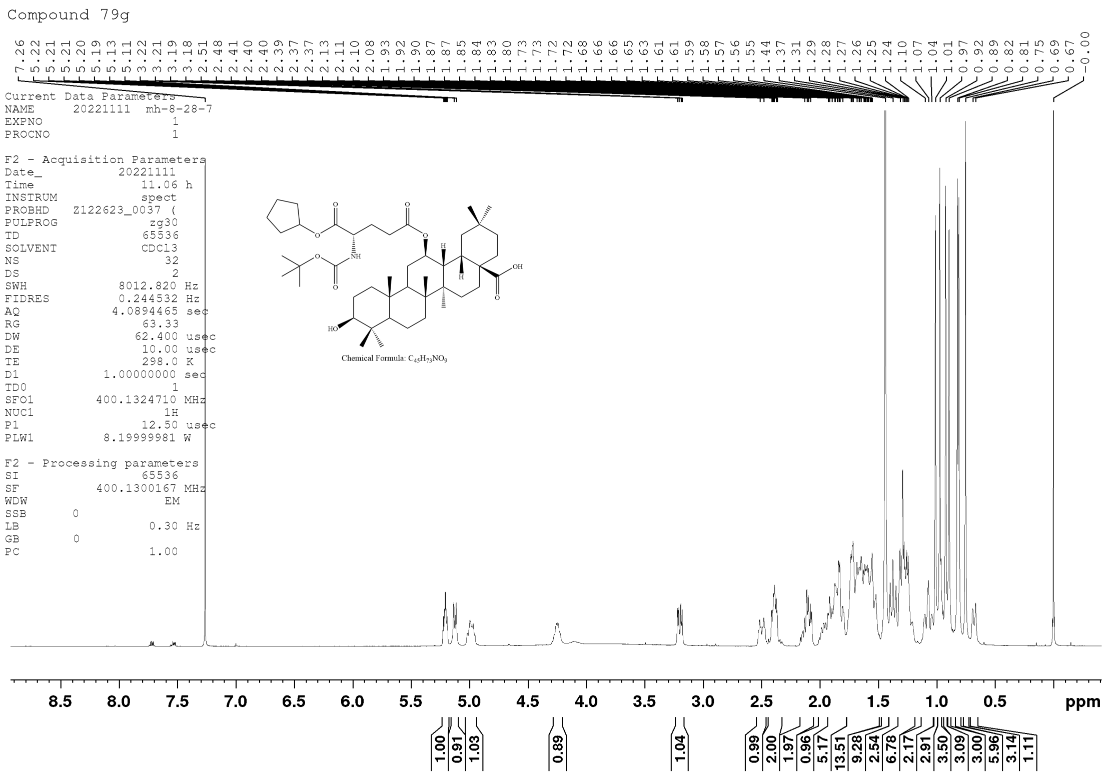

Fig. S75. ^{1}H NMR of compound **20g**

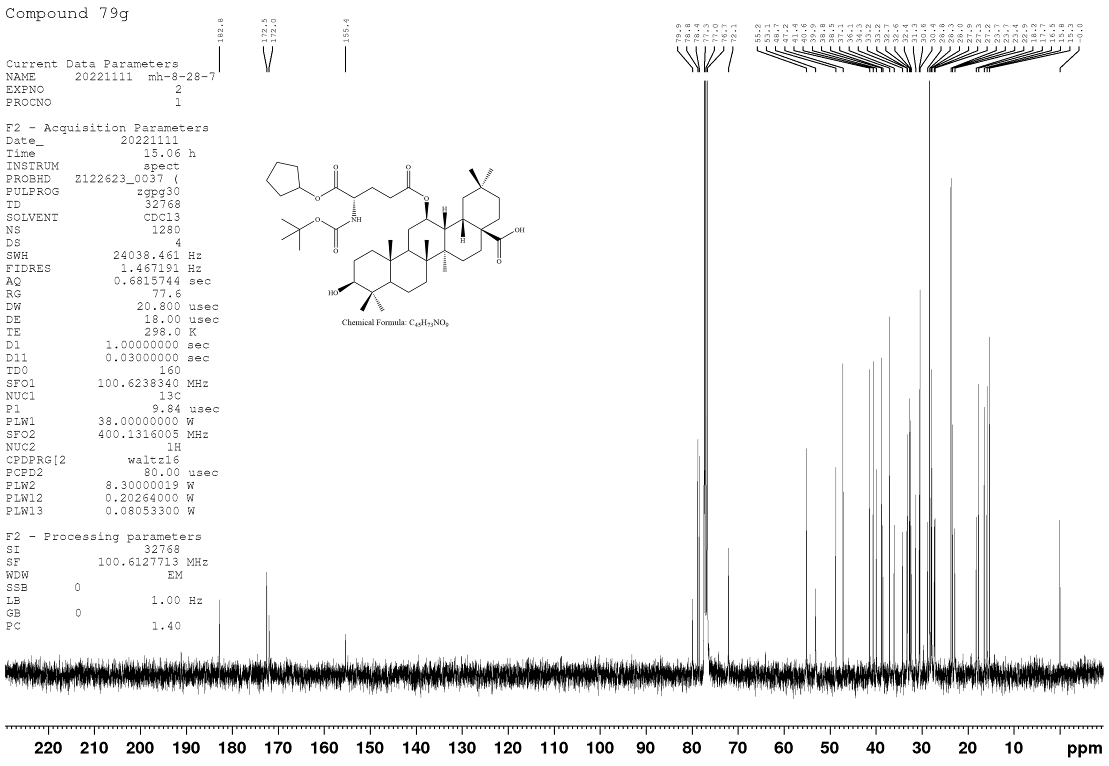

Fig. S76. ^{13}C NMR of compound **20g**

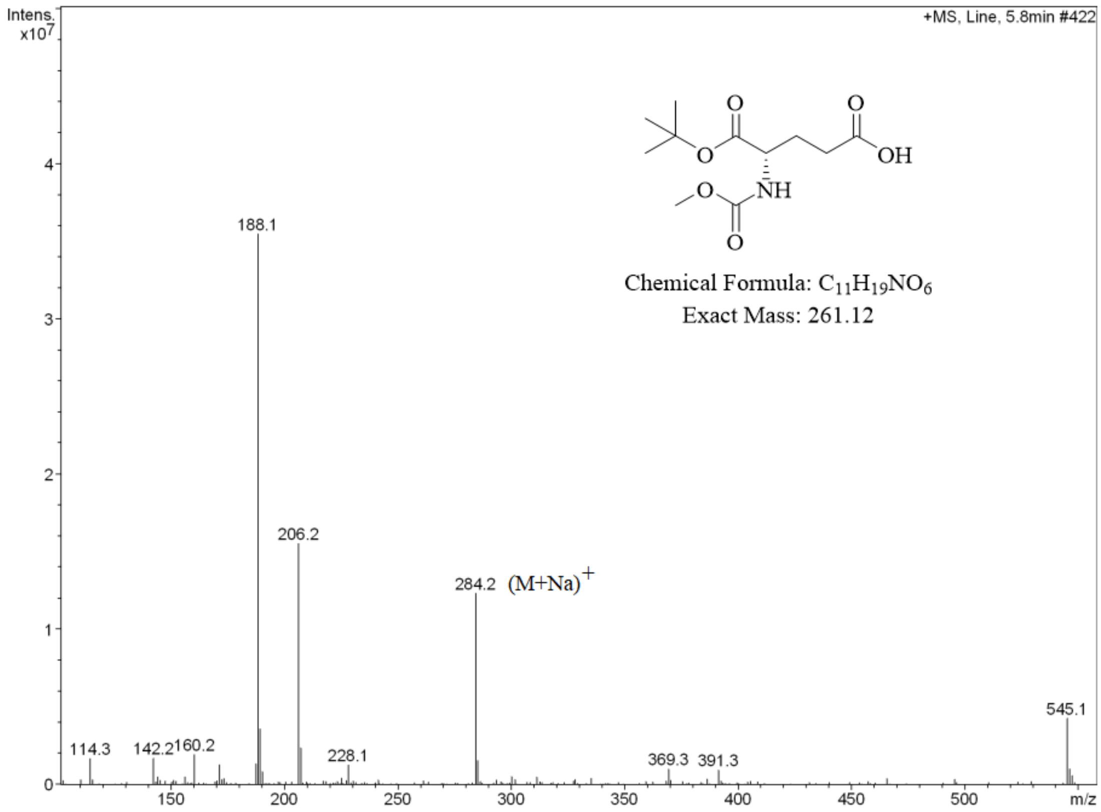

Fig. S77. MS of intermediate **14a**

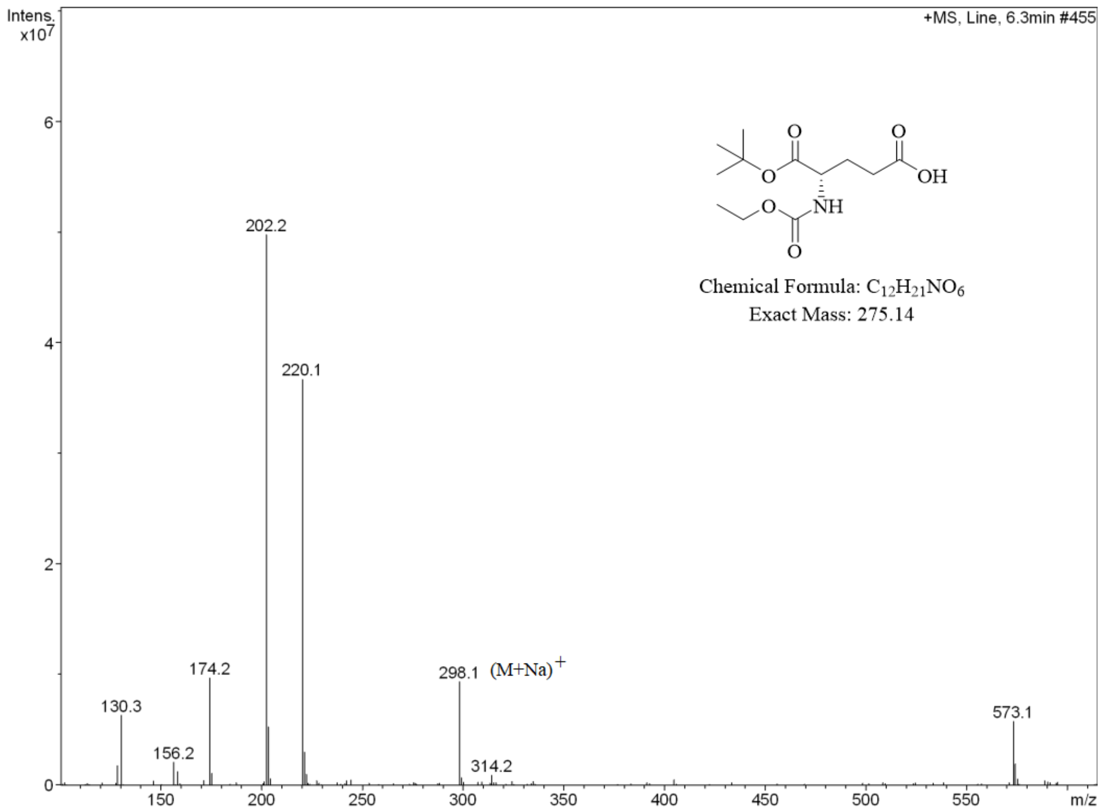

Fig. S78. MS of intermediate **14b**

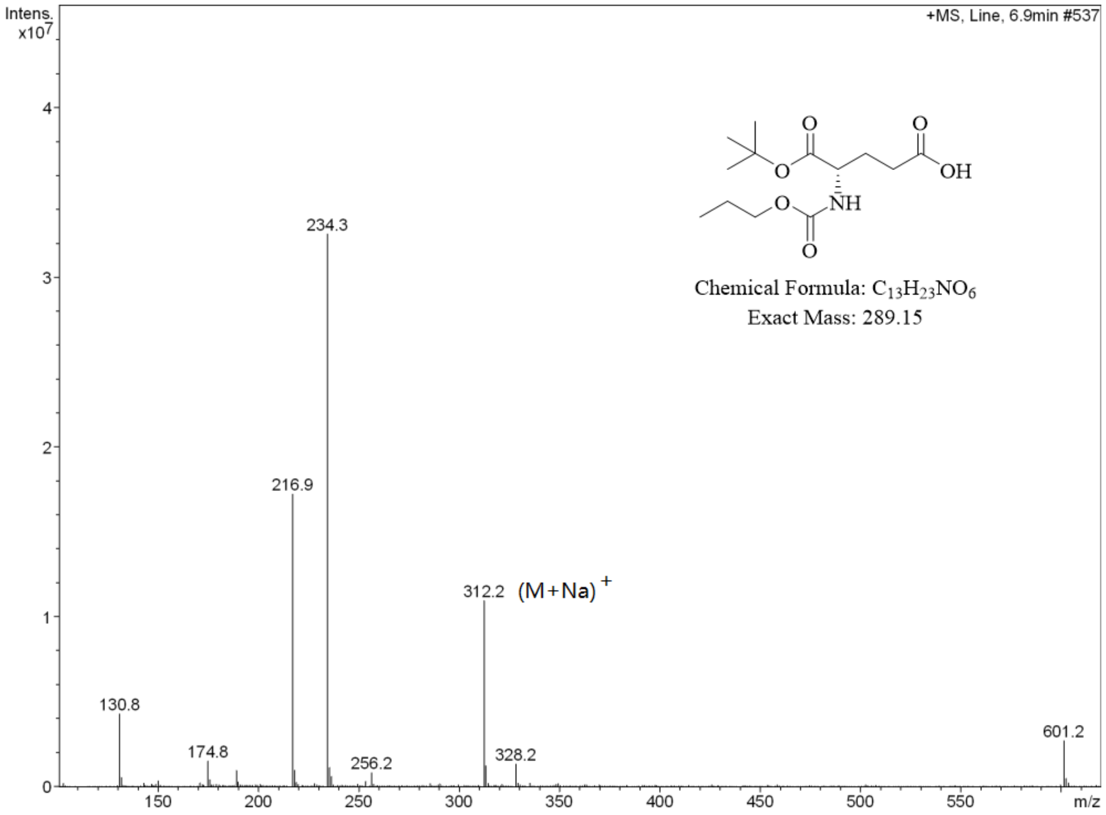

Fig. S79. MS of intermediate **14c**

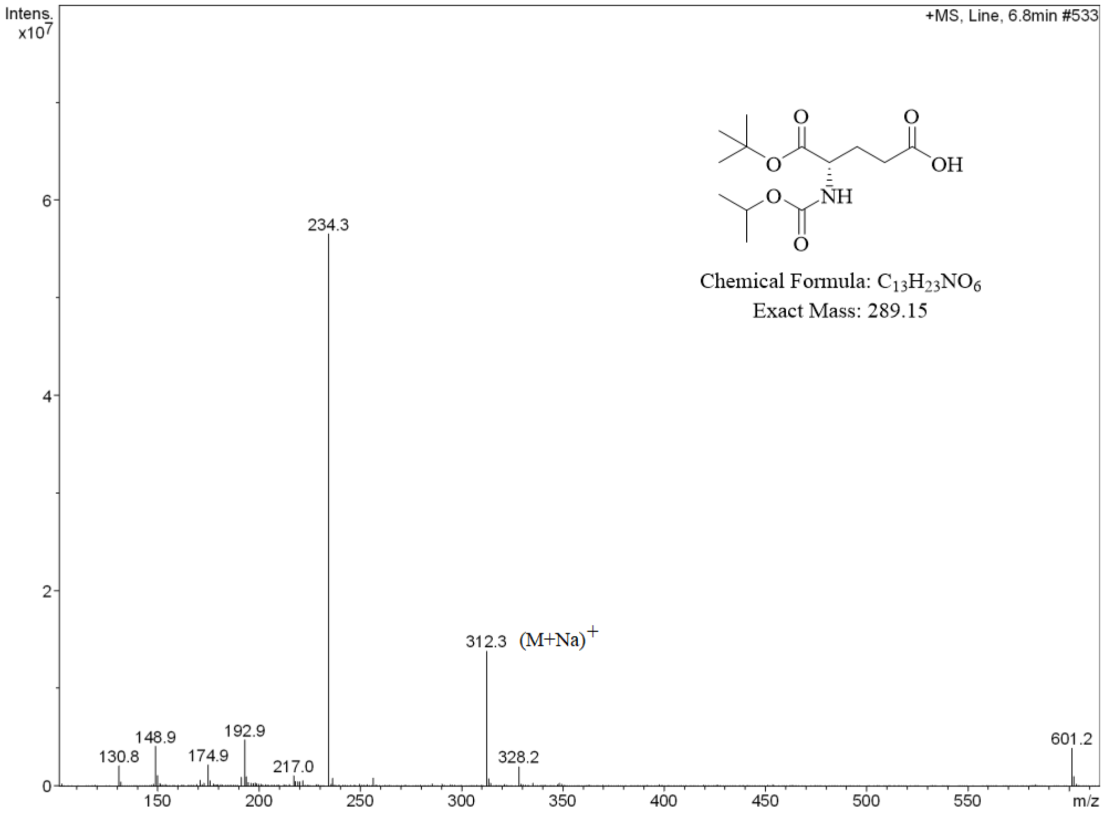

Fig. S80. MS of intermediate **14d**

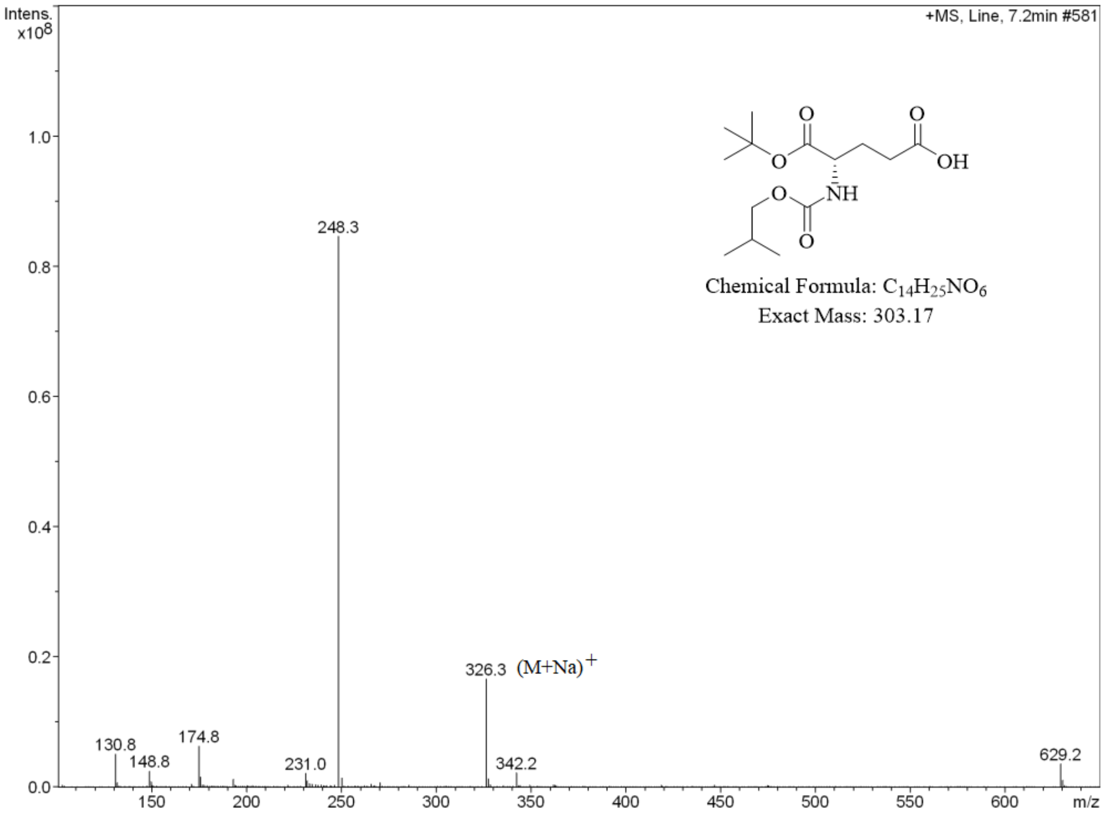

Fig. S81. MS of intermediate **14e**

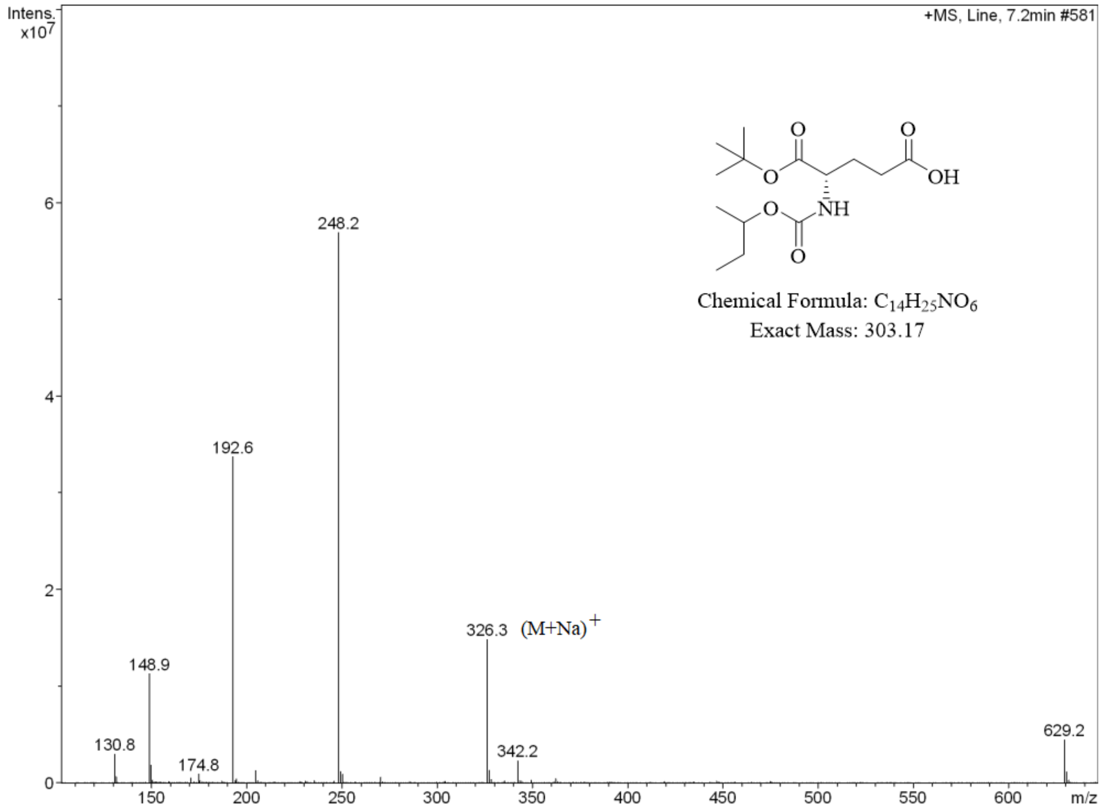

Fig. S82. MS of intermediate **14f**

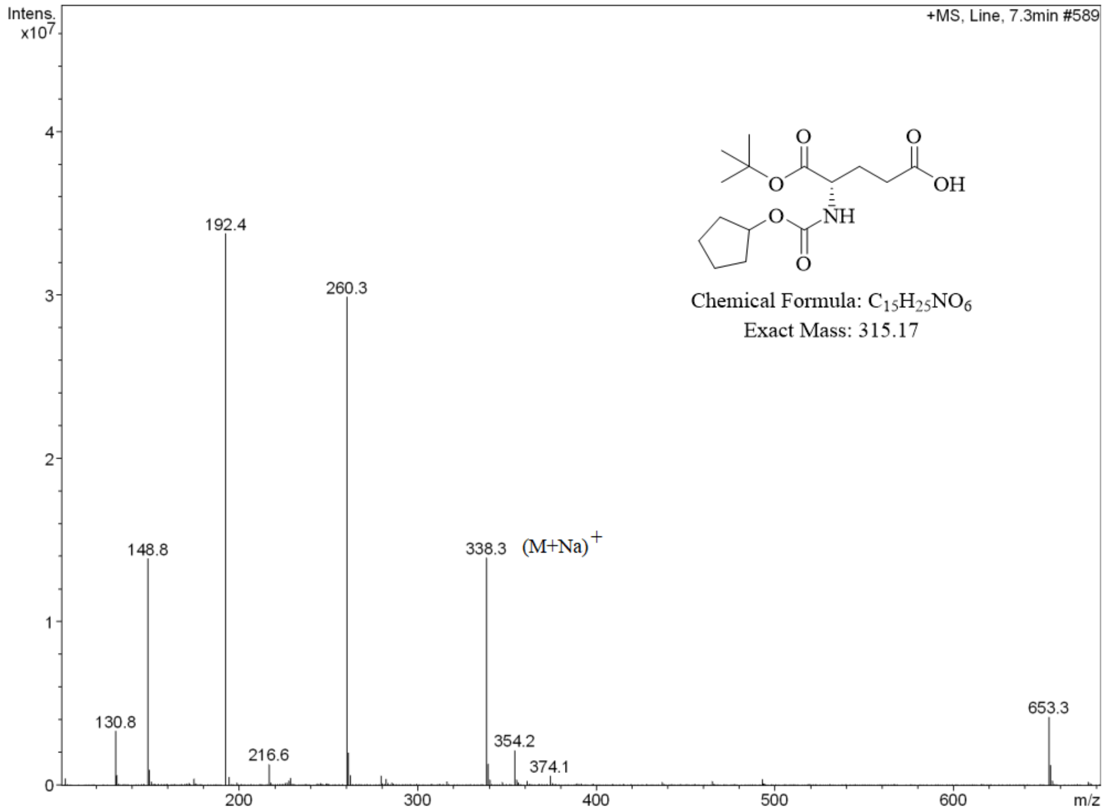

Fig. S83. MS of intermediate **14g**

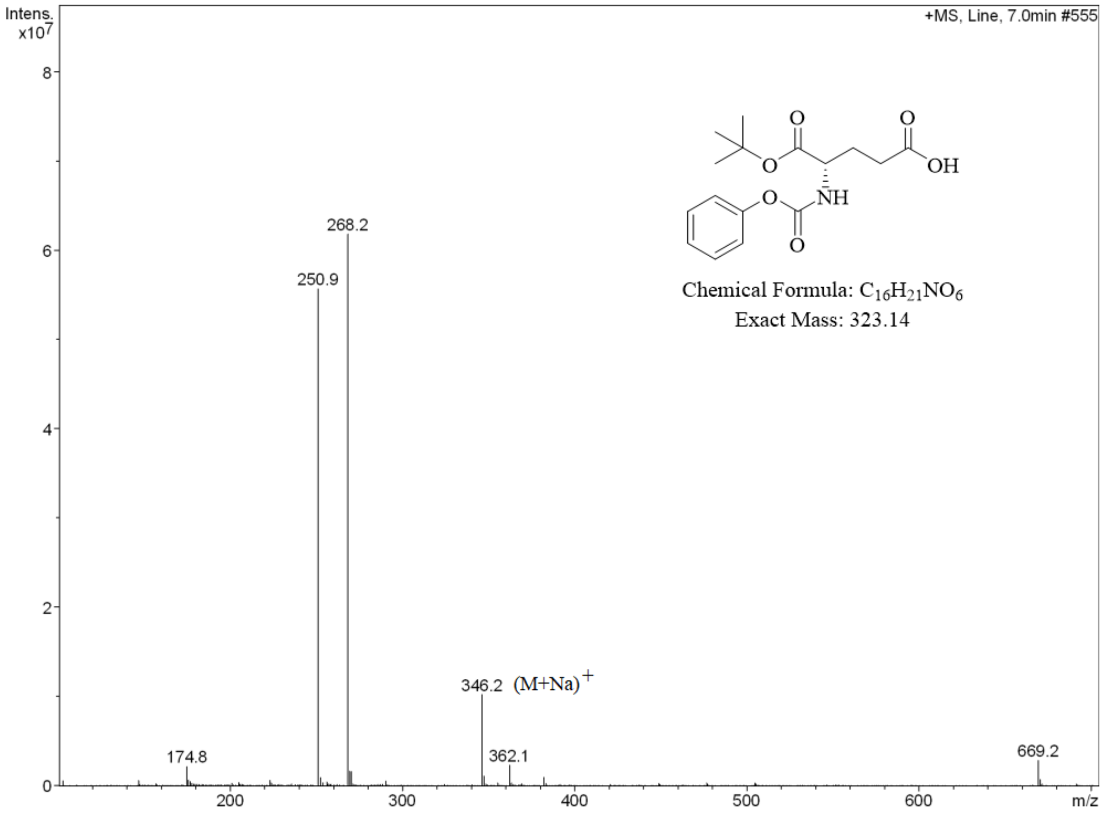

Fig. S84. MS of intermediate **14h**

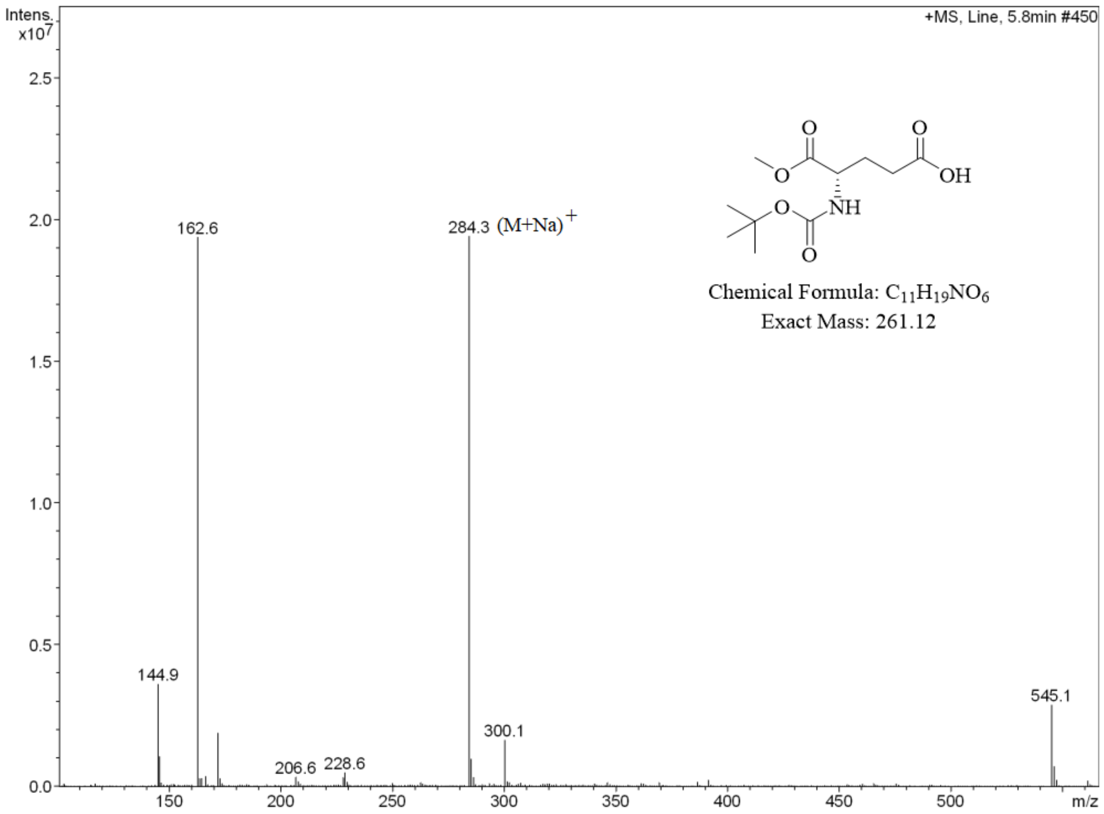

Fig. S85. MS of intermediate **18a**

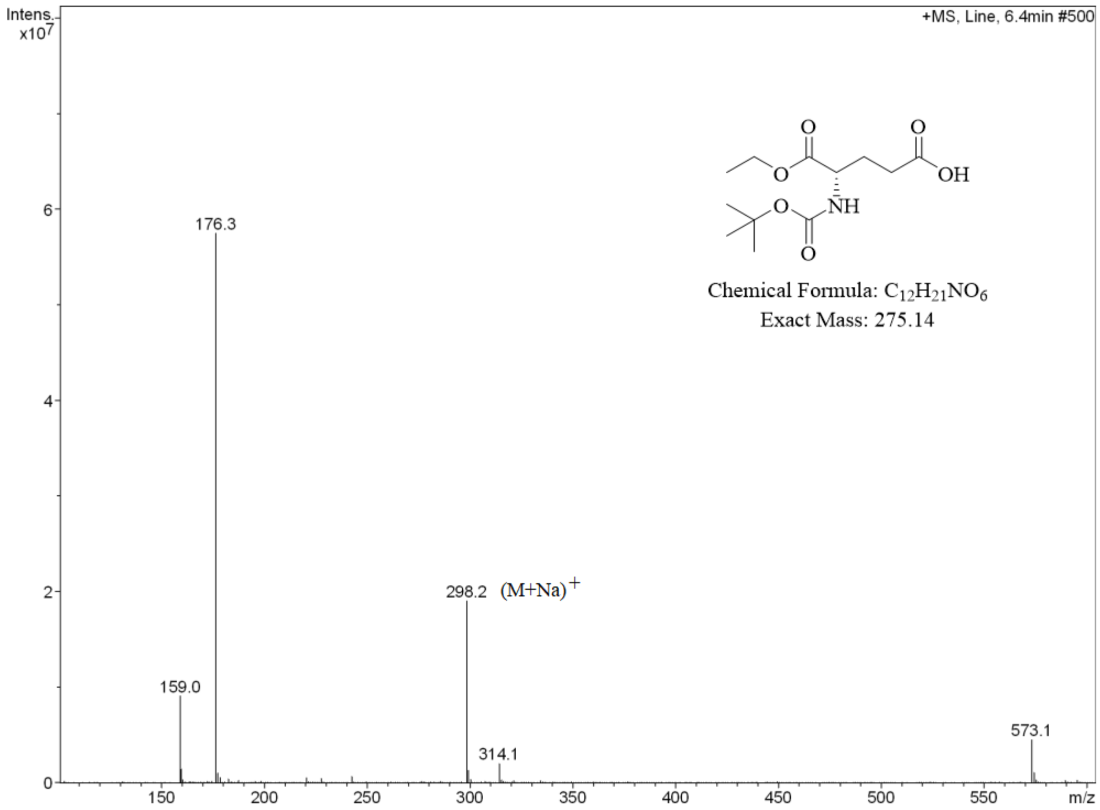

Fig. S86. MS of intermediate **18b**

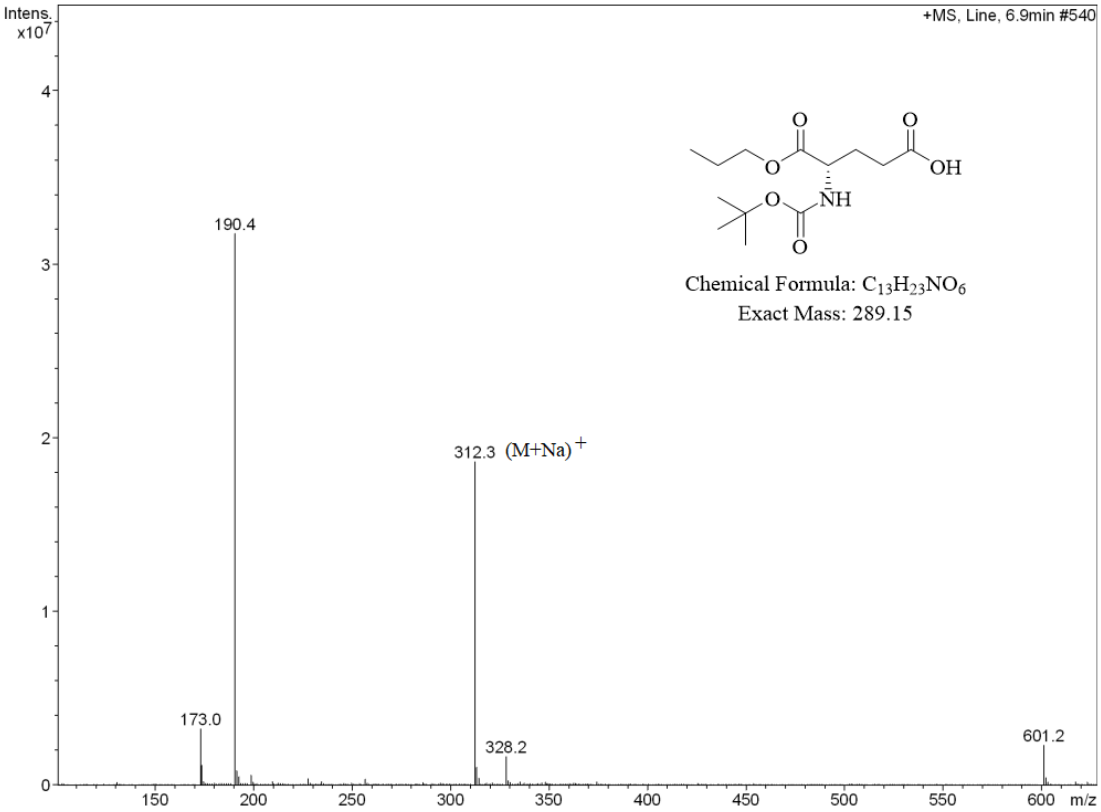

Fig. S87. MS of intermediate **18c**

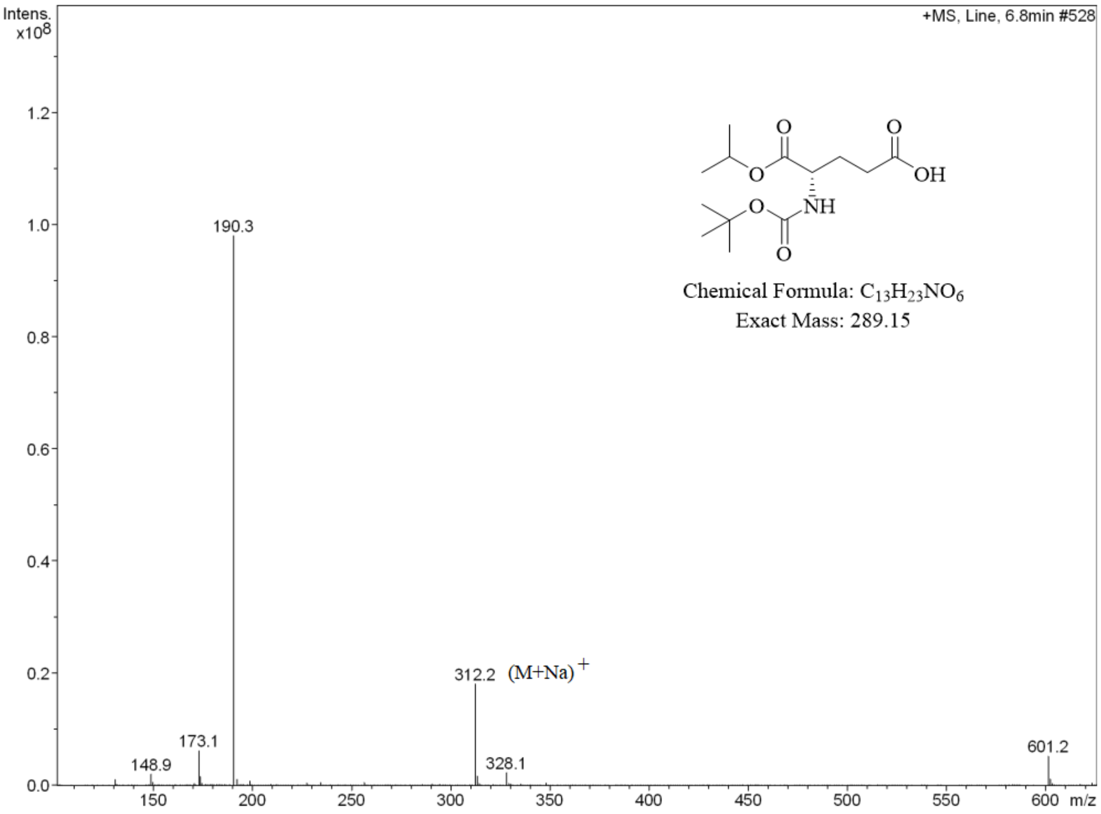

Fig. S88. MS of intermediate **18d**

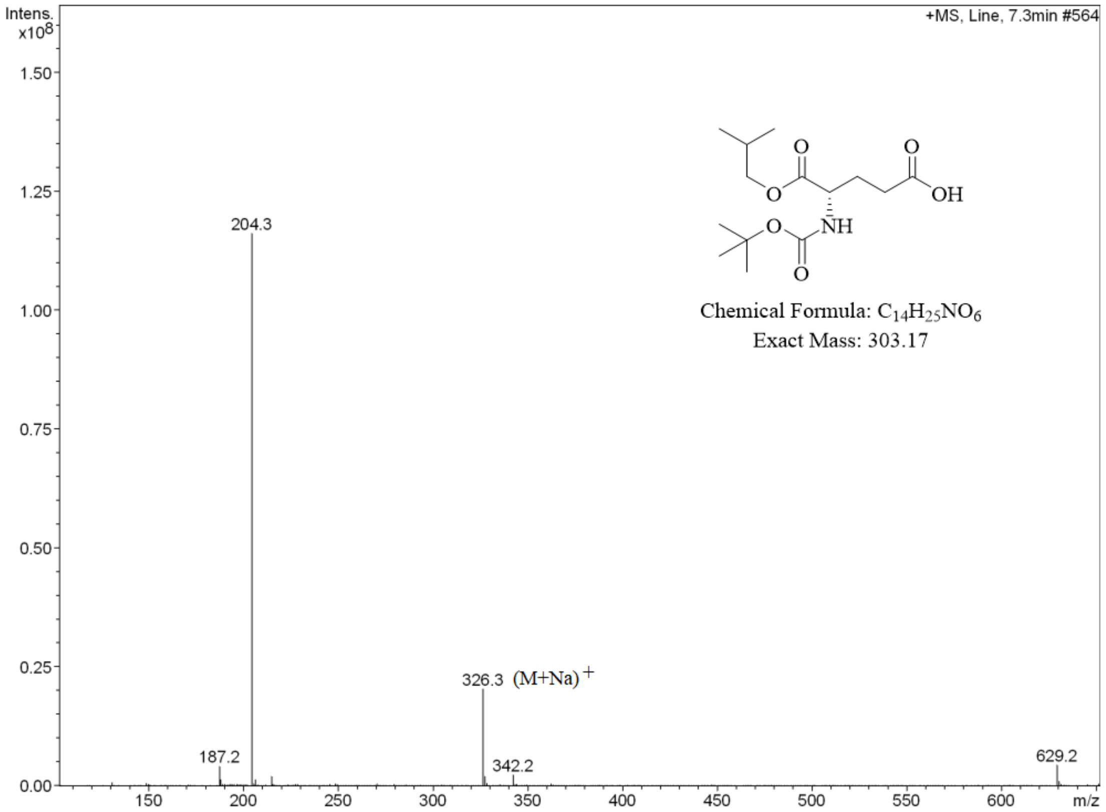

Fig. S89. MS of intermediate **18e**

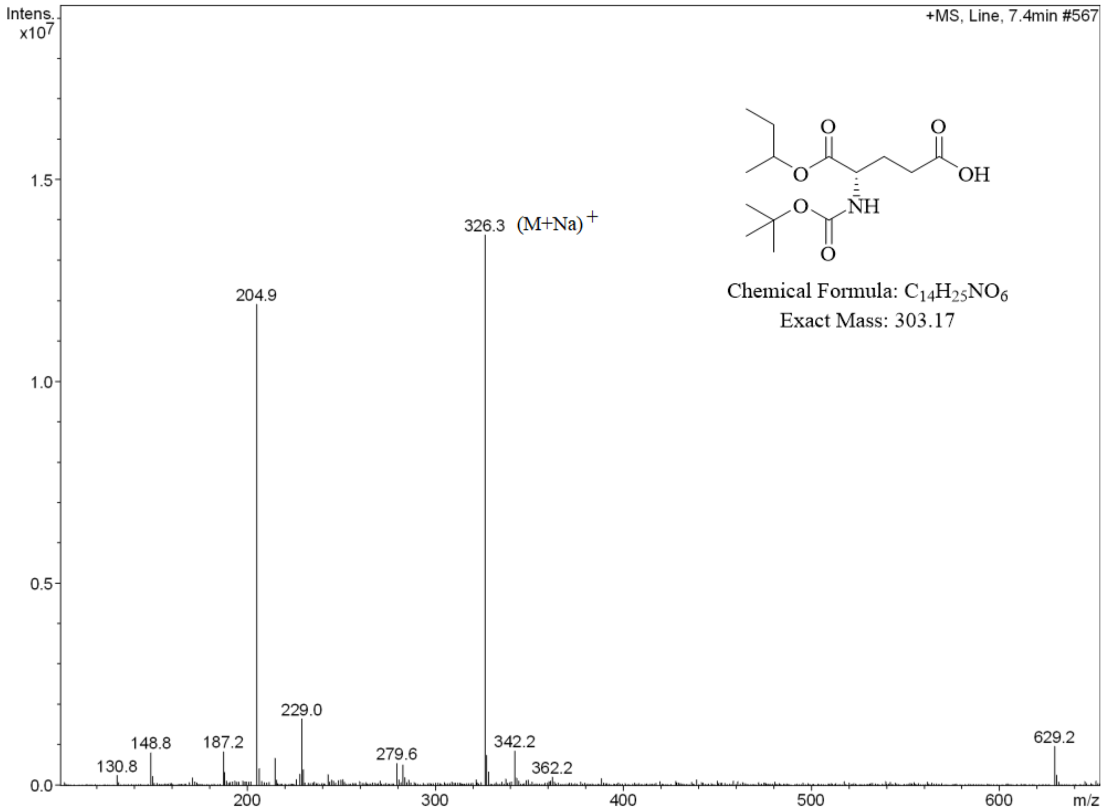

Fig. S90. MS of intermediate **18f**

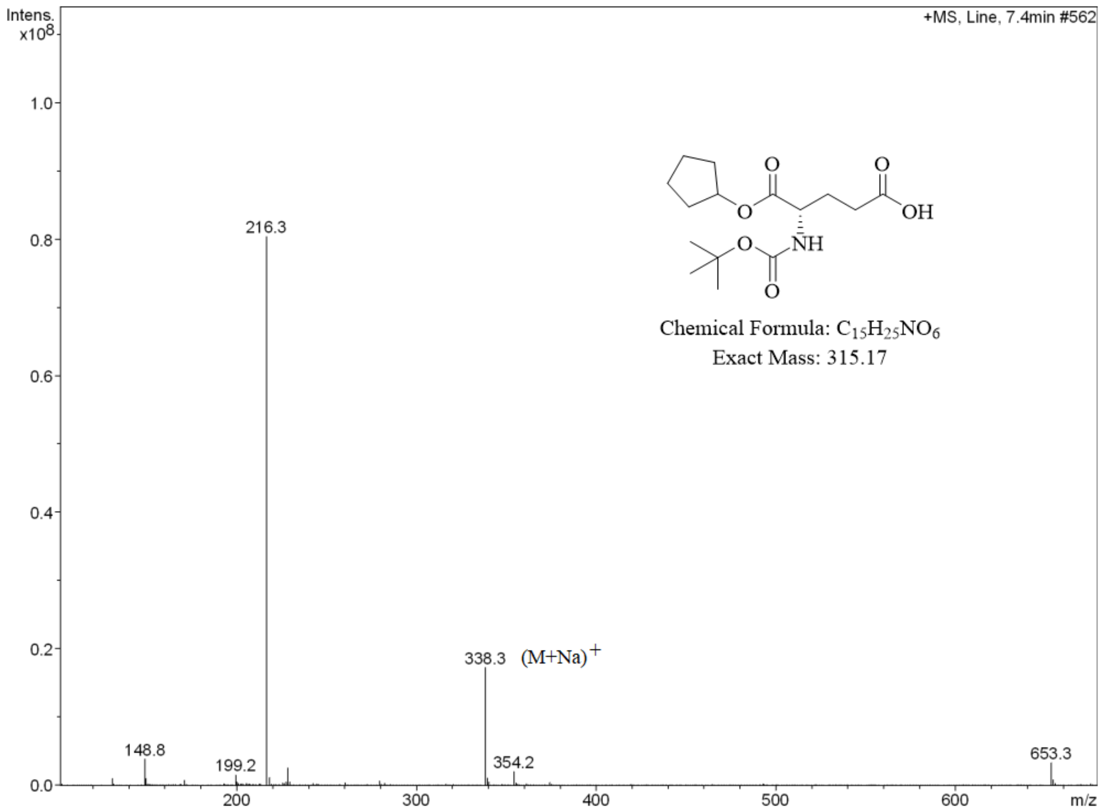

Fig. S91. MS of intermediate **18g**

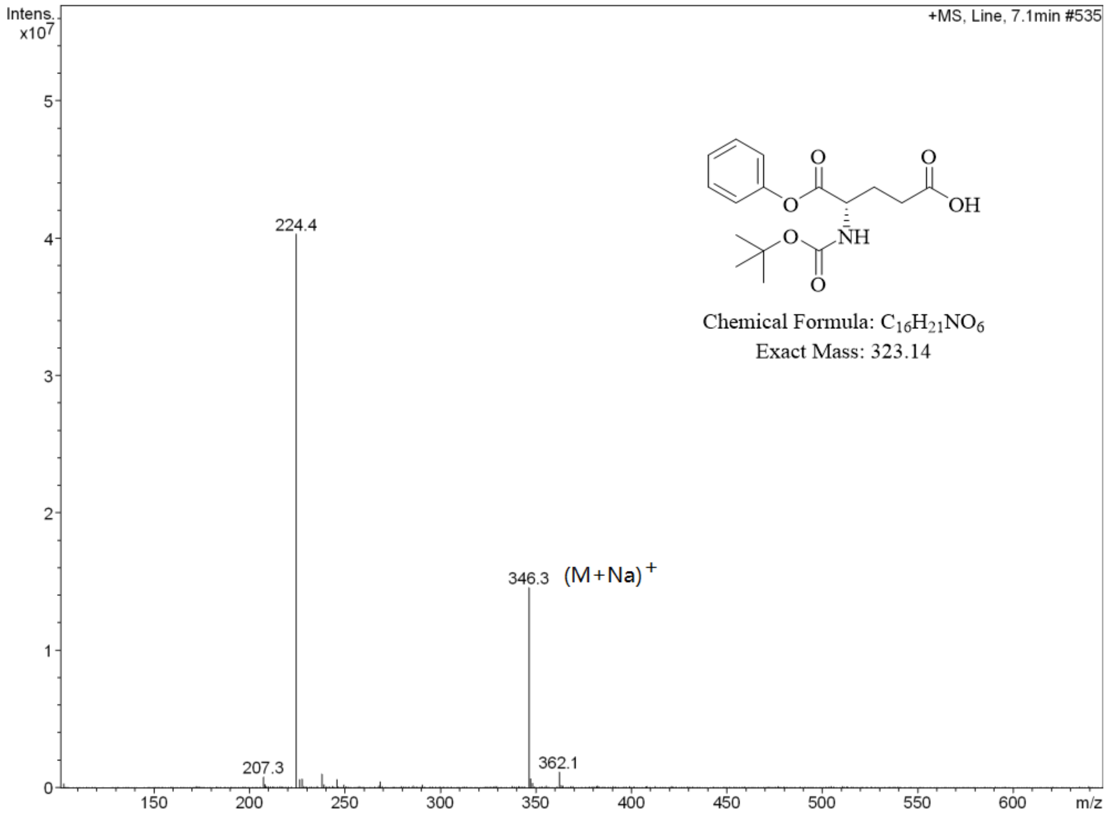

Fig. S92. MS of intermediate **18h**

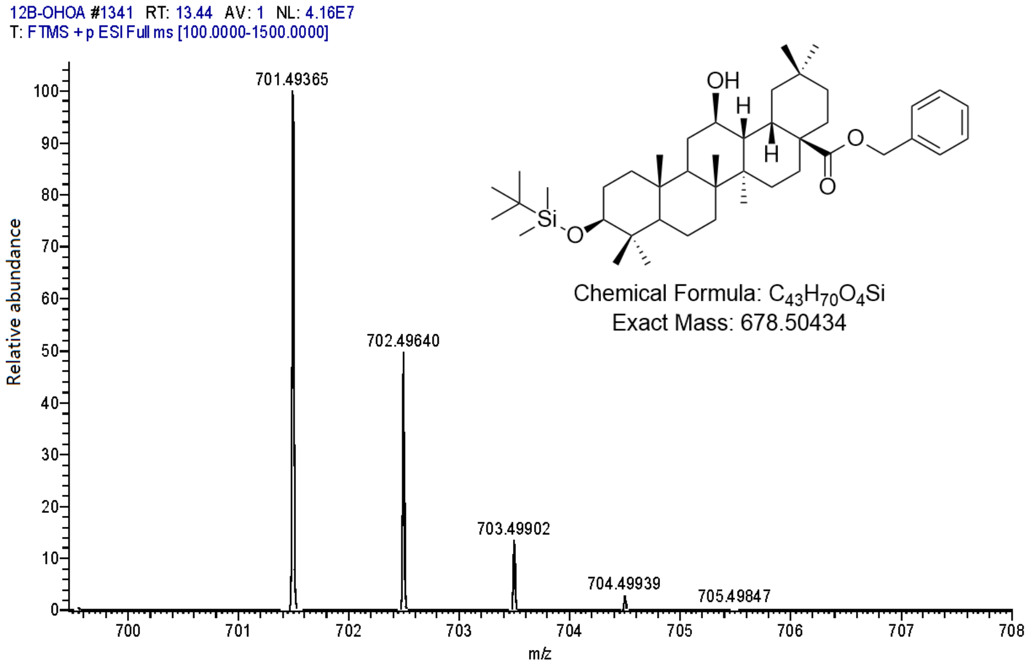

m/z	Theo. Mass	Delta (ppm)	RDB equiv.	Composition	
701.49365	701.49356	0.092	9.5	C43 H70 O4 Na Si	M+Na

Fig. S93. HRMS of compound **8**

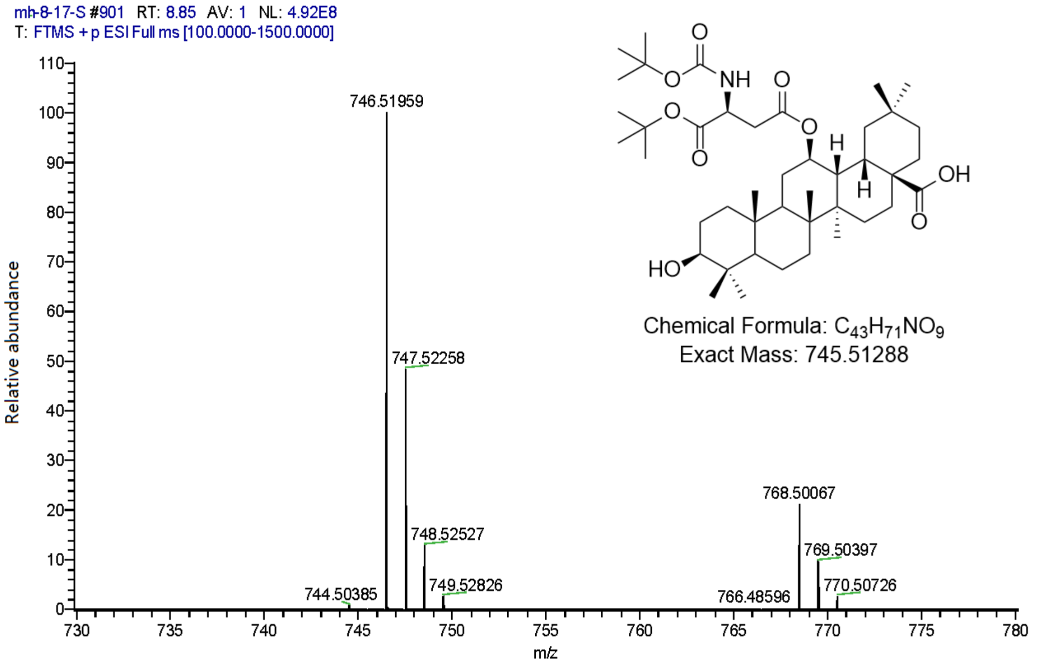

m/z	Theo. Mass	Delta (ppm)	RDB equiv.	Composition	
746.51959	746.52016	-0.569	8.5	C43 H72 O9 N	M+H

Fig. S94. HRMS of compound **9a**

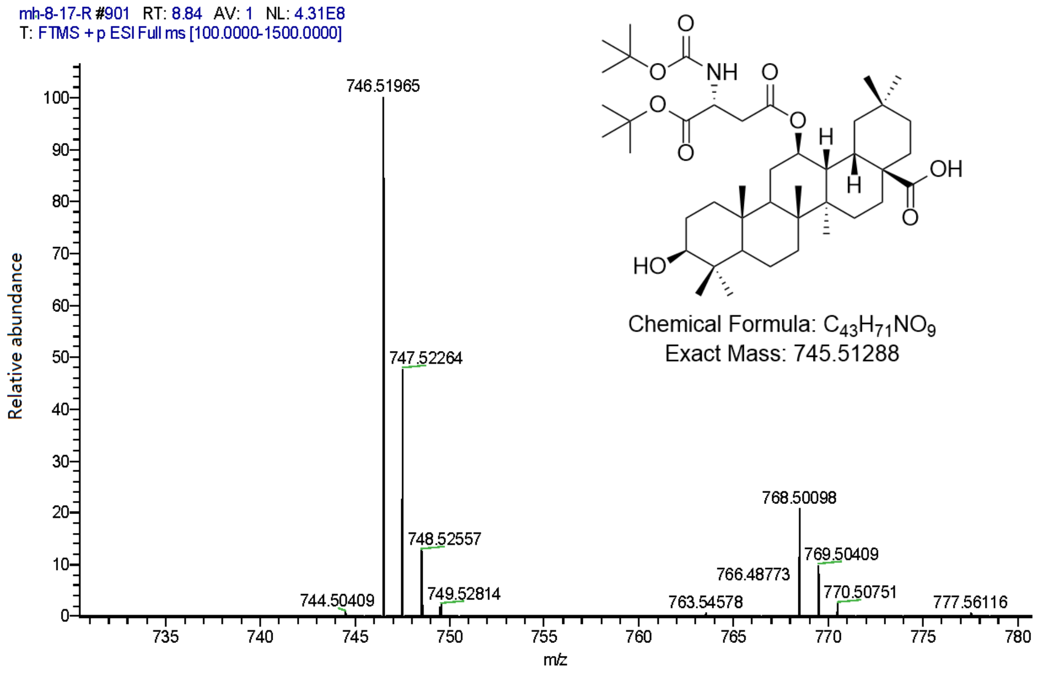

m/z	Theo. Mass	Delta (ppm)	RDB equiv.	Composition	
746.51965	746.52016	-0.509	8.5	C43 H72 O9 N	M+H

Fig. S95. HRMS of compound **9b**

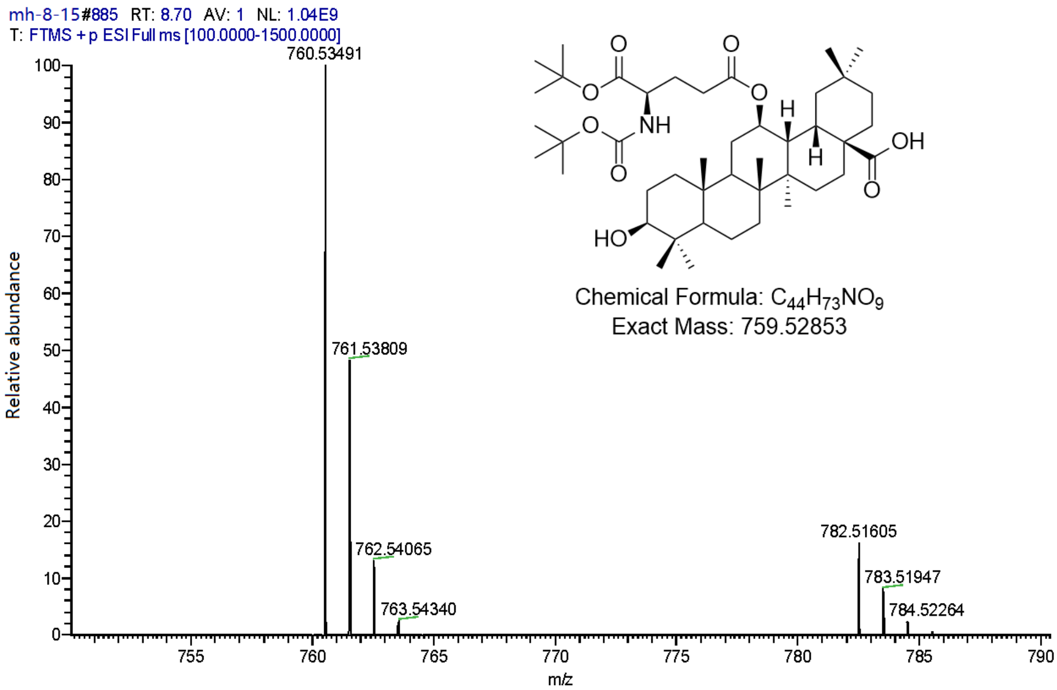

m/z	Theo. Mass	Delta (ppm)	RDB equiv.	Composition	
760.53491	760.53581	-0.899	8.5	C44 H74 O9 N	M+H

Fig. S96. HRMS of compound **10a**

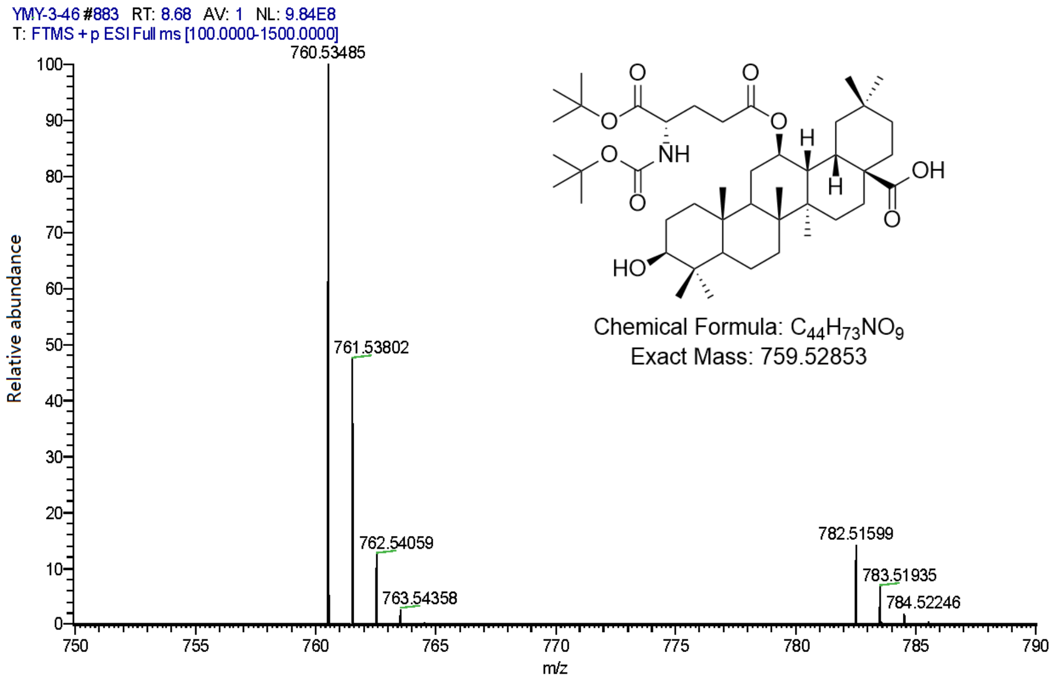

m/z	Theo. Mass	Delta (ppm)	RDB equiv.	Composition	
760.53485	760.53581	-0.959	8.5	C44 H74 O9 N	M+H

Fig. S97. HRMS of compound **10b**

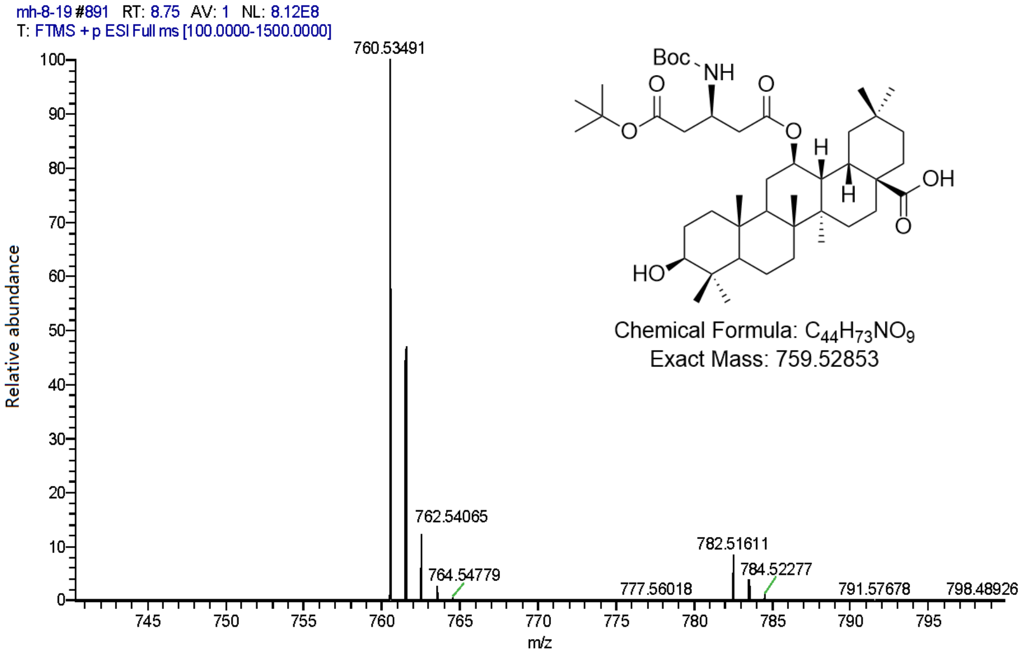

m/z	Theo. Mass	Delta (ppm)	RDB equiv.	Composition	
760.53491	760.53581	-0.899	8.5	C44 H74 O9 N	M+H

Fig. S98. HRMS of compound **10c**

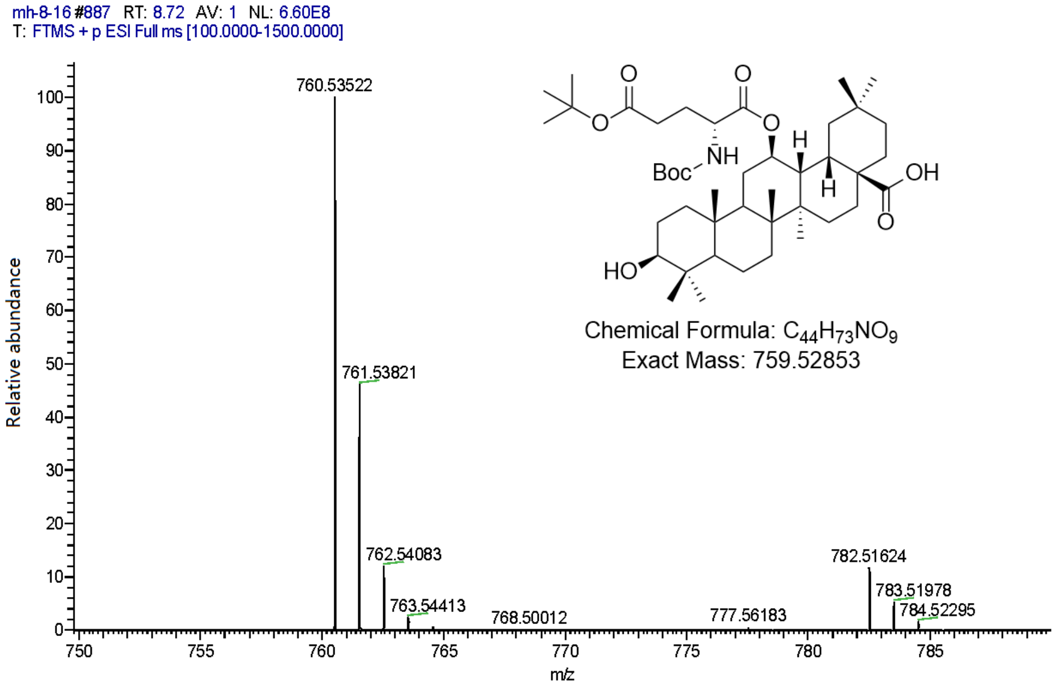

m/z	Theo. Mass	Delta (ppm)	RDB equiv.	Composition	
760.53522	760.53581	-0.589	8.5	C44 H74 O9 N	M+H

Fig. S99. HRMS of compound **10d**

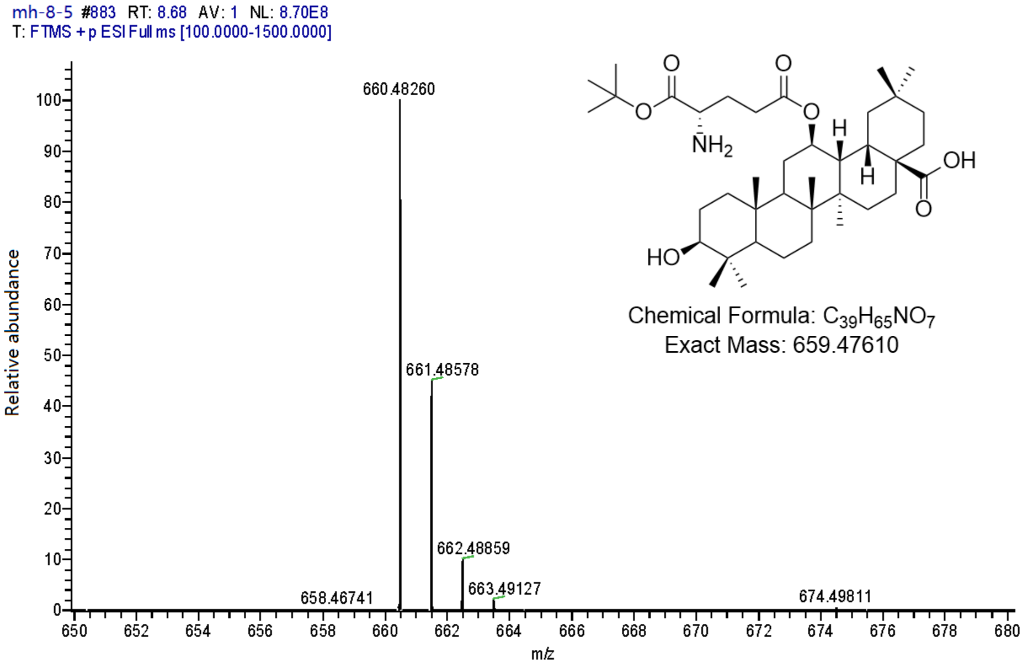

m/z	Theo. Mass	Delta (ppm)	RDB equiv.	Composition	
660.48260	660.48338	-0.78	7.5	C39 H66 O7 N	M+H

Fig. S100. HRMS of compound **11a**

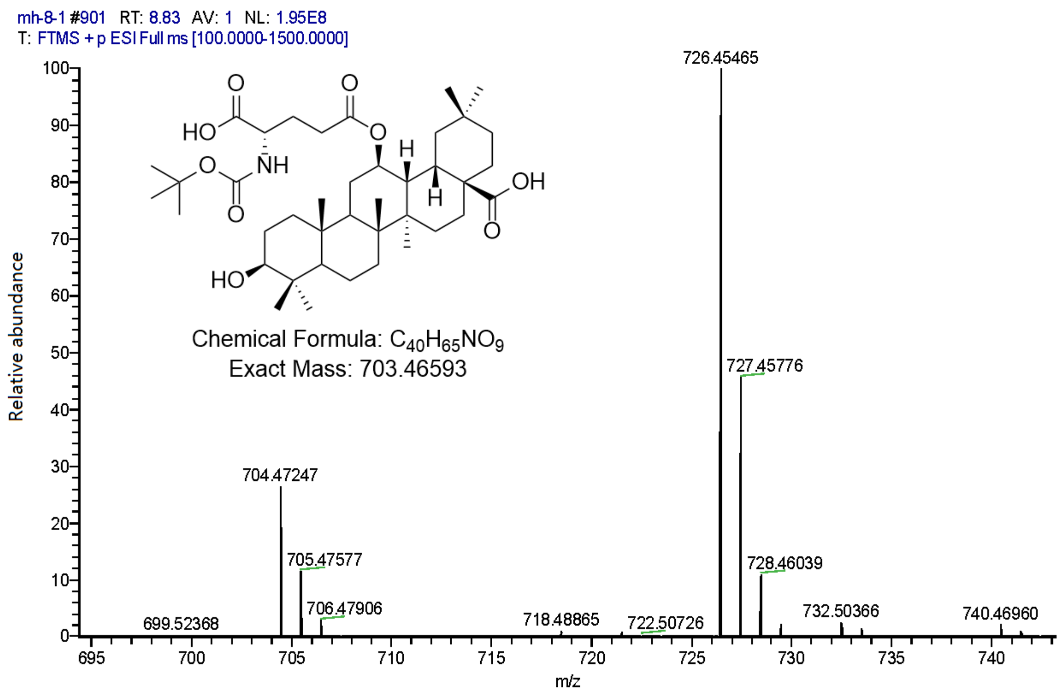

m/z	Theo. Mass	Delta (ppm)	RDB equiv.	Composition	
726.45465	726.45515	-0.504	8.5	C40 H65 O9 N Na	M+Na

Fig. S101. HRMS of compound **11b**

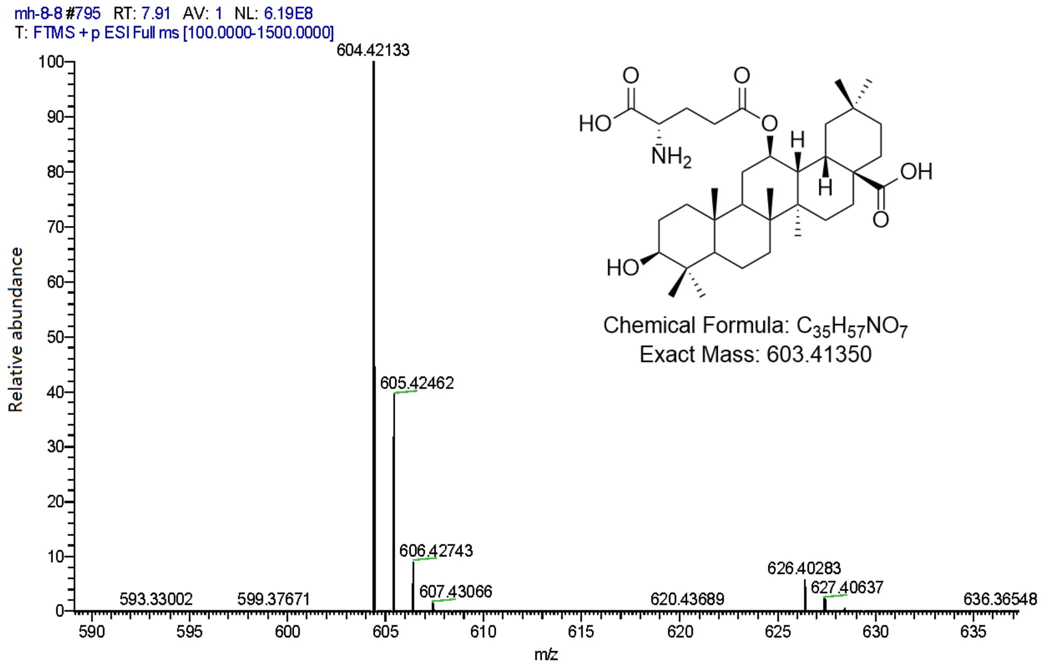

m/z	Theo. Mass	Delta (ppm)	RDB equiv.	Composition	
604.42133	604.42078	0.55	7.5	C35 H58 O7 N	M+H

Fig. S102. HRMS of compound **11c**

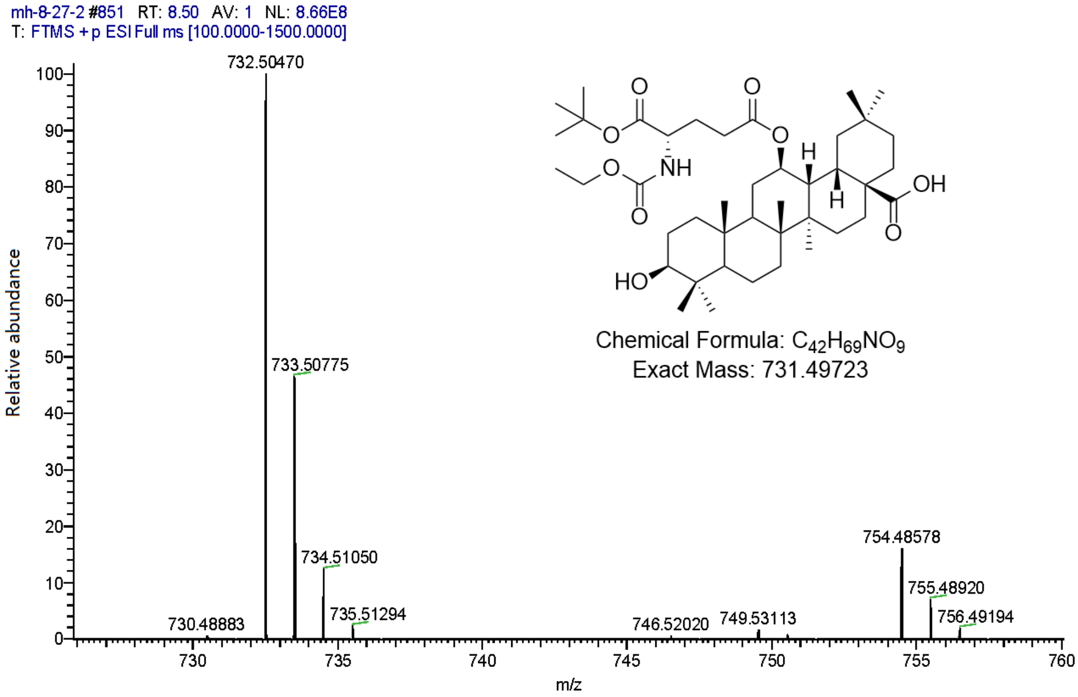

m/z	Theo. Mass	Delta (ppm)	RDB equiv.	Composition	
732.50470	732.50451	0.191	8.5	C42 H70 O9 N	M+H

Fig. S103. HRMS of compound **19b**

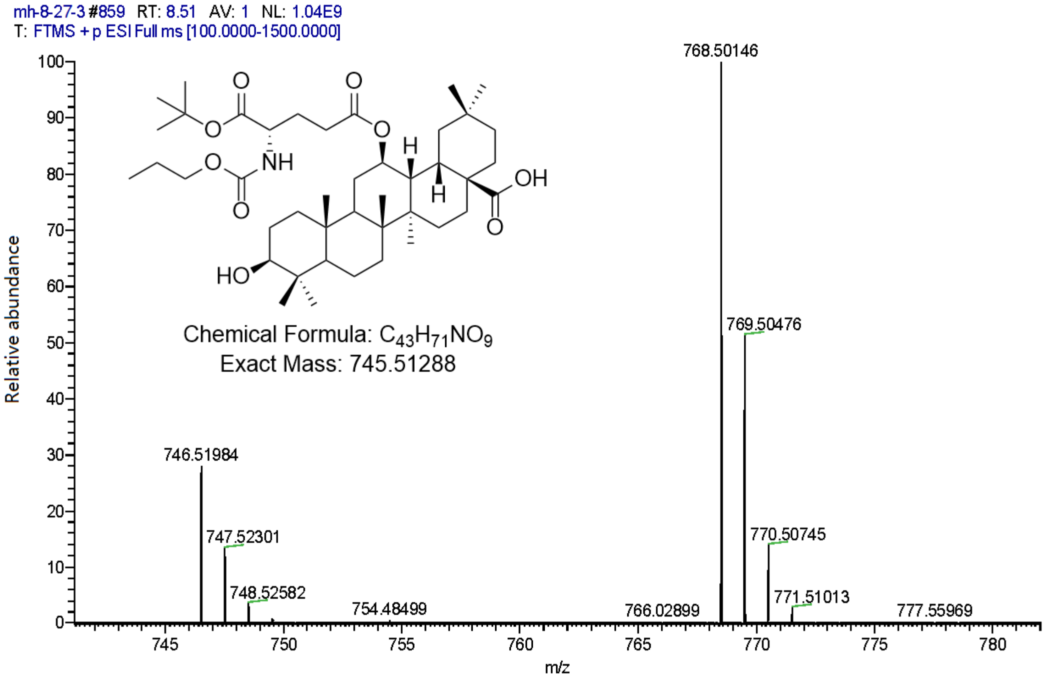

m/z	Theo. Mass	Delta (ppm)	RDB equiv.	Composition	
768.50146	768.50210	-0.644	8.5	C43 H71 O9 N Na	M+Na

Fig. S104. HRMS of compound **19c**

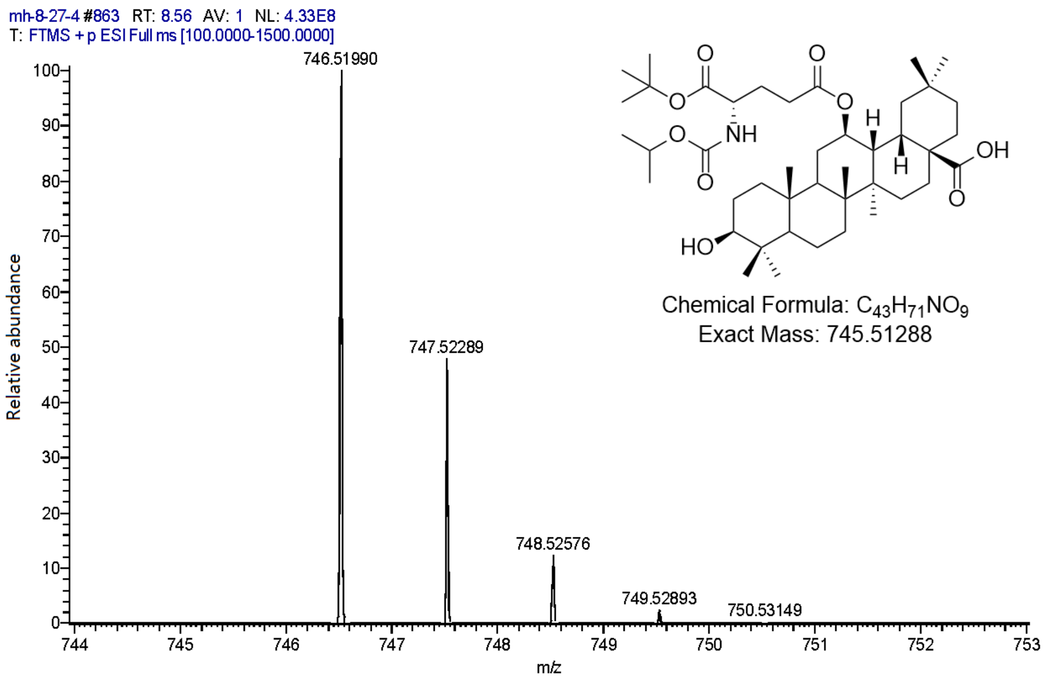

m/z	Theo. Mass	Delta (ppm)	RDB equiv.	Composition	
746.51990	746.52016	-0.259	8.5	C43 H72 O9 N	M+H

Fig. S105. HRMS of compound **19d**

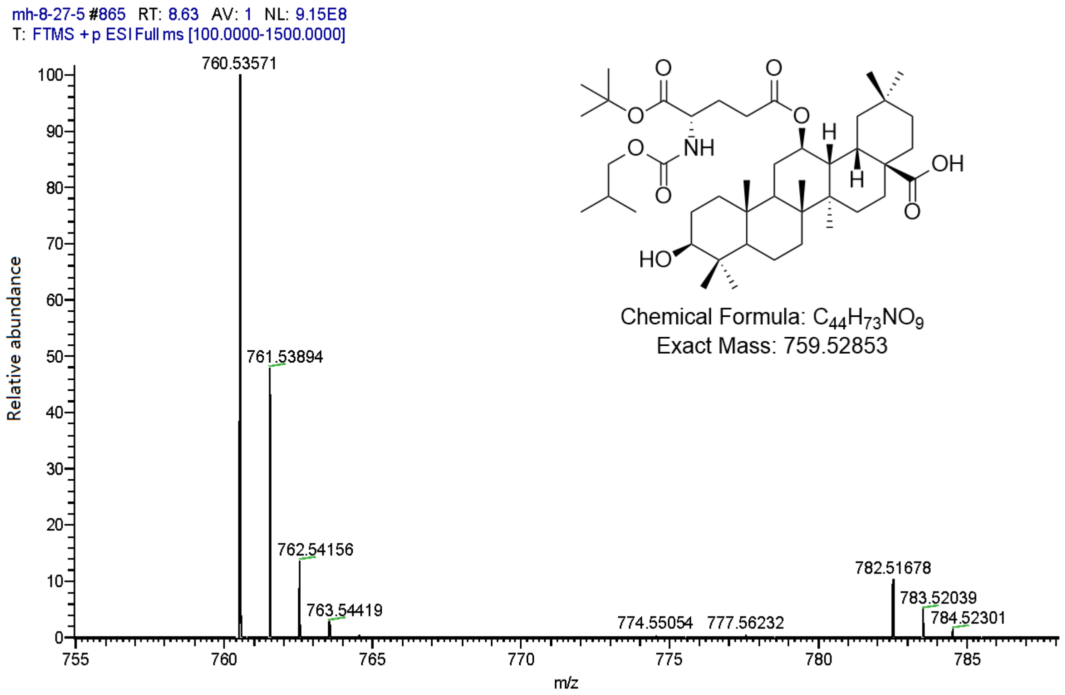

m/z	Theo. Mass	Delta (ppm)	RDB equiv.	Composition	
760.53571	760.53581	-0.099	8.5	C44 H74 O9 N	M+H

Fig. S106. HRMS of compound **19e**

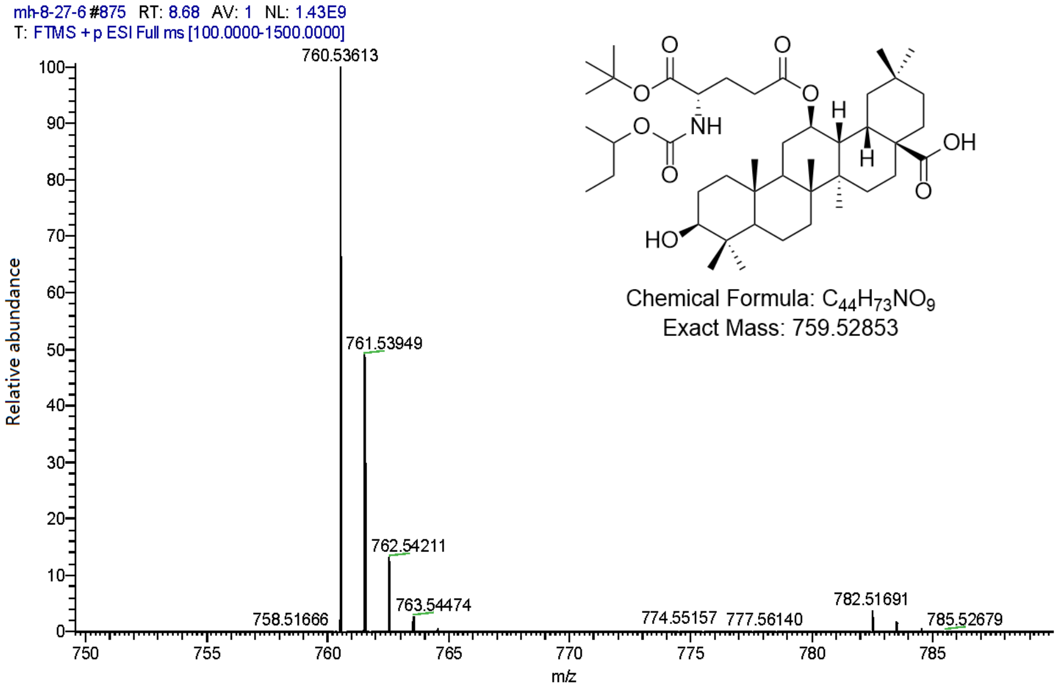

m/z	Theo. Mass	Delta (ppm)	RDB equiv.	Composition	
760.53613	760.53581	0.321	8.5	C44 H74 O9 N	M+H

Fig. S107. HRMS of compound **19f**

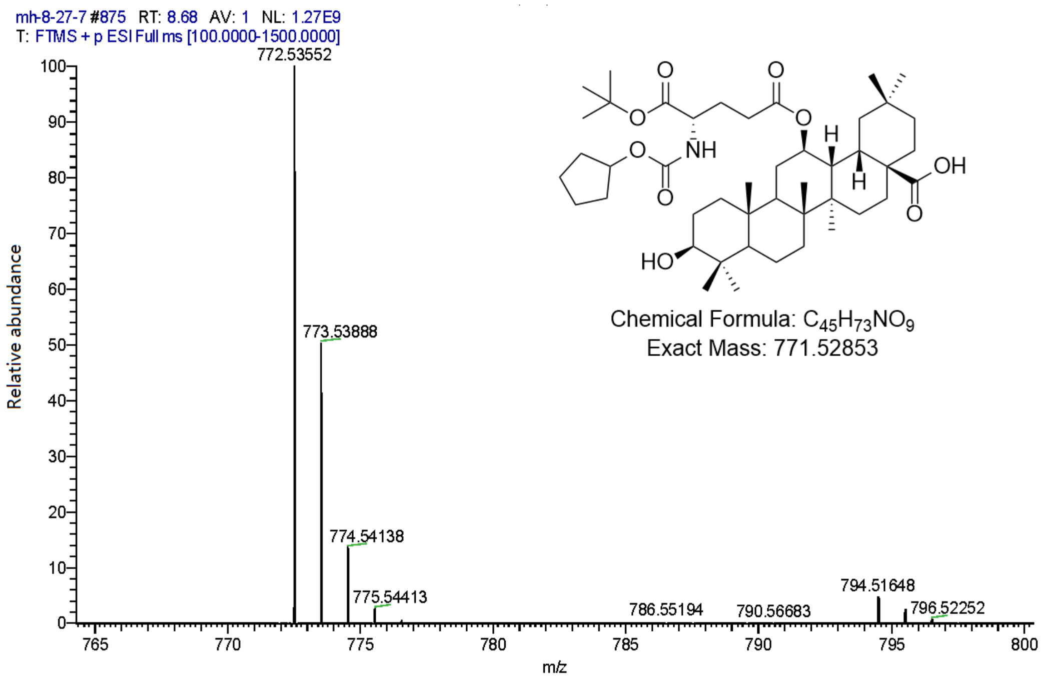

m/z	Theo. Mass	Delta (ppm)	RDB equiv.	Composition	
772.53552	772.53581	-0.289	9.5	C45 H74 O9 N	M+H

Fig. S108. HRMS of compound **19g**

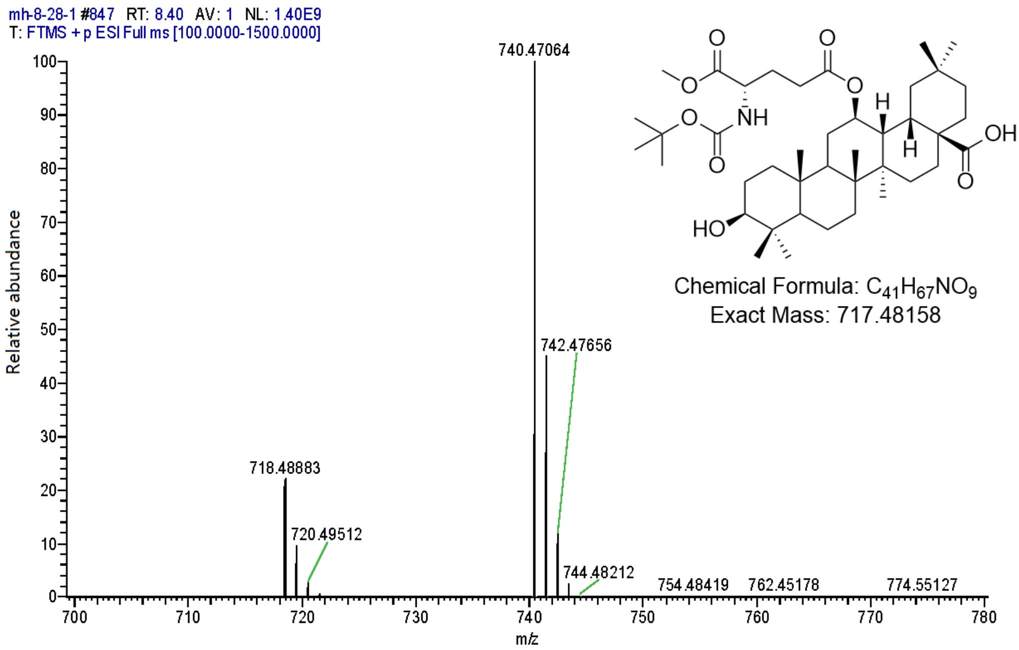

m/z	Theo. Mass	Delta (ppm)	RDB equiv.	Composition	
740.47064	740.47080	-0.164	8.5	C41 H67 O9 N Na	M+Na

Fig. S109. HRMS of compound **20a**

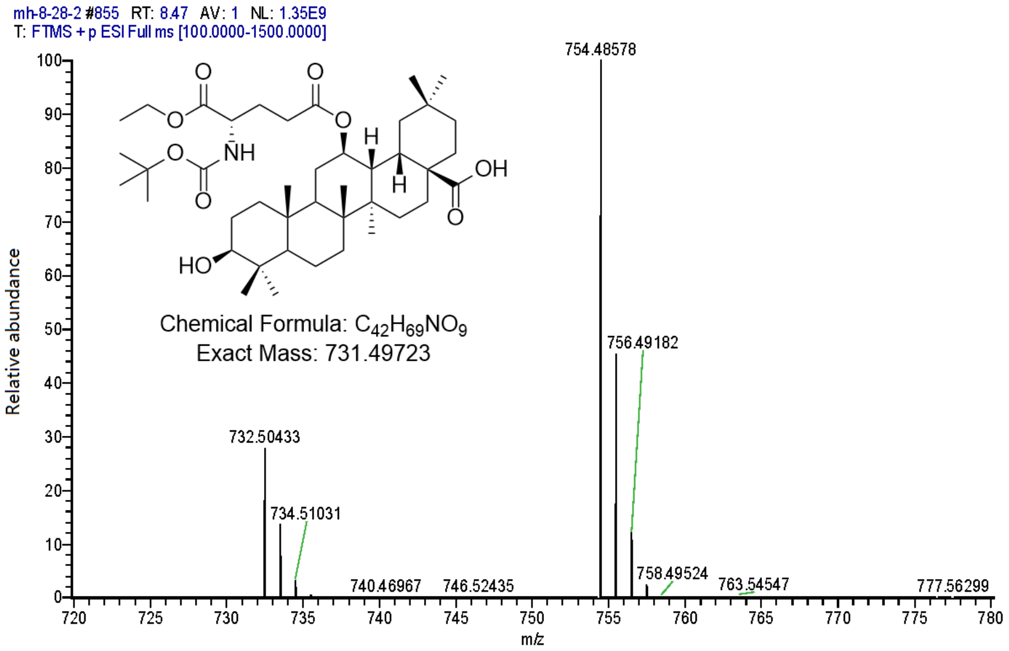

m/z	Theo. Mass	Delta (ppm)	RDB equiv.	Composition	
754.48578	754.48645	-0.674	8.5	C42 H69 O9 N Na	M+Na

Fig. S110. HRMS of compound **20b**

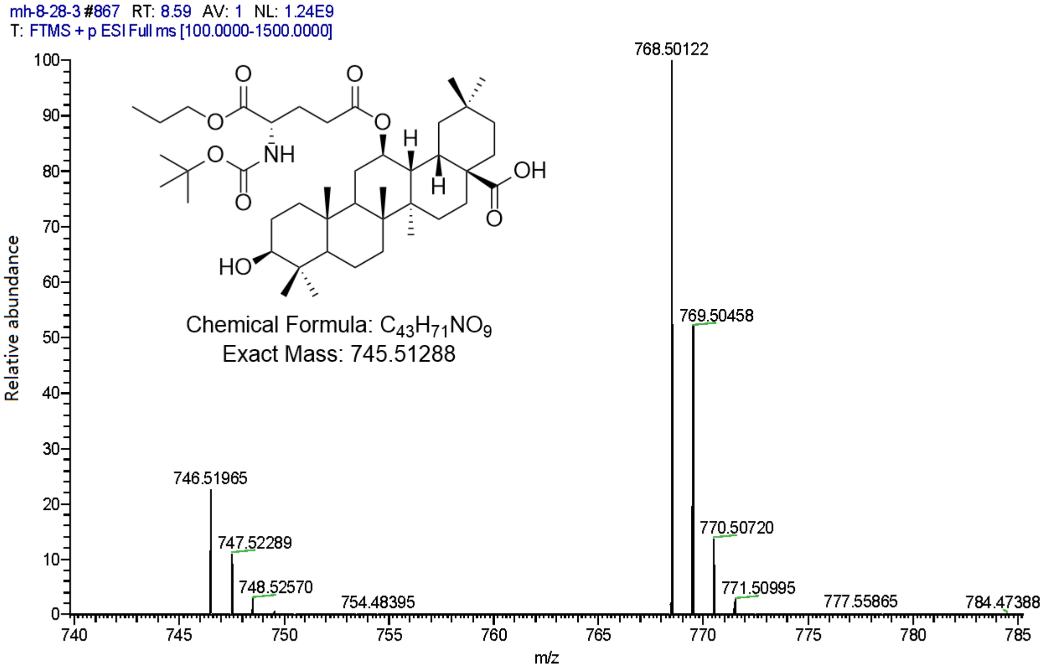

m/z	Theo. Mass	Delta (ppm)	RDB equiv.	Composition	
768.50122	768.50210	-0.884	8.5	C43 H71 O9 N Na	M+Na

Fig. S111. HRMS of compound **20c**

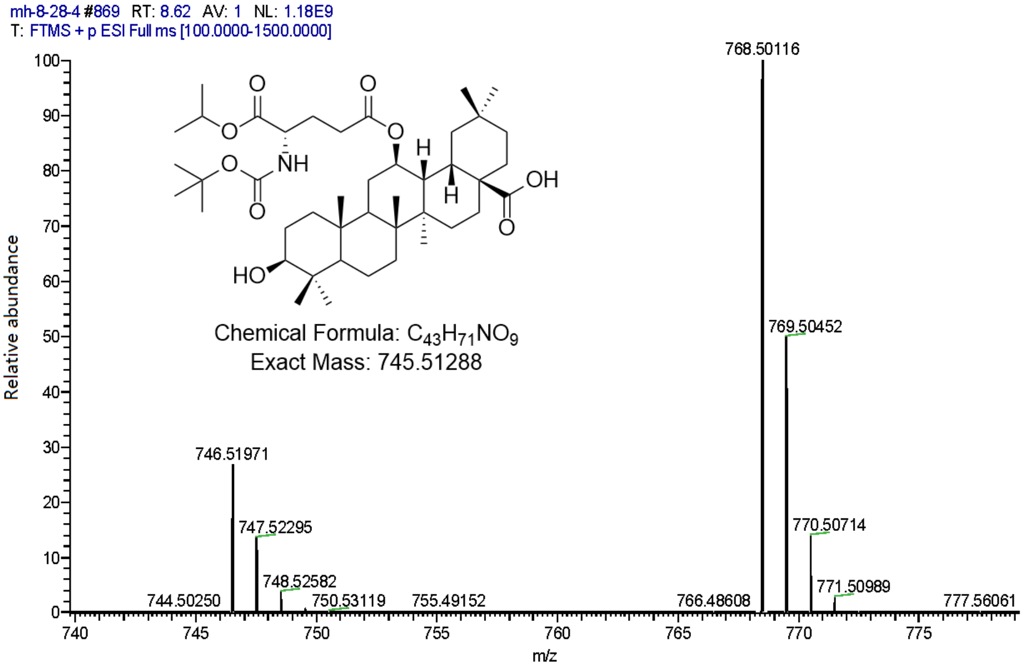

m/z	Theo. Mass	Delta (ppm)	RDB equiv.	Composition	
768.50116	768.50210	-0.944	8.5	C43 H71 O9 N Na	M+Na

Fig. S112. HRMS of compound **20d**

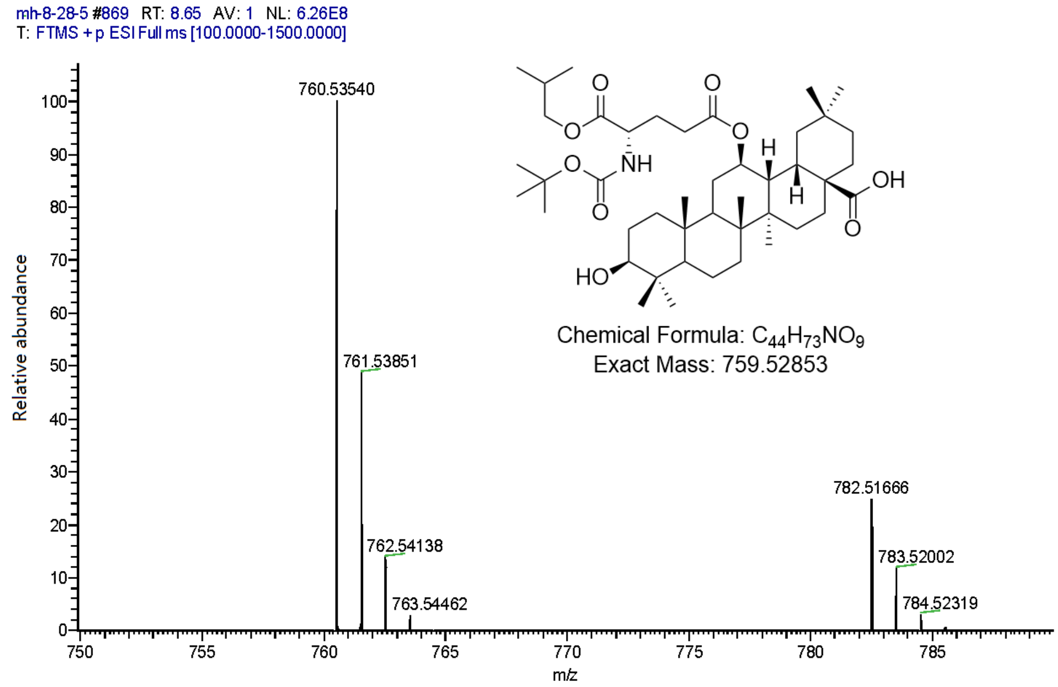

m/z	Theo. Mass	Delta (ppm)	RDB equiv.	Composition	
760.53540	760.53581	-0.409	8.5	C44 H74 O9 N	M+H

Fig. S113. HRMS of compound **20e**

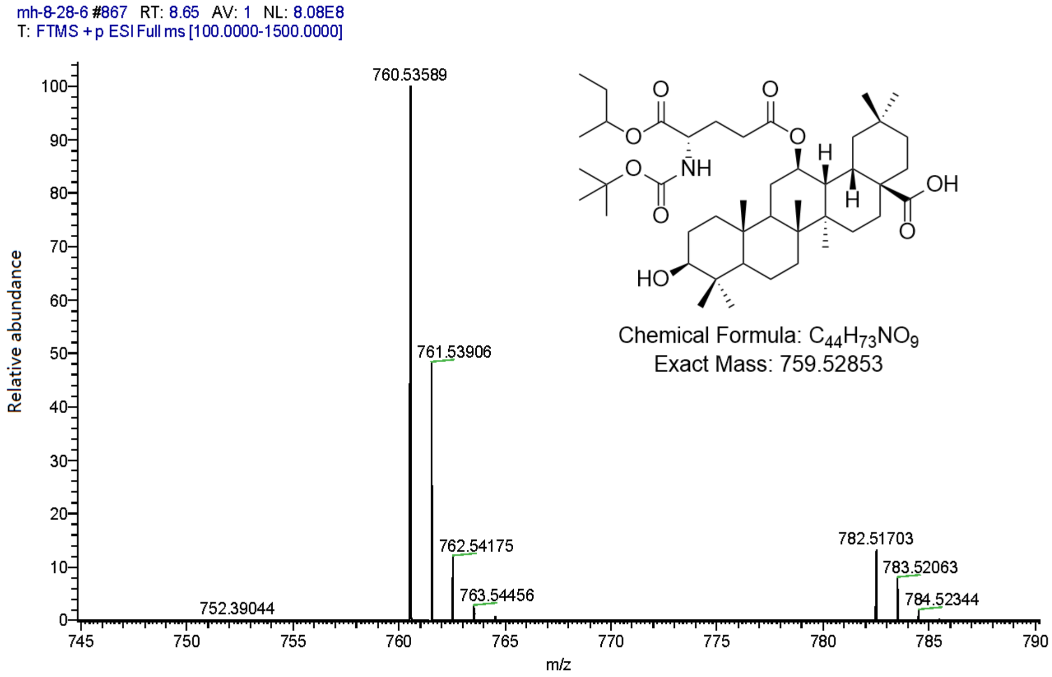

m/z	Theo. Mass	Delta (ppm)	RDB equiv.	Composition	
760.53589	760.53581	0.081	8.5	C44 H74 O9 N	M+H

Fig. S114. HRMS of compound **20f**

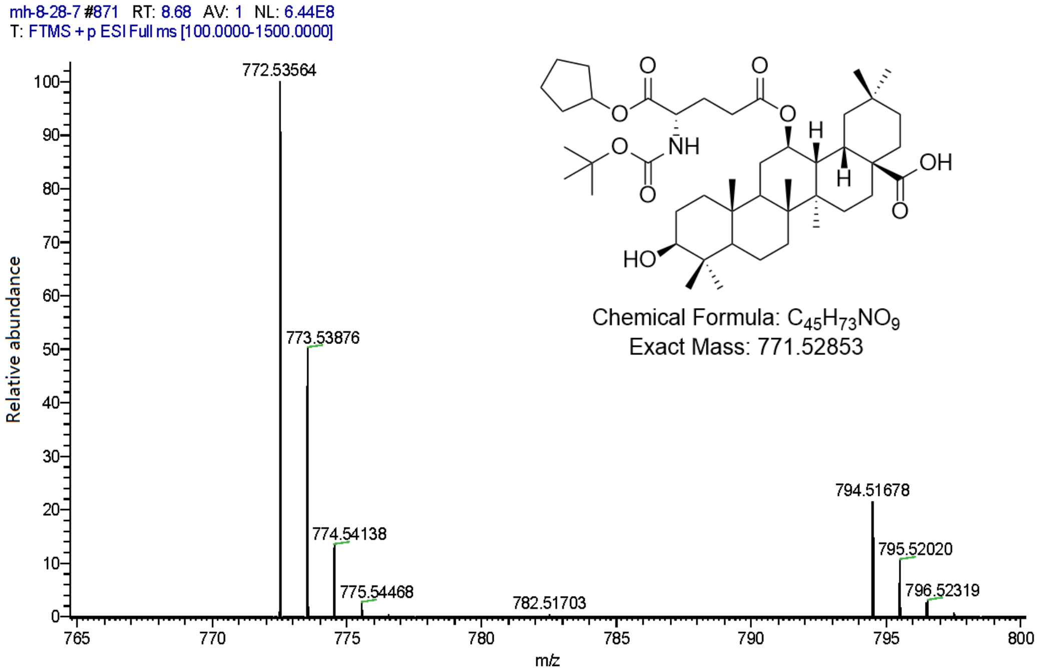

m/z	Theo. Mass	Delta (ppm)	RDB equiv.	Composition	
772.53564	772.53581	-0.169	9.5	C45 H74 O9 N	M+H

Fig. S115. HRMS of compound **20g**
